# Thirty years of research on physical activity, mental health, and wellbeing: A scientometric analysis of hotspots and trends

**DOI:** 10.3389/fpubh.2022.943435

**Published:** 2022-08-09

**Authors:** Michel Sabe, Chaomei Chen, Othman Sentissi, Jeroen Deenik, Davy Vancampfort, Joseph Firth, Lee Smith, Brendon Stubbs, Simon Rosenbaum, Felipe Barreto Schuch, Marco Solmi

**Affiliations:** ^1^Division of Adult Psychiatry, Department of Psychiatry, University Hospitals of Geneva, Thonex, Switzerland; ^2^College of Computing and Informatics, Drexel University, Philadelphia, PA, United States; ^3^Scientific Research Department, GGz Centraal, Amersfoort, Netherlands; ^4^School for Mental Health and Neuroscience, Maastricht University, Maastricht, Netherlands; ^5^Katholieke Universiteit Leuven Department of Rehabilitation Sciences, Leuven, Belgium; ^6^University Psychiatric Center Katholieke Universiteit Leuven, Leuven, Belgium; ^7^Division of Psychology and Mental Health, Manchester Academic Health Science Centre, University of Manchester, Manchester, United Kingdom; ^8^Greater Manchester Mental Health National Health Service Foundation Trust, Manchester Academic Health Science Centre, Manchester, United Kingdom; ^9^Centre for Health, Performance and Wellbeing, Anglia Ruskin University, Cambridge, United Kingdom; ^10^Physiotherapy Department, South London and Maudsley National Health Service Foundation Trust, London, United Kingdom; ^11^Department of Psychological Medicine, Institute of Psychiatry, Psychology and Neuroscience, King's College London, London, United Kingdom; ^12^Discipline of Psychiatry and Mental Health, Medicine and Health, University of New South Wales, Kensington, NSW, Australia; ^13^School of Health Sciences, Medicine and Health, University of New South Wales, Kensington, NSW, Australia; ^14^Department of Sports Methods and Techniques, Federal University of Santa Maria, Santa Maria, Brazil; ^15^Department of Psychiatry, University of Ottawa, Ottawa, ON, Canada; ^16^Department of Mental Health, The Ottawa Hospital, Ottawa, ON, Canada; ^17^Ottawa Hospital Research Institute, Clinical Epidemiology Program, University of Ottawa, Ottawa, ON, Canada; ^18^Department of Child and Adolescent Psychiatry, Charité Universitätsmedizin, Berlin, Germany

**Keywords:** physical exercise, mental illness, evidence synthesis, scientometrics, CiteSpace

## Abstract

The sheer volume of research publications on physical activity, mental health, and wellbeing is overwhelming. The aim of this study was to perform a broad-ranging scientometric analysis to evaluate key themes and trends over the past decades, informing future lines of research. We searched the Web of Science Core Collection from inception until December 7, 2021, using the appropriate search terms such as “physical activity” or “mental health,” with no limitation of language or time. Eligible studies were articles, reviews, editorial material, and proceeding papers. We retrieved 55,353 documents published between 1905 and 2021. The annual scientific production is exponential with a mean annual growth rate of 6.8% since 1989. The 1988–2021 co-cited reference network identified 50 distinct clusters that presented significant modularity and silhouette scores indicating highly credible clusters (*Q* = 0.848, *S* = 0.939). This network identified 6 major research trends on physical activity, namely cardiovascular diseases, somatic disorders, cognitive decline/dementia, mental illness, athletes' performance, related health issues, and eating disorders, and the COVID-19 pandemic. A focus on the latest research trends found that greenness/urbanicity (2014), concussion/chronic traumatic encephalopathy (2015), and COVID-19 (2019) were the most active clusters of research. The USA research network was the most central, and the Chinese research network, although important in size, was relatively isolated. Our results strengthen and expand the central role of physical activity in public health, calling for the systematic involvement of physical activity professionals as stakeholders in public health decision-making process.

## Introduction

Physical activity can be considered as medicine and has been used in both the treatment and prevention of a variety of chronic conditions (1). Longitudinal cohort studies demonstrate that a low cardiorespiratory fitness constitutes the largest attributable fraction for all-cause mortality (2). There is also overwhelming evidence that low physical activity (i.e., not meeting physical activity recommendations) is considered as an important risk factor for chronic conditions including some cancers, cardiovascular disease, diabetes, dementia, and in particular for a patient with mental illness (schizophrenia, bipolar disorder, or major depressive disorder) (3–5). Patients with mental illness have poor physical health compared with the general population, with reduced life expectancy and a higher risk of premature death beyond suicide, from natural causes (6). At least partially, among other factors, their poor physical health is due to higher sedentary behavior and lower physical activity compared with the general population (7, 8). Physical activity, and its structured form of exercise, seem to affect the brain and mind, beyond physical health, both as a factor associated with poor mental health and quality of life and as a treatment for mental disorders (9). Indeed, exercise has shown to be efficacious in a number of mental disorders, according to a previous umbrella review pooling 27 systematic reviews (10, 11). Exercise is also now seen as a potential preventive or disease-modifying treatment of dementia and brain aging (12) or as a possible treatment for negative symptoms in schizophrenia (13).

Importantly, systematic reviews, meta-analysis, and umbrella reviews have offered a deep synthesis of specific research questions addressed within the exponential volume of physical activity literature related to mental health and wellbeing. However, such systematic methods may not be appropriate to encompass hundreds or thousands of new publications per year. In fact, systematic reviews have to be narrow in their inclusion criteria and offer a comprehensive view on a specific and restricted research or clinical question. For instance, a meta-analysis can inform if an intervention is efficacious for a given population on an outcome of interest (14, 15) or an umbrella review can assess the credibility of an association between a risk factor and an incident condition (16–19). Nevertheless, none of the two offers an insight on the temporal trend of research, the complex network of topics, authors, publications, networks, institutions, and their bibliometric performance. Gaining such overarching views of how an entire field of research on a particular topic is important and useful, in order to gauge how the academic literature is developing and inform the next steps for the science to pursue.

The integration of developments in data visualization, text mining, and network analysis has permitted the emergence of a new framework and a new generation of research synthesis of both evidence and influence, named research weaving (20). This framework combines visual analytics and scientometrics to visualize and delineate the development of a field, its underlying intellectual structure and the dynamics of scholarly communication over time (21). A comprehensive delineation of how scientometrics and bibliometrics overlap and distinct can be found in Hood and Wilson 2001 paper (22).

To the best of our knowledge, no broad-ranging scientometric study of research trends and influence networks of physical activity, mental health and wellbeing has yet been conducted. Thus, in this article, we present one to bridge the gap.

## Materials and methods

### Search strategy and data collection

We searched the Web of Science Core Collection (WOSCC) on December 7, 2021, using a combination of keywords and Medical Subject Headings such as “physical activity,” “mental health,” and “mental illness^*^.” WOSCC provides full references and complete citations of articles published in major journals since 1900 and is one of the largest comprehensive sources for bibliometric studies (23). The full protocol with the search key is available on osf.io. This current study protocol is based on a first large-scale scientometric analysis (24). The database source was limited to the Web of Science Citation Index Expanded. The document types are limited to “article,” “review,” “editorial material,” and “proceeding papers,” without restrictions on language or time. The dataset was extracted from the WOSCC in tag-delimited plain text files.

**Figure 1:**
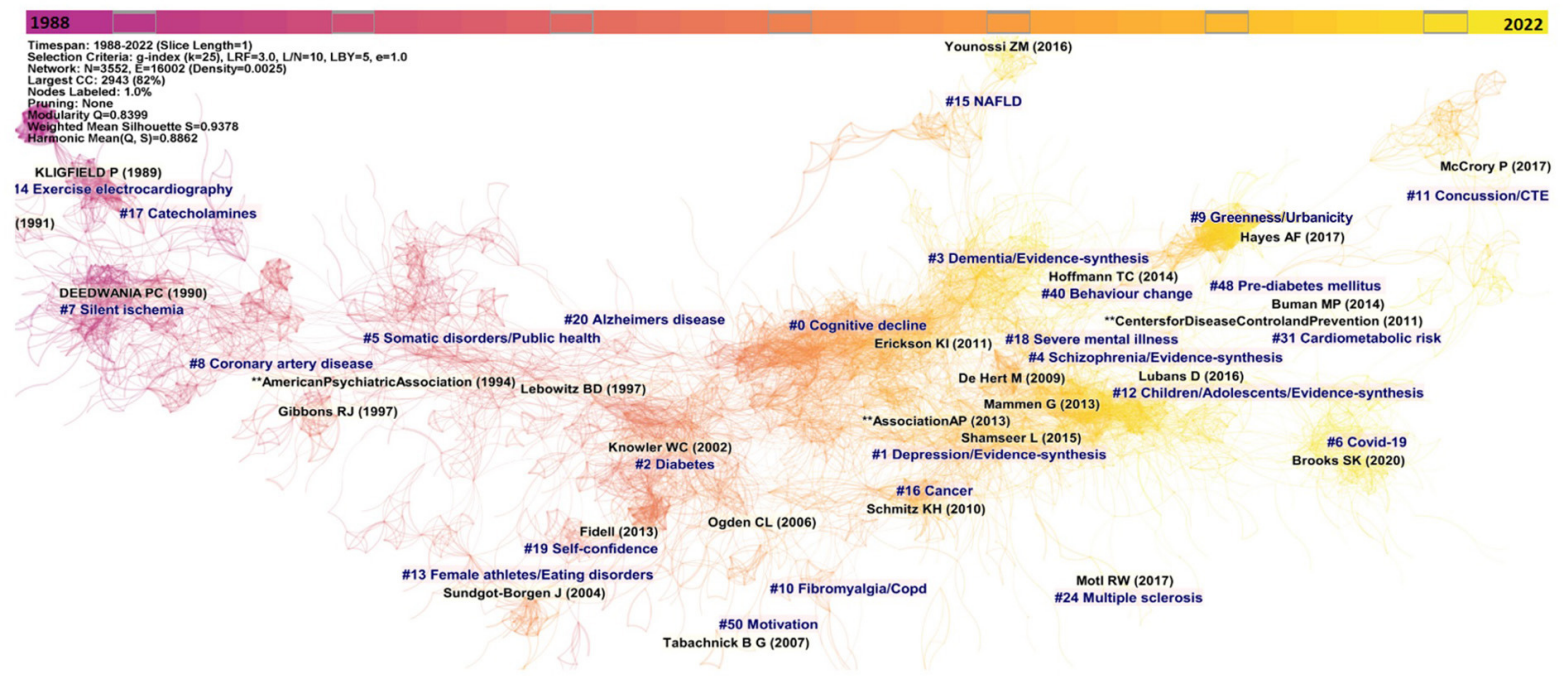
Co-citation reference network with cluster visualization (1988–2021). The unit of measure are articles and constitutes nodes. Nodes are organized according to year of publication. The size of a node (article) is proportional to the number of times the node has been co-cited. Colored shades indicate the passage of the time, from past (purplish) to the present time (yellowish).

In order to assess the quality of the reference filtering process and the homogeneity of the dataset, we independently inspected each of the most cited references (604 articles in total), and a randomly selected sample of 10% of included articles to allow a margin of error (i.e., inclusion of non-relevant papers) of 5% with a 95% confidence interval (Supplementary Table 1; Figure 1).

### Objectives

The primary outcome was to visualize research trends on physical activity related to mental health and wellbeing and to characterize the evolution of research trends using networks of co-cited references and networks of co-occurring keywords assigned to relevant publications.

The secondary outcome was to provide clinicians, researchers, and policymakers with a specific unit of measure of the research network (countries, institutions, authors, and journals) and to identify emerging trends and limitations.

### Data analysis

Two different software tools for constructing bibliometric networks were used: Bibliometrix R package (3.1.4) (25) and CiteSpace (version 5.8.R4) (21). Bibliometric outcomes included citation counts, co-citations, and co-occurrences. A co-citation count is defined as the frequency with which two published articles are cited together by subsequently published articles (26). Co-occurrence networks are based on how frequently two entities, such as keywords, appear in the same articles.

The Bibliometrix R package was used for the analysis of publication outputs and the trend of growth. CiteSpace was used for the study of several types of networks, namely, networks of co-cited references, networks of co-cited authors, and co-occurrence networks of authors, keywords, institutions, and countries. For instance, the co-cited (authors') institutions network accounts for the cooperation between two or more institutions, which reflects the cooperation between authors and the influence networks.

CiteSpace produces a variety of metrics of significance, with temporal metrics such as citation burstness, structural metrics such as betweenness centrality, modularity, and silhouette score as well as a combination of both, namely, the sigma metric. The betweenness centrality of a node measures the fraction of shortest paths in an underlying network passing through the node (27). The burstness of the frequency of an entity over time indicates a specific duration of a surge of the frequency (28). The sigma indicator combines structural and temporal properties of a node, namely, its betweenness centrality and citation burst (29). Modularity (the Q score) measures the quality of dividing a network into clusters, and the silhouette score (the S score) of a cluster measures the quality of a clustering configuration (30). The Q score ranges from 0 to +1. The cluster structure is considered significant with a Q score >0.3, and higher values indicate a well-structured network. The S score ranges from −1 to +1. If the S score is >0.3, 0.5, or 0.7, the network is considered homogenous, reasonable, or highly credible, respectively. In addition, we conducted a structural variation analysis that focuses on novel boundary-spanning connections to detect transformative papers ranked on their divergence modularity (31). These transformative papers can potentially change to the existing structure of knowledge.

We extracted cluster labels from keywords associated with articles that are responsible for the formation of a cluster selected by the likelihood ratio test (*p* < 0.001). Each cluster was closely inspected, and eventually cluster labels were improved based on the authors' judgment.

The second level of the data filtering process was applied during the generation of networks within each dataset (e.g., most cited reference) in order to detect duplicates, references without authors, or any non-relevant unit of measure that was excluded (e.g., DSM reference; CIM-10) or merged (e.g., author Motl RW and Motl W Robert).

The g-index was used for all calculations. This index permits to give credit to lowly cited or non-cited papers while giving credit for highly cited papers, thus partially alleviating bias from highly cited papers as seen with the h-index (32). CiteSpace general parameters are reported in Supplementary Information 1.

## Results

### Analysis of publication outputs, major journals, and growth trend prediction

We report a flowchart with detail of the 56,442 retrieved documents from the WOS Science citation index expanded and the different steps of our scientometric study: identification and screening of studies, software analyses, and expert review's interpretation (Supplementary Figure 1).

Among the retrieved documents, 1,089 documents were excluded, and 55,353 documents encompassing 1,306,828 references were retained (47,105 articles; 6,671 reviews; 564 editorial material; 1,013 proceeding papers). The data filtering process consisted of the inspection of each 604 highly cited papers, editorial material, and proceeding papers and the inspection of 10% randomly selected titles of the retrieved documents. Only 4% (*n* = 224 articles) were not relevant (Supplementary Figure 1).

The retained 55,353 articles were published between 1905 and May 2022 in 24 different languages (95.1% of articles in English). The annual scientific production is still in 2022 exponential with a mean annual growth rate of 6.8% since 1989 (*n* = 17) and 2022 (*n* = 5,604) (Supplementary Figures 2, 3).

The first article identified was a Franz SI and Hamilton GV article on “the effects of exercise upon the retardation in conditions of depression” published in the American Journal of Insanity (33).

### Analysis of co-citation reference: Clusters of research and most cited papers

#### Clusters of research

We constructed a synthesized network of co-cited references based on articles published during the 1988–2021 time period as suggested by CiteSpace after the removal of empty time intervals to optimize time slicing (Figure 1). In this network, each node represents a highly co-cited article. We further explored the latest research trends with the extraction of co-citation networks for the 2016-(May) 2022 time period, and the monthly time sliced networks for the year of 2021 (Supplementary Figure 4). All three networks presented significant modularity and silhouette scores indicating highly credible clusters (*Q* = 0.8481, *S* = 0.9394; *Q* = 0.7712, *S* = 0.9445; and *Q* = 0.4854, *S* = 0.8376, respectively).

The 1988–2021 network identified 50 different clusters, with a single constellation of 26 clusters that reveals six distinct major trends of research on physical activity, namely cardiovascular disease, somatic disorders, cognitive decline/dementia, mental illness, athletes' performance, related health issues and eating disorders and COVID-19 pandemic.

The earliest research trend identified concerns physical activity and cardiovascular diseases consisting of four distinct clusters during the years 1991 to 1997 as follows, with clusters number (clusters' size decreased from cluster number #0), label, silhouette score, size, pooled mean year of publication, the most representative reference; #14, “exercise electrocardiography” (*S* = 0.987; 65; 1987) (34), #7 “silent ischemia” (*S* = 0.964; 145; 1989) (35), #17 “catecholamine” (*S* = 0.987; 46; 1989) (36), and #8 “coronary artery disease” (*S* = 0.962; 141; 1994) (37). This research trend then vanished until it recently reappeared in the 2016–2021 network with cluster #14 “cardio-metabolic health markers” (*S* = 0.991; 6; 2014) (38), #31 “cardiometabolic risk” (*S* = 0.999; 5; 2014) (39), and continues to evolve, as shown in the 2021 network with cluster #9 “cardiovascular disease” (*S* = 0.998; 4; 2016) (40).

The second major trend of research emerged in 1995 on “somatic disorders/public health,” cluster #5 (*S* = 0.953; 238; 1995) (41) that directly evolved into cluster #2 “diabetes” (*S* = 0.918; 289; 2001) (42) and further develop into a relatively isolated cluster #10 “fibromyalgia/copd” (*S* = 0.981; 97; 2005) (43), compared to a succession of other clusters on somatic disorders #16 “cancer” (*S* = 0.993; 52; 2009) (44), #15 “NAFLD” (*S* = 0.998; 55; 2011) (45), #48 “pre-diabetes” (*S* = 0.994; 4; 2012) (46) and #24 “multiple sclerosis” (*S* = 0.999; 9; 2014) (47).

The third major trend concerned cognitive decline and dementia and started in 1997 with a small cluster #20 “Alzheimer's disease” (*S* = 0.993; 16; 1997) (48), then evolved in a much larger cluster #3 “dementia” (*S* = 0.916; 269; 2014) (49), and the largest cluster of the network, cluster #0 “cognitive decline” (*S* = 0.923; 324; 2006) (50). This cluster continues as the most prominent cluster of the 2016–2021 network #0 “evidence-synthesis/cognitive decline” (*S* = 0.938; 221; 2015) (51) and also extended to a cluster on frailty, #9 “frailty” (*S* = 0.991; 15; 2014) (52).

The fourth major trend on research concerned mental illness. This trend started in 2007 with a small cluster #18 “severe mental illness” (*S* = 0.985; 46; 2007) (53), and rapidly evolved in two major clusters, #1 “depression” (*S* = 0.823; 292; 2009) (42), and #4 “schizophrenia” (*S* = 0.912; 267; 2015) (54). The 2016-2021 network confirmed the importance of this major trend with #2 “evidence-synthesis/depression” (*S* = 0.819; 142; 2016) (55). This trend now mainly focus on evidence-synthesis and became #12 “children/adolescents/evidence-synthesis” (*S* = 0.963; 75; 2016) (56).

The fifth trend concerns physical activity, athlete's performance, related health issues, and eating disorders with a succession of small and isolated clusters: #19 “self-confidence” (*S* = 0.995; 38; 1998) (57), #13 “female athletes/eating disorders” (*S* = 0.967; 74; 2000) (58), #50 “motivation” (*S* = 0.997; 4; 2005) (59), and #11 “concussion/chronic traumatic encephalopathy” (*S*0 = 0.996; 11; 2014) (60). A focus on the 2021 network reveals the latest cluster of the trend, #7 “elite athletes” (*S* = 0.986; 75; 2017) (61).

The sixth and last trend concerned COVID-19 pandemic and starts with cluster #6 “COVID-19' (*S* = 0.968; 26; 2019) (62), that continues to evolve in the 2016–2021 network with #1 “COVID-19” (*S* = 0.987; 172; 2019) (63), #20 “post-COVID-19/long COVID” (*S* = 1; 4; 2019) (64) and became in 2021 the most important cluster with #0 “COVID” (*S* = 0.818; 147; 2019) (63), and #4 “COVID/children” (*S* = 0.837; 59; 2019) (65).

Finally, two recent isolated clusters that we cannot relate to a specific trend have also emerged: cluster #9 “greenness/urbanicity” (*S* = 0.998; 2015) (66), and #40 “behavior change” (*S* = 0.996; 7; 2013) (67).

The link walkthrough over time between clusters based on burstness dynamics for the 1988–2021 network is available as a video on osf.io.

#### Most cited papers

We report the top 10 most co-cited references for the 1988–2021 time period in Table 1. The top three most co-cited articles in our network were the Schuch et al.'s meta-analysis on exercise as a treatment of depression (55), followed by the Erickson et al.'s randomized-controlled trial (RCT) on exercise increasing the size of the anterior hippocampus in older adults (50), and the Ngandu et al.'s RCT on the multidomain intervention of diet, exercise, cognitive training, and vascular risk monitoring vs. control to prevent cognitive decline in at-risk elderly people (54).

**Table 1:** The top 10 most cited journals and reference.

**Top 10 co-cited references**										
**Number of citations in the network**	**Number of citations in the literature** ^a^	**Cited reference**	**Year**	**Source**	**Vol**	**Page**	**Title**	**Doi**	**Type of paper**	**Related cluster in** **Figure 1**
311	981	Schuch et al. (14)	2016	J Psychiatr Res	77	42–51	Exercise as a treatment for depression: A meta-analysis adjusting for publication bias	10.1016/j.jpsychires.2016.02.023	Meta-analysis	1
251	4,418	Erickson et al. (50)	2011	Proc Natl Acad Sci USA	108	3,017	Exercise training increases size of hippocampus and improves memory	10.1073/pnas.1015950108	RCT	0
245	2,163	Ngandu et al. (54)	2015	The Lancet	385	2,255	A 2-year multidomain intervention of diet, exercise, cognitive training, and vascular risk monitoring vs. control to prevent cognitive decline in at-risk elderly people (FINGER): a randomized controlled trial	10.1016/S0140-6736(15)60461-5	RCT	3
227	3,691	Livingston et al. (68)	2017	The Lancet	390	2,673	Dementia prevention, intervention, and care	10.1016/S0140-6736(17)31363-6	Review	3
176	645	Schuch et al. (4)	2018	AJP	175	631	Physical Activity and Incident Depression: A Meta-Analysis of Prospective Cohort Studies	10.1176/appi.ajp.2018.17111194	Meta-analysis	1
171	2,025	Norton et al. (69)	2014	Lancet Neurol	13	788	Potential for primary prevention of Alzheimer's disease: an analysis of population-based data	10.1016/S1474-4422(14)70136-X	Meta-analysis	3
165	1,994	Lautenschlager et al. (70)	2008	The Lancet	300	1,027	Effect of physical activity on cognitive function in older adults at risk for Alzheimer disease: a randomized trial	10.1001/jama.300.9.1027	RCT	0
165	10,654	Brooks et al. (63)	2020	The Lancet	395	912–920	The psychological impact of quarantine and how to reduce it: rapid review of the evidence	10.1016/S0140-6736(20)30460-8	Review	6
156	489	Firth et al. (71)	2015	Psychol Med	45	1,343–1,361	A systematic review and meta-analysis of exercise interventions in schizophrenia patients	10.1017/S0033291714003110	Meta-analysis	4
153	454	Vancampfort et al. (7)	2017	World Psychiatry	16	308–315	Sedentary behavior and physical activity levels in people with schizophrenia, bipolar disorder and major depressive disorder: a global systematic review and meta-analysis	10.1002/wps.20458.	Meta-analysis	4

a *Number of citations in the literature according to the journal where the paper was published*.

Moreover, we produced the analysis of burstness for the top references of the 1988–2021, 2016–2021, and 2021 time periods (Supplementary Tables 2P–R). The analysis of burstness revealed that the top three references with the latest and strongest beginning of citation burst were the Warburton and Bredin paper on health benefits of physical activity (72), the Brooks et al. paper on the psychological impact of quarantine (63), and the Stubbs et al. EPA guidance on physical activity as a treatment for severe mental illness (11).

Another important aspect of scientometric studies is the detection of potentially transformative papers, by conducting a structural variation analysis for the 2016–2021 and the 2021–2021 time period (Supplementary Table 3). For the 2016–2021 time period, the top three identified articles based on the strongest centrality divergence were the Stubbs et al. study on factors influencing physical activity among 204,186 people across 46 low-and middle-income countries (73), Vancampfort et al.'s meta-analysis on sedentary behavior and physical activity levels in people with severe mental illness (7), and Vancampfort et al.'s review on physical activity and metabolic disease among people with severe mental illness (74). For the time period January 2021 to May 2021, the top three studies were the Aguilar et al.'s study on the association between leisure-time exercise and depressive symptoms (75), the Schuch et al.'s study on the ELSA-Brasil cohort concerning the association between leisure-time, transport, depression and anxiety symptoms (76), and the van Sluijs et al.'s review on physical activity behaviors during adolescence (77).

### Analysis of co-occurrence of keywords

**Figure 2:**
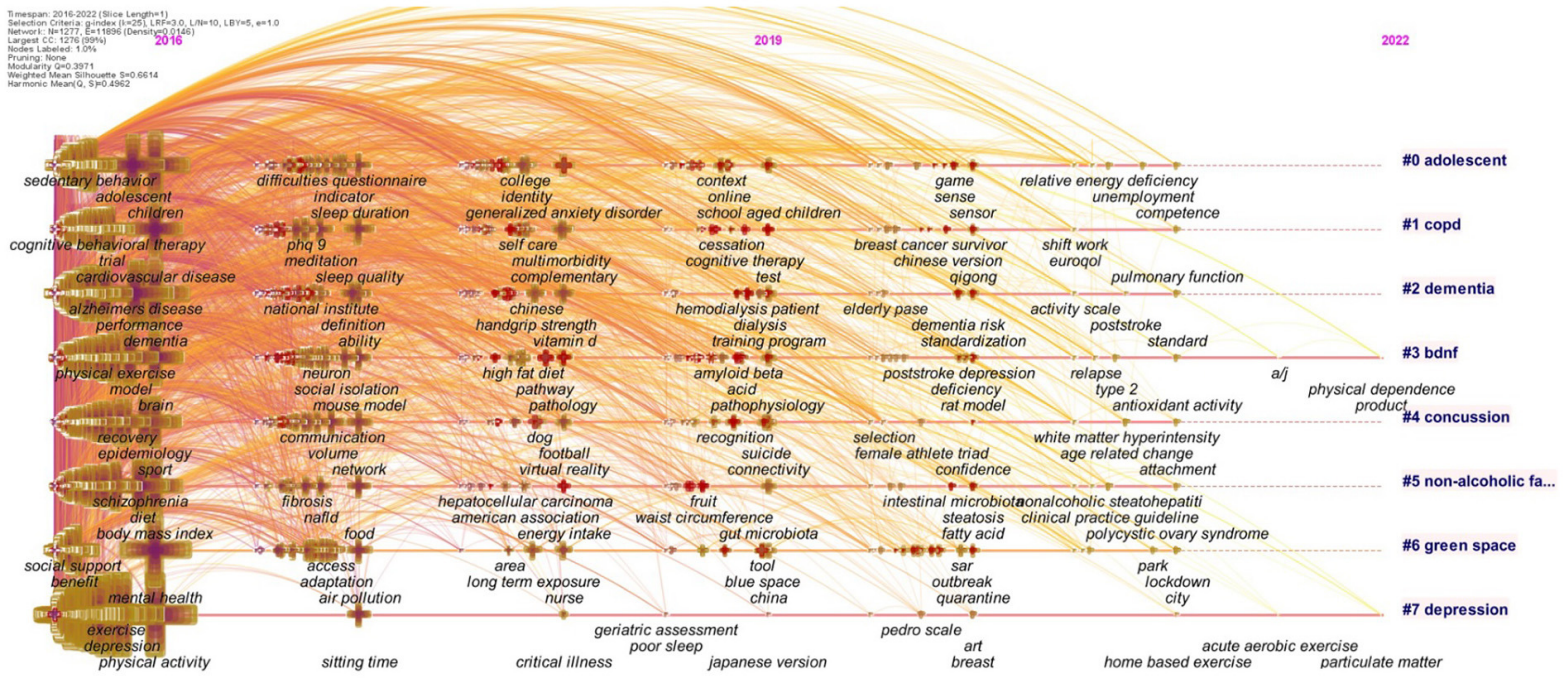
Co-occurrence authors' keyword network (2016–2021). In this co-occurrence author's keywords analysis, the size of the cross is proportional to the frequency of keyword occurrence.

The use of author keywords can help identify the latest trends of research and choose search keywords for future reviews. The co-occurrence author keywords network for 1988–2021 is shown in Supplementary Figure 5, and the 2016–2021 time period is shown in Figure 2. In this network, each node is a highly co-occurring keyword. Both networks presented significant modularity and silhouette scores indicating credible clusters (*Q* = 0.3327, *S* = 0.6823 and *Q* = 0.3971, *S* = 0.6614 respectively).

The 1988–2021 network presented six different clusters: #0 “mental health”; #1 “hippocampus”; #2 “quality of life”; #3 “coronary artery disease”; #4 “obesity,” and #5 “dementia,” and the 2016–2021 network presented seven different clusters: #0 “adolescent”; #1 “copd”; #2 “dementia”; #3 “bdnf”; #4 “concussion”; #5 “non-alcoholic fatty liver disease”; #6 “green space” and #7 “depression”.

The burstness analysis extracted the top 30 co-cited keywords; the latest and strongest beginning of citation bursts for the 1988–2021 network were “quality of life,” “major depression,” “controlled trial,” “meta-analysis,” and “sedentary behavior,” and for the 2016–2021 network were “psychological impact,” “acute respiratory syndrome,” “rat model,” “epidemic,” and “deficiency” (Supplementary Tables 3S–V).

### Analysis of influence and co-operation network

#### Co-cited countries and co-cited institutions network

**Figure 3:**
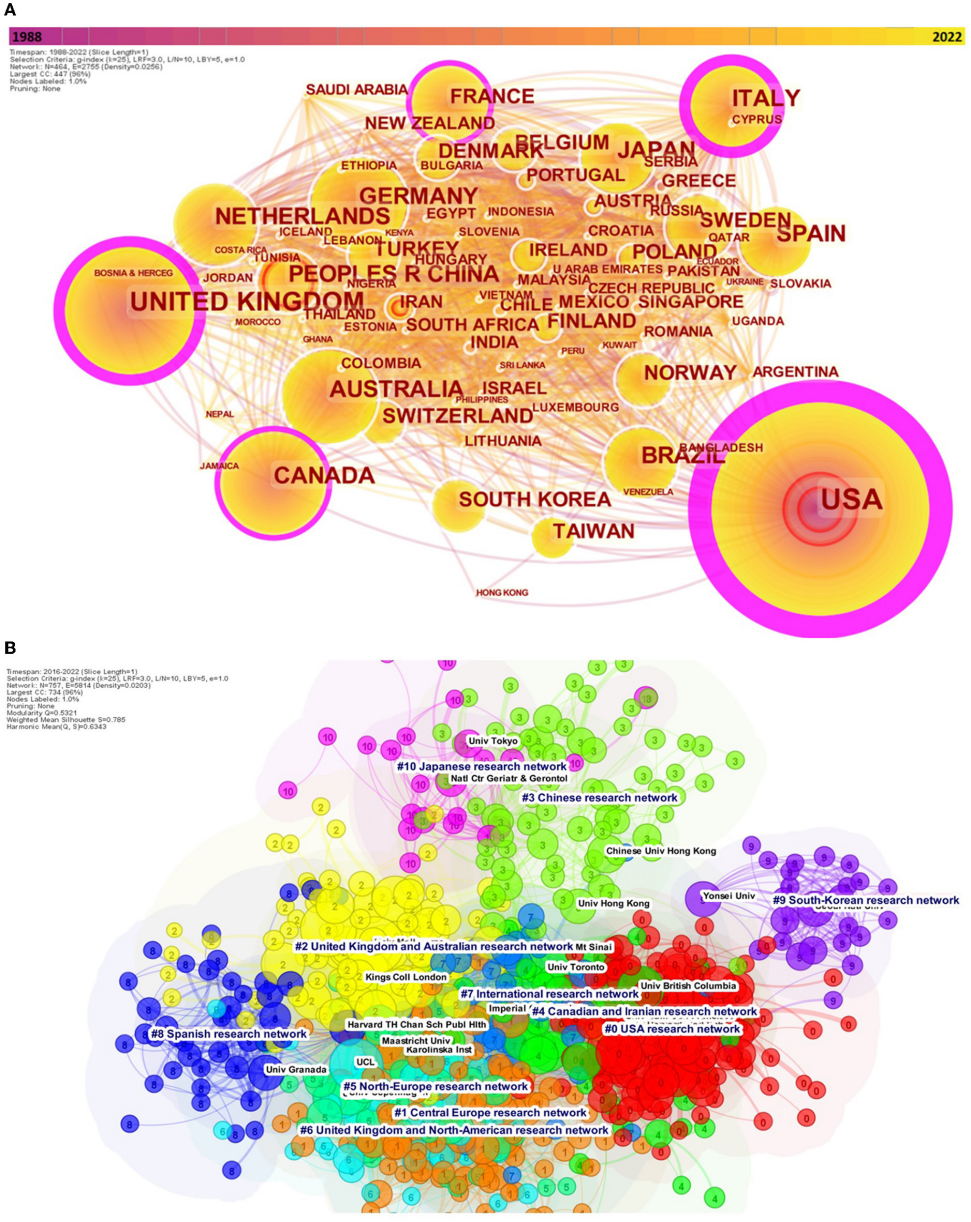
Co-cited author's countries **(A)** (1988–2021) and co-cited author's institutions network with corresponding clusters **(B)** (2016–2021). Both the co-cited author's countries and co-cited author's institutions permits to reveal the collaborative country network. Betweenness centrality organize the network, with the countries presenting the most important centrality being at the center of the network. Nodes are according to each network, countries or institutions. The outermost purple ring denotes the centrality level, and highly central nodes are considered pivotal points in the research field. We limited the nodes to the 80 first countries.

We produced the co-cited countries and co-cited institutions network (Figures 3A,B). Units of measures were authors' countries and authors' institutions. A significant modularity and silhouette score were found (*Q* = 0.5321; *S* = 0.785).

Overall, 176 different countries were identified. In the 1988–2021 network, the country with the most important number of author's citation were the United States of America (USA) (*n* = 17,988), followed by the United Kingdom (*n* = 5,720) Australia (*n* = 4,431), Canada (*n* = 3,773), and People's Republic of China (*n* = 3,160). Similarly, in the 2016–2021 network, the most cited top countries were identical; however, China was now in fourth place (Supplementary Figure 6; Supplementary Table 4). The analysis of burstness reveals confirmed that China was from far the country with the most important strength of burst these last 2 years (231.72), whereas the USA latest important burst date to the 1998–2003 period (83.54) (Supplementary Tables 2A,B). The co-cited author's institutions network reveals what institutions are the most cited. We produced the last five-year network (2016–2021) and identified 757 different organizations (Figure 3B, Supplementary Figure 7).

The most central network was the USA research network #0, with the greatest betweenness centrality to other clusters, such as the Central Europe research network #1, or the United Kingdom and Australian research network #2. The Chinese research network #3, although important in size, was relatively isolated, sharing few links with the Japanese research network #10, whereas the Spanish #8 and the South Korean #9 network were more isolated (Supplementary Table 4). The burstness analysis revealed that the five institutions with the latest and strongest strength of citation burst were as follows: Central South University (China), University of Extremadura (Spain), Federal University of Santa Maria (Brazil), University of Paris (France), and University of Lisbon (Portugal) (Supplementary Tables 2C,D). The sigma score revealed that the institutions with the greatest scores were Charité (#1; 2016), Medical University of Vienna (#1; 2017), and Peking University (#3; 2016).

#### Co-authorship, co-cited and co-cited journals network

Our dataset includes 1,306,827 citations with an average of 31.85 citations per document. About 175,508 different authors were found, with an average of 3.17 authors and 5.76 co-authors per document in 4,193 different sources (e.g., books and journals) (Supplementary Figure 1).

We produced the co-authorship networks, which are the social networks encompassing researchers that reflect collaboration among them, each node representing a different highly cited co-author (Supplementary Figure 8, Supplementary Table 4). The network revealed that French researchers are closely collaborating within France and on physical exercise and aging/depression (#9; 2018). The burstness analysis revealed that the co-authors that were the most participating in articles these last years were Stubbs B, Smith L, De Hert M, Vancampfort D and Probst M (Supplementary Tables 2G,H). We further produce the co-cited author network that permits to visualize “who cites who” for the last 5 years (2016–2021 network) was also conducted (Supplementary Figure 9). The burstness analysis revealed that the most co-cited first authors according to our datasets were Brooks SK, Wang CY, Ogden CL, Holmes EA, and Kandola SA. Furthermore, the latest top cited authors (as first authors) with the most important strength of burst were Brooks SK, Schuch FB, Wang CY, Firth J, and Stubbs B (Supplementary Tables 3I,J).

The top five journals with the most documents were as follows: the *International Journal of Environmental Research and Public Health* (*n* = 1,164) in first place with a massive raise of documents these last 3 years; *PLOS ONE* (*n* = 1,017); *BMC Public Health* (*n* = 625); *BMJ OPEN* (*n* = 513) and the *Journal of Affective Disorders* (*n* = 453) (Supplementary Figure 10). We conducted the co-cited journal network that retained 2,879 journals and showed the highly cited journals with high betweenness centrality (Supplementary Figure 11).

The top five highly cited journals were *Archives of General Psychiatry* (JAMA), *The Lancet, PLOS ONE, Medicine and Science in Sports and Exercise*, and the *New England Journal of Medicine* (Table 2). The burstness analysis further reveals that five journals with the latest beginning of burst were *Frontiers in Psychology, The Lancet Psychiatry, International Journal of Environmental Research and Public Health, Nutrients*, and *Frontiers in Psychiatry* (Supplementary Tables 2E,F).

**Table 2:** Journals with most articles and citations.

**Top journals**						
**Journals with most articles (1980–2021)**	**Initial year**	**Impact factor (2020–2021)**	**Total articles of the dataset (%)**	**Total articles**	**Journals with most citations (1980–2021)**	**Total citations in our dataset**
1. International Journal of Environmental Research and Public Health	2004	3.39	2.1	1,164	1. Archives of General Psychiatry (JAMA)	20,557
2. PLoS ONE	2006	3.24	1.8	1,017	2. The Lancet	13,884
3. BMC Public Health	2000	3.17	1.1	625	3. PLoS ONE	12,418
4. BMJ OPEN	2011	2.69	0.92	513	4. Medicine and Science in Sports and Exercise	11,568
5. Journal of Affective Disorders	1979	4.83	0.81	453	5. Journal of the American Geriatrics Society	11,203
6. Journal of the American Geriatrics Society	1953	5.56	0.81	448	6. BMJ	8,576
7. Psychology of Sport and Exercise Physiology	1996	4.78	0.57	317	7. Circulation	7,686
8. American Journal of Cardiology	1958	2.77	0.56	313	8. American Journal of Psychiatry	7,823
9. BMC Geriatrics	2001	3.73	0.55	309	9. Archives of Internal Medicine	7,492
10. Frontiers in Psychiatry	2010	3.53	0.51	287	10. American Journal of Epidemiology	6,919

## Discussion

### Summary of the main findings

To the best of our knowledge, this is the first broad scientometric that proposes a comprehensive overview of the development of research on physical activity, mental health, and wellbeing.

We retained 55,353 documents revealing an exponential growth of scientific production since the 90s. The USA holds for decades the leading position in research; however, China is very active since 2020 with an important burst of citations, mainly due to publication on COVID-19. The King's College London and Harvard University were the most influential institutions in terms of citation count. In supplement to actual reviews, this scientometric study reveals the influence and collaboration network, which could help researchers to identify major scholarly communities and establish potential research collaboration.

### Identification of research trends

The six distinct major trends of research identified expose the history and the latest development of research on physical activity, mental health, and wellbeing. The first major trend of research concerns physical activity and cardiovascular disease, reminding the past and present intertwine. First research focused on cardiovascular disease (35). The large body of research on evidence synthesis of the last decades that mainly focused on the prevention to treatment role of physical activity for cardiovascular disease started with guidelines for exercise testing (37, 78), and that continues to date with consideration of cardiometabolic risk factors (39).

The extension of prevention and treatment of physical activity to other somatic disorders constituted the second major trend, making levels of physical activity a public health priority (41), that continues to date (79). Another trend, which emerged after 2000, is the potential of physical activity for the prevention and treatment of dementia with increased importance of evidence-synthesis studies (51, 80, 81).

Physical activity has also been explored as a potential intervention for the prevention and treatment of dementia. As regards to prevention, it has been demonstrated that physical activity is a protective factor against Alzheimer's disease and other types of dementia (82, 83). As a treatment, recently an umbrella review has pooled evidence from as many as 27 systematic reviews, including 18 with meta-analyses, overall reporting on 28,205 participants with mild cognitive impairment or dementia (84). The authors showed that mind-body intervention and mixed physical activity interventions had a small effect on global cognition, whereas resistance training had a large effect on global cognition in those with mild cognitive impairment. In people affected by dementia, a small effect of physical activity/exercise emerged in improving global cognition in Alzheimer's disease and all types of dementia. Importantly, physical activity/exercise also improved other outcomes not strictly related to cognition, including the risk of falls, and neuropsychiatric symptoms.

Adjacently, a massive body of evidence has organized an important trend of research on the benefits of physical activity for both prevention and treatment of severe mental disorders, in particular depression (4, 85, 86) and schizophrenia (71, 87). More recently, the evidence has focused on evidence-synthesis (10, 74) and mental health/wellbeing (9).

Other lesser, although highly relevant trends were also uncovered, such as the importance of physical activity for athlete's performance (88, 89). While most of the research efforts in that area have focused on how to optimize performance in the context of professional athletics (90), perfectionism, and excessive physical activity can also be a symptom of mental disorders, and eating disorders in particular (58). This research trends now focus on concussion and its consequence (chronic traumatic encephalopathy) (60).

Finally, a large body of research has focused on physical activity and COVID-19. Physical activity is a protective factor for COVID-19 complications (91). During COVID-19 research has also focused on restrictions and physical activity (63). Finally, physical activity's relevance has also been shown to extend beyond the clinical sciences and start to dialogue with greenness and urban planning (66, 92, 93).

Although various trends of research have developed these last decades, we can identify two important gaps, the one of the roles of physical activity in the prevention or treatment of substance-use disorders, and the one regarding the socioeconomic inequalities in access to physical exercise (94). Meta-review covering this subject (10) concluded that exercise can improve multiple mental health outcomes in those with alcohol-use disorders and substance-use disorders; however, further research is needed in these conditions, notably with the use of mind-body practices (95, 96).

### Strengths and limitations

This work has strengths and weaknesses. Strengths are its novel evidence-synthesis approach, complete systematic reviews, and meta-analysis, by providing information on the evolution of research trends over time, the visualization of networks of authors, countries, and institutions, and that go beyond common measures of academic bibliometric performance (i.e., impact factor, H-Index, number of papers or citations). This novel research framework permits repeatable, reproducible, and comparable analysis with less bias than conventional time-consuming reviews that are vulnerable to biased coverage/selection.

Limitations are that, despite the quality check procedures outlined in the methods, this is not a systematic review. Furthermore, gathered data were only obtained from WOSCC, which can limit retrieved publication (94, 97). Also, the centrality and number of citations are not necessarily indicative of the quality of a work, as faulty publications can be highly cited because they are frequently criticized as well (98). Finally, no reporting guidance is available for scientometric studies yet, given their recent introduction in the literature.

## Conclusion

In conclusion, researchers have consistently focused on the role of physical activity on cardiovascular disease, other somatic disorders, dementia, mental disorders, athlete's performance, and eating disorders and more recently on COVID-19 pandemic, which clearly shows the role of physical activity as medicine across physical and mental disorders. More recently, the literature has focused on green space, urban planning, and behavior change, further expanding the multidisciplinary reach of physical activity. Taken together our results strengthen and expand the specific and central role of physical activity in public health, calling for the systematic involvement of physical activity professionals as stakeholders in the public health decision-making process.

## Data availability statement

The raw data supporting the conclusions of this article will be made available by the authors, without undue reservation.

## Author contributions

MSa and MSo: conceptualization and writing—original draft preparation. MSa, CC, and MSo: methodology, formal analysis, and investigation. MSa, CC, OS, JD, DV, JF, LS, BS, SR, FS, and MSo: writing—review and editing. CC and MSo: supervision. All authors contributed to the article and approved the submitted version.

## Funding

Open access funding was provided by the University of Geneva.

## Conflict of interest

Author OS has received advisory board honoraria from Otsuka, Lilly, Lundbeck, Sandoz, and Janssen in an institutional account for research and teaching. Author JF has received consultancy fees from Parachute BH for a separate project. Author BS is on the Editorial Board of Ageing Research Reviews, Mental Health and Physical Activity, the Journal of Evidence Based Medicine and the Brazilian Journal of Psychiatry. Author BS has received honorarium from a co-edited a book on exercise and mental illness, advisory work from ASICS & ParachuteBH for unrelated work. Author MSo has received honoraria/has been a consultant for Angelini, Lundbeck and Otsuka. The remaining authors declare that the research was conducted in the absence of any commercial or financial relationships that could be construed as a potential conflict of interest.

## Publisher's note

All claims expressed in this article are solely those of the authors and do not necessarily represent those of their affiliated organizations, or those of the publisher, the editors and the reviewers. Any product that may be evaluated in this article, or claim that may be made by its manufacturer, is not guaranteed or endorsed by the publisher.
