# Erratum: Thirty years of research on physical activity, mental health, and wellbeing: A scientometric analysis of hotspots and trends

**DOI:** 10.3389/fpubh.2023.1178895

**Published:** 2023-03-14

**Authors:** Frontiers Production Office

**Affiliations:** Frontiers Media SA, Lausanne, Switzerland

**Keywords:** physical exercise, mental illness, evidence synthesis, scientometrics, CiteSpace

An omission to the funding section of the original article was made in error. The following sentence has been added: “Open access funding was provided by the University of Geneva.”

The original article has been updated.

