# Supplementary material for: Thirty years of research on physical activity, mental health, and wellbeing: A scientometric analysis of hotspots and trends
Source: Front Public Health. 2022 Aug 9;10:943435. doi: 10.3389/fpubh.2022.943435 (PMC9396383; doi:10.3389/fpubh.2022.943435)
Supplement: Supplementary file 1 [file Data_Sheet_1.PDF]

Supplementary Material

Contents

Page 2. Supplementary Information 1. CiteSpace general parameters
Page 3. Supplementary Table 1. False-positive among the 604 highly cited articles of our dataset.
Page 4-17. Supplementary Table 2. Burstness analysis for countries, institutions, authors, references and keywords (1988-2021, 2016-2021 and 2021)
Page 18. Supplementary Table 3. Top 10 articles with the strongest centrality divergence scores for the 2016-2021 and the 2021 time periods.
Page 19. Supplementary Table 4. The top countries, institutions and top cited authors (1988-2021 and 2016-2021 period)
Top journals
Page 20. Supplementary Table 5. Journals with most articles and citations
Page 21. **Supplementary Figure 1.** Flow chart of the scientometric analysis
Page 22. Supplementary Figure 2. Annual scientific production for the 1981-2021 time period
Page 23. Supplementary Figure 3. Average article citations per year for the 1967-2021 time period
Page 24-78. Supplementary Figure 4. Detail of the reference co-citation network clusters (time map) and clusters' details for the 1988-2021 (A), reference co-citation network clusters (burstness, clusters, time map), and clusters' details for the 2016-2021 time period (B) and reference co-citation network clusters (burstness, clusters, time map), and clusters' details for 2021 (C) time period
Page 79. Supplementary Figure 5. Co-occurrence authors' keyword network (1988-2021)
Page 80. Supplementary Figure 6. Co-cited author's countries network (2016-2021)
Page 81-93. Supplementary Figure 7. Co-cited institution network details (2016-2021)
Page 94-104. Supplementary Figure 8. Co-authorship network detail (1988-2021)
Page 105-121. Supplementary Figure 9. Author co-citation network detail (2016-2021)
Page 122. Supplementary Figure 10. Source growths for the top 10 Journals based on the number of articles in our dataset (1988-2021 time period)
Page 123-124. Supplementary Figure 11. Journal co-citation network analysis (A), with corresponding clusters (B) (2016-2021)

Supplementary Information 1. CiteSpace general parameters

General CiteSpace parameters used for analysis:

CiteSpace parameters were as follows:

- Link retaining factor of 3.0
- Look back years (-1)
- Time span (1980–2021) with one slice per year
- Links (strength: cosine, scope: within slices)
- Selection criteria (g-index scale factor of 25)
- Minimum duration set to 5 years for the 1988-2021 networks, and to 2 years for the 2016-2021 networks

Supplementary Table 1. False-positive among the 604 highly cited articles of our dataset.

DOI	Title	Reason of possible false positive
10.1016/j.clinph.2016.10.087	Evidence-based guidelines on the therapeutic use of transcranial direct current stimulation (tDCS)	Highly cited reference by retained reviews
10.1093/brain/awr039	Harnessing neuroplasticity for clinical applications	Non relevant keywords plus: ‘‘induced movement therapy’’/ Highly cited reference by retained reviews
10.1016/j.healthplace.2012.04.001	Crime, fear of crime, environment, and mental health and wellbeing: Mapping review of theories and causal pathways	Non relevant keywords plus: ‘‘physical-activity’’
10.3233/JAD-179939	Religious Orders Study and Rush Memory and Aging Project	Non relevant f keywords plus: ‘‘physical-activity’’
10.1016/j.jacc.2019.04.034	Vascular Cognitive Impairment and Dementia	Non relevant keywords plus: ‘‘physical-activity’’/ Highly cited reference by retained reviews
10.1016/j.neuroimage.2010.10.070	Quantitative imaging of intrinsic magnetic tissue properties using MRI signal phase: An approach to in vivo brain iron metabolism?	Highly cited reference by neuroimaging studies
10.1001/jamainternmed.2014.8063	Duration of Menopausal Vasomotor Symptoms Over the Menopause Transition	Non relevant keywords plus: ‘‘physical-activity’’/ Highly cited reference by retained reviews
10.1093/scan/nsaa046	Neurostructural correlates of hope: dispositional hope mediates the impact of the SMA gray matter volume on subjective well-being in late adolescence	Non relevant keywords plus: ‘‘physical-activity’’/ Highly cited reference by retained reviews
10.3390/ani6030021	Updating Animal Welfare Thinking: Moving beyond the "Five Freedoms" towards "A Life Worth Living"	Highly cited reference by retained reviews
10.1016/j.biopsych.2015.04.016	Dynamic Network Communication as a Unifying Neural Basis for Cognition, Development, Aging, and Disease	Highly cited reference by retained reviews
10.1542/peds.2009-3592	Health Effects of Energy Drinks on Children, Adolescents, and Young Adults	Non relevant keywords plus: ‘‘exercise; performance’’/ Highly cited reference by retained reviews

Supplementary Table 2. Burstness analysis for countries, institutions, authors, references and keywords (1988-2021, 2016-2021 and 2021)

A. Top 20 countries with the strongest beginning year of citation burst (1988-2021)

Countries	Year	Strength	Begin	End	1988 - 2021
ISRAEL	1988	17.08	1988	2001	
ITALY	1988	9.56	1990	1992	
RUSSIA	1988	8.99	1990	2005	
UNITED KINGDOM	1988	61.88	1991	2007	
HONG KONG	1988	11.83	1992	1998	
VENEZUELA	1988	4.35	1995	2004	
FINLAND	1988	18.61	1996	2006	
USA	1988	83.54	1998	2003	
JAPAN	1988	22.6	1998	2001	
GREECE	1988	4.63	2004	2010	
SCOTLAND	1988	4.89	2013	2014	
UGANDA	1988	9.04	2016	2018	
ETHIOPIA	1988	9.27	2019	2021	
CYPRUS	1988	8.63	2019	2021	
PEOPLES R CHINA	1988	231.72	2020	2021	
IRAN	1988	18.21	2020	2021	
SAUDI ARABIA	1988	16.69	2020	2021	
BANGLADESH	1988	12.59	2020	2021	
U ARAB EMIRATES	1988	8.14	2020	2021	
VIETNAM	1988	7.41	2020	2021	

B. Top 20 countries with the strongest strength of citation bursts (1988-2021)

Countries	Year	Strength	Begin	End	1988 - 2021
PEOPLES R CHINA	1988	231.72	2020	2021	
USA	1988	83.54	1998	2003	
UNITED KINGDOM	1988	61.88	1991	2007	
JAPAN	1988	22.6	1998	2001	
FINLAND	1988	18.61	1996	2006	
IRAN	1988	18.21	2020	2021	
ISRAEL	1988	17.08	1988	2001	
SAUDI ARABIA	1988	16.69	2020	2021	
BANGLADESH	1988	12.59	2020	2021	
HONG KONG	1988	11.83	1992	1998	
ITALY	1988	9.56	1990	1992	
ETHIOPIA	1988	9.27	2019	2021	
UGANDA	1988	9.04	2016	2018	
RUSSIA	1988	8.99	1990	2005	
CYPRUS	1988	8.63	2019	2021	
U ARAB EMIRATES	1988	8.14	2020	2021	
VIETNAM	1988	7.41	2020	2021	
INDONESIA	1988	6.07	2020	2021	
ROMANIA	1988	5.26	2020	2021	
PAKISTAN	1988	5.24	2020	2021	

C. Top 25 institutions with the strongest beginning year of citation burst (2016-2021)

Institutions	Year	Strength	Begin	End	2016 - 2021
Hosp Clin Porto Alegre	2016	10.7	2016	2018	
SUNY Buffalo	2016	8.09	2016	2018	
Bangor Univ	2016	8.01	2016	2017	
Acad Med Ctr	2016	7.8	2016	2018	
Vrije Univ Amsterdam Med Ctr	2016	7.23	2016	2018	
Univ Tampere	2016	7.12	2016	2017	
Univ Duisburg Essen	2016	6.65	2016	2018	
Univ North Carolina Chapel Hill	2016	9.06	2017	2018	
Ctr Dis Control & Prevent	2016	7.95	2017	2019	
Butabika Natl Referral & Mental Hlth Hosp	2016	7.71	2017	2018	
Wake Forest Univ	2016	6.9	2017	2018	
Univ York	2016	7.52	2018	2019	
Haukeland Hosp	2016	7.41	2018	2019	
Natl Ctr Neurol & Psychiat	2016	6.7	2018	2019	
Swinburne Univ Technol	2016	9.38	2019	2020	
Sorbonne Univ	2016	7.81	2019	2020	
Jagiellonian Univ	2016	6.56	2019	2020	
Cent South Univ	2016	11.36	2020	2021	
Univ Extremadura	2016	9.65	2020	2021	
Univ Fed Santa Maria	2016	8.8	2020	2021	
Univ Paris	2016	8.52	2020	2021	
Univ Lisbon	2016	7.8	2020	2021	
Med Univ Silesia	2016	7.66	2020	2021	
Sun Yat Sen Univ	2016	6.92	2020	2021	
Univ Pavia	2016	6.81	2020	2021	

D. Top 25 institutions with the strongest strength of citation burst (2016-2021)

Institutions	Year	Strength	Begin	End	2016 - 2021
Cent South Univ	2016	11.36	2020	2021	
Hosp Clin Porto Alegre	2016	10.7	2016	2018	
Univ Extremadura	2016	9.65	2020	2021	
Swinburne Univ Technol	2016	9.38	2019	2020	
Univ North Carolina Chapel Hill	2016	9.06	2017	2018	
Univ Fed Santa Maria	2016	8.8	2020	2021	
Univ Paris	2016	8.52	2020	2021	
SUNY Buffalo	2016	8.09	2016	2018	
Bangor Univ	2016	8.01	2016	2017	
Ctr Dis Control & Prevent	2016	7.95	2017	2019	
Sorbonne Univ	2016	7.81	2019	2020	
Acad Med Ctr	2016	7.8	2016	2018	
Univ Lisbon	2016	7.8	2020	2021	
Butabika Natl Referral & Mental Hlth Hosp	2016	7.71	2017	2018	
Med Univ Silesia	2016	7.66	2020	2021	
Univ York	2016	7.52	2018	2019	
Haukeland Hosp	2016	7.41	2018	2019	
Vrije Univ Amsterdam Med Ctr	2016	7.23	2016	2018	
Univ Tampere	2016	7.12	2016	2017	
Sun Yat Sen Univ	2016	6.92	2020	2021	
Wake Forest Univ	2016	6.9	2017	2018	
Univ Pavia	2016	6.81	2020	2021	
Natl Ctr Neurol & Psychiat	2016	6.7	2018	2019	
Univ Duisburg Essen	2016	6.65	2016	2018	
Jagiellonian Univ	2016	6.56	2019	2020	

E. Top 25 journals with the strongest beginning year of citation burst (1988-2021)

Cited Journals	Year	Strength	Begin	End	1988 - 2021
AM J CARDIOL	1988	1001.51	1988	2007	
NEW ENGL J MED	1988	900.81	1988	2008	
CIRCULATION	1988	750.62	1988	2005	
AM HEART J	1988	669.41	1988	2007	
J AM COLL CARDIOL	1988	651.83	1988	2005	
BRIT MED J	1988	558.56	1988	2009	
ANN INTERN MED	1988	465.46	1988	2009	
BRIT HEART J	1988	434.13	1988	2005	
AM J MED	1988	374.23	1988	2009	
EUR HEART J	1988	332.81	1989	2005	
ARCH GEN PSYCHIAT	1988	494.65	1990	2007	
AM J PHYSIOL	1988	308.83	1990	2007	
AM J PSYCHIAT	1988	306.91	1990	2007	
J APPL PHYSIOL	1988	242.79	1990	2007	
J GERONTOL	1988	332.43	1991	2009	
ARCH INTERN MED	1988	243.12	1993	2012	
DIAGN STAT MAN MENT	1988	259.73	1996	2013	
PLOS ONE	1988	257.88	2018	2021	
SCI REP-UK	1988	443.15	2019	2021	
BMJ OPEN	1988	375.73	2019	2021	
FRONT PSYCHOL	1988	372.84	2019	2021	
LANCET PSYCHIAT	1988	256.36	2019	2021	
INT J ENV RES PUB HE	1988	701.24	2020	2021	
NUTRIENTS	1988	304.21	2020	2021	
FRONT PSYCHIATRY	1988	227.95	2020	2021	

F. Top 25 journals with the strongest strength of citation burst (1988-2021)

Cited Journals	Year	Strength	Begin	End	1988 - 2021
AM J CARDIOL	1988	1001.51	1988	2007	
NEW ENGL J MED	1988	900.81	1988	2008	
CIRCULATION	1988	750.62	1988	2005	
INT J ENV RES PUB HE	1988	701.24	2020	2021	
AM HEART J	1988	669.41	1988	2007	
J AM COLL CARDIOL	1988	651.83	1988	2005	
BRIT MED J	1988	558.56	1988	2009	
ARCH GEN PSYCHIAT	1988	494.65	1990	2007	
ANN INTERN MED	1988	465.46	1988	2009	
SCI REP-UK	1988	443.15	2019	2021	
BRIT HEART J	1988	434.13	1988	2005	
BMJ OPEN	1988	375.73	2019	2021	
AM J MED	1988	374.23	1988	2009	
FRONT PSYCHOL	1988	372.84	2019	2021	
EUR HEART J	1988	332.81	1989	2005	
J GERONTOL	1988	332.43	1991	2009	
AM J PHYSIOL	1988	308.83	1990	2007	
AM J PSYCHIAT	1988	306.91	1990	2007	
NUTRIENTS	1988	304.21	2020	2021	
DIAGN STAT MAN MENT	1988	259.73	1996	2013	
PLOS ONE	1988	257.88	2018	2021	
LANCET PSYCHIAT	1988	256.36	2019	2021	
ARCH INTERN MED	1988	243.12	1993	2012	
J APPL PHYSIOL	1988	242.79	1990	2007	
FRONT PSYCHIATRY	1988	227.95	2020	2021	

G. Top 25 co-authors with strongest beginning of citation busts (1988-2021)

Authors	Year	Strength	Begin	End	1988 - 2021
PM OKIN	1988	12.9	1991	1996	
P KLIGFIELD	1988	11.06	1991	1996	
VF FROELICHER	1988	10.73	1991	2002	
JM GURALNIK	1988	16.43	1999	2006	
DAVID A BENNETT	1988	12.79	2006	2012	
ARON S BUCHMAN	1988	10.33	2007	2011	
MICHAEL BERK	1988	13.24	2008	2014	
MIKA KIVIMAKI	1988	10.87	2008	2012	
KERRY S COURNEYA	1988	10.54	2008	2012	
EDWARD MCAULEY	1988	11.29	2009	2016	
MARC DE HERT	1988	25.74	2011	2016	
MICHEL PROBST	1988	23.37	2011	2016	
FELICE N JACKA	1988	11.62	2011	2016	
ANDREW SOUNDY	1988	11.5	2014	2016	
BRENDON STUBBS	1988	36.11	2015	2020	
DAVY VANCAMPFORT	1988	23.94	2015	2018	
SIMON ROSENBAUM	1988	20.63	2015	2019	
PHILIP B WARD	1988	13.32	2015	2019	
TERESA LIUAMBROSE	1988	11.68	2015	2018	
FELIPE B SCHUCH	1988	12.3	2016	2021	
JOSEPH FIRTH	1988	22.84	2017	2020	
AI KOYANAGI	1988	22.38	2017	2021	
MATS HALLGREN	1988	18.01	2017	2020	
NICOLA VERONESE	1988	12.46	2017	2020	
LEE SMITH	1988	31.47	2019	2021	

H. Top 25 co-authors with the strongest strength of citation bursts (1988-2021)

Authors	Year	Strength	Begin	End	1988 - 2021
BRENDON STUBBS	1988	36.11	2015	2020	
LEE SMITH	1988	31.47	2019	2021	
MARC DE HERT	1988	25.74	2011	2016	
DAVY VANCAMPFORT	1988	23.94	2015	2018	
MICHEL PROBST	1988	23.37	2011	2016	
JOSEPH FIRTH	1988	22.84	2017	2020	
AI KOYANAGI	1988	22.38	2017	2021	
SIMON ROSENBAUM	1988	20.63	2015	2019	
MATS HALLGREN	1988	18.01	2017	2020	
JM GURALNIK	1988	16.43	1999	2006	
PHILIP B WARD	1988	13.32	2015	2019	
MICHAEL BERK	1988	13.24	2008	2014	
PM OKIN	1988	12.9	1991	1996	
DAVID A BENNETT	1988	12.79	2006	2012	
NICOLA VERONESE	1988	12.46	2017	2020	
FELIPE B SCHUCH	1988	12.3	2016	2021	
TERESA LIUAMBROSE	1988	11.68	2015	2018	
FELICE N JACKA	1988	11.62	2011	2016	
ANDREW SOUNDY	1988	11.5	2014	2016	
EDWARD MCAULEY	1988	11.29	2009	2016	
P KLIGFIELD	1988	11.06	1991	1996	
MIKA KIVIMAKI	1988	10.87	2008	2012	
VF FROELICHER	1988	10.73	1991	2002	
KERRY S COURNEYA	1988	10.54	2008	2012	
ARON S BUCHMAN	1988	10.33	2007	2011	

I. Top 25 cited authors with strongest beginning of citation bursts (1988-2021)

Cited Authors	Year	Strength	Begin	End	1988 - 2021
PAFFENBARGER RS	1988	106.56	1988	2012	
SPIELBERGER CD	1988	93.01	1988	2008	
BRUCE RA	1988	87.9	1988	2005	
ELLESTAD MH	1988	75.78	1988	2001	
WEINER DA	1988	137.22	1989	2002	
DETRANO R	1988	84.03	1989	2001	
OKIN PM	1988	75.31	1989	2004	
DEANFIELD JE	1988	96.18	1991	2001	
KANNEL WB	1988	77.84	1991	2004	
FLETCHER GF	1988	73.02	1992	2008	
GARNER DM	1988	72.33	1992	2007	
BLAIR SN	1988	81.52	1993	2012	
STEWART AL	1988	98.69	1994	2010	
KING AC	1988	77.73	1995	2009	
MARTENS R	1988	84.96	1996	2012	
MARTINSEN EW	1988	84.04	1996	2013	
GIBBONS RJ	1988	83.52	1998	2013	
PENNINX BWJH	1988	75.88	2000	2011	
WARE JE	1988	175.31	2003	2011	
DUNN AL	1988	88.76	2003	2013	
STUBBS B	1988	85.01	2017	2020	
SCHUCH FB	1988	108.67	2018	2021	
FIRTH J	1988	85.68	2018	2021	
LIVINGSTON G	1988	79.86	2019	2021	
BROOKS SK	1988	117.34	2020	2021	

J. Top 25 cited authors with the strongest strength of citation bursts (1988-2021)

Cited Authors	Year	Strength	Begin	End	1988 - 2021
WARE JE	1988	175.31	2003	2011	
WEINER DA	1988	137.22	1989	2002	
BROOKS SK	1988	117.34	2020	2021	
SCHUCH FB	1988	108.67	2018	2021	
PAFFENBARGER RS	1988	106.56	1988	2012	
STEWART AL	1988	98.69	1994	2010	
DEANFIELD JE	1988	96.18	1991	2001	
SPIELBERGER CD	1988	93.01	1988	2008	
DUNN AL	1988	88.76	2003	2013	
WANG CY	1988	88.06	2020	2021	
BRUCE RA	1988	87.9	1988	2005	
FIRTH J	1988	85.68	2018	2021	
STUBBS B	1988	85.01	2017	2020	
MARTENS R	1988	84.96	1996	2012	
MARTINSEN EW	1988	84.04	1996	2013	
DETRANO R	1988	84.03	1989	2001	
GIBBONS RJ	1988	83.52	1998	2013	
BLAIR SN	1988	81.52	1993	2012	
LIVINGSTON G	1988	79.86	2019	2021	
KANNEL WB	1988	77.84	1991	2004	
KING AC	1988	77.73	1995	2009	
PENNINX BWJH	1988	75.88	2000	2011	
ELLESTAD MH	1988	75.78	1988	2001	
OKIN PM	1988	75.31	1989	2004	
FLETCHER GF	1988	73.02	1992	2008	

K. Top 25 cited authors with strongest beginning of citation bursts (2016-2021)

Cited Authors	Year	Strength	Begin	End	2016 - 2021
OGDEN CL	2016	33.12	2016	2018	
SOUNDY A	2016	25.22	2016	2018	
DAUMIT GL	2016	24.53	2016	2018	
MORRIS JN	2016	21.87	2016	2017	
RIMMER JH	2016	20.5	2016	2017	
LORIG K	2016	19.93	2016	2018	
MILLER WR	2016	19.31	2016	2018	
COONEY GM	2016	18.82	2016	2018	
LEUCHT S	2016	18.7	2016	2017	
WING RR	2016	18.7	2016	2018	
PEREIRA AC	2016	18.39	2016	2018	
SNAITH RP	2016	18.77	2017	2019	
MOUSSAVI S	2016	18.24	2017	2018	
BROOKS SK	2016	68.09	2020	2021	
WANG CY	2016	52.93	2020	2021	
HOLMES EA	2016	26.57	2020	2021	
KANDOLA A	2016	26.57	2020	2021	
GUTHOLD R	2016	24.43	2020	2021	
ASHDOWN-FRANKS G	2016	21.1	2020	2021	
RODRIGUEZ-AYLLON M	2016	20.58	2020	2021	
PIERCY KL	2016	20.17	2020	2021	
CAO WJ	2016	19.53	2020	2021	
HUANG YE	2016	18.75	2020	2021	
JAMES SL	2016	18.75	2020	2021	
QIU JY	2016	18.23	2020	2021	

L. Top 25 cited authors with the strongest strength of citation bursts (2016-2021)

Cited Authors	Year	Strength	Begin	End	2016 - 2021
BROOKS SK	2016	68.09	2020	2021	
WANG CY	2016	52.93	2020	2021	
OGDEN CL	2016	33.12	2016	2018	
HOLMES EA	2016	26.57	2020	2021	
KANDOLA A	2016	26.57	2020	2021	
SOUNDY A	2016	25.22	2016	2018	
DAUMIT GL	2016	24.53	2016	2018	
GUTHOLD R	2016	24.43	2020	2021	
MORRIS JN	2016	21.87	2016	2017	
ASHDOWN-FRANKS G	2016	21.1	2020	2021	
RODRIGUEZ-AYLLON M	2016	20.58	2020	2021	
RIMMER JH	2016	20.5	2016	2017	
PIERCY KL	2016	20.17	2020	2021	
LORIG K	2016	19.93	2016	2018	
CAO WJ	2016	19.53	2020	2021	
MILLER WR	2016	19.31	2016	2018	
COONEY GM	2016	18.82	2016	2018	
SNAITH RP	2016	18.77	2017	2019	
HUANG YE	2016	18.75	2020	2021	
JAMES SL	2016	18.75	2020	2021	
LEUCHT S	2016	18.7	2016	2017	
WING RR	2016	18.7	2016	2018	
PEREIRA AC	2016	18.39	2016	2018	
MOUSSAVI S	2016	18.24	2017	2018	
QIU JY	2016	18.23	2020	2021	

M. Top 25 references with strongest beginning year of citation busts (1988-2021)

References	Year	Strength	Begin	End	1980 - 2021
Colcombe S, 2003, PSYCHOL SCI, V14, P125, DOI 10.1111/1467-9280.t01-1-01430, DOI	2003	43.14	2004	2008	
Dunn AL, 2005, AM J PREV MED, V28, P1, DOI 10.1016/j.amepre.2004.09.003, DOI	2005	65.98	2005	2010	
Weuve J, 2004, JAMA-J AM MED ASSOC, V292, P1454, DOI 10.1001/jama.292.12.1454, DOI	2004	45.29	2005	2009	
Rovio S, 2005, LANCET NEUROL, V4, P705, DOI 10.1016/S1474-4422(05)70198-8, DOI	2005	54.62	2006	2010	
Podewils LJ, 2005, AM J EPIDEMIOL, V161, P639, DOI 10.1093/aje/kwi092, DOI	2005	48.8	2006	2010	
Larson EB, 2006, ANN INTERN MED, V144, P73, DOI 10.7326/0003-4819-144-2-200601170-00004, DOI	2006	77.98	2007	2011	
Ogden CL, 2006, JAMA-J AM MED ASSOC, V295, P1549, DOI 10.1001/jama.295.13.1549, DOI	2006	42.31	2007	2011	
Haskell WL, 2007, MED SCI SPORT EXER, V39, P1423 DOI 10.1249/mss.0b013e3180616b27	2007	70.39	2008	2012	
Blumenthal JA, 2007, PSYCHOSOM MED, V69, P587, DOI 10.1097/PSY.0b013e318148c19a, DOI	2007	53.85	2008	2012	
Nelson ME, 2007, CIRCULATION, V116, P1094 DOI 10.1161/CIRCULATIONAHA.107.185650	2007	48.35	2008	2012	
Cotman CW, 2007, TRENDS NEUROSCI, V30, P464, DOI 10.1016/j.tins.2007.06.011, DOI	2007	41.19	2008	2012	
Lautenschlager NT, 2008, JAMA-J AM MED ASSOC, V300, P1027, DOI 10.1001/jama.300.9.1027, DOI	2008	88.03	2009	2013	
Hillman CH, 2008, NAT REV NEUROSCI, V9, P58, DOI 10.1038/nrn2298, DOI	2008	47.93	2009	2013	
Strohle A, 2009, J NEURAL TRANSM, V116, P777, DOI 10.1007/s00702-008-0092-x, DOI	2009	46.32	2010	2014	
Scarmeas N, 2009, JAMA-J AM MED ASSOC, V302, P627, DOI 10.1001/jama.2009.1144, DOI	2009	45.29	2010	2014	
Baker LD, 2010, ARCH NEUROL-CHICAGO, V67, P71, DOI 10.1001/archneurol.2009.307, DOI	2010	62.18	2011	2015	
Smith PJ, 2010, PSYCHOSOM MED, V72, P239, DOI 10.1097/PSY.0b013e3181d14633, DOI	2010	48.06	2011	2015	
Luppino FS, 2010, ARCH GEN PSYCHIAT, V67, P220, DOI 10.1001/archgenpsychiatry.2010.2, DOI	2010	47.57	2011	2015	
Erickson KI, 2011, P NATL ACAD SCI USA, V108, P3017, DOI 10.1073/pnas.1015950108, DOI	2011	110.76	2012	2016	
Barnes DE, 2011, LANCET NEUROL, V10, P819, DOI 10.1016/S1474-4422(11)70072-2, DOI	2011	44.71	2012	2016	
Sofi F, 2011, J INTERN MED, V269, P107, DOI 10.1111/j.1365-2796.2010.02281.x, DOI	2011	41.97	2012	2016	
Lee IM, 2012, LANCET, V380, P219, DOI 10.1016/S0140-6736(12)61031-9, DOI	2012	59.65	2013	2017	
Rimer J, 2012, COCHRANE DB SYST REV, V0, P0, DOI 10.1002/14651858.CD004366.pub5, DOI	2012	41.71	2013	2017	
Cooney GM, 2013, COCHRANE DB SYST REV, V0, P0, DOI 10.1002/14651858.CD004366.pub6, DOI	2013	49.79	2014	2018	
Norton S, 2014, LANCET NEUROL, V13, P788, DOI 10.1016/S1474-4422(14)70136-X, DOI	2014	58.28	2015	2019	

N. Top 25 references sorted by strengths of burst (1988-2021)

References	Year	Strength	Begin	End	1980 - 2021
Erickson KI, 2011, P NATL ACAD SCI USA, V108, P3017, DOI 10.1073/pnas.1015950108, DOI	2011	110.76	2012	2016	
Lautenschlager NT, 2008, JAMA-J AM MED ASSOC, V300, P1027, DOI 10.1001/jama.300.9.1027, DOI	2008	88.03	2009	2013	
Larson EB, 2006, ANN INTERN MED, V144, P73, DOI 10.7326/0003-4819-144-2-200601170-00004, DOI	2006	77.98	2007	2011	
Haskell WL, 2007, MED SCI SPORT EXER, V39, P1423 DOI 10.1249/mss.0b013e3180616b27	2007	70.39	2008	2012	
Dunn AL, 2005, AM J PREV MED, V28, P1, DOI 10.1016/j.amepre.2004.09.003, DOI	2005	65.98	2005	2010	
Baker LD, 2010, ARCH NEUROL-CHICAGO, V67, P71, DOI 10.1001/archneurol.2009.307, DOI	2010	62.18	2011	2015	
Lee IM, 2012, LANCET, V380, P219, DOI 10.1016/S0140-6736(12)61031-9, DOI	2012	59.65	2013	2017	
Norton S, 2014, LANCET NEUROL, V13, P788, DOI 10.1016/S1474-4422(14)70136-X, DOI	2014	58.28	2015	2019	
Rovio S, 2005, LANCET NEUROL, V4, P705, DOI 10.1016/S1474-4422(05)70198-8, DOI	2005	54.62	2006	2010	
Blumenthal JA, 2007, PSYCHOSOM MED, V69, P587, DOI 10.1097/PSY.0b013e318148c19a, DOI	2007	53.85	2008	2012	
Rosenbaum S, 2014, J CLIN PSYCHIAT, V75, P964, DOI 10.4088/JCP.13r08765, DOI	2014	51.44	2015	2019	
Cooney GM, 2013, COCHRANE DB SYST REV, V0, P0, DOI 10.1002/14651858.CD004366.pub6, DOI	2013	49.79	2014	2018	
Podewils LJ, 2005, AM J EPIDEMIOL, V161, P639, DOI 10.1093/aje/kwi092, DOI	2005	48.8	2006	2010	
Nelson ME, 2007, CIRCULATION, V116, P1094 DOI 10.1161/CIRCULATIONAHA.107.185650	2007	48.35	2008	2012	
Smith PJ, 2010, PSYCHOSOM MED, V72, P239, DOI 10.1097/PSY.0b013e3181d14633, DOI	2010	48.06	2011	2015	
Hillman CH, 2008, NAT REV NEUROSCI, V9, P58, DOI 10.1038/nrn2298, DOI	2008	47.93	2009	2013	
Luppino FS, 2010, ARCH GEN PSYCHIAT, V67, P220, DOI 10.1001/archgenpsychiatry.2010.2, DOI	2010	47.57	2011	2015	
Strohle A, 2009, J NEURAL TRANSM, V116, P777, DOI 10.1007/s00702-008-0092-x, DOI	2009	46.32	2010	2014	
Weuve J, 2004, JAMA-J AM MED ASSOC, V292, P1454, DOI 10.1001/jama.292.12.1454, DOI	2004	45.29	2005	2009	
Scarmeas N, 2009, JAMA-J AM MED ASSOC, V302, P627, DOI 10.1001/jama.2009.1144, DOI	2009	45.29	2010	2014	
Barnes DE, 2011, LANCET NEUROL, V10, P819, DOI 10.1016/S1474-4422(11)70072-2, DOI	2011	44.71	2012	2016	
Colcombe S, 2003, PSYCHOL SCI, V14, P125, DOI 10.1111/1467-9280.t01-1-01430, DOI	2003	43.14	2004	2008	
Ogden CL, 2006, JAMA-J AM MED ASSOC, V295, P1549, DOI 10.1001/jama.295.13.1549, DOI	2006	42.31	2007	2011	
Sofi F, 2011, J INTERN MED, V269, P107, DOI 10.1111/j.1365-2796.2010.02281.x, DOI	2011	41.97	2012	2016	

O. Top 25 cited references with strongest beginning of citation busts (2016-2021)

References	Year	Strength	Begin	End	2016 - 2021
Mammen G, 2013, AM J PREV MED, V45, P649, DOI 10.1016/j.amepre.2013.08.001, DOI	2013	37.15	2016	2018	
Lee IM, 2012, LANCET, V380, P219, DOI 10.1016/S0140-6736(12)61031-9, DOI	2012	32.04	2016	2017	
Cooney GM, 2013, COCHRANE DB SYST REV, V0, P0, DOI 10.1002/14651858.CD004366.pub6, DOI	2013	28.22	2016	2018	
Vancampfort D, 2012, ACTA PSYCHIAT SCAND, V125, P352, DOI 10.1111/j.1600-0447.2011.01814.x, DOI	2012	21.8	2016	2017	
Spruit MA, 2013, AM J RESP CRIT CARE, V188, P0, DOI 10.1164/rccm.201309-1634ST, DOI	2013	19.96	2016	2018	
Ferrari AJ, 2013, PLOS MED, V10, P0, DOI 10.1371/journal.pmed.1001547, DOI	2013	19.96	2016	2018	
Rosenbaum S, 2014, J CLIN PSYCHIAT, V75, P964, DOI 10.4088/JCP.13r08765, DOI	2014	19.76	2016	2019	
Hallal PC, 2012, LANCET, V380, P247, DOI 10.1016/S0140-6736(12)60646-1, DOI	2012	19.48	2016	2017	
Prince M, 2013, ALZHEIMERS DEMENT, V9, P63, DOI 10.1016/j.jalz.2012.11.007, DOI	2013	19.32	2016	2018	
Higgins J, 2012, COCHRANE DB SYST REV, V0, P0, DOI 10.1002/14651858.ED000049, DOI	2012	19.01	2016	2017	
Whiteford HA, 2013, LANCET, V382, P1575, DOI 10.1016/S0140-6736(13)61611-6, DOI	2013	18.69	2016	2018	
Ogden CL, 2014, JAMA-J AM MED ASSOC, V311, P806, DOI 10.1001/jama.2014.732, DOI	2014	18.05	2016	2018	
Barnes J, 2012, APPL PHYSIOL NUTR ME, V37, P540 DOI 10.1590/1980-549720190050	2012	17.62	2016	2017	
Buchman AS, 2012, NEUROLOGY, V78, P1323, DOI 10.1212/WNL.0b013e3182535d35, DOI	2012	17.62	2016	2017	
McCrory P, 2013, BRIT J SPORT MED, V47, P250, DOI 10.1136/bjsports-2013-092313, DOI	2013	17.42	2016	2018	
Bridle C, 2012, BRIT J PSYCHIAT, V201, P180, DOI 10.1192/bjp.bp.111.095174, DOI	2012	16.69	2016	2017	
McKee AC, 2013, BRAIN, V136, P43, DOI 10.1093/brain/aws307, DOI	2013	16.46	2016	2018	
Clegg A, 2013, LANCET, V381, P752, DOI 10.1016/S0140-6736(12)62167-9, DOI	2013	16.15	2016	2018	
Norton S, 2014, LANCET NEUROL, V13, P788, DOI 10.1016/S1474-4422(14)70136-X, DOI	2014	24.09	2017	2019	
Hartig T, 2014, ANNU REV PUBL HEALTH, V35, P207, DOI 10.1146/annurev-publhealth-032013-182443, DOI	2014	18.93	2017	2019	
Correll CU, 2017, WORLD PSYCHIATRY, V16, P163, DOI 10.1002/wps.20420, DOI	2017	18.36	2018	2019	
Schuch FB, 2018, AM J PSYCHIAT, V175, P631, DOI 10.1176/appi.ajp.2018.17111194, DOI	2018	17.31	2019	2020	
Livingston G, 2017, LANCET, V390, P2673, DOI 10.1016/S0140-6736(17)31363-6, DOI	2017	17.04	2019	2020	
Brooks SK, 2020, LANCET, V395, P912 DOI 10.1016/S0140-6736(20)30460-8	2020	38.71	2020	2021	
Wang CY, 2020, INT J ENV RES PUB HE, V17, P0, DOI 10.3390/ijerph17051729, DOI	2020	28.81	2020	2021	

P. Top 23 references sorted by strengths of burst (2016-2021)

References	Year	Strength	Begin	End	2016 - 2021
Brooks SK, 2020, LANCET, V395, P912	2020	38.71	2020	2021	
Mammen G, 2013, AM J PREV MED, V45, P649, DOI 10.1016/j.amepre.2013.08.001, DOI	2013	37.15	2016	2018	
Lee IM, 2012, LANCET, V380, P219, DOI 10.1016/S0140-6736(12)61031-9, DOI	2012	32.04	2016	2017	
Wang CY, 2020, INT J ENV RES PUB HE, V17, P0, DOI 10.3390/ijerph17051729, DOI	2020	28.81	2020	2021	
Cooney GM, 2013, COCHRANE DB SYST REV, V0, P0, DOI 10.1002/14651858.CD004366.pub6, DOI	2013	28.22	2016	2018	
Norton S, 2014, LANCET NEUROL, V13, P788, DOI 10.1016/S1474-4422(14)70136-X, DOI	2014	24.09	2017	2019	
Vancampfort D, 2012, ACTA PSYCHIAT SCAND, V125, P352, DOI 10.1111/j.1600-0447.2011.01814.x, DOI	2012	21.8	2016	2017	
Holmes EA, 2020, LANCET PSYCHIAT, V7, P547, DOI 10.1016/S2215-0366(20)30168-1, DOI	2020	21.05	2020	2021	
Spruit MA, 2013, AM J RESP CRIT CARE, V188, P0, DOI 10.1164/rccm.201309-1634ST, DOI	2013	19.96	2016	2018	
Ferrari AJ, 2013, PLOS MED, V10, P0, DOI 10.1371/journal.pmed.1001547, DOI	2013	19.96	2016	2018	
Rosenbaum S, 2014, J CLIN PSYCHIAT, V75, P964, DOI 10.4088/JCP.13r08765, DOI	2014	19.76	2016	2019	
Hallal PC, 2012, LANCET, V380, P247, DOI 10.1016/S0140-6736(12)60646-1, DOI	2012	19.48	2016	2017	
Prince M, 2013, ALZHEIMERS DEMENT, V9, P63, DOI 10.1016/j.jalz.2012.11.007, DOI	2013	19.32	2016	2018	
Hartig T, 2014, ANNU REV PUBL HEALTH, V35, P207, DOI 10.1146/annurev-publhealth-032013-182443, DOI	2014	18.93	2017	2019	
Whiteford HA, 2013, LANCET, V382, P1575, DOI 10.1016/S0140-6736(13)61611-6, DOI	2013	18.69	2016	2018	
Correll CU, 2017, WORLD PSYCHIATRY, V16, P163, DOI 10.1002/wps.20420, DOI	2017	18.36	2018	2019	
Ogden CL, 2014, JAMA-J AM MED ASSOC, V311, P806, DOI 10.1001/jama.2014.732, DOI	2014	18.05	2016	2018	
Buchman AS, 2012, NEUROLOGY, V78, P1323, DOI 10.1212/WNL.0b013e3182535d35, DOI	2012	17.62	2016	2017	
McCrory P, 2013, BRIT J SPORT MED, V47, P250, DOI 10.1136/bjsports-2013-092313, DOI	2013	17.42	2016	2018	
Schuch FB, 2018, AM J PSYCHIAT, V175, P631, DOI 10.1176/appi.ajp.2018.17111194, DOI	2018	17.31	2019	2020	
Livingston G, 2017, LANCET, V390, P2673, DOI 10.1016/S0140-6736(17)31363-6, DOI	2017	17.04	2019	2020	
Bridle C, 2012, BRIT J PSYCHIAT, V201, P180, DOI 10.1192/bjp.bp.111.095174, DOI	2012	16.69	2016	2017	
McKee AC, 2013, BRAIN, V136, P43, DOI 10.1093/brain/aws307, DOI	2013	16.46	2016	2018	

Q. Top 24 cited references with strongest beginning of citation bursts (2021)

References	Year	Strength	Begin	End	2021-JAN - 2021-DEC
Rajkumar RP, 2020, ASIAN J PSYCHIATR, V52, P0, DOI 10.1016/j.ajp.2020.102066, DOI	2020-JAN	5.21	2021-JAN	2021-MAY	
Zhu N, 2020, NEW ENGL J MED, V382, P727, DOI 10.1056/NEJMoa2001017, DOI	2020-JAN	4.85	2021-JAN	2021-MAR	
Vos T, 2016, LANCET, V388, P1545, DOI 10.1016/S0140-6736(16)31678-6, DOI	2016-JAN	3.82	2021-JAN	2021-FEB	
Cellini N, 2020, J SLEEP RES, V29, P0, DOI 10.1111/jsr.13074, DOI	2020-JAN	3.82	2021-JAN	2021-FEB	
Brooks SK, 2020, LANCET, V395, P912, DOI 10.1016/S0140-6736(20)30460-8, DOI	2020-JAN	7.41	2021-FEB	2021-MAR	
EASL, 2016, DIABETOLOGIA, V59, P1121, DOI 10.1007/s00125-016-3902-y, DOI	2016-JAN	4.18	2021-FEB	2021-MAR	
Altena E, 2020, J SLEEP RES, V29, P0, DOI 10.1111/jsr.13052, DOI	2020-JAN	3.84	2021-FEB	2021-APR	
Qiu JY, 2020, GEN PSYCHIAT, V33, P0, DOI 10.1136/gpsych-2020-100213, DOI	2020-JAN	3.53	2021-FEB	2021-MAR	
Jimenez-Pavon D, 2020, PROG CARDIOVASC DIS, V63, P386, DOI 10.1016/j.pcad.2020.03.009, DOI	2020-JAN	4.54	2021-MAR	2021-JUN	
Mazza C, 2020, INT J ENV RES PUB HE, V17, P0, DOI 10.3390/ijerph17093165, DOI	2020-JAN	4.33	2021-MAR	2021-JUN	
Wang CY, 2020, BRAIN BEHAV IMMUN, V87, P40, DOI 10.1016/j.bbi.2020.04.028, DOI	2020-JAN	3.83	2021-MAR	2021-APR	
Chen PJ, 2020, J SPORT HEALTH SCI, V9, P103, DOI 10.1016/j.jshs.2020.02.001, DOI	2020-JAN	5.72	2021-APR	2021-MAY	
Bao YP, 2020, LANCET, V395, P0, DOI 10.1016/S0140-6736(20)30309-3, DOI	2020-JAN	4.78	2021-APR	2021-MAY	
Lavie CJ, 2019, CIRC RES, V124, P799, DOI 10.1161/CIRCRESAHA.118.312669, DOI	2019-JAN	4.08	2021-MAY	2021-JUN	
Zhou SJ, 2020, EUR CHILD ADOLES PSY, V29, P749, DOI 10.1007/s00787-020-01541-4, DOI	2020-JAN	3.57	2021-MAY	2021-JUN	
Schuch FB, 2020, PSYCHIAT RES, V292, P0, DOI 10.1016/j.psychres.2020.113339, DOI	2020-JAN	3.98	2021-JUN	2021-SEP	
Iverson GL, 2017, BRIT J SPORT MED, V51, P0, DOI 10.1136/bjsports-2017-097729, DOI	2017-JAN	4.49	2021-AUG	2021-SEP	
Xiong JQ, 2020, J AFFECT DISORDERS, V277, P55, DOI 10.1016/j.jad.2020.08.001, DOI	2020-JAN	4.18	2021-AUG	2021-OCT	
Dadvand P, 2016, ENVIRON INT, V91, P161, DOI 10.1016/j.envint.2016.02.029, DOI	2016-JAN	4.04	2021-AUG	2021-SEP	
Guessoum SB, 2020, PSYCHIAT RES, V291, P0, DOI 10.1016/j.psychres.2020.113264, DOI	2020-JAN	3.83	2021-AUG	2021-DEC	
Twohig-Bennett C, 2018, ENVIRON RES, V166, P628, DOI 10.1016/j.envres.2018.06.030, DOI	2018-JAN	4.51	2021-SEP	2021-DEC	
Warburton DER, 2017, CURR OPIN CARDIOL, V32, P541, DOI 10.1097/HCO.0000000000000437, DOI	2017-JAN	3.97	2021-SEP	2021-OCT	
Brooks SK, 2020, LANCET, V395, P912	2020-JAN	5.92	2021-OCT	2021-DEC	
Stubbs B, 2018, EUR PSYCHIAT, V54, P124, DOI 10.1016/j.eurpsy.2018.07.004, DOI	2018-JAN	4.37	2021-OCT	2021-DEC	

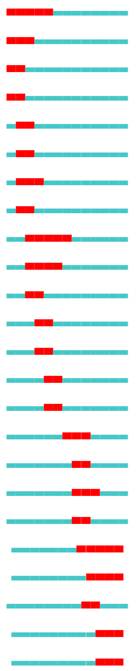

R. Top 24 references with the strongest strength of citation bursts (2021)

References	Year	Strength	Begin	End	2021-JAN - 2021-DEC
Cumpston M, 2019, COCHRANE DB SYST REV, V0, P0, DOI 10.1002/14651858.ED000142, DOI	2019-JAN	9.89	2021-SEP	2021-DEC	
Brooks SK, 2020, LANCET, V395, P912, DOI 10.1016/S0140-6736(20)30460-8, DOI	2020-JAN	5.92	2021-OCT	2021-DEC	
Chen PJ, 2020, J SPORT HEALTH SCI, V9, P103, DOI 10.1016/j.jshs.2020.02.001, DOI	2020-JAN	5.72	2021-APR	2021-MAY	
Rajkumar RP, 2020, ASIAN J PSYCHIATR, V52, P0, DOI 10.1016/j.ajp.2020.102066, DOI	2020-JAN	5.21	2021-JAN	2021-MAY	
Zhu N, 2020, NEW ENGL J MED, V382, P727, DOI 10.1056/NEJMoa2001017, DOI	2020-JAN	4.85	2021-JAN	2021-MAR	
Bao YP, 2020, LANCET, V395, P0, DOI 10.1016/S0140-6736(20)30309-3, DOI	2020-JAN	4.78	2021-APR	2021-MAY	
Huang C, 2020, LIM ANAL THEOR SOIL, V395, P497, DOI 10.1007/978-981-15-1572-9_1], DOI	2020-JAN	4.56	2021-APR	2021-JUN	
Jimenez-Pavon D, 2020, PROG CARDIOVASC DIS, V63, P386, DOI 10.1016/j.pcad.2020.03.009, DOI	2020-JAN	4.54	2021-MAR	2021-JUN	
Twohig-Bennett C, 2018, ENVIRON RES, V166, P628, DOI 10.1016/j.envres.2018.06.030, DOI	2018-JAN	4.51	2021-SEP	2021-DEC	
Iverson GL, 2017, BRIT J SPORT MED, V51, P0, DOI 10.1136/bjsports-2017-097729, DOI	2017-JAN	4.49	2021-AUG	2021-SEP	
Stubbs B, 2018, EUR PSYCHIAT, V54, P124, DOI 10.1016/j.eurpsy.2018.07.004, DOI	2018-JAN	4.37	2021-OCT	2021-DEC	
Mazza C, 2020, INT J ENV RES PUB HE, V17, P0, DOI 10.3390/ijerph17093165, DOI	2020-JAN	4.33	2021-MAR	2021-JUN	
EASL, 2016, DIABETOLOGIA, V59, P1121, DOI 10.1007/s00125-016-3902-y, DOI	2016-JAN	4.18	2021-FEB	2021-MAR	
Xiong JQ, 2020, J AFFECT DISORDERS, V277, P55, DOI 10.1016/j.jad.2020.08.001, DOI	2020-JAN	4.18	2021-AUG	2021-OCT	
Lavie CJ, 2019, CIRC RES, V124, P799, DOI 10.1161/CIRCRESAHA.118.312669, DOI	2019-JAN	4.08	2021-MAY	2021-JUN	
Dadvand P, 2016, ENVIRON INT, V91, P161, DOI 10.1016/j.envint.2016.02.029, DOI	2016-JAN	4.04	2021-AUG	2021-SEP	
Schuch FB, 2020, PSYCHIAT RES, V292, P0, DOI 10.1016/j.psychres.2020.113339, DOI	2020-JAN	3.98	2021-JUN	2021-SEP	
Warburton DER, 2017, CURR OPIN CARDIOL, V32, P541, DOI 10.1097/HCO.0000000000000437, DOI	2017-JAN	3.97	2021-SEP	2021-OCT	
Altena E, 2020, J SLEEP RES, V29, P0, DOI 10.1111/jsr.13052, DOI	2020-JAN	3.84	2021-FEB	2021-APR	
Guessoum SB, 2020, PSYCHIAT RES, V291, P0, DOI 10.1016/j.psychres.2020.113264, DOI	2020-JAN	3.83	2021-AUG	2021-DEC	
Wang CY, 2020, BRAIN BEHAV IMMUN, V87, P40, DOI 10.1016/j.bbi.2020.04.028, DOI	2020-JAN	3.83	2021-MAR	2021-APR	
Vos T, 2016, LANCET, V388, P1545, DOI 10.1016/S0140-6736(16)31678-6, DOI	2016-JAN	3.82	2021-JAN	2021-FEB	
Cellini N, 2020, J SLEEP RES, V29, P0, DOI 10.1111/jsr.13074, DOI	2020-JAN	3.82	2021-JAN	2021-FEB	
Zhou SJ, 2020, EUR CHILD ADOLES PSY, V29, P749, DOI 10.1007/s00787-020-01541-4, DOI	2020-JAN	3.57	2021-MAY	2021-JUN	

S. Top 30 cited keywords with strongest beginning of citation busts (1988-2021)

Keywords	Year	Strength	Begin	End	1990 - 2021
acute myocardial infarction	1990	74.86	1990	2008	
response	1990	60.67	1990	2004	
exercise	1990	56.37	1990	1998	
chest pain	1990	38.44	1990	2003	
coronary artery disease	1990	297.29	1991	2006	
men	1990	95.04	1991	2008	
angina pectori	1990	90.73	1991	2005	
st segment depression	1990	87.22	1991	2006	
ischemia	1990	83.53	1991	2008	
myocardial infarction	1990	81.04	1991	2008	
myocardial ischemia	1990	77.33	1991	2005	
heart disease	1990	76.94	1991	2001	
double blind	1990	76.24	1991	2011	
infarction	1990	71.48	1991	2003	
coronary heart disease	1990	68.62	1991	2011	
artery disease	1990	64.99	1991	2006	
pectori	1990	62.7	1991	2003	
anorexia nervosa	1990	53.82	1991	2008	
follow up	1990	52.36	1991	2004	
rat	1990	47.98	1991	2010	
trial	1990	45.31	1991	2004	
eating disorder	1990	45.02	1991	2007	
angina	1990	44.05	1991	2005	
nifedipine	1990	40.87	1991	2001	
women	1990	76.96	1993	2006	
quality of life	1990	56.64	1995	2003	
major depression	1990	49.34	1998	2012	
controlled trial	1990	47.28	1999	2012	
metaanalysis	1990	45.45	2017	2018	
sedentary behavior	1990	43.55	2018	2021	

T. Top 30 keywords with the strongest strength of citation bursts (1988-2021)

Keywords	Year	Strength	Begin	End	1990 - 2021
double blind	1988	76.25	1991	2011	
coronary heart disease	1988	68.62	1991	2011	
rat	1988	47.98	1991	2010	
acute myocardial infarction	1988	74.86	1990	2008	
men	1988	95.04	1991	2008	
ischemia	1988	83.53	1991	2008	
myocardial infarction	1988	81.04	1991	2008	
anorexia nervosa	1988	53.82	1991	2008	
eating disorder	1988	45.02	1991	2007	
coronary artery disease	1988	297.29	1991	2006	
st segment depression	1988	87.22	1991	2006	
artery disease	1988	64.99	1991	2006	
angina pectori	1988	90.73	1991	2005	
myocardial ischemia	1988	77.33	1991	2005	
response	1988	60.67	1990	2004	
major depression	1988	49.34	1998	2012	
angina	1988	44.05	1991	2005	
women	1988	76.96	1993	2006	
follow up	1988	52.36	1991	2004	
controlled trial	1988	47.28	1999	2012	
trial	1988	45.31	1991	2004	
chest pain	1988	38.44	1990	2003	
infarction	1988	71.48	1991	2003	
pectori	1988	62.7	1991	2003	
heart disease	1988	76.94	1991	2001	
nifedipine	1988	40.87	1991	2001	
of life	1988	56.64	1995	2003	
exercise	1988	56.37	1990	1998	
sedentary behavior	1988	43.55	2018	2021	
metaanalysis	1988	45.45	2017	2018	

U. Top 30 cited keywords with strongest beginning of citation bursts (2016-2021)

Keywords	Year	Strength	Begin	End	2016 - 2021
randomized controlled trial	2016	27.24	2016	2018	
prospective cohort	2016	12.69	2016	2018	
placebo controlled trial	2016	11.37	2016	2018	
us adult	2016	7.67	2016	2018	
serious mental illness	2016	7.14	2016	2018	
in vivo	2016	7.14	2016	2018	
vascular dementia	2016	6.87	2016	2018	
womens health	2016	6.83	2016	2017	
physiotherapy	2016	6.83	2016	2017	
synaptic plasticity	2016	6.61	2016	2017	
environmental enrichment	2016	6.41	2016	2018	
atherosclerosis	2016	6.41	2016	2017	
marital status	2016	6.41	2016	2017	
agreement	2016	6.34	2016	2018	
human	2016	6.15	2016	2018	
general population	2016	8.67	2017	2018	
sf 36 health survey	2016	6.61	2017	2018	
occupational therapy	2016	6.22	2017	2018	
power	2016	6.61	2018	2019	
prostate cancer	2016	6.61	2018	2019	
amyloid beta	2016	7.75	2019	2020	
cognitive therapy	2016	7.13	2019	2020	
quarantine	2016	10.41	2020	2021	
sar	2016	9.49	2020	2021	
generalized anxiety disorder	2016	8.05	2020	2021	
psychological impact	2016	7.65	2020	2021	
acute respiratory syndrome	2016	7.04	2020	2021	
rat model	2016	6.43	2020	2021	
epidemic	2016	6.12	2020	2021	
deficiency	2016	6.12	2020	2021	

V. Top 25 keywords with the strongest strength of citation bursts (2016-2021)

Keywords	Year	Strength	Begin	End	2016 - 2021
randomized controlled trial	2016	27.24	2016	2018	
prospective cohort	2016	12.69	2016	2018	
placebo controlled trial	2016	11.37	2016	2018	
quarantine	2016	10.41	2020	2021	
sar	2016	9.49	2020	2021	
general population	2016	8.67	2017	2018	
generalized anxiety disorder	2016	8.05	2020	2021	
amyloid beta	2016	7.75	2019	2020	
us adult	2016	7.67	2016	2018	
psychological impact	2016	7.65	2020	2021	
serious mental illness	2016	7.14	2016	2018	
in vivo	2016	7.14	2016	2018	
cognitive therapy	2016	7.13	2019	2020	
acute respiratory syndrome	2016	7.04	2020	2021	
vascular dementia	2016	6.87	2016	2018	
womens health	2016	6.83	2016	2017	
physiotherapy	2016	6.83	2016	2017	
sf 36 health survey	2016	6.61	2017	2018	
synaptic plasticity	2016	6.61	2016	2017	
power	2016	6.61	2018	2019	
prostate cancer	2016	6.61	2018	2019	
rat model	2016	6.43	2020	2021	
environmental enrichment	2016	6.41	2016	2018	
atherosclerosis	2016	6.41	2016	2017	
marital status	2016	6.41	2016	2017	
agreement	2016	6.34	2016	2018	
occupational therapy	2016	6.22	2017	2018	
human	2016	6.15	2016	2018	
epidemic	2016	6.12	2020	2021	
deficiency	2016	6.12	2020	2021	

Supplementary Table 3. Top 10 articles with the strongest centrality divergence scores for the 2016-2021 and the 2021-2021 time periods.

Number of current citations in the world (Feb 2021)	Modularity divergence	Centrality divergence	Authors	Journal	Title	DOI
**January 2016 to May 2022**						
60	8.15	0.08	Stubbs B, 2017	Schizophrenia Bulletin	Physical Activity Levels and Psychosis: A Mediation Analysis of Factors Influencing Physical Activity Target Achievement Among 204 186 People Across 46 Low- and Middle-Income Countries	10.1093/schbul/sbw111
461	7.17	0.11	Vancampfort D, 2017	World Psychiatry	Sedentary behavior and physical activity levels in people with schizophrenia, bipolar disorder and major depressive disorder: a global systematic review and meta-analysis	10.1002/wps.20458
24	5.94	0.03	Vancampfort D, 2017,	Journal of Affective Disorders	Physical activity and metabolic disease among people with affective disorders: Prevention, management and implementation	10.1016/j.jad.2016.07.042
363	5.51	0.10	Schuch F, 2017	Journal of Affective Disorders	Physical activity and sedentary behavior in people with major depressive disorder: A systematic review and meta-analysis	10.1016/j.jad.2016.10.050
14	3.87	0.11	Stubbs B, 2017	Psychiatry Research	Bidirectional longitudinal relationship between leisure-time physical activity and psychotropic medication usage: A register linked follow-up study	10.1016/j.psychres.2016.11.03 3
185	3.55	0.01	Vancampfort D, 2017	Sports Medicine	Cardiorespiratory Fitness in Severe Mental Illness: A Systematic Review and Meta-analysis	10.1007/s40279-016-0574-1
139	3.38	0.11	Ashdown-Franks G, 2020,	Sports Medicine	Exercise as Medicine for Mental and Substance Use Disorders: A Meta-review of the Benefits for Neuropsychiatric and Cognitive Outcomes	10.1007/s40279-019-01187-6
366	3.11	0.06	Firth J, 2017	Schizophrenia Bulletin	Aerobic Exercise Improves Cognitive Functioning in People With Schizophrenia: A Systematic Review and Meta-Analysis	10.1093/schbul/sbw115
23	2.76	0.11	Campos C, 2017	CNS & Neurological Disorders	Exercise Induced Neuroplasticity to Enhance Therapeutic Outcomes of Cognitive Remediation in Schizophrenia: Analyzing the Role of Brain derived Neurotrophic Factor	10.2174/187152731566616122 3142918
87	2.23	0.05	Kandola A, 2018	Current Psychiatry Reports	Moving to Beat Anxiety: Epidemiology and Therapeutic Issues with Physical Activity for Anxiety	10.1007/s11920-018-0923-x
**January 2021 to May 2022**						
4	5.20	0.21	Aguilar B A S, 2021	European Journal of Sport Science	Leisure-time exercise is associated with lower depressive symptoms in community dwelling adults	10.1080/17461391.2021.18958 92
2	5.04	0.04	Schuch F B, 2021	Journal of Psychiatry Research	Cross-sectional associations of leisure and transport related physical activity with depression and anxiety	10.1016/j.jpsychires.2021.05.0 53
18	4.17	0.04	van Sluijs E M F, 2021	The Lancet	Physical activity behaviours in adolescence: current evidence and opportunities for intervention	10.1016/S0140-6736(21)01259-9
3	3.21	0.03	Kandola A A, 2021	BMC Medicine	Impact of replacing sedentary behaviour with other movement behaviours on depression and anxiety symptoms: a prospective cohort study in the UK Biobank	10.1186/s12916-021-02007-3
1	2.97	0.15	Dotson V M, 2021	Journal of the International Neuropsychological Society	Be Fit, Be Sharp, Be Well: The Case for Exercise as a Treatment for Cognitive Impairment in Late-life Depression	10.1017/S1355617721000710
27	2.69	0.17	Werneck A O, 2021	The Journal of Psychosomatic Research	Physical inactivity and elevated TV-viewing reported changes during the COVID-19 pandemic are associated with mental health: A survey with 43,995 Brazilian adults	10.1016/j.jpsychores.2020.110 292
0	2.46	0.19	Puterman Eli, 2021	British Journal of Sports Medicine	COVID-19 Pandemic and Exercise (COPE) trial: a multigroup pragmatic randomised controlled trial examining effects of app-based at-home exercise programs on depressive symptoms	10.1136/bjsports-2021-104379
0	2.24	0.01	Seet V, 2021	BMC Psychiatry	Physical activity, sedentary behaviour and smoking status among psychiatric patients in Singapore – a cross-sectional study	10.1186/s12888-021-03103-7
1	2.12	0.05	Golshani S, 2021	Healthcare (Basel)	When Much Is Too Much—Compared to Light Exercisers, Heavy Exercisers Report More Mental Health Issues and Stress, but Less Sleep Complaints	10.3390/healthcare9101289
3	2.07	0.16	Felez-Nobrega M, 2021	Age Ageing	Moving more, ageing happy: findings from six low- and middle-income countries	10.1093/ageing/afaa137

Note: The modularity of a network is a measure of the overall structure of the network and permits to identify the transformative papers. Its range is between −1 and 1. The modularity change rate of a scientific paper measures the relative structural change due to the information from the published paper with reference to a baseline network. The centrality divergence metric measures the structural variation caused by an article a in terms of the divergence of the distribution of betweenness centrality CB(vi) of nodes vi in the baseline network. The centrality divergence metric is potentially valuable for detecting boundary-spanning activities at interdisciplinary levels.

Supplementary Table 4. The top countries, institutions and top cited authors (1988-2021 and 2016-2021 period)

**1988-2021 period**							
**Countries ranked by centrality**	**Degree of centrality**	**Countries ranked by citation counts**	**Total number of citations**	**Institutions ranked by centrality**	**Degree of centrality**	**Institutions ranked by citation counts**	**Total number of citations**
1. United Stated of America	172	1. United Stated of America	17,988	1. Harvard University	123	1. University Toronto	834
2. United Kingdom	111	2. United Kingdom	5,720	2. University Pittsburgh	123	2. University of Melbourne	745
3. Italy	89	3. Australia	4,431	3. University Calif Los Angeles	120	3. Kings College London	717
4. France	79	4. Canada	3,773	4. Columbia University	118	4. University Sydney	690
5. Canada	76	5. Peoples Republic of China	3,160	5. Stanford University	112	5. Karolinska Institutet	660
6. Australia	72	6. Germany	3,131	6. University of Pennsylvania	109	6. Harvard University	646
7. Germany	72	7. Netherlands	2,479	7. Johns Hopkins University	108	7. University Pittsburgh	645
8. Netherlands	72	8. Spain	2,389	8. University of California	105	8. University Washington	643
9. South Africa	70	9. Italy	2,352	9. University of North Carolina	103	9. University College of London	634
10. Switzerland	69	10. Japan	2,055	10. University of California, San Diego	101	10. University of California, San Francisco	625
**2016-2021 period**							
**Countries ranked by Sigma score**	**Degree of centrality**	**Countries ranked by citation counts**	**Total number of citations**	**Institutions ranked by centrality**	**Degree of centrality**	**Institutions ranked by citation counts**	**Total number of citations**
1. Philippines	0.08	1. United Stated of America	7,933	1. Imperial College of London	65	1. Kings College London	497
2. South Africa	0.06	2. United Kingdom	3,035	2. Finnish Institute of Occupational Health	58	2. Harvard Medical School	485
3. Russia	0.06	3. Australia	2,639	3. University College Cork	58	3. University of Melbourne	466
4. Malawi	0.05	4. Peoples Republic of China	2,462	4. University of New Mexico	55	4. University Toronto	465
5. Kenya	0.05	5. Canada	1,980	5. Swansea University	54	5. University Sydney	430
6. Benin	0.05	6. Germany	1,586	6. Medical University of Vienna	51	6. Karolinska Institutet	415
7. Belgium	0.05	7. Spain	1,534	7. Bispebjerg Hospital	51	7. University College of London	376
8. Mexico	0.05	8. Italy	1,245	8. University of Belgrade	50	8. University of California	319
9. United Arab Emirates	0.04	9. Netherlands	1,221	9. Hacettepe University	50	9. University of Queensland	313
10. Zimbabwe	0.04	10. Brazil	1,130	10. University of Melbourne	49	10. University of Pittsburgh	286

**Top authors**								
**Top authors per number of publications (all time)**	**Number of papers in our dataset**	**Worldwide number of citations**	**Top authors cited as first authors (2016-2021)**	**Number of citations**	**Top authors cited as last authors (2016-2021)**	**Number of papers**	**Number of citations**	**Worldwide number of citations**
1. Stubbs B	281	38,186	1. Van Dyck D	3,397	1. Allen C	76	3,482	12,975
2. Vancampfort D	261	28,404	2. Ware J	1,356	2. Vancampfort D	200	2,093	28,858
3. Firth J	88	15,197	3. Cohen J	1,285	3. Stubbs B	369	1,312	38,803
4. Rosenbaum S	76	15,012	4. Folstein M	1,110	4. Bellis M	144	1,054	18,573
5. Koyanagi A	65	71,126	5. Kroenke K	1,069	5. Sawyer S M	73	1,054	31,555
6. Probst M	52	12,045	6. Kessler R	1,066	6. Légaré F	245	1,046	33,890
7. Smith L	47	11,376	7. Zigmond A	981	7. Stevens G	42	914	57,916
8. Schuch FB	42	10,480	8. Vancampfort D	895	8. Hancock M	46	861	13,207
9. De Hert M	40	32,295	9. Craig C	853	9. Stein J	28	852	71,350
10. Ward PB	39	18,250	10. Lee J	825	10. Baglioni C	65	761	4,776

Note: The top countries, institutions and top cited authors are extracted from our database, and do not necessarily reflect the real influence

Supplementary Table 5. Journals with most articles and citations

Top journals						
**Journals with most articles (1980-2021)**	**Initial year**	**Impact factor (2020-2021)**	**Total articles of the dataset (%)**	**Total articles**	**Journals with most citations (1980-2021)**	**Total citations in our dataset**
1. International Journal of Environmental Research and Public Health	2004	3.39	2.1	1164	1. Archives of General Psychiatry (JAMA)	20,557
2. PLOS ONE	2006	3.24	1.8	1017	2. The Lancet	13,884
3. BMC Public Health	2000	3.17	1.1	625	3. PLOS ONE	12,418
4. BMJ OPEN	2011	2.69	0.92	513	4. Medicine and Science in Sports and Exercise	11,568
5. Journal of Affective Disorders	1979	4.83	0.81	453	5. Journal of the American Geriatrics Society	11,203
6. Journal of the American Geriatrics Society	1953	5.56	0.81	448	6. BMJ	8,576
7. Psychology of Sport and Exercise Physiology	1996	4.78	0.57	317	7. Circulation	7,686
8. American Journal of Cardiology	1958	2.77	0.56	313	8. American Journal of Psychiatry	7,823
9. BMC Geriatrics	2001	3.73	0.55	309	9. Archives of Internal Medicine	7,492
10. Frontiers in Psychiatry	2010	3.53	0.51	287	10. American Journal of Epidemiology	6,919

Supplementary Figure 1. Flow chart of the scientometric study

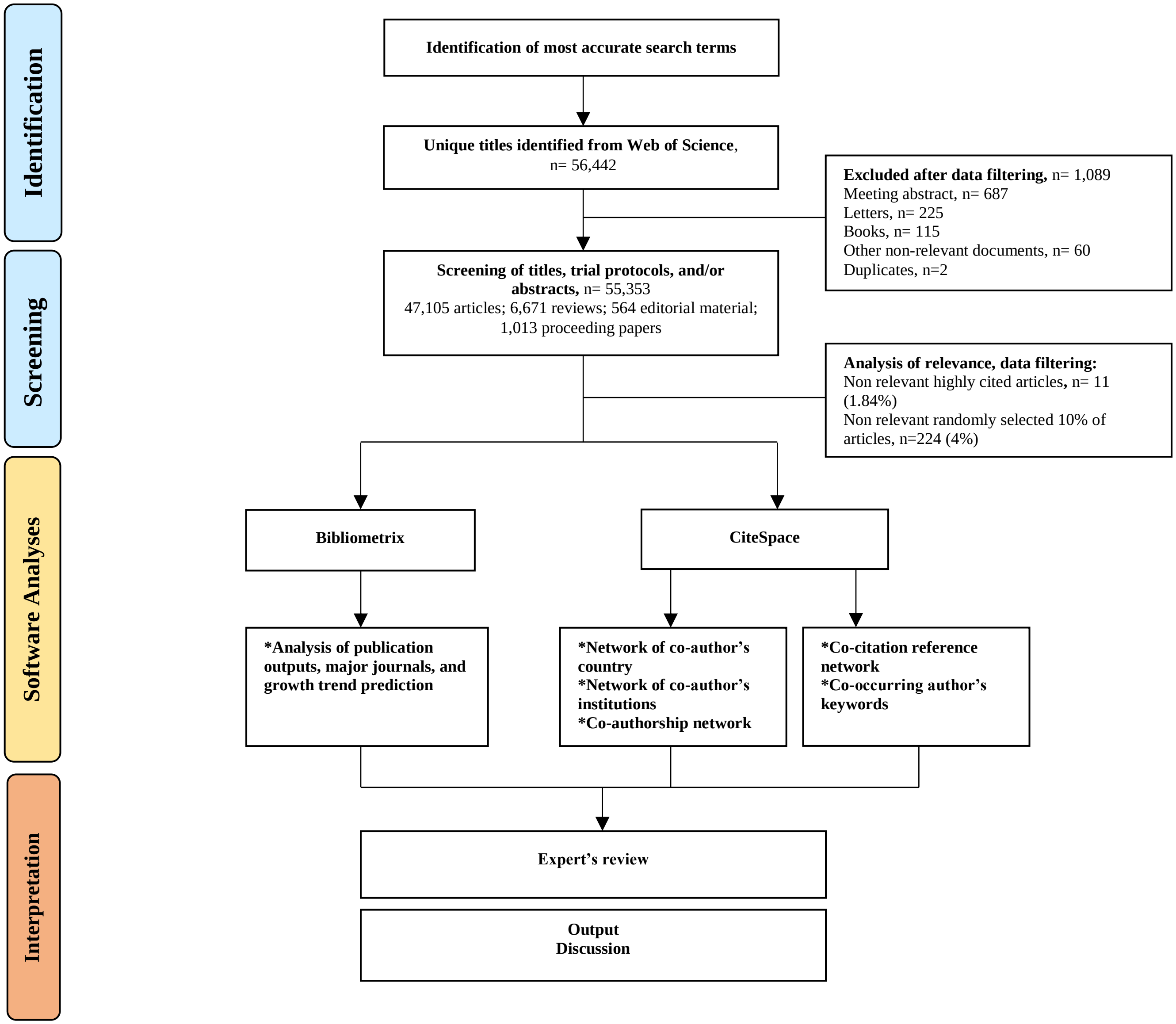

Supplementary Figure 2. Annual scientific production for the 1981-2021 time period

1989: 17; 1991: 334; 2001: 581; 2007: 959; 2011: 1916; 2016: 3226; 2020: 5291; 2021: 5604- Mean annual growth rate of 6.8%

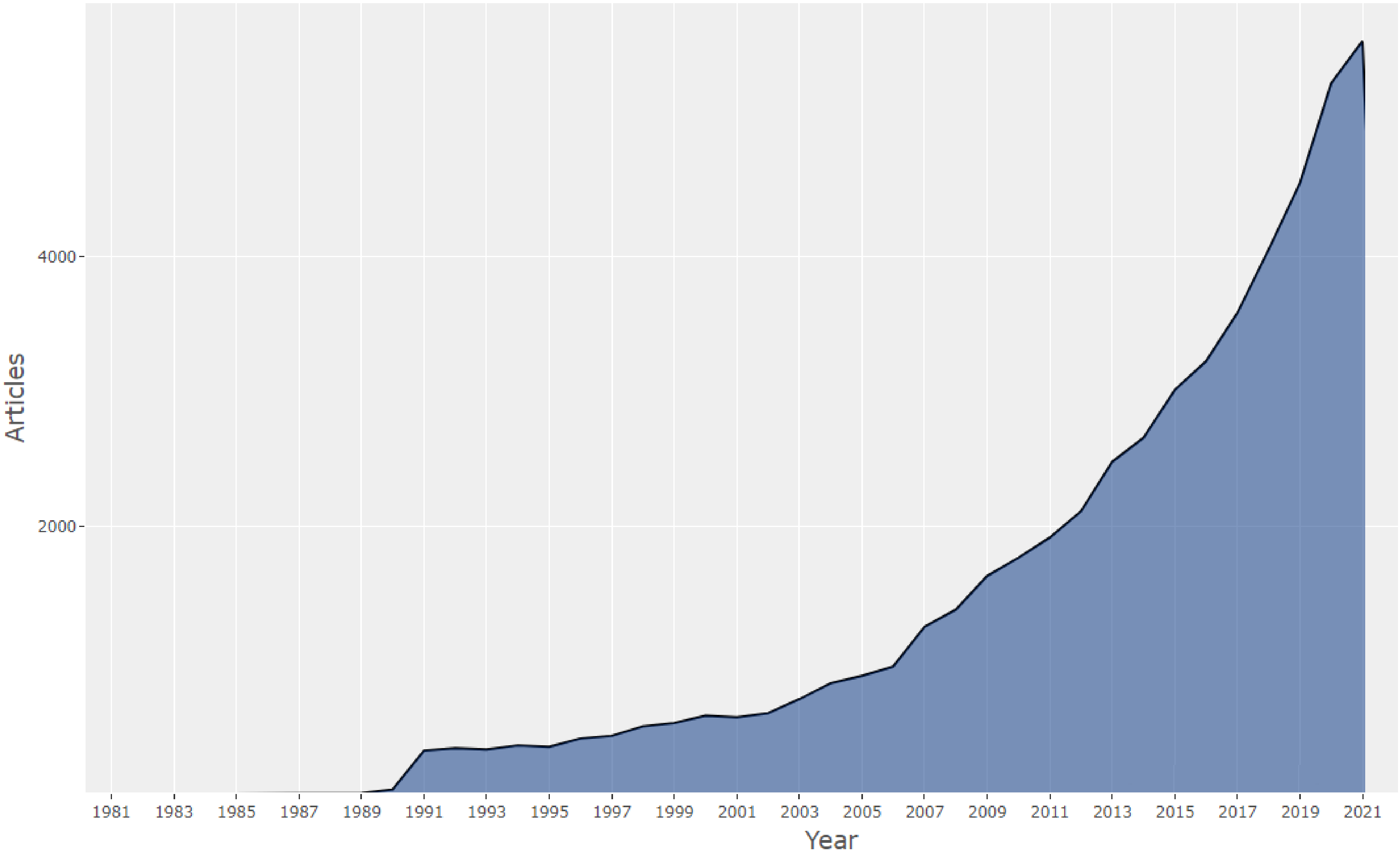

Supplementary Figure 3. Average article citations per year for the 1967-2021 time period

Average citation per year: 1989: 1.8; 1991: 3.8; 2001: 3.6; 2007: 4.5; 2011: 4.3; 2016: 4.3; 2020: 2.9- Mean annual growth rate of 6.8%

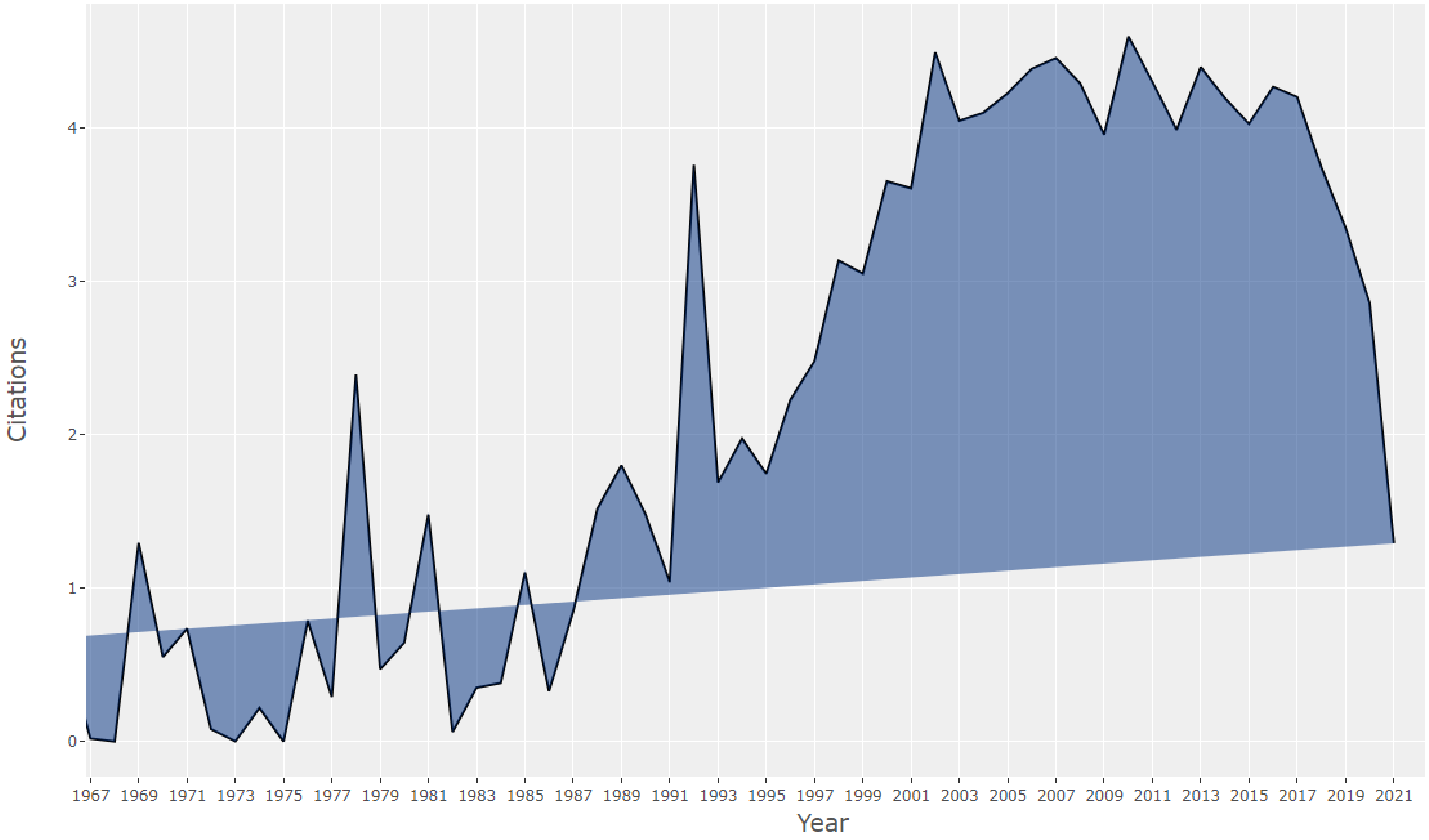

Supplementary Figure 4. Detail of the reference co-citation network clusters (time map) and clusters' details for the 1988-2021 (A), reference co-citation network clusters (burstness, clusters, time map), and clusters' details for the 2016-2021 time period (B) and reference co-citation network clusters (burstness, clusters, time map), and clusters' details for 2021 time period (C)

A. Detail of reference co-citation network clusters (1988-2021)

Co-citation reference network with highlight of corresponding clusters (1988-2021)

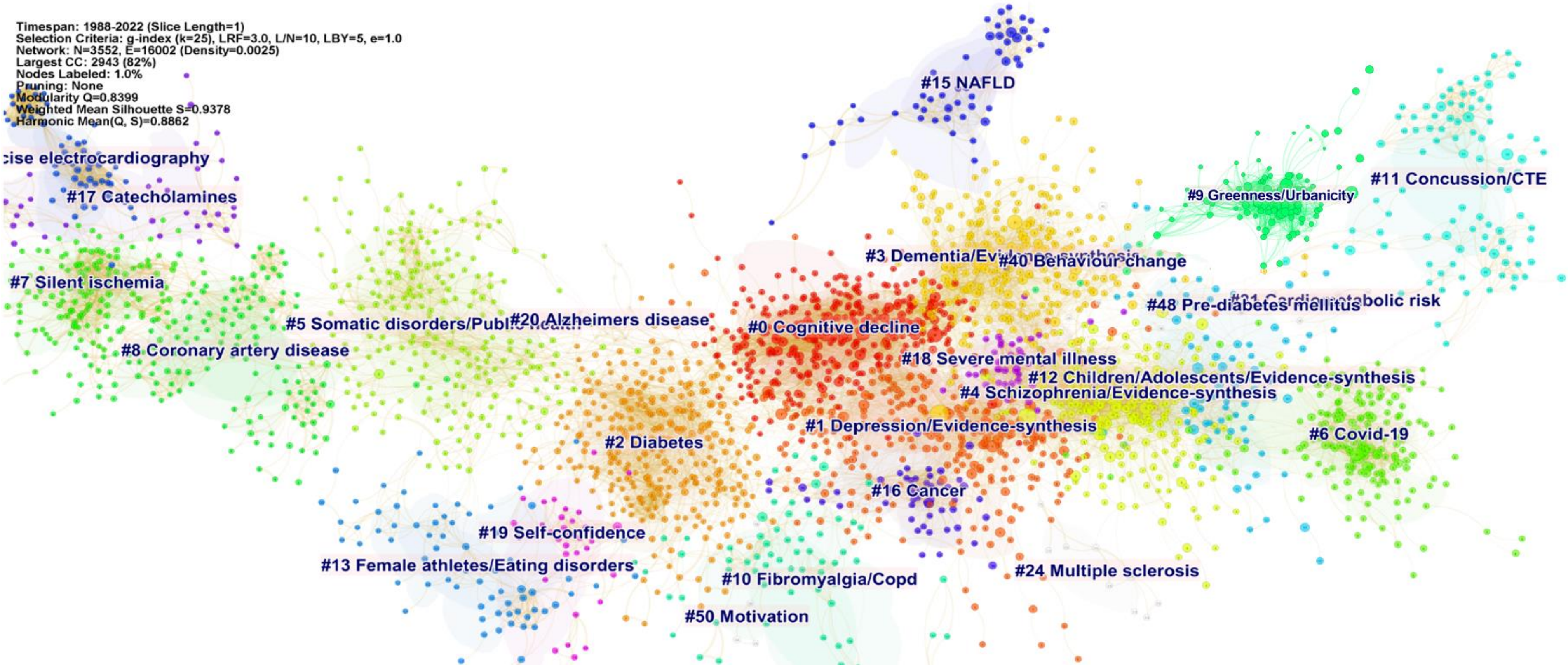

Reference co-citation network time map (1988-2021)

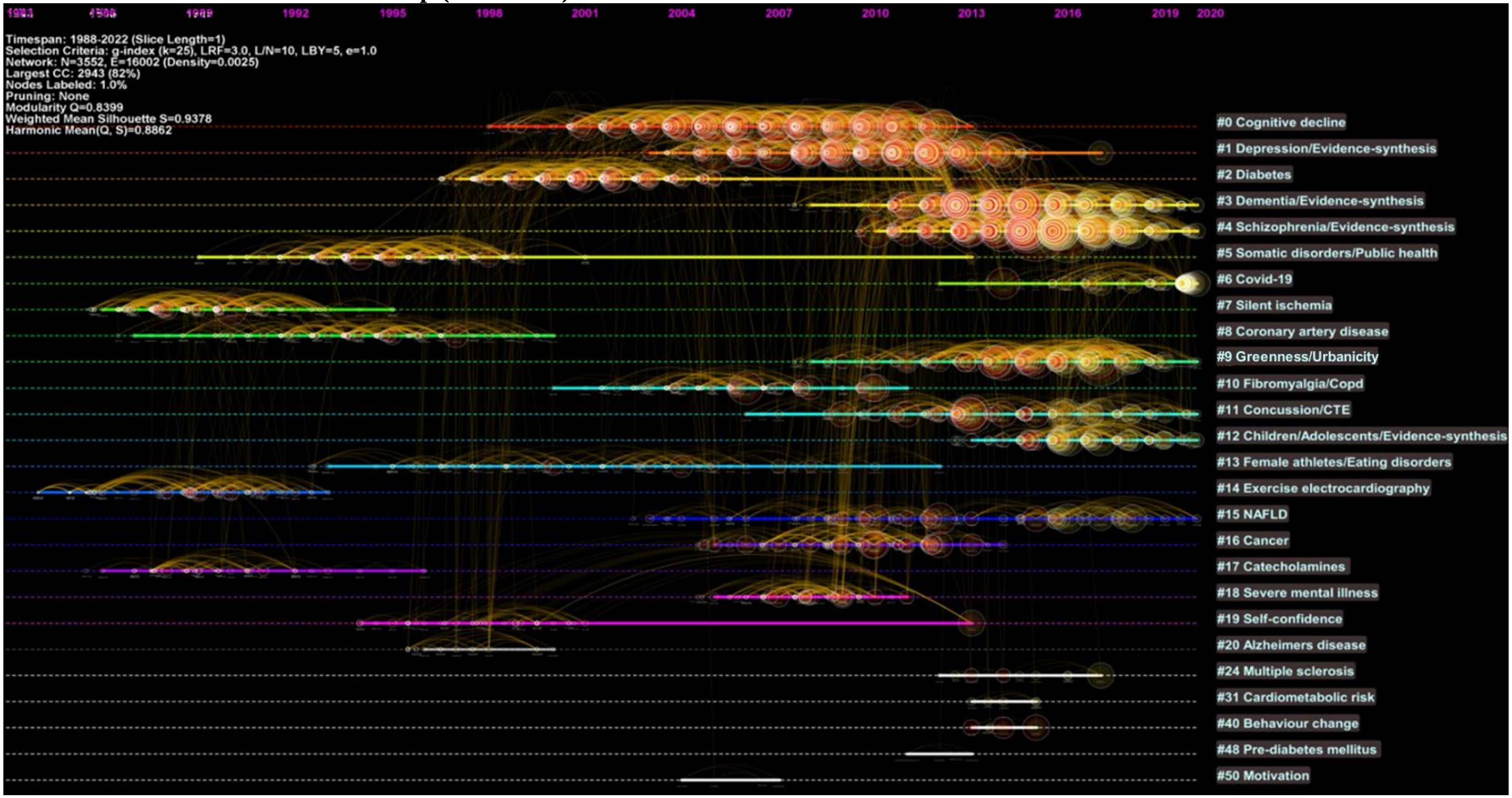

Cluster	Size	Silhouette score	Mean year	Top extracted terms based on titles	Top extracted terms based on keywords (Log-likelihood ratio algorithm; p-level)
0	324	0.923	2006	alzheimers disease; mental decline; temporal self-regulation theory; spontaneous seizures; aerobic fitness | physical activity; vascular dementia; risk factor; mental decline; temporal self-regulation theory	dementia (182.59, 1.0E-4); alzheimers disease (146.33, 1.0E-4); cognition (98.33, 1.0E-4); aging (62.85, 1.0E-4); mental health (58.86, 1.0E-4)

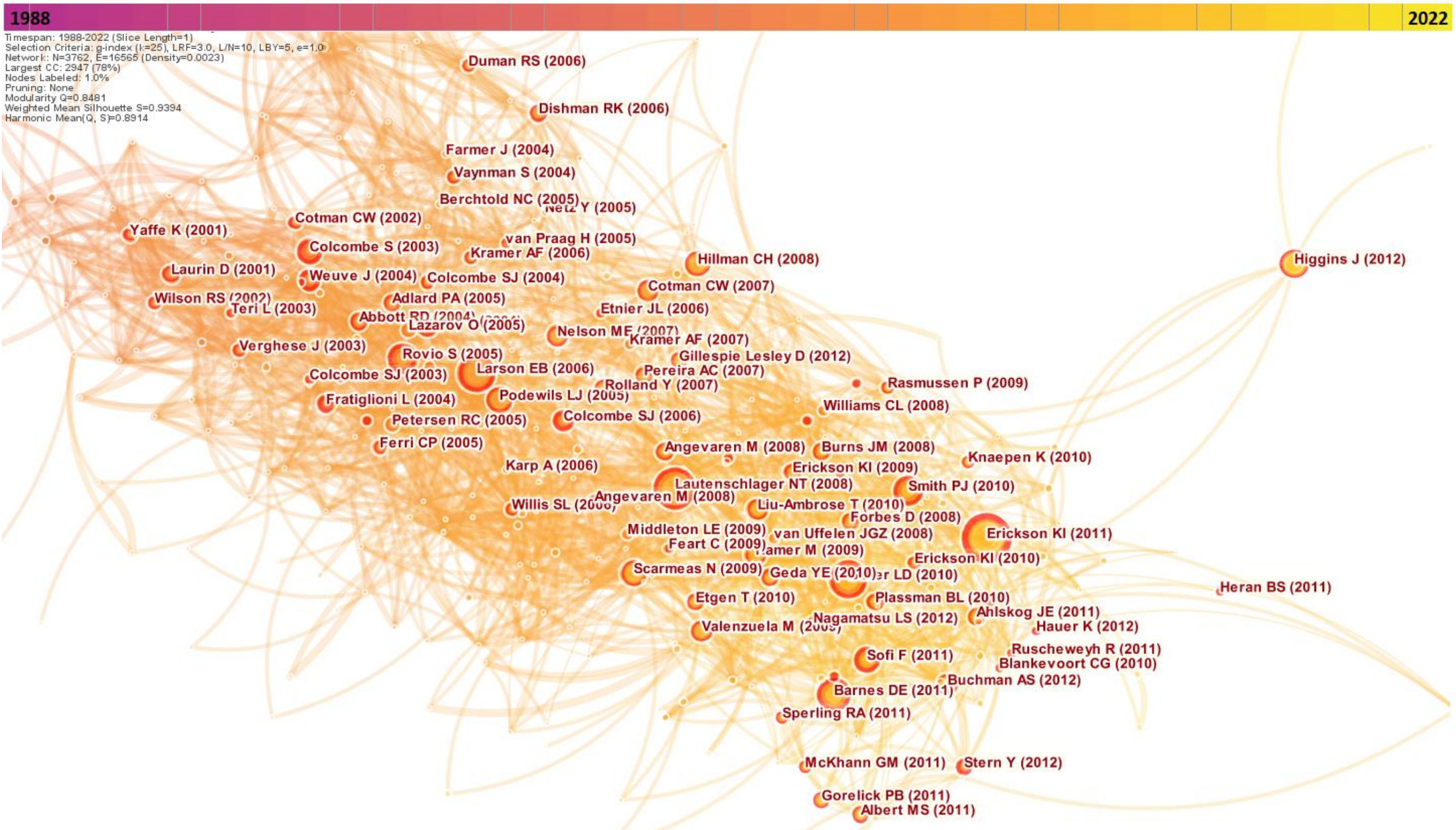

Papers with highest centrality:

Colcombe 2003- Fitness effects on the cognitive function of older adults: a meta-analytic study;
Weuve 2004- Physical activity, including walking, and cognitive function in older women
Rovio 2005- Leisure-time physical activity at midlife and the risk of dementia and Alzheimer's disease
Erickson 2011- Exercise training increases size of hippocampus and improves memory
Larson 2006- Exercise is associated with reduced risk for incident dementia among persons 65 years of age and older
Hillman 2008- Be smart, exercise your heart: exercise effects on brain and cognition
Angevaren 2008- Physical activity and enhanced fitness to improve cognitive function in older people without known cognitive impairment
Lautenschlager 2008- Effect of physical activity on cognitive function in older adults at risk for Alzheimer disease: a randomized trial
Liu-Ambrose 2010- Resistance training and executive functions: a 12-month randomized controlled trialErickson 2011- Exercise training increases size of hippocampus and improves memory

**Cluster 1- Physical exercise and 'depression'**					
1	292	0.823	2009	physical activity; mental health; sedentary behavior; life satisfaction; physical education | exercise; depression; neurogenesis; angiogenesis; vegf	depression (221.67, 1.0E-4); exercise (84.08, 1.0E-4); covid-19 (68.19, 1.0E-4); dementia (66.47, 1.0E-4); anxiety (45.52, 1.0E-4)

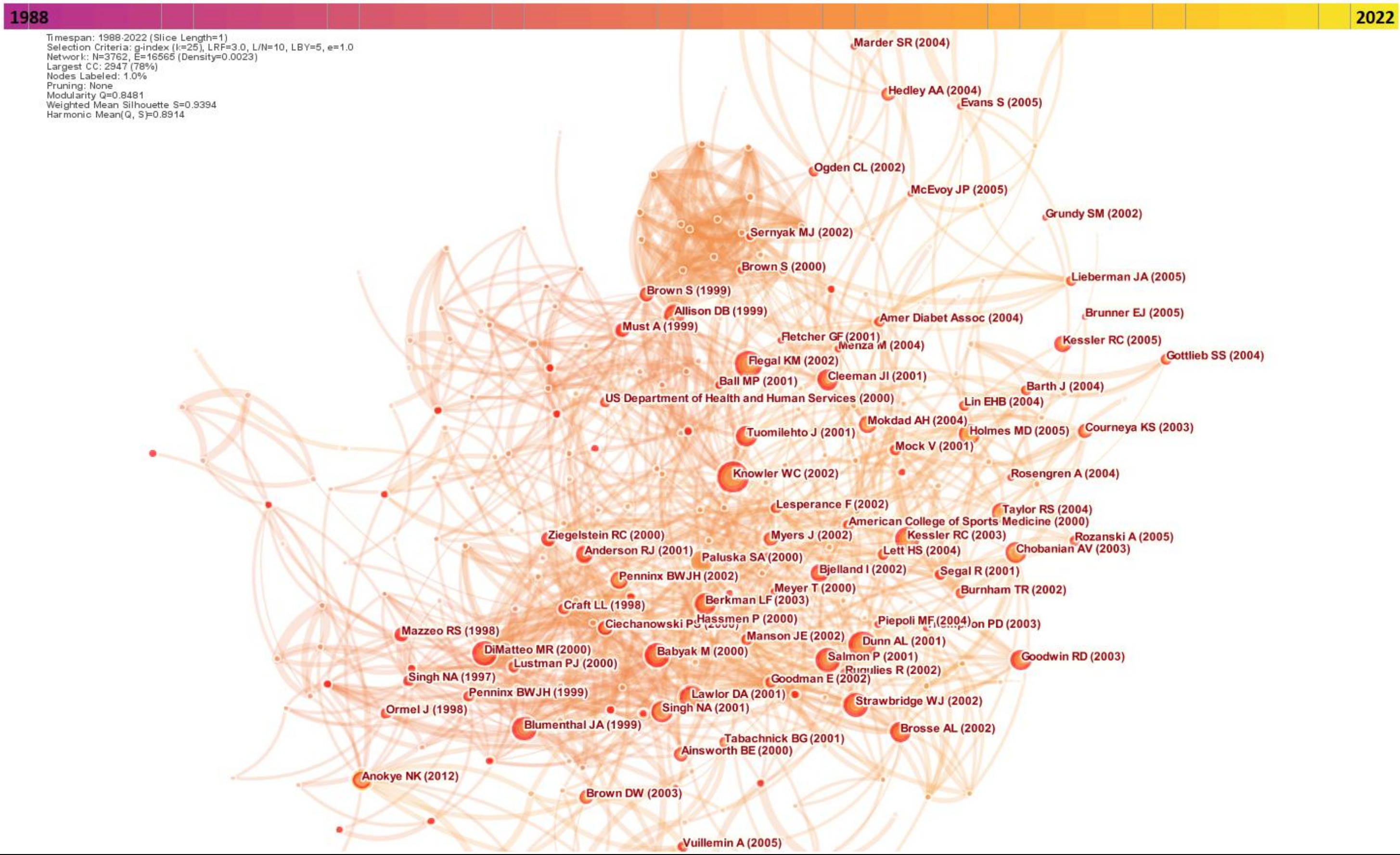

Papers with highest centrality:
Anokye 2012- Physical activity and health related quality of life
DiMatteo 2000- Depression Is a Risk Factor for Noncompliance With Medical Treatment Meta-analysis of the Effects of Anxiety and Depression on Patient Adherence
Blumenthal 1999- Effects of exercise training on older patients with major depression
Babyak 2000- Exercise treatment for major depression: maintenance of therapeutic benefit at 10 months
Lawlor 2001- The effectiveness of exercise as an intervention in the management of depression: systematic review and meta-regression analysis of randomised controlled trials
Salmon 2001- Effects of physical exercise on anxiety, depression, and sensitivity to stress: a unifying theory
Strawbridege 2002- Physical activity reduces the risk of subsequent depression for older adults
Knowler 2002- Reduction in the incidence of type 2 diabetes with lifestyle intervention or metformin
Chobanian 2003- Seventh report of the Joint National Committee on Prevention, Detection, Evaluation, and Treatment of High Blood Pressure
Holmes 2005- Physical activity and survival after breast cancer diagnosis

Cluster 2- Physical exercise and 'diabetes'

2	289	0.918	2001	physical activity; self-reported physical activity; antidepressive agents; treatment of depression; metabolic control | mental health; psychological distress; self-reported physical activity; antidepressive agents; treatment of depression	diabetes (46.63, 1.0E-4); weight gain (33.39, 1.0E-4); atypical antipsychotics (31.29, 1.0E-4); cognition (31.27, 1.0E-4); obesity (29.9, 1.0E-4)

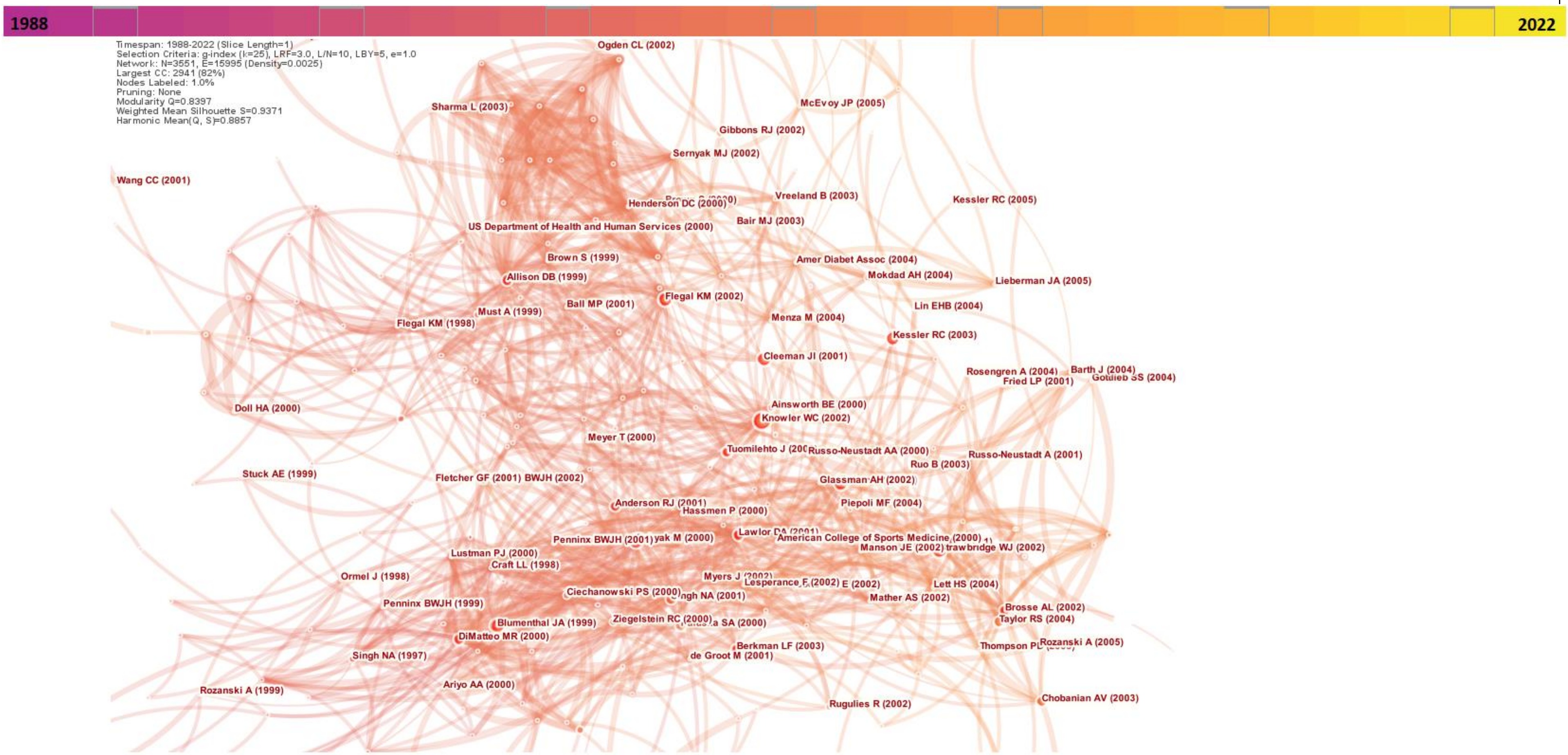

Papers with highest centrality:
Knowler 2002- Reduction in the Incidence of Type 2 Diabetes with Lifestyle Intervention or Metformin.
Flegal 2002- Prevalence and Trends in Obesity Among US Adults, 1999-2000
Allison 1999- Prognostic significance of exercise-induced systemic hypertension in healthy subjects
Kessler 2003- The epidemiology of major depressive disorder: results from the National Comorbidity Survey Replication (NCS-R)
Anderson 2001- The Prevalence of Comorbid Depression in Adults With Diabetes: A meta-analysis

Cluster 3- Physical exercise and 'dementia'

3	269	0.916	2014	physical activity; cognitive decline; mediterranean diet; prospective cohort studies; body mass index | alzheimers disease; cognitive function; controlled trial; isotemporal substitution model; white matter integrity	dementia (418.34, 1.0E-4); mild cognitive impairment (215.34, 1.0E-4); cognition (176.45, 1.0E-4); alzheimers disease (176.41, 1.0E-4); mental health (99.52, 1.0E-4)

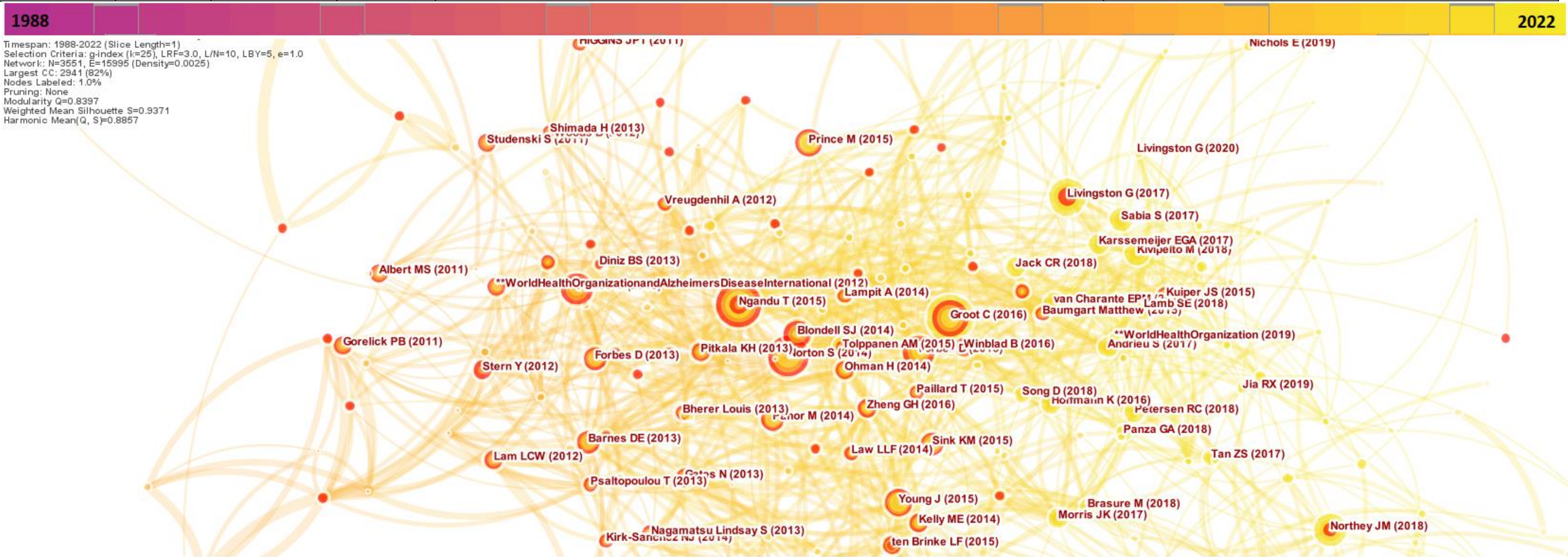

Papers with highest centrality:

Groot C 2016- The effect of physical activity on cognitive function in patients with dementia: A meta-analysis of randomized control trials

Ngandu T 2015- A 2 year multidomain intervention of diet, exercise, cognitive training, and vascular risk monitoring versus control to prevent cognitive decline in at-risk elderly people (FINGER): a randomised controlled trial

Norton 2014- Potential for primary prevention of Alzheimer's disease: an analysis of population-based data

Livingston 2017- Dementia prevention, intervention, and care

Kelly 2014- The impact of cognitive training and mental stimulation on cognitive and everyday functioning of healthy older adults: A systematic review and meta-analysis

Northey 2018- Exercise interventions for cognitive function in adults older than 50: a systematic review with meta-analysis

Blondell 2014- Does physical activity prevent cognitive decline and dementia?: A systematic review and meta-analysis of longitudinal studies

Cluster 4- Physical exercise and 'schizophrenia'

4	267	0.912	2015	physical activity; sedentary time; university students; perceived stress; independent associations | mental health; exercise psychology; television viewing; psychological outcomes; health trajectory	schizophrenia (352.9, 1.0E-4); psychosis (205.31, 1.0E-4); physical activity (128.08, 1.0E-4); dementia (101.37, 1.0E-4); alzheimers disease (82.15, 1.0E-4)

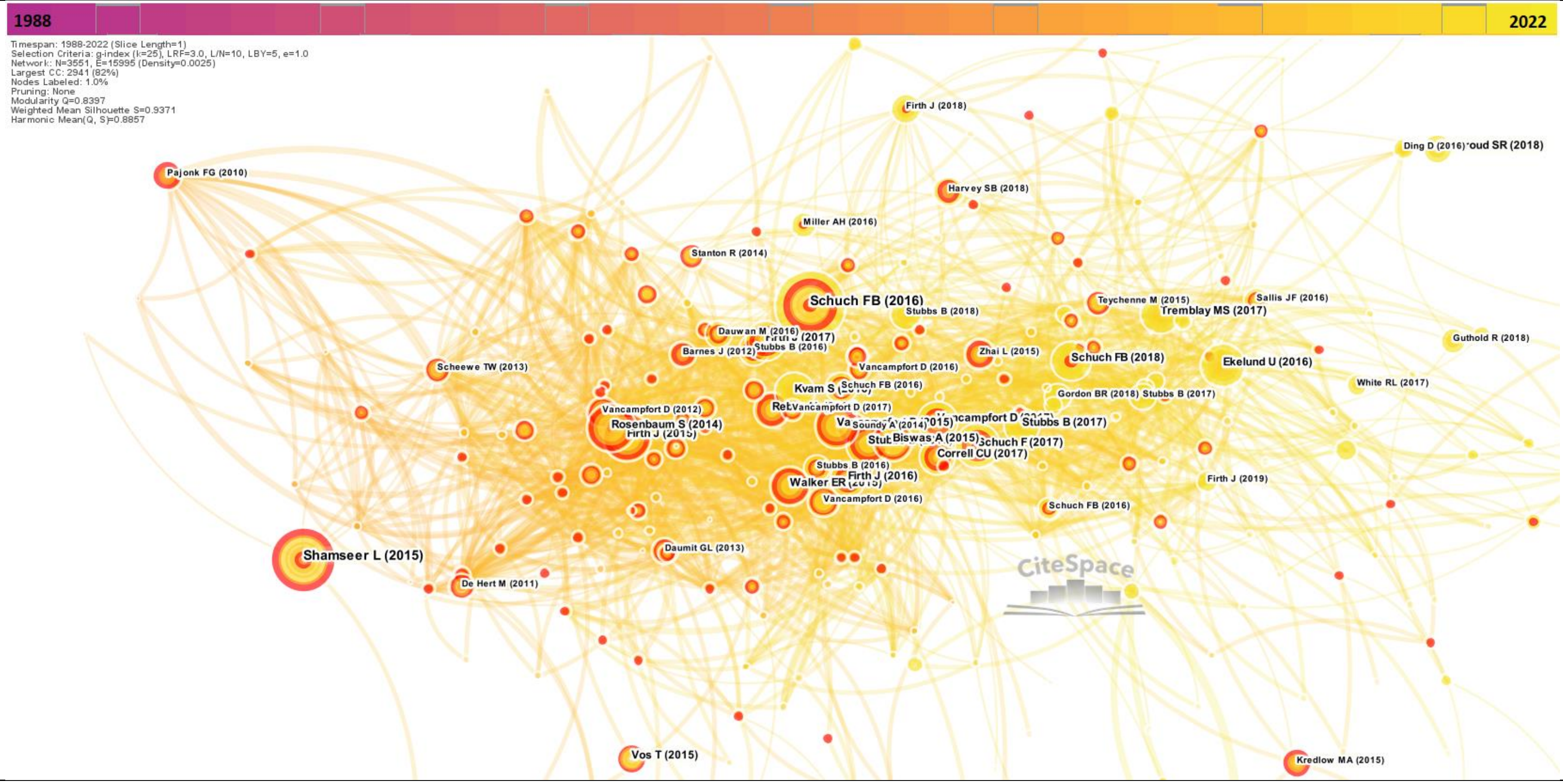

Papers with highest centrality:

Pajonk 2010- Hippocampal plasticity in response to exercise in schizophrenia

Shamseer 2015- Preferred reporting items for systematic review and meta-analysis protocols (PRISMA-P) 2015: elaboration and explanation

Vancampfort 2012- A systematic review of correlates of physical activity in patients with schizophrenia

Vancampfort 2015- Exercise improves cardiorespiratory fitness in people with schizophrenia: A systematic review and meta-analysis

Rosembaum 2014- Physical activity interventions for people with mental illness: a systematic review and meta-analysis

Firth 2015- A systematic review and meta-analysis of exercise interventions in schizophrenia

Firth 2017- The pro-cognitive mechanisms of physical exercise in people with schizophrenia

Schuch 2016- Exercise as a treatment for depression: A meta-analysis adjusting for publication bias

Schuch 2017- Physical activity and sedentary behavior in people with major depressive disorder: A systematic review and meta-analysis

Vancampfort 2017- Physical activity is associated with the physical, psychological, social and environmental quality of life in people with mental health problems in a low resource setting

Stubbs 2017- An examination of the anxiolytic effects of exercise for people with anxiety and stress-related disorders: A meta-analysis

Trewmblay 2017- Sedentary Behavior Research Network (SBRN) – Terminology Consensus Project process and outcome

Cluster 5- Physical exercise and 'somatic disorders and public health'

5	238	0.953	1995	risk factors; gait problems; coronary artery disease; affect positive; depressed mood | systemic lupus erythematosus; renal dialysis; renal failure; lupus nephritis; coronary artery disease	systemic lupus erythematosus (35.68, 1.0E-4); physical activity (30.67, 1.0E-4); morbidity (29.7, 1.0E-4); chronic fatigue syndrome (28.86, 1.0E-4); disability (24.14, 1.0E-4)

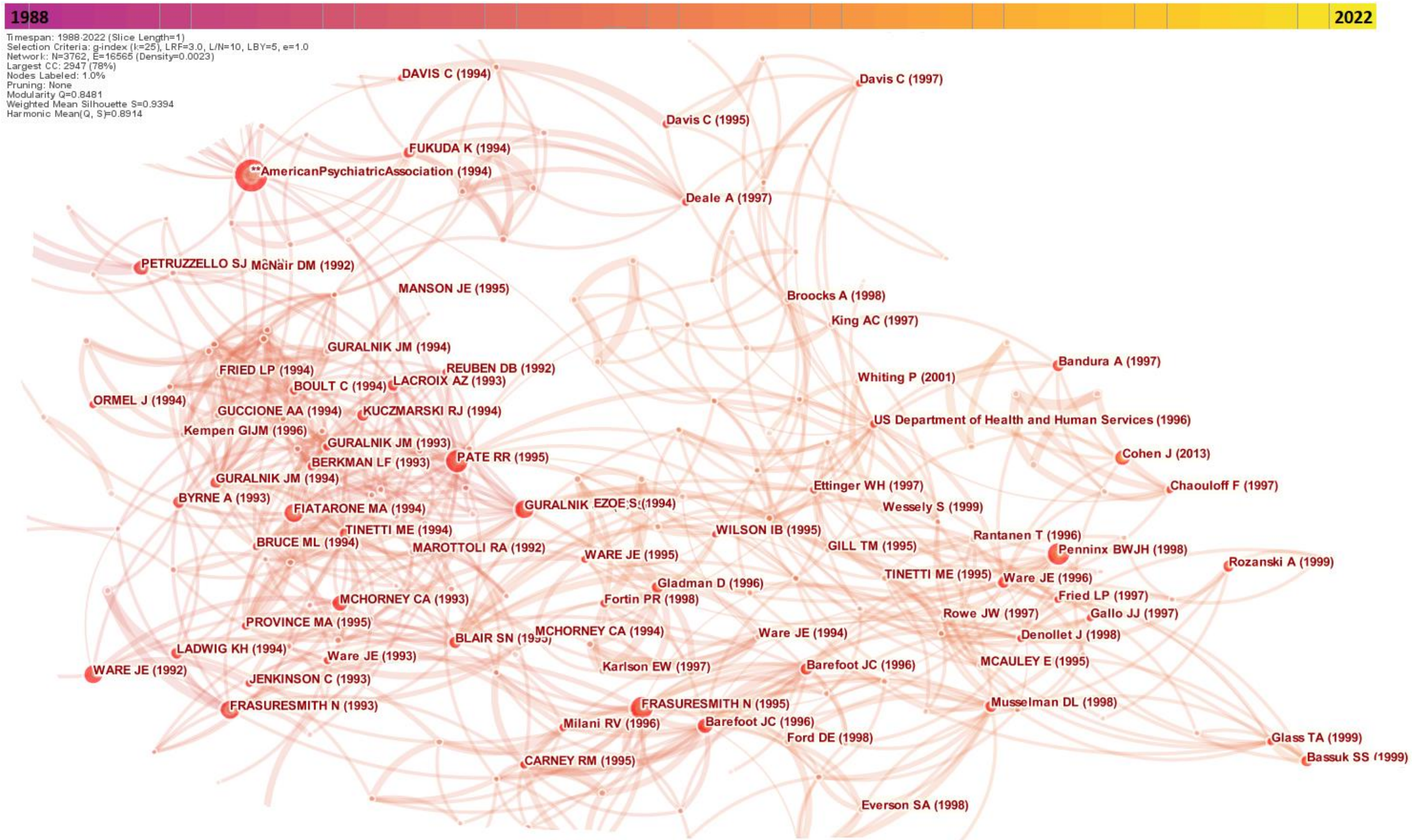

Papers with highest centrality:
Ware 1992- The MOS 36-Item Short-Form Health Survey (SF-36): Conceptual Framework and Item Selection
Mchorney 1993- The MOS 36-Item Short-Form Health Survey (SF-36): II. Psychometric and clinical tests of validity in measuring physical and mental health constructs
Tinetti 1994- A multifactorial intervention to reduce the risk of falling among elderly people living in the community
Fiatarone 1994- Exercise training and nutritional supplementation for physical frailty in very elderly people
Pate 1995- Physical activity and public health. A recommendation from the Centers for Disease Control and Prevention and the American College of Sports Medicine
Guralnik 1994- A short physical performance battery assessing lower extremity function: association with self-reported disability and prediction of mortality and nursing home admission
Frasuresmith 1995- The impact of negative emotions on prognosis following myocardial infarction: Is it more than depression?
Penninx 1998- Depressive Symptoms and Physical Decline in Community-Dwelling Older Persons
Cohen 2013- Physical activity in parks: A randomized controlled trial using community engagement

Cluster 6- Physical exercise and 'covid-19'

6	191	0.979	2019	mental health; university students; suicidal behavior; psychological intervention; physical disabilities | physical activity; outpatient care; health behaviour; ambulatory care; information sufficiency	covid-19 (920.2, 1.0E-4); pandemic (208.38, 1.0E-4); lockdown (153.85, 1.0E-4); coronavirus (127.27, 1.0E-4); quarantine (74.13, 1.0E-4)

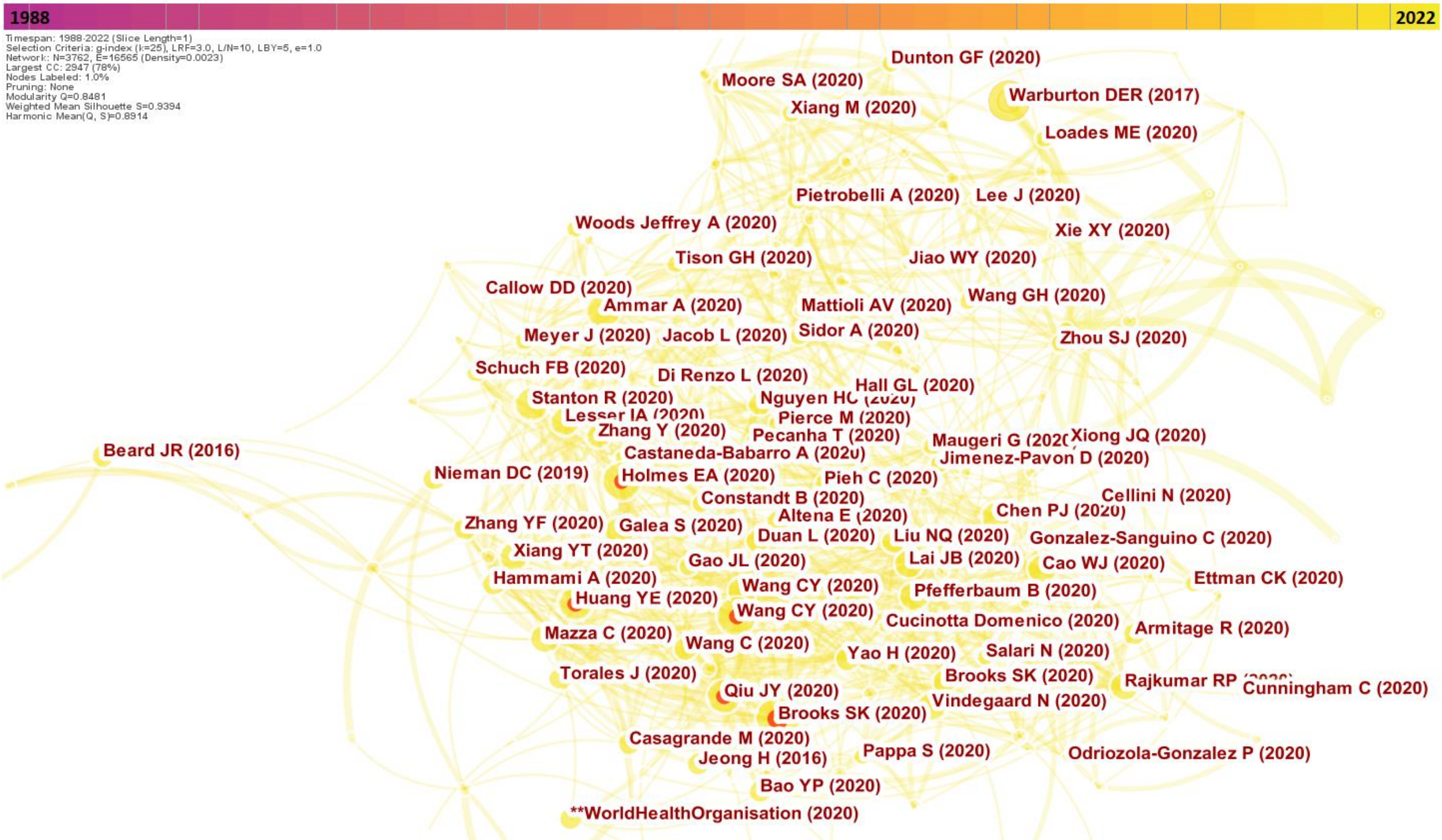

Papers with highest centrality:

Warburton 2017- Health benefits of physical activity: a systematic review of current systematic reviews
Xiang 2020- Relationship of Physical Activity With Anxiety and Depression Symptoms in Chinese College Students During the COVID-19 Outbreak
Ammar 2020- Effects of COVID-19 Home Confinement on Eating Behaviour and Physical Activity: Results of the ECLB-COVID19 International Online Survey
Stanton 2020- Depression, Anxiety and Stress during COVID-19: Associations with Changes in Physical Activity, Sleep, Tobacco and Alcohol Use in Australian Adults
Holmes 2020- Validity of Smartphone Heart Rate Variability Pre- and Post-Resistance Exercise
Wang 2020- A Preventive Role of Exercise Across the Coronavirus 2 (SARS-CoV-2) Pandemic
Qi 2020- Physical Activity, Health-Related Quality of Life, and Stress among the Chinese Adult Population during the COVID-19 Pandemic
Brooks 2020- Social distancing and COVID-19: an unprecedented active transport public health opportunity
Huang 2020- Effects of Exercise on Depression, Anxiety, Cognitive Control, Craving, Physical Fitness and Quality of Life in Methamphetamine-Dependent Patients

Cluster 7- Physical exercise and 'silent ischemia'

7	145	0.964	1989	silent ischemia; stable angina; calcium antagonists; coronary heart; angina pectoris | myocardial infarction; ventricular function; ambulatory electrocardiographic monitoring; exercise testing; st segment depression	silent ischemia (56.12, 1.0E-4); prognosis (44.6, 1.0E-4); ambulatory electrocardiographic monitoring (33.59, 1.0E-4); diltiazem (33.59, 1.0E-4); myocardial ischemia (26.87, 1.0E-4)

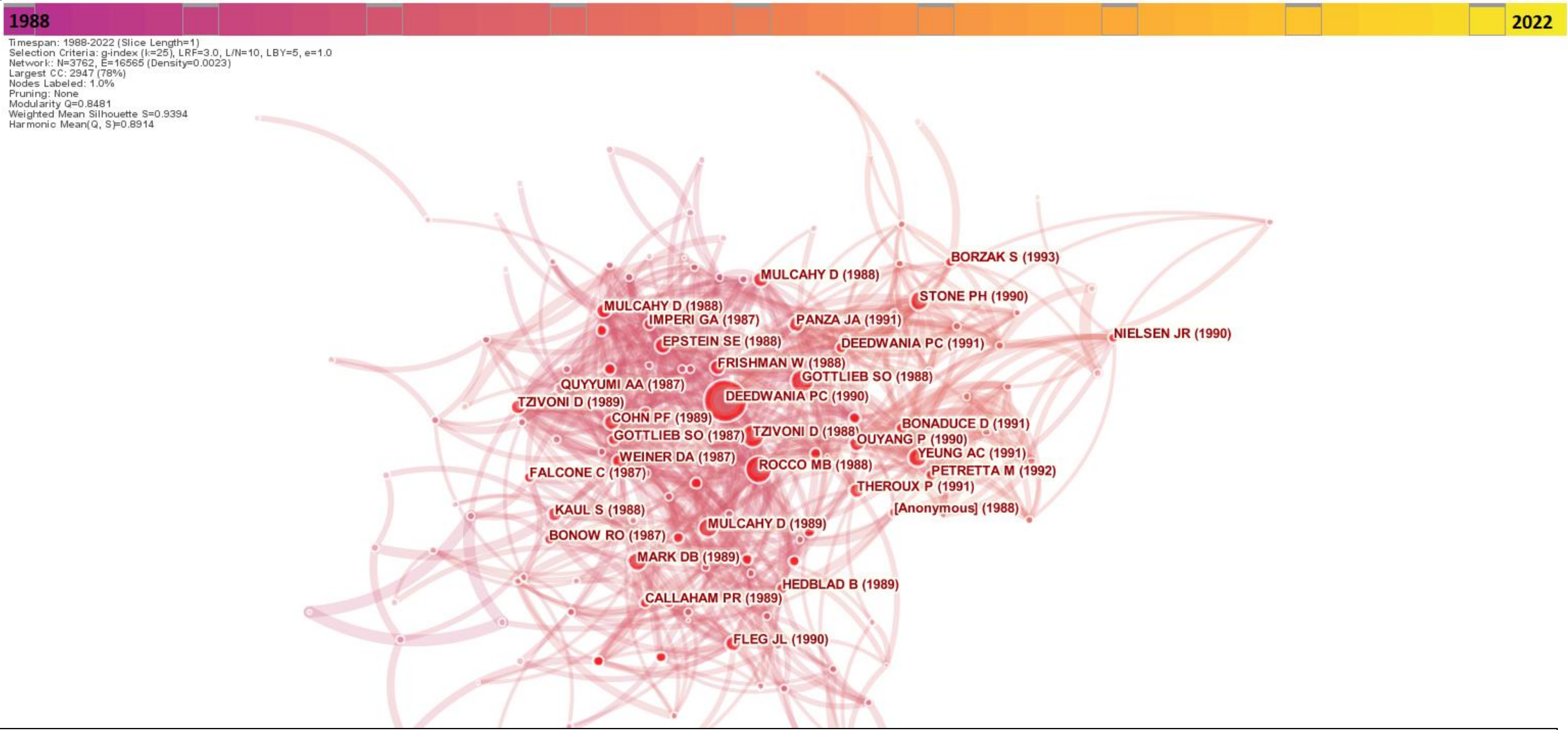

Papers with highest centrality:
Stone 1990- Muscle conditioning and muscle injuries
Mulcahy 1988- The long-term work record of post-infarction patients subjected to an informal rehabilitation and secondary prevention programme
Petretta 1992- Characterization and prognostic significance of silent myocardial ischemia on predischarge electrocardiographic monitoring in unselected patients with myocardial infarction
Epstein 1988- PERSONALITY, EXERCISE AND PSYCHOLOGICAL WELL-BEING: STATIC RELATIONSHIPS IN THE COMMUNITY
Deedwania 1990- Silent ischemia during daily life is an independent predictor of mortality in stable angina.
Weiner 1987- Value of exercise testing in determining the risk classification and the response to coronary artery bypass grafting in three-vessel coronary artery disease: a report from the Coronary Artery Surgery Study (CASS) registry

Cluster 8- Physical exercise and ‘coronary artery disease’

8	141	0.962	1994	coronary artery disease; prediction equations; qtc dispersion; beta-adrenergic blockers; myocardial infarction | coronary disease; exercise testing; coronary artery disease; qtc dispersion; beta-adrenergic blockers	coronary artery disease (92.04, 1.0E-4); dobutamine (47.2, 1.0E-4); coronary disease (43.29, 1.0E-4); amlodipine (41.82, 1.0E-4); angina pectoris (38.87, 1.0E-4)

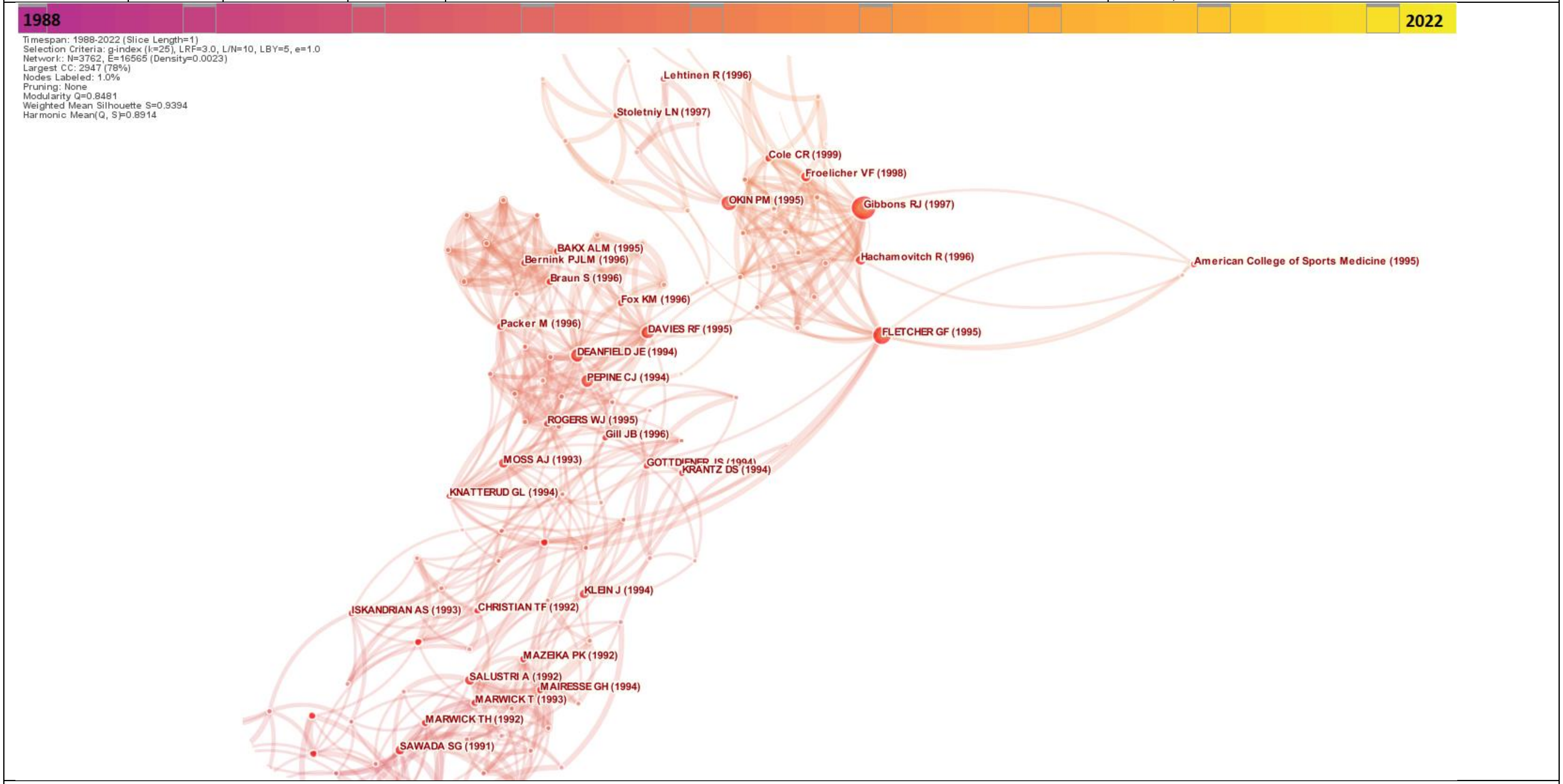

Papers with highest centrality:
Sawanda 1991- Echocardiographic Detection of Coronary Artery Disease During Dobutamine Infusion
Pepine 1994- Effects of Treatment on Outcome in Mildly Symptomatic Patients With Ischemia During Daily Life
The Atenolol Silent Ischemia Study (ASIST)
Deanfield 1994- Amlodipine reduces transient myocardial ischemia in patients with coronary artery disease: double-blind Circadian Anti-Ischemia Program in Europe (CAPE Trial)
Bakx 1995- Effects of the new calcium antagonist mibefradil (Ro 40-5967) on exercise duration in patients with chronic stable angina pectoris: A multicenter, placebo-controlled study
Okin 1995- Heart Rate Adjustment of ST Segment Depression and Performance of the Exercise Electrocardiogram: A Critical Evaluation
Gibbons 1997- ACC/AHA Guidelines for Exercise Testing. A report of the American College of Cardiology/American Heart Association Task Force on Practice Guidelines (Committee on Exercise Testing)
Fletcher 1995- Exercise Standards. A Statement for Healthcare Professionals From the American Heart Association

Cluster 9- Physical exercise and ‘greenness/urbanicity’

9	108	0.998	2015	green space; mental health; structural equation model; urban sustainability; exposure assessment | physical activity; ecosystem services; social support; air quality; general health	green space (311.19, 1.0E-4); greenspace (121.24, 1.0E-4); nature (104.58, 1.0E-4); greenness (98.41, 1.0E-4); exercise (91.71, 1.0E-4)

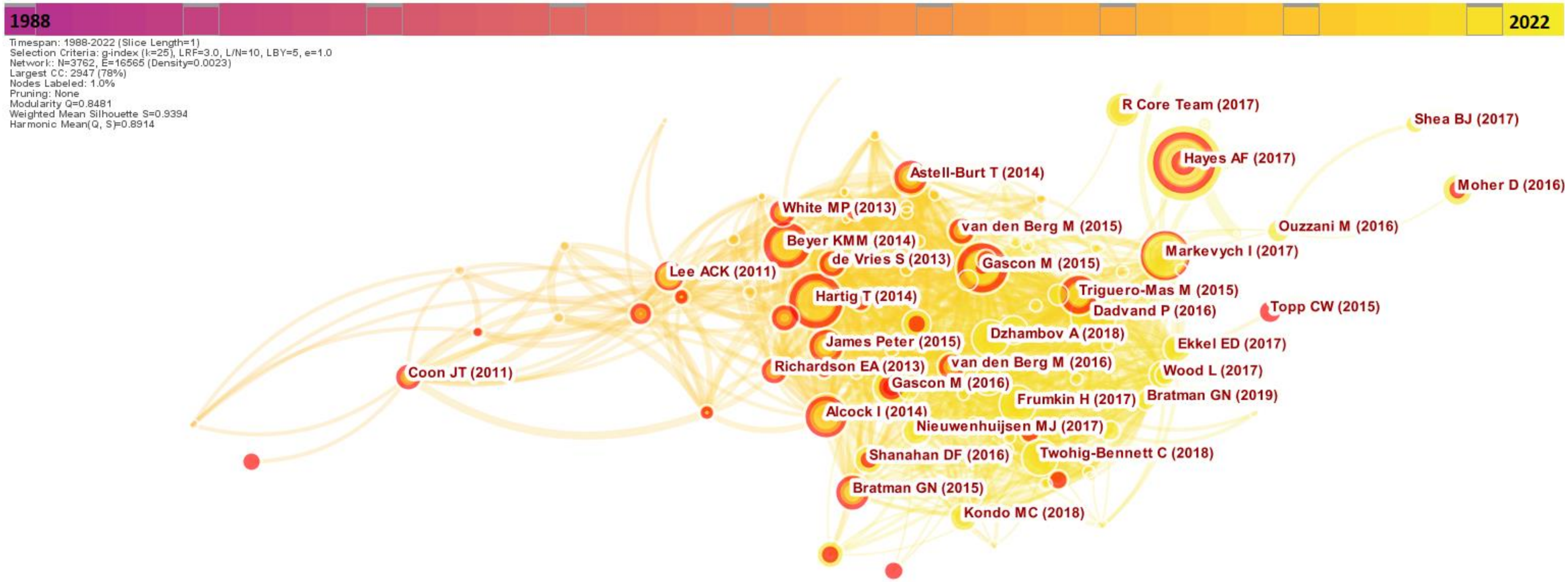

Papers with highest centrality:
Beyer 2014- Exposure to neighborhood green space and mental health: evidence from the survey of the health of Wisconsin
Hartig 2014- Nature and health
Alcock 2014- Longitudinal Effects on Mental Health of Moving to Greener and Less Green Urban Areas
Bratman 2015- Nature experience reduces rumination and subgenual prefrontal cortex activation
Gascon 2015- Residential green spaces and mortality: A systematic review
Astell-burt 2014- Do low-income neighbourhoods have the least green space? A cross-sectional study of Australia's most populous cities
Markevych 2017- Exploring pathways linking greenspace to health: Theoretical and methodological guidance
Hayes 2017- Introduction to Mediation, Moderation, and Conditional Process Analysis Second Edition A Regression-Based Approach
Dzhambov 2018- Multiple pathways link urban green- and bluespace to mental health in young adults

Cluster 10- Physical exercise and 'fibromyalgia/copd'

10	97	0.981	2005	physical activity; health-related quality; pilates exercises; eating disorders; sedentary time | chronic obstructive pulmonary disease; pilates exercises; eating disorders; sedentary time; middle-aged women	fibromyalgia (200.02, 1.0E-4); copd (63.56, 1.0E-4); chronic obstructive pulmonary disease (56.49, 1.0E-4); obesity (42.43, 1.0E-4); pulmonary rehabilitation (28.21, 1.0E-4)

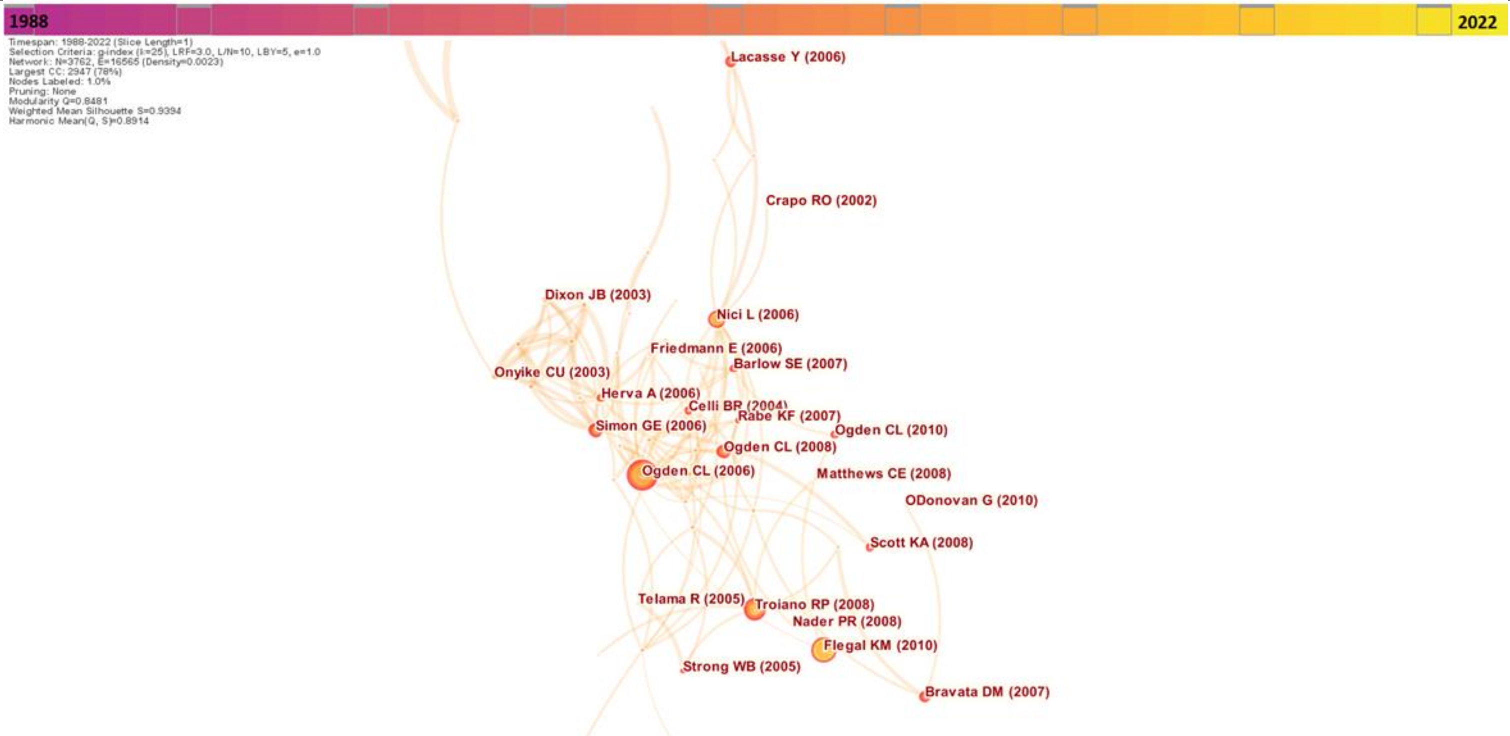

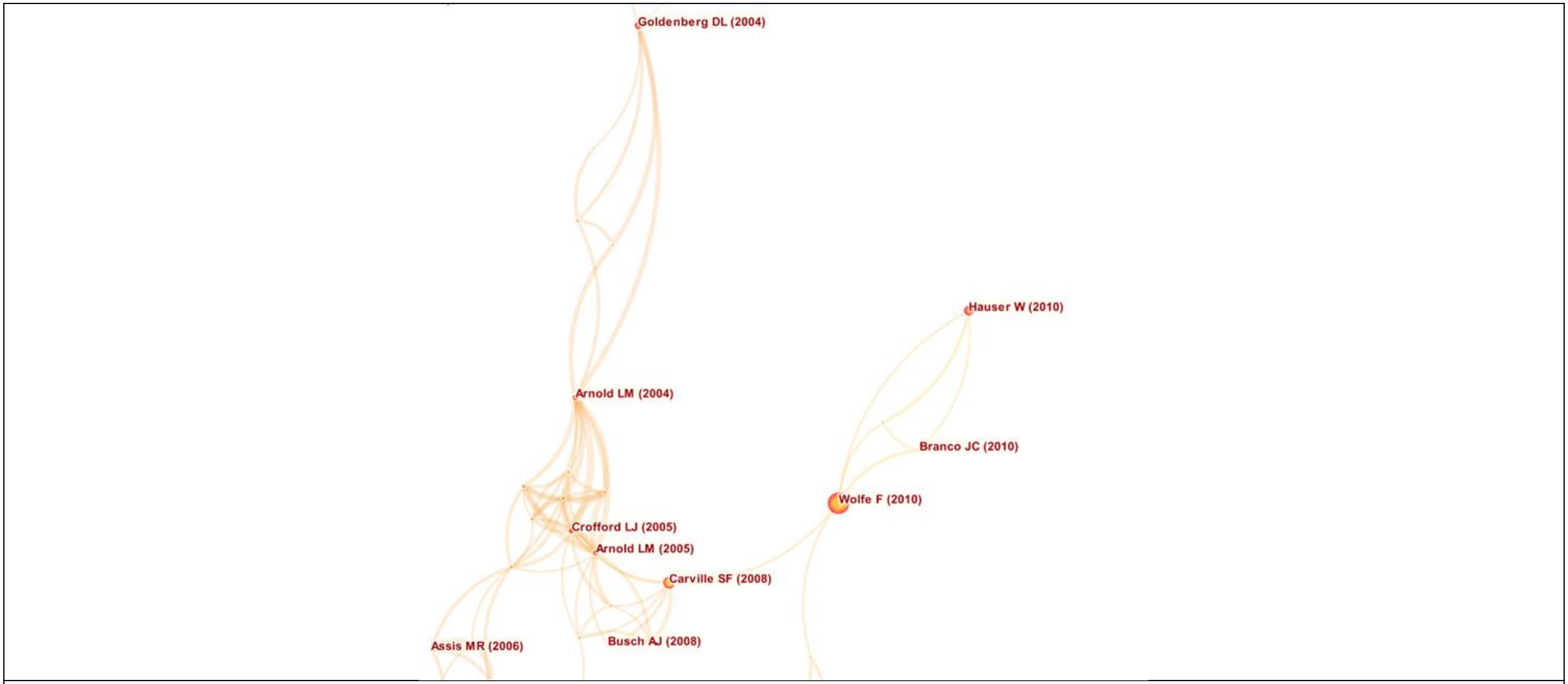

Papers with highest centrality:
Troiano 2008- Physical activity in the United States measured by accelerometer
Nici 2006- American Thoracic Society/European Respiratory Society Statement on Pulmonary Rehabilitation
Simon 2006- Association Between Obesity and Psychiatric Disorders in the US Adult Population
Ogden 2006- Obesity Among Adults in the United States: No Change Since 2003-2004
Flegal 2010- Prevalence and Trends in Obesity Among US Adults, 1999-2008
Goldenberg 2004- Management of fibromyalgia syndrome
Wolfe 2010- The development of fibromyalgia – I: Examination of rates and predictors in patients with rheumatoid arthritis (RA)
Carville 2008- EULAR evidence-based recommendations for the management of fibromyalgia syndrome
Arnold lm 2004- A Double-Blind, Multicenter Trial Comparing Duloxetine With Placebo in the Treatment of Fibromyalgia Patients With or Without Major Depressive Disorder

Cluster 11- Physical exercise and ‘concussion/chronic traumatic encephalopathy’

11	95	0.996	2014	mental health; mood disorders; anxiety disorders; white matter; sports-related concussions | chronic traumatic encephalopathy; traumatic brain injuries; contact sports; frontotemporal dementia; attention deficit	concussion (390.07, 1.0E-4); chronic traumatic encephalopathy (236.67, 1.0E-4); traumatic brain injury (163.51, 1.0E-4); mild traumatic brain injury (120.97, 1.0E-4); physical activity (96.77, 1.0E-4)

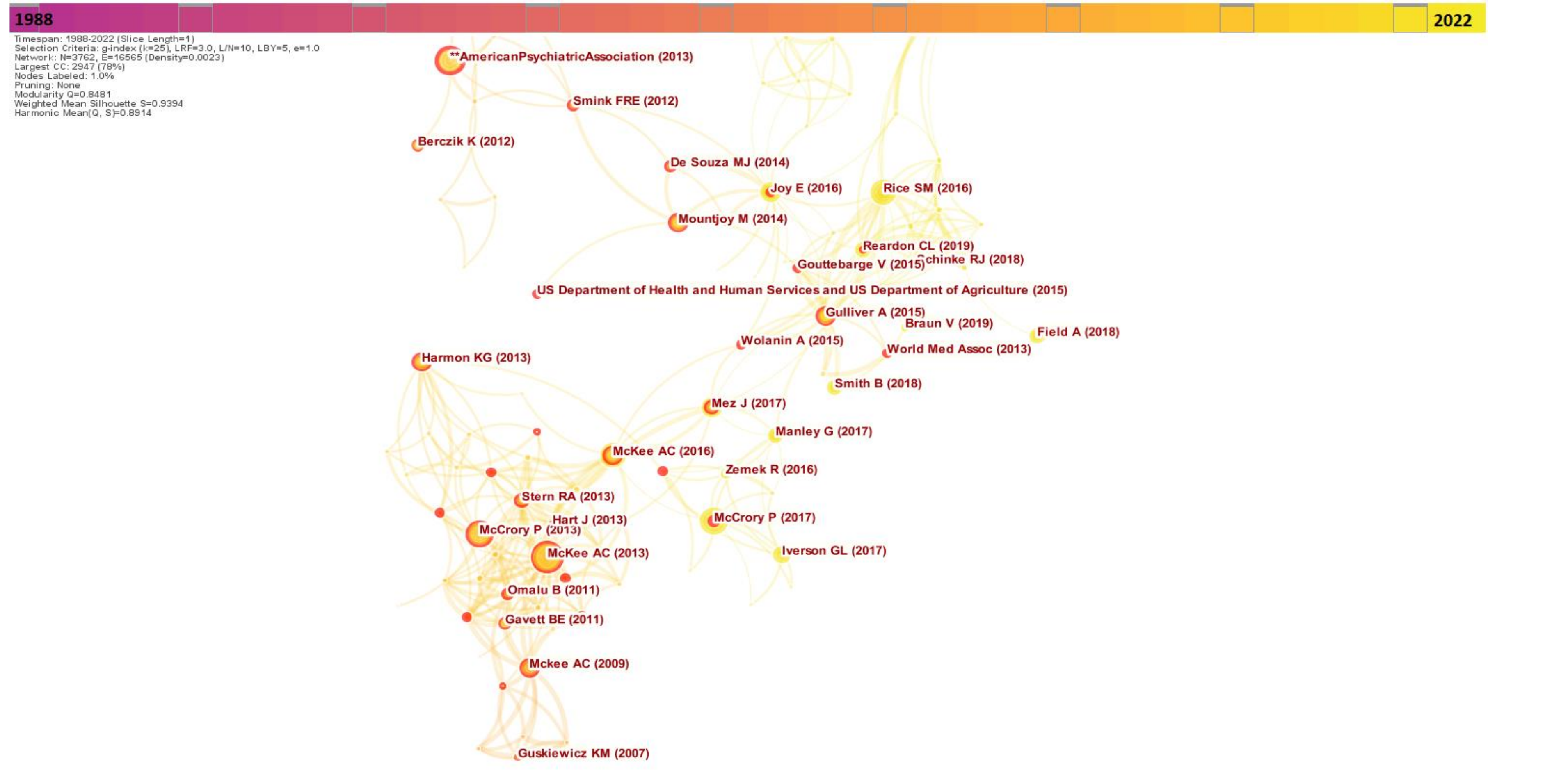

Papers with highest centrality:

Harmon 2013- American Medical Society for Sports Medicine position statement: concussion in sport
McCrory 2013- Consensus statement on Concussion in Sport—The 4th International Conference on Concussion in Sport held in Zurich, November 2012
McKee 2013- The neuropathology of sport
McKee 2016- The first NINDS/NIBIB consensus meeting to define neuropathological criteria for the diagnosis of chronic traumatic encephalopathy
Mez 2017- Clinicopathological Evaluation of Chronic Traumatic Encephalopathy in Players of American Football
Gulliver 2015- The mental health of Australian elite athletes
Mountjoy 2014- The IOC consensus statement: beyond the Female Athlete Triad—Relative Energy Deficiency in Sport (RED-S)
Rice SM 2016- The Mental Health of Elite Athletes: A Narrative Systematic Review

**Cluster 12- Physical exercise and 'children/adolescents/Evidence synthesis'**					
12	75	0.963	2016	physical activity; executive function; cardiorespiratory fitness; acute aerobic exercise; brain activation | mental health; sleep problems; physical education; handgrip strength; bone resorption	adolescents (120.15, 1.0E-4); children (110.83, 1.0E-4); screen time (68.13, 1.0E-4); adolescent (63.3, 1.0E-4); sleep (61.49, 1.0E-4)

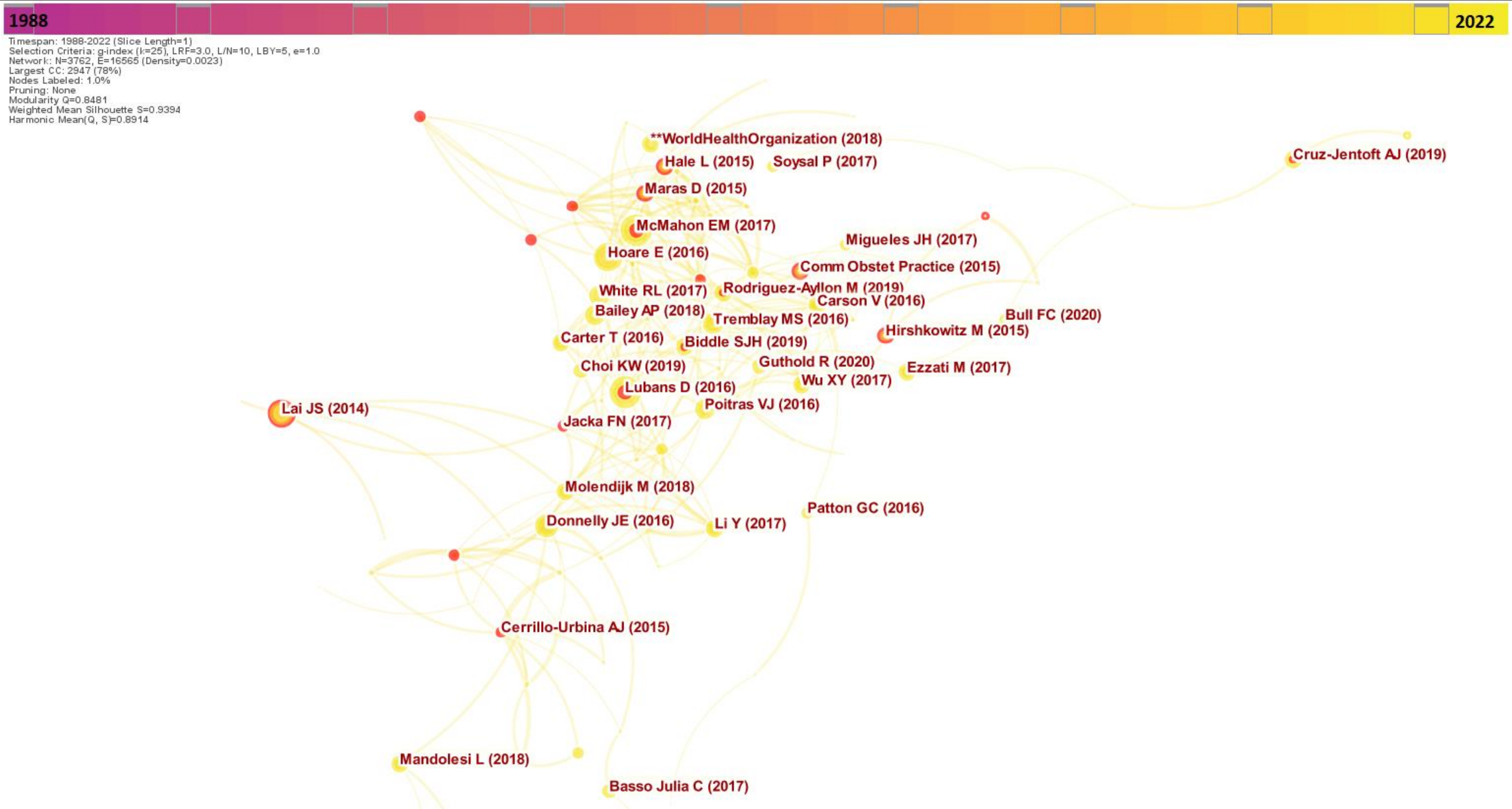

Papers with highest centrality:
Lai 2014- A systematic review and meta-analysis of dietary patterns and depression in community-dwelling adults
Hale 2015- Screen time and sleep among school-aged children and adolescents: a systematic literature review
Maras D 2015- Screen time is associated with depression and anxiety in Canadian youth
Hoare E 2016- physical exercise
White 2017- Domain-Specific Physical Activity and Mental Health: A Meta-analysis
Lubans 2016- Physical Activity for Cognitive and Mental Health in Youth: A Systematic Review of Mechanisms
Donnelly 2016- Physical Activity, Fitness, Cognitive Function, and Academic Achievement in Children: A Systematic Review
Tremblay 2016- Canadian 24-Hour Movement Guidelines for Children and Youth: An Integration of Physical Activity, Sedentary Behaviour, and Sleep
Rodriguez-Ayllon 2019- Role of Physical Activity and Sedentary Behavior in the Mental Health of Preschoolers, Children and Adolescents: A Systematic Review and Meta-Analysis
Hirshkowitz 2015- National Sleep Foundation's sleep time duration recommendations: methodology and results summary

**Cluster 13- Physical exercise and ‘female athletes/eating disorders’**					
13	74	0.967	2000	eating disorders; menstrual dysfunction; bone mineral density; energy intake; stress fractures | disordered eating; female athlete triad; eating disorder; elite rhythmic gymnasts; menstrual disturbances	eating disorders (105.47, 1.0E-4); amenorrhea (91.13, 1.0E-4); anorexia nervosa (57.76, 1.0E-4); disordered eating (56.12, 1.0E-4); osteoporosis (53.37, 1.0E-4)

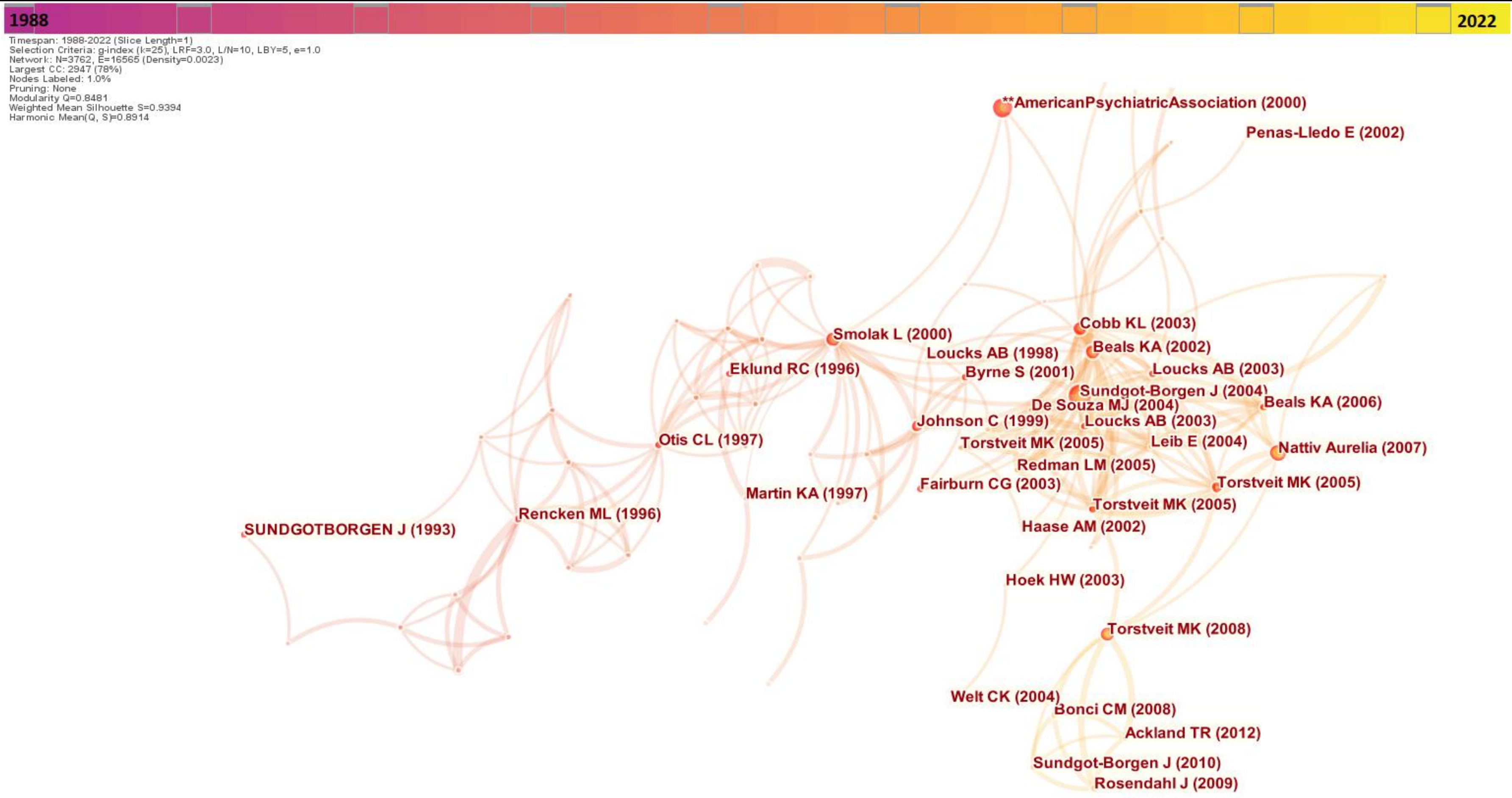

Papers with highest centrality:

Smolak 2000- Female athletes and eating problems: a meta-analysis
Cobb 2003- Disordered Eating, Menstrual Irregularity, and Bone Mineral Density in Female Runners
Beals 2002- Disorders of the female athlete triad among collegiate athletes
Sundgot-borgen 2004- Prevalence of eating disorders in elite athletes is higher than in the general population
Nattiv Aurelia 2007- American College of Sports Medicine position stand. The female athlete triad
Torstveit 2005- The Female Athlete Triad: Are Elite Athletes at Increased Risk?
Tirstveit 2008- Prevalence of eating disorders and the predictive power of risk models in female elite athletes: A controlled study
Otis 1997- American College of Sports Medicine position stand. The Female Athlete Triad

**Cluster 14- Physical exercise and ‘exercise electrocardiography’**					
14	65	0.987	1987	exercise electrocardiography; myocardial ischemia; hr slope; coronary artery disease; computerized analysis | framingham offspring study; risk stratification; st segment depression; coronary heart disease; coronary artery disease	heart rate (24.3, 1.0E-4); exercise electrocardiography (21.99, 1.0E-4); myocardial ischemia (19.09, 1.0E-4); framingham offspring study (12.87, 0.001); st/hr index (12.87, 0.001)

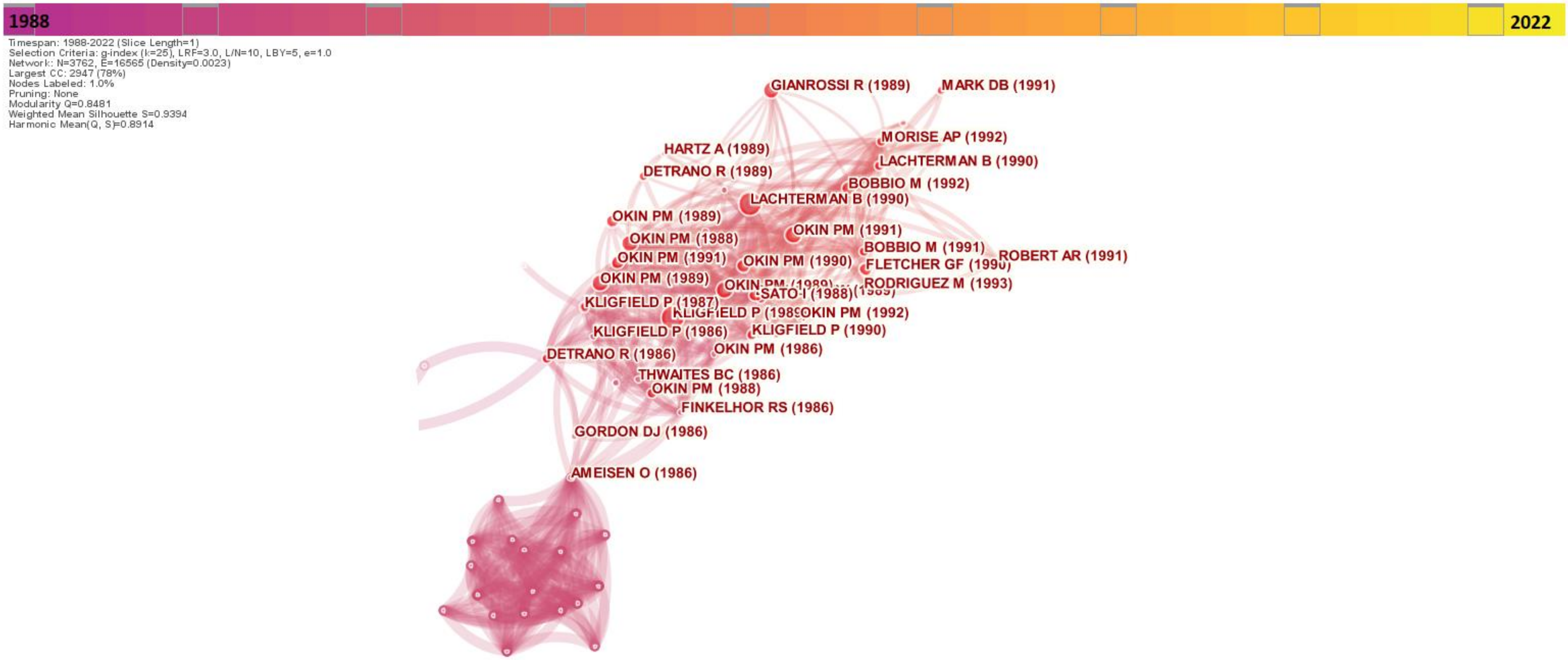

Papers with highest centrality:

Gianrossi 1989- Exercise-induced ST depression in the diagnosis of coronary artery disease. A meta-analysis.
Lachterman 1990- "Recovery only" ST-segment depression and the predictive accuracy of the exercise test.
Okin 1989- Recovery-phase patterns of ST segment depression in the heart rate domain. Identification of coronary artery disease by the rate-recovery loop.
Okin 1991- Heart rate adjustment of exercise-induced ST segment depression. Improved risk stratification in the Framingham Offspring Study
Fletcher 1990- Statement on Exercise: Benefits and Recommendations for Physical Activity Programs for All Americans - A Statement for Health Professionals by the Committee on Exercise and Cardiac Rehabilitation of the Council on Clinical Cardiology, American Heart Association
Bobbio 1991- Relation of Physical Activity and Self-Esteem

Cluster 15- Physical exercise and 'NAFLD'					
15	55	0.998	2011	non-alcoholic fatty liver disease; non-alcoholic steatohepatitis; hepatocellular carcinoma; intrahepatic cholangiocarcinoma; liraglutide | physical activity; insulin resistance; diabetes mellitus; liraglutide; obesity fatty liver	non-alcoholic fatty liver disease (236.71, 1.0E-4); non-alcoholic steatohepatitis (164.56, 1.0E-4); nafld (150.17, 1.0E-4); nash (78.46, 1.0E-4); steatosis (71.31, 1.0E-4)

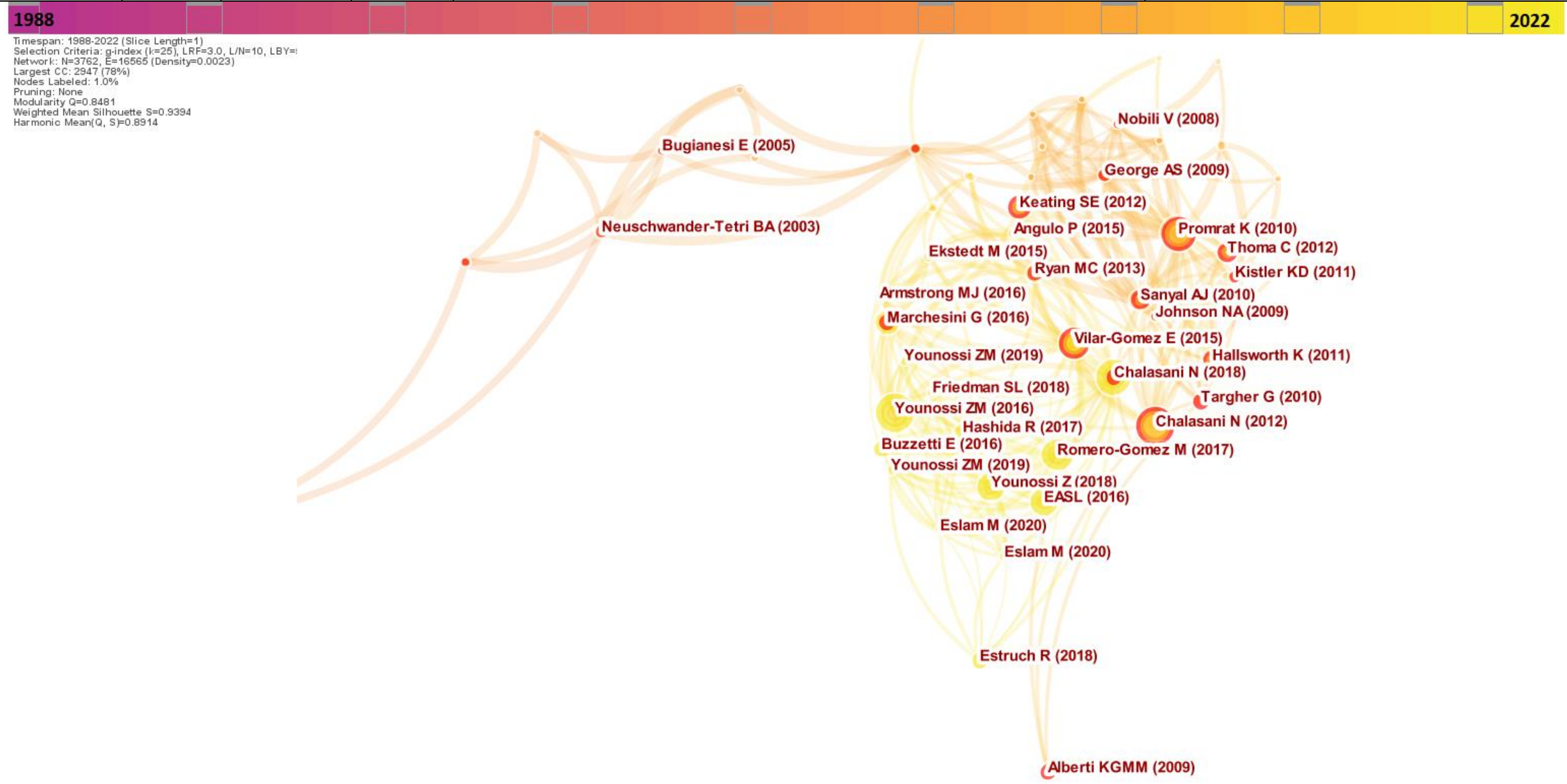

Papers with highest centrality:
Younossi 2016- Global epidemiology of nonalcoholic fatty liver disease-Meta-analytic assessment of prevalence, incidence, and outcomes
Romero-gomez 2017- Treatment of NAFLD with diet, physical activity and exercise
Marchesini 2016- Diet, Weight Loss, and Liver Health in Nonalcoholic Fatty Liver Disease: Pathophysiology, Evidence, and Practice
Keating 2012- Exercise and non-alcoholic fatty liver disease: a systematic review and meta-analysis
Promrat 2010- Randomized controlled trial testing the effects of weight loss on nonalcoholic steatohepatitis
Thoma 2012- Lifestyle interventions for the treatment of non-alcoholic fatty liver disease in adults: A systematic review
Chalasani 2012- The diagnosis and management of nonalcoholic fatty liver disease: Practice guidance from the American Association for the Study of Liver Diseases
Vilar-gomez 2015- Weight Loss Through Lifestyle Modification Significantly Reduces Features of Nonalcoholic Steatohepatitis
Sanyal 2010- Pioglitazone, vitamin E, or placebo for nonalcoholic steatohepatitis

Cluster 16- Physical exercise and ‘cancer’					
16	52	0.993	2009	physical activity; breast cancer survivors; health-related quality; sedentary time; outcomes | breast cancer; controlled trial; exercise therapy; survivorship; womac	breast cancer (124.04, 1.0E-4); cancer (115.41, 1.0E-4); fatigue (105.62, 1.0E-4); quality of life (88.05, 1.0E-4); oncology (72.53, 1.0E-4)

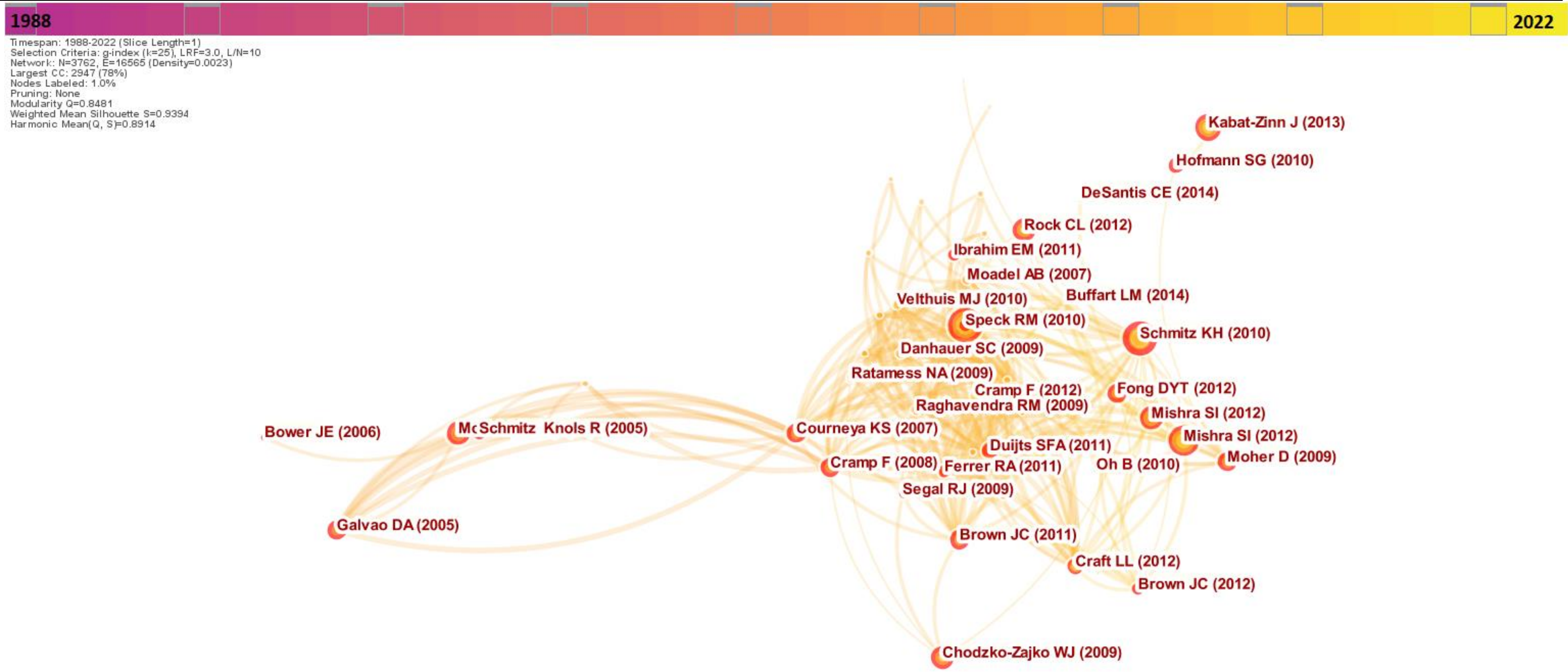

Papers with highest centrality:

Kabat-Zinn 2013- Full Catastrophe Living, Revised Edition: How to cope with stress, pain and illness using mindfulness meditation
Rock 2012- Nutrition and physical activity guidelines for cancer survivors
Speck 2010- An update of controlled physical activity trials in cancer survivors: a systematic review and meta-analysis
Schmitz 2010- Weight lifting for women at risk for breast cancer-related lymphedema: a randomized trial
Fong 2012- Physical activity for cancer survivors: meta-analysis of randomized controlled trials
Mishra 2012- Exercise interventions on health-related quality of life for people with cancer during active treatment
Moher 2009- Preferred Reporting Items for Systematic Reviews and Meta-Analyses: The PRISMA Statement
Dujits 2011- Effectiveness of behavioral techniques and physical exercise on psychosocial functioning and health-related quality of life in breast cancer patients and survivors--a meta-analysis
Courneya 2007- Effects of aerobic and resistance exercise in breast cancer patients receiving adjuvant chemotherapy: a multicenter randomized controlled trial
Cramp 2008- Exercise for the management of cancer-related fatigue in adults

Cluster 17- Physical exercise and ‘catecholamines’

17	46	0.987	1989	exercise; mood; catecholamines; coping; stress | normal coronary arteriograms; catecholamines; mood; continuous ventricular function monitor; exercise	catecholamines (23.93, 1.0E-4); normal coronary arteriograms (23.93, 1.0E-4); angina pectoris and normal coronary arteriograms (11.94, 0.001); tl-201 scintigraphy (11.94, 0.001); supine exercise (11.94, 0.001)

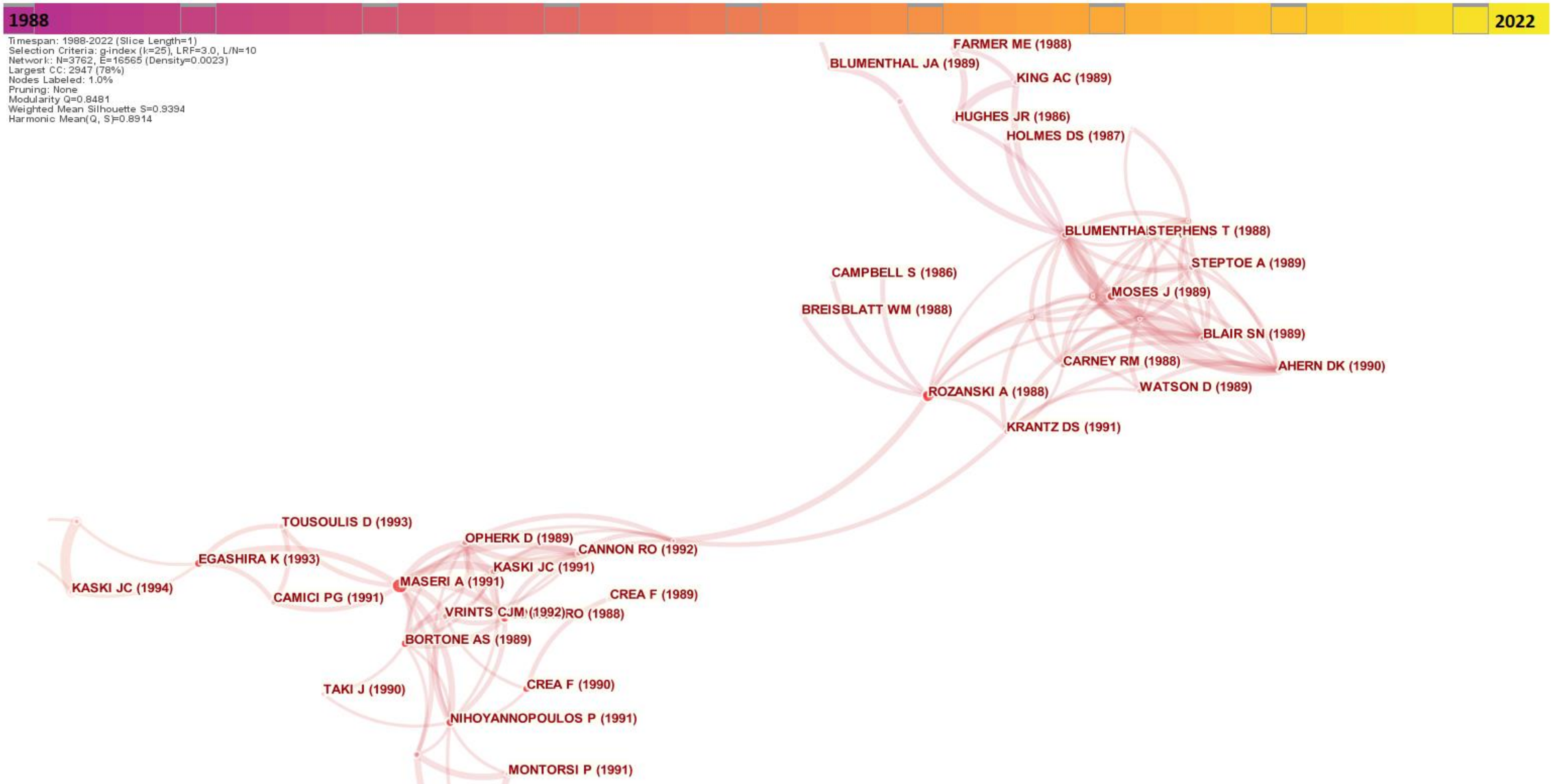

Papers with highest centrality:
Maseri 1991- Coronary artery spasm and vasoconstriction. The case for a distinction
Bortone 1989- Abnormal coronary vasomotion during exercise in patients with normal coronary arteries and reduced coronary flow reserve.
Crea 1990- Exercise-based cardiac rehabilitation for adults with stable angina
Rozanski 1988- Mental stress and the induction of silent myocardial ischemia in patients with coronary artery disease
Moses 1989- The effects of exercise training on mental well-being in the normal population: a controlled trial exercise
Steptoe 1989- The effects of exercise training on mood and perceived coping ability in anxious adults from the general population

**Cluster 18- Physical exercise and 'severe mental illness'**					
18	46	0.985	2007	physical activity; health promotion; mental health care; individual treatment; health education | metabolic syndrome; severe mental illness; weight loss; cardiometabolic risk factors; somatic treatment	schizophrenia (91.75, 1.0E-4); metabolic syndrome (32.34, 1.0E-4); depression (28.6, 1.0E-4); severe mental illness (22.84, 1.0E-4); nursing (20.4, 1.0E-4)

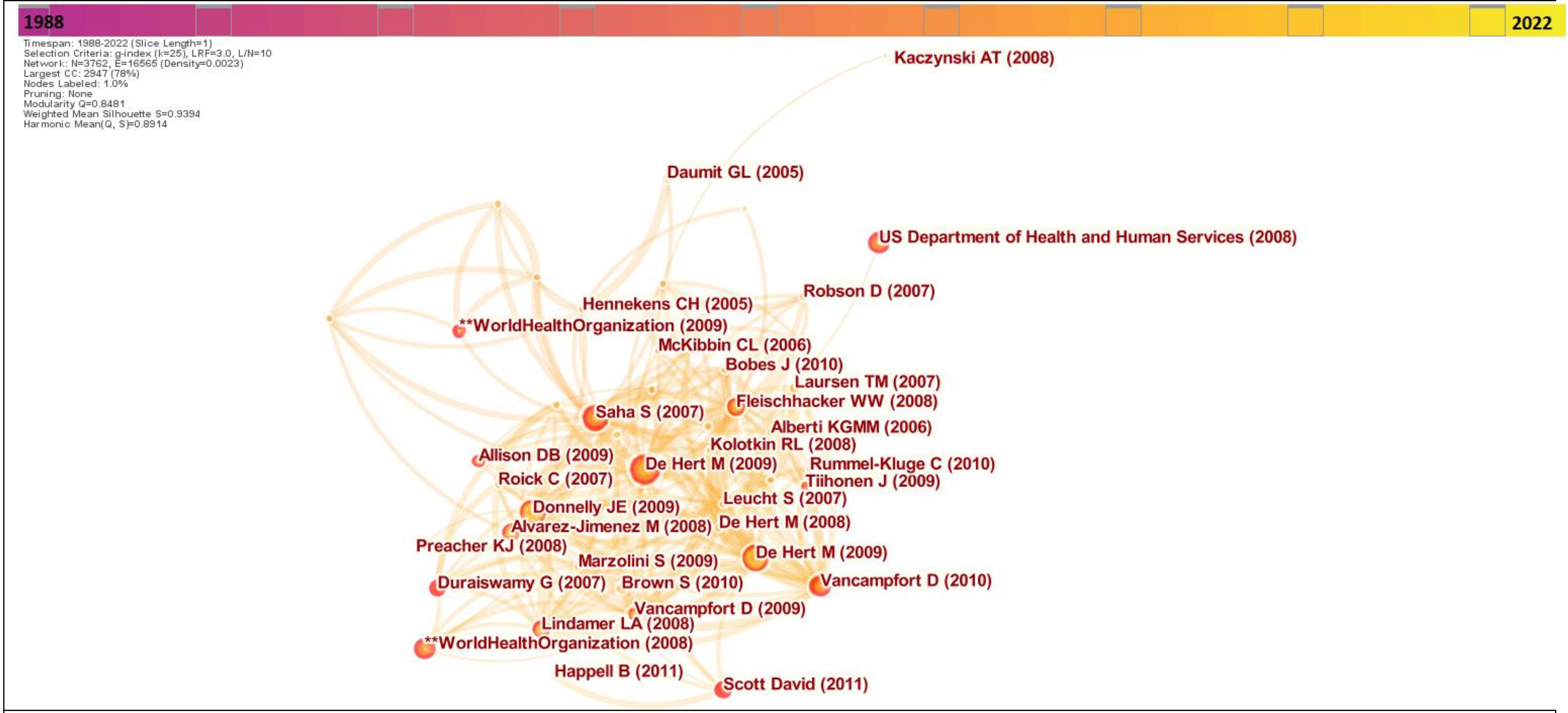

Papers with highest centrality:
Saha 2007- A Systematic Review of Mortality in Schizophrenia Is the Differential Mortality Gap Worsening Over Time?
De Hert 2009- Metabolic syndrome in people with schizophrenia: a review
Vancampfort 2010- A systematic review of correlates of physical activity in patients with schizophrenia
Fleischhacker 2008- Comorbid somatic illnesses in patients with severe mental disorders: clinical, policy, and research challenges
Allison 2009- Fixed Effects Regression Models
Lindamer 2008- Assessment of physical activity in middle-aged and older adults with schizophrenia
Duraiswamy 2007- Yoga therapy as an add-on treatment in the management of patients with schizophrenia--a randomized controlled trial

Cluster 19- Physical exercise and ‘self-confidence’

19	38	0.995	1998	mental imagery; goal profiles; goal orientation; isccs; exercise therapy | isccs; coping; performance; emotion; validity	self-confidence (43.47, 1.0E-4); direction (21.69, 1.0E-4); mental imagery (21.69, 1.0E-4); debilitators (21.69, 1.0E-4); facilitators (21.69, 1.0E-4)

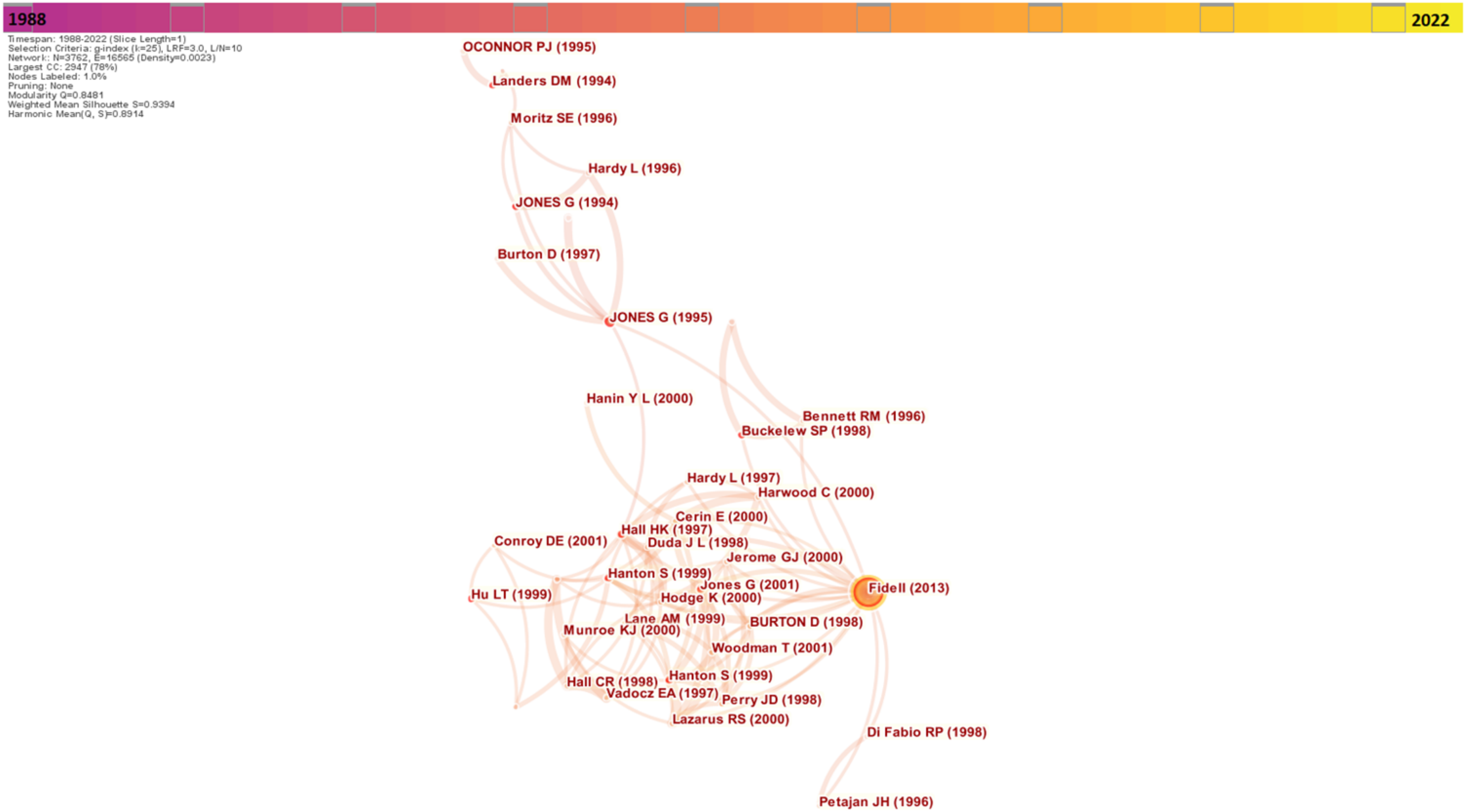

Papers with highest centrality:

Fidell 2013- Using Multivariate Statistics, 6th Edition
Burton 1998- Measuring competitive state anxiety.
Hall 1997- Self-confidence and anxiety interpretation: A qualitative investigation
Jones 1994- Intensity and interpretation of anxiety symptoms in elite and non-elite sports performers
Hanton 1999- Self-confidence and anxiety interpretation: A qualitative investigation

**Cluster 20- Physical exercise and 'alzheimers disease'**					
20	16	0.993	1997	alzheimers disease; cholinesterase inhibitors; therapeutic use; biopsychological arousal theory; clinical course | physical activity; exercise psychology; biopsychological arousal theory; functional impairment; alzheimers disease	arousal theory (13.57, 0.001); psychological outcomes (13.57, 0.001); functional impairment (13.57, 0.001); ad clinical course (13.57, 0.001); pharmacoeconomics (13.57, 0.001)

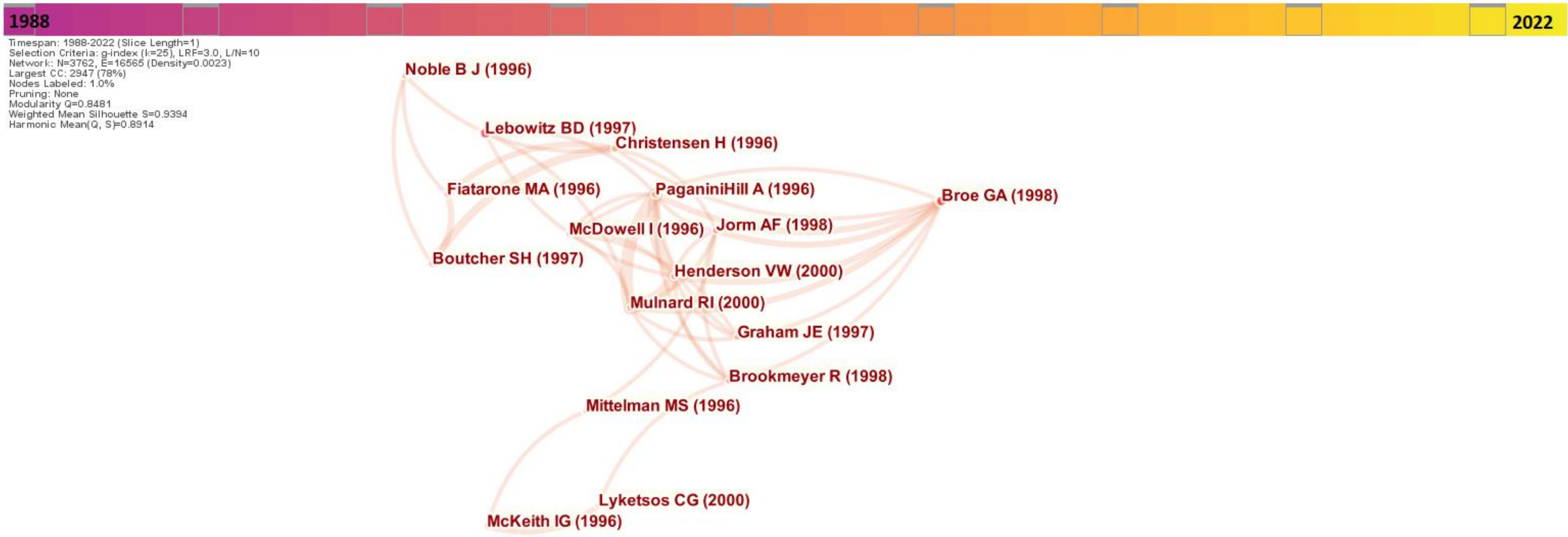

Papers with highest centrality:

Broe 1998- Health habits and risk of cognitive impairment and dementia in old age: a prospective study on the effects of exercise, smoking and alcohol consumption

Lebowitz 1997- Diagnosis and treatment of depression in late life. Consensus statement update

PaganiniHill 1996- Estrogen replacement therapy and risk of Alzheimer disease

Henderson 2000- Estrogen for Alzheimer's disease in women Randomized, double-blind, placebo-controlled trial

Graham 1997- Differentiating the roles of the hippocampal complex and the neocortex in long-term memory storage: evidence from the study of semantic dementia and Alzheimer's disease

Brookmeyer 1998- Projections of Alzheimer's disease in the United States and the public health impact of delaying disease onset

**Cluster 24- Physical exercise and 'multiple sclerosis'**					
24	9	0.999	2014	multiple sclerosis; behaviour change techniques; endurance training; clinical exercise physiology; coordinative training | physical activity; behaviour change techniques; endurance training; clinical exercise physiology; coordinative training	multiple sclerosis (118.85, 1.0E-4); fatigue (37.49, 1.0E-4); wellness (20.3, 1.0E-4); rehabilitation (12.63, 0.001); neuroimmunology (10.14, 0.005)
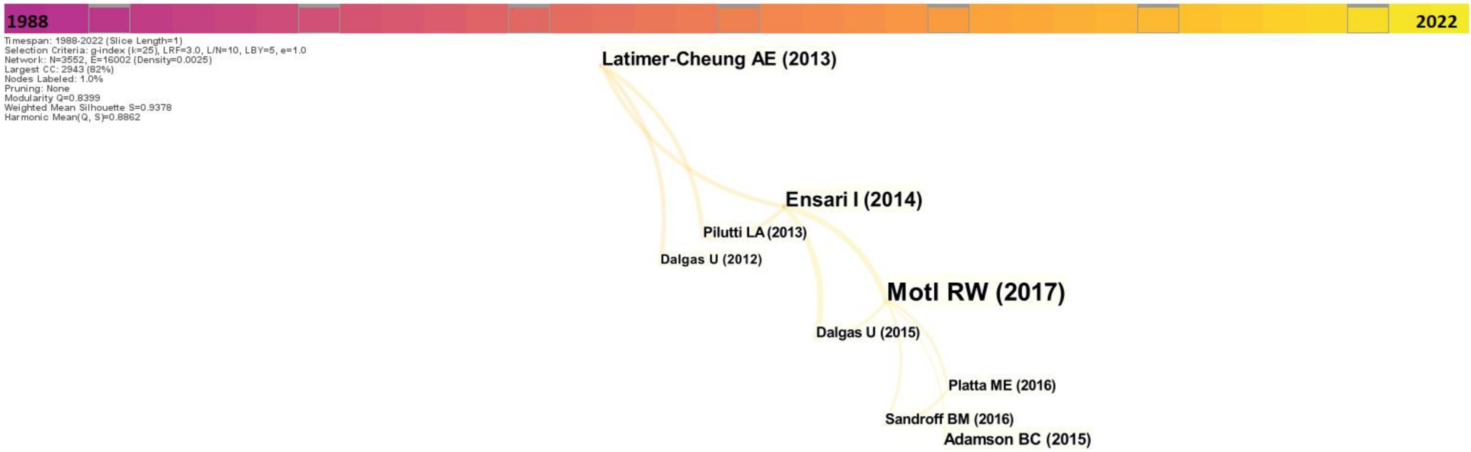					
**Papers with highest centrality:**<br>Motl 2017- Exercise in patients with multiple sclerosis<br>Ensari I 2014- Exercise training improves depressive symptoms in people with multiple sclerosis: results of a meta-analysis<br>Platta 2016- Effect of Exercise Training on Fitness in Multiple Sclerosis: A Meta-Analysis<br>Dalgas 2015- The effect of exercise on depressive symptoms in multiple sclerosis based on a meta-analysis and critical review of the literature					
**Cluster 31- Physical exercise and 'cardiometabolic risk'**					
31	5	0.999	2014	physical activity; compositional data analysis; cardiometabolic risk; sedentary behaviors; mental health | isotemporal substitution; cardiometabolic risk; sedentary behaviors; coronary artery risk development; mental health	isotemporal substitution (37.88, 1.0E-4); sport siences (12.57, 0.001); accerelometry (12.57, 0.001); objective measure (12.57, 0.001); oa (12.57, 0.001)
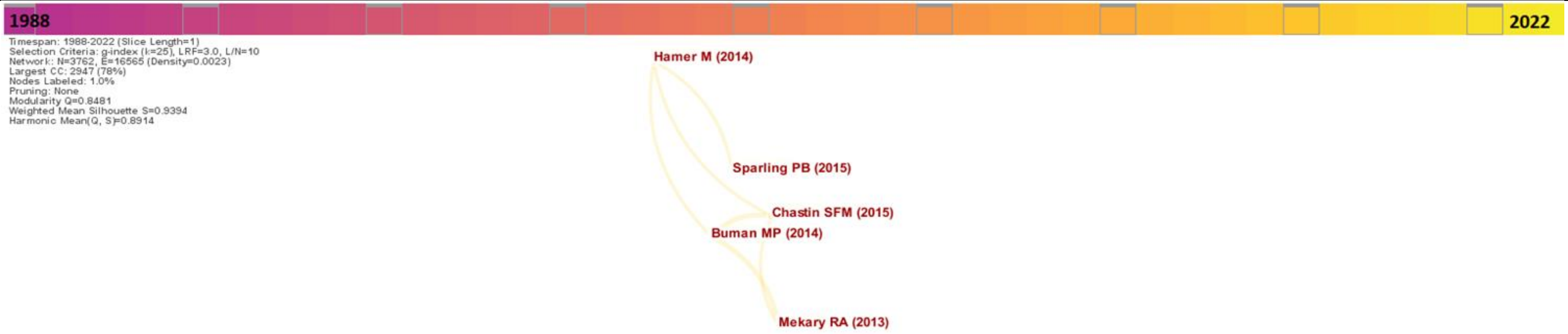					
**Papers with highest centrality:**<br>Hamer 2014- Taking up physical activity in later life and healthy ageing: the English longitudinal study of ageing<br>Mekary 2013- Isotemporal substitution analysis for physical activity, television watching, and risk of depression<br>Buman 2014- Reallocating time to sleep, sedentary behaviors, or active behaviors: associations with cardiovascular disease risk biomarkers, NHANES 2005-2006<br>Chastin 2015- Combined Effects of Time Spent in Physical Activity, Sedentary Behaviors and Sleep on Obesity and Cardio-Metabolic Health Markers: A Novel Compositional Data Analysis Approach<br>Sparling 2015- Recommendations for physical activity in older adults					

Cluster 40- Physical exercise and 'behaviour change'					
40	7	0.996	2013	physical activity; chronic heart failure; 6-min walk test; general diabetes; independent mobility | behaviour change; sedentary behaviour; self-management strategies; implementation strategies; external facilitation	behaviour change (27, 1.0E-4); occupational health (15.81, 1.0E-4); self-management (14.1, 0.001); osteoarthritis (14.1, 0.001); depression (13.35, 0.001)

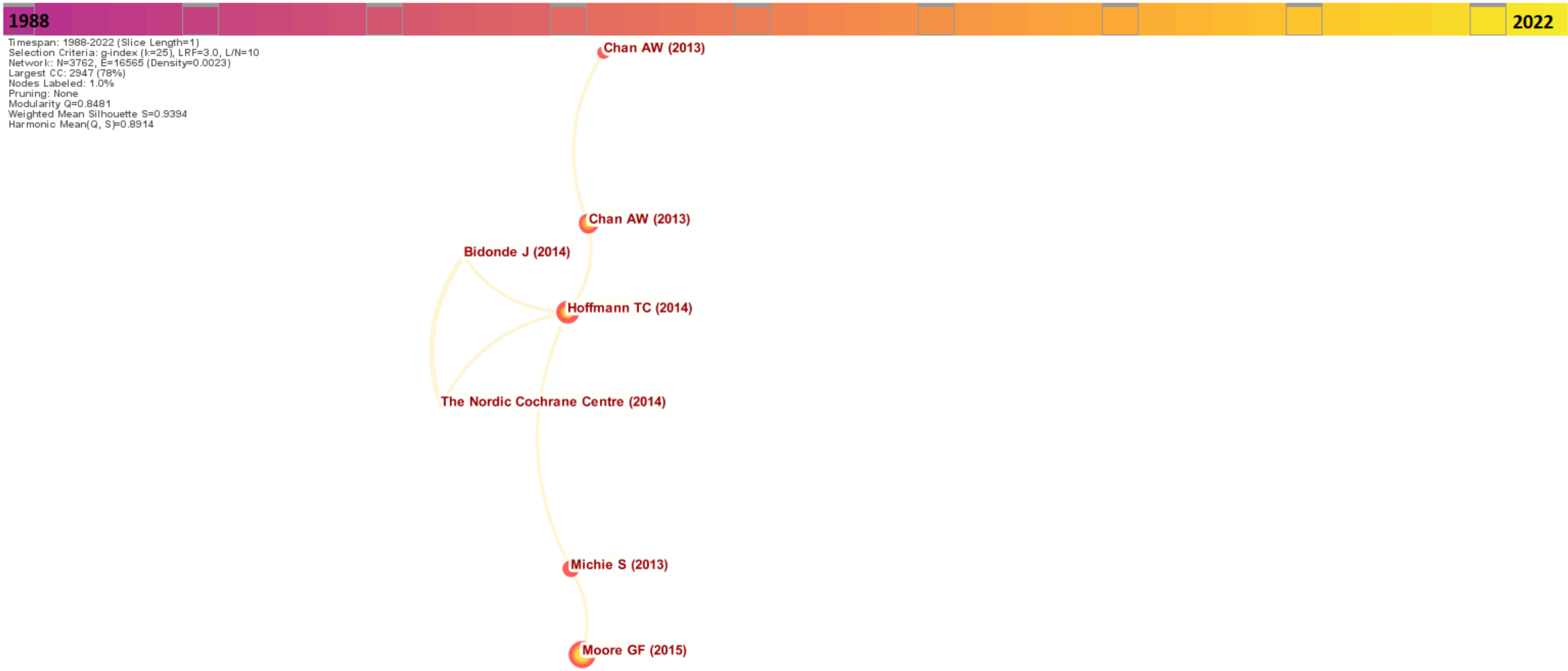

Papers with highest centrality:
Chan 2013- SPIRIT 2013 Statement: Defining Standard Protocol Items for Clinical Trials
Chan 2013- SPIRIT 2013 explanation and elaboration: guidance for protocols of clinical trials
Hoffmann 2014- Moderate-to-High Intensity Physical Exercise in Patients with Alzheimer's Disease: A Randomized Controlled Trial
Michie 2013- The Behavior Change Technique Taxonomy (v1) of 93 Hierarchically Clustered Techniques: Building an International Consensus for the Reporting of Behavior Change Interventions
Moore 2015- Process evaluation of complex interventions: Medical Research Council guidance

**Cluster 48- Physical exercise and 'pre-diabetes mellitus'**					
48	4	0.994	2012	education; older adults; depression; diabetes; self-care | depressive symptoms; health disparities; childhood disadvantage; education; regression tree model	pre-diabetes mellitus (13.15, 0.001); type 2 diabetes mellitus risk (13.15, 0.001); self-management behaviors (13.15, 0.001); self-assessment score (13.15, 0.001); sulfonylurea (13.15, 0.001)
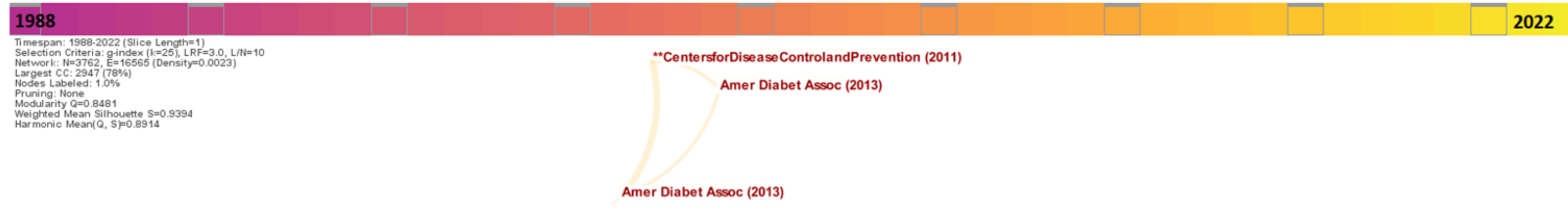					
**Papers with highest centrality:**<br>American Diabetes Association 2013- Standards of Medical Care in Diabetes—2013					
**Cluster 50- Physical exercise and 'motivation'**					
50	4	0.997	2005	approach; mastery; avoidance; competition; descriptive analysis | motivation; achievement; burnout; perfectionism; athletics	motivation (29.71, 1.0E-4); avoidance (23.31, 1.0E-4); achievement (23.31, 1.0E-4); mastery (23.31, 1.0E-4); approach (23.31, 1.0E-4)
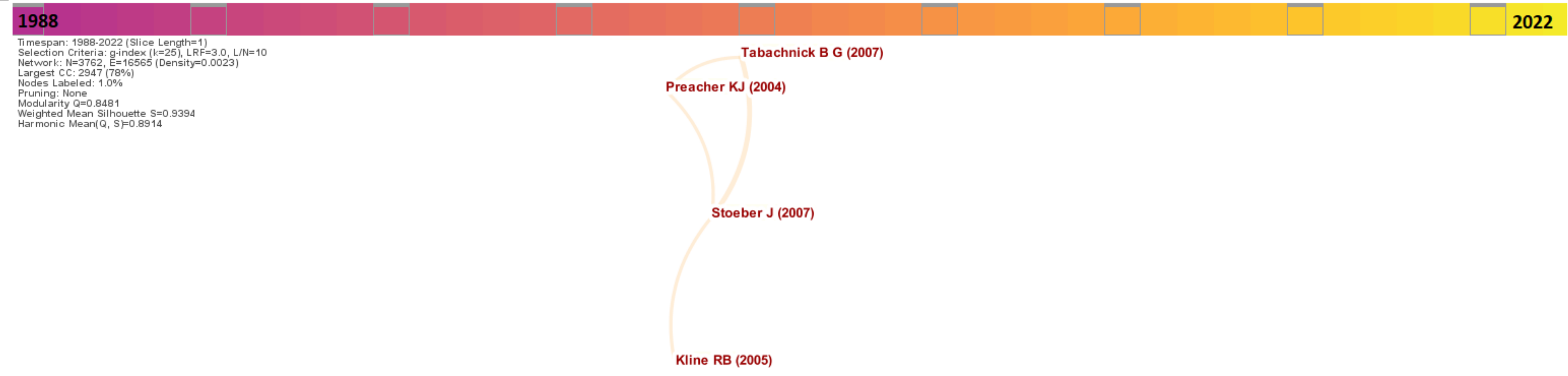					
**Papers with highest centrality:**<br>Kline 2005- Principles and practice of structural equation modeling, 2nd ed.<br>Stoeber 2007- Perfectionism in adolescent school students: Relations with motivation, achievement, and well-being.<br>Preacher 2004- SPSS and SAS procedures for estimating indirect effects in simple mediation models.<br>Tabachnick 2007- Using multivariate statistics (5th ed.)					

Figure 1 text:

Figure 2 text:

B. Detail of reference co-citation network clusters (2016-2021)

Co-citation references networks (2016-2021)

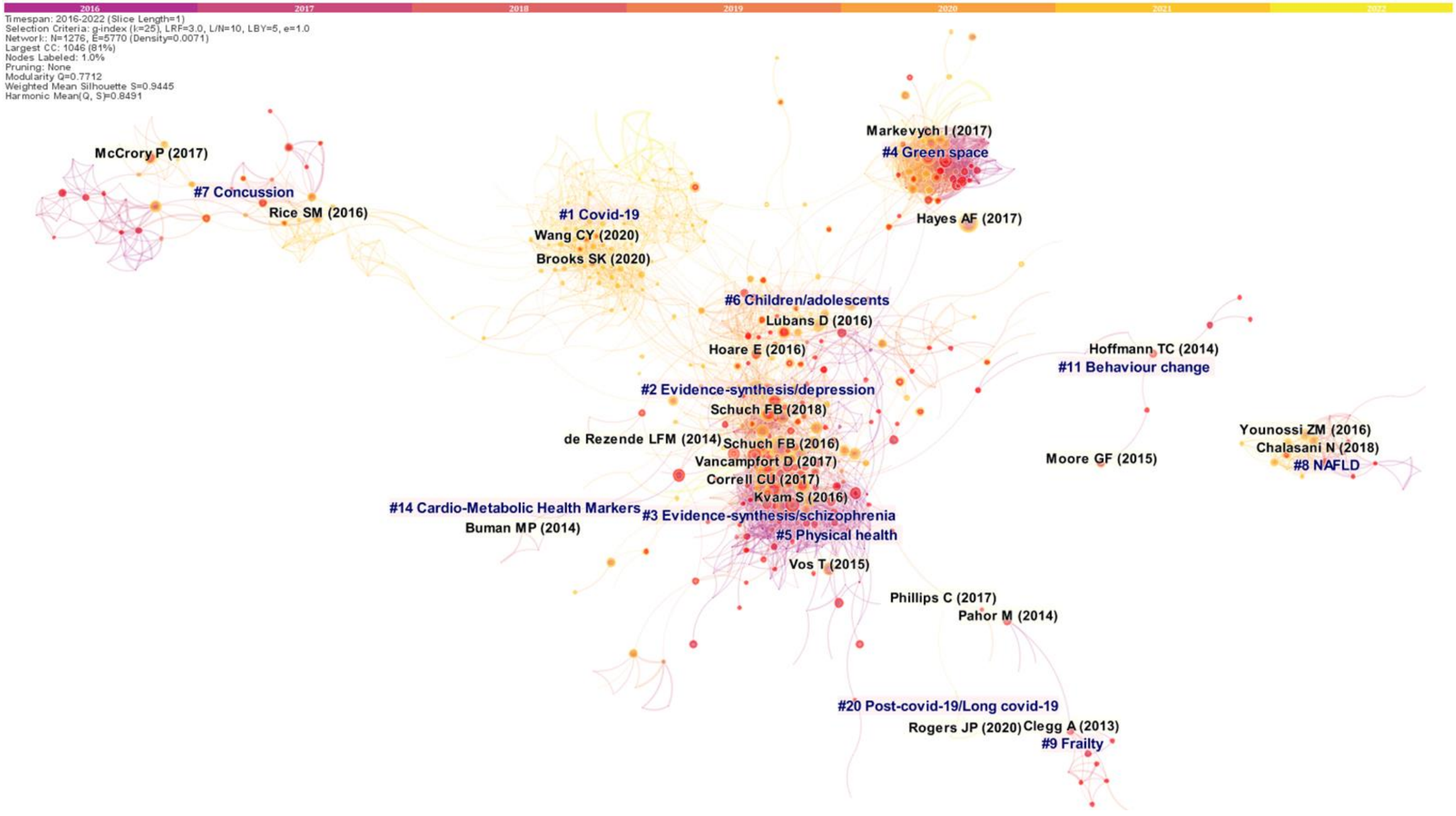

Co-citation reference networks with corresponding clusters (2016-2021)

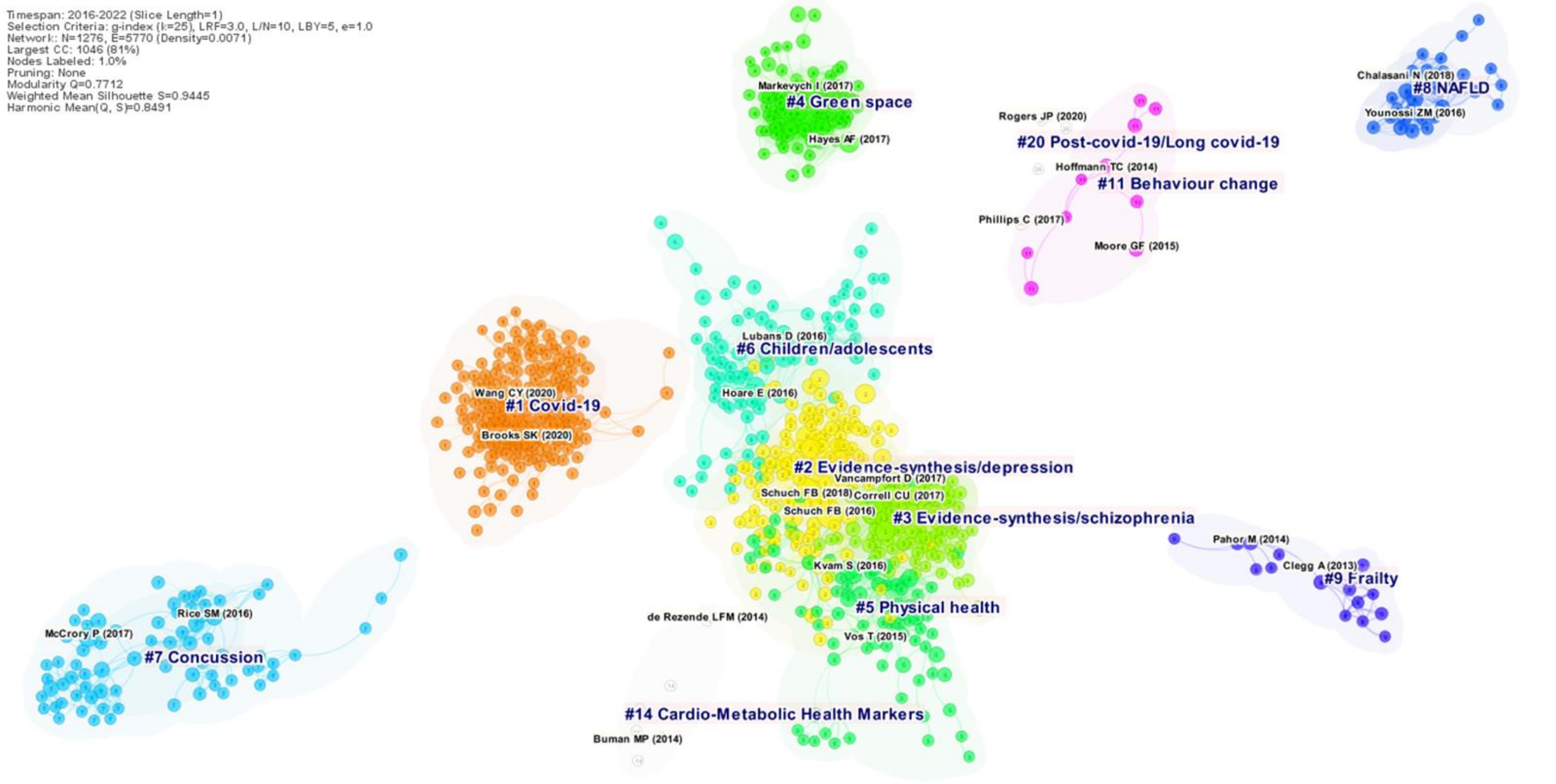

Clusters are numbered depending on their size, starting with the largest (0). Clusters labels are extracted using the log-likelihood ratio. Node colour (or numbers) indicates cluster membership. Lines connect nodes that are highly co-cited.

Co-citation reference network time map (2016-2021)

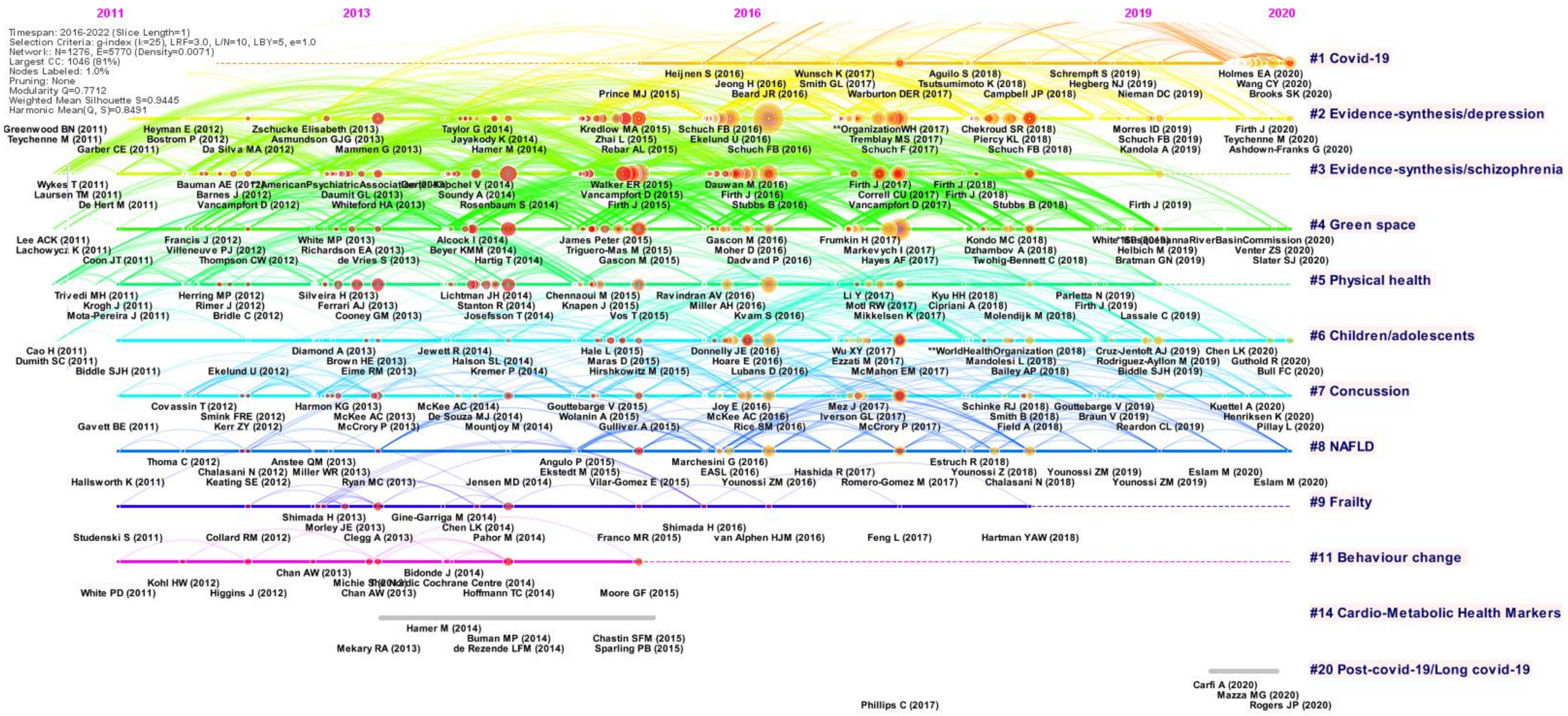

Cluster 0- Physical activity and 'evidence synthesis/cognitive decline'

0	221	0.938	2015	dementia (469.6, 1.0E-4); alzheimers disease (257.76, 1.0E-4); mild cognitive impairment (184.71, 1.0E-4); cognition (176.48, 1.0E-4); aging (144.18, 1.0E-4)

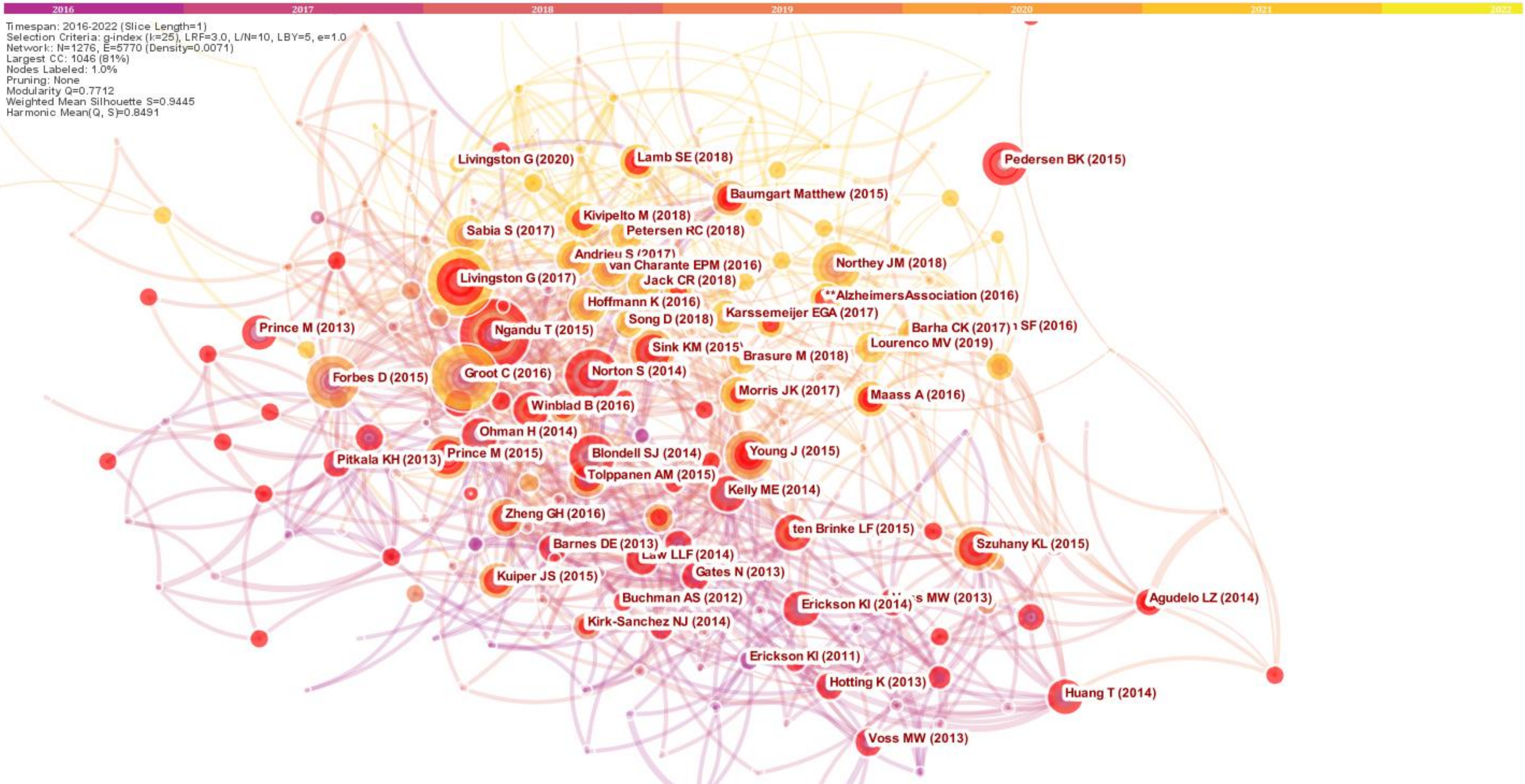

Papers with highest centrality:

Pedersen 2015- Exercise as medicine - evidence for prescribing exercise as therapy in 26 different chronic diseases
Livingston 2017- Dementia prevention, intervention, and care
Groot 2016- The effect of physical activity on cognitive function in patients with dementia: A meta-analysis of randomized control trials
Norton 2014- Potential for primary prevention of Alzheimer's disease: an analysis of population-based data
Sabia 2017- Physical activity, cognitive decline, and risk of dementia: 28 year follow-up of Whitehall II cohort study
Forbes 2015-Exercise programs for people with dementia
Szuhany 2015- A meta-analytic review of the effects of exercise on brain-derived neurotrophic factor
Northery 2018- Exercise interventions for cognitive function in adults older than 50: a systematic review with meta-analysis

**Cluster 1- Physical exercise and ‘covid-19’**				
1	172	0.987	2019	covid-19 (573.01, 1.0E-4); pandemic (137.48, 1.0E-4); lockdown (96.41, 1.0E-4); coronavirus (76.22, 1.0E-4); quarantine (48.24, 1.0E-4)

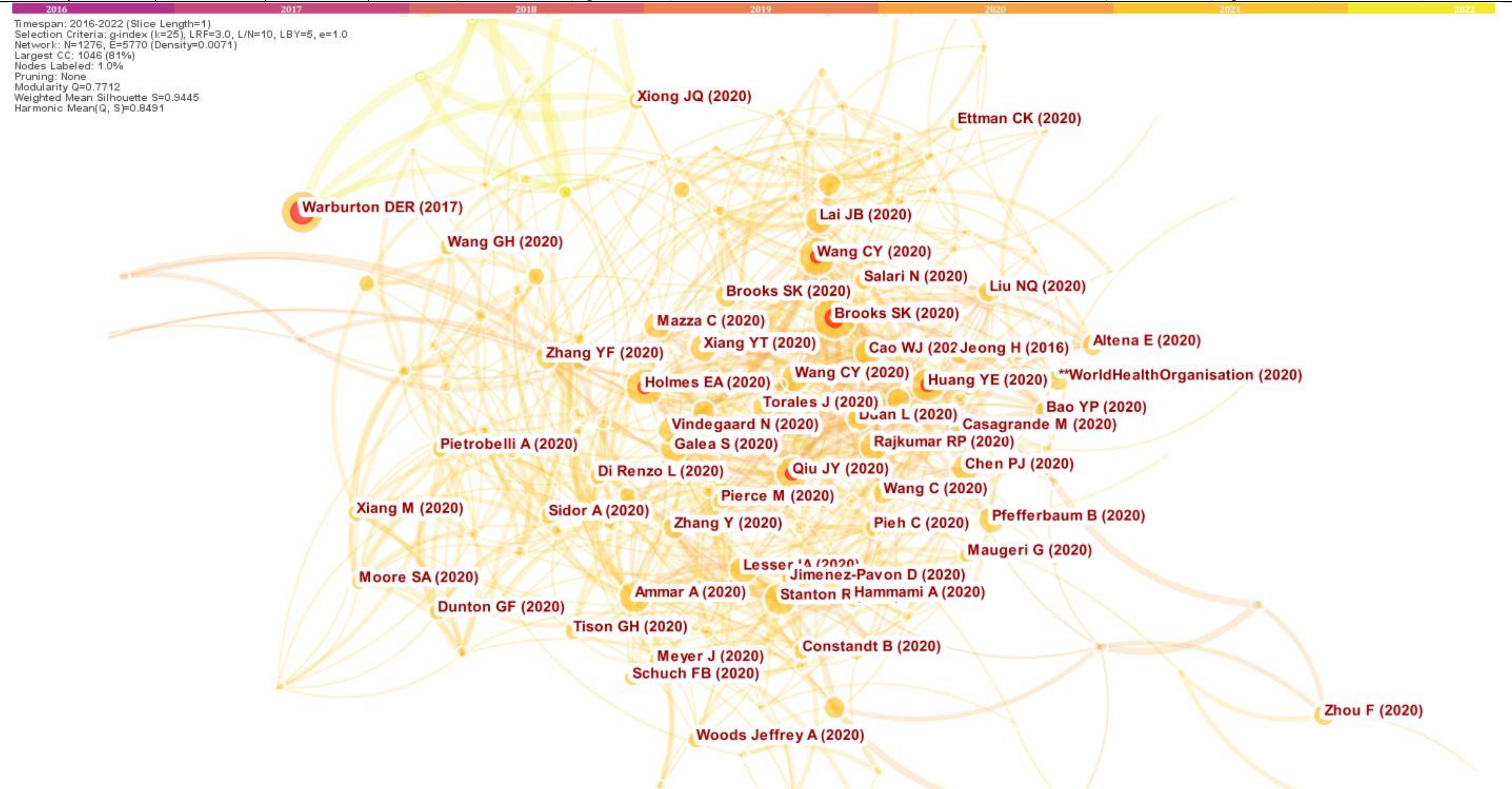

Papers with highest centrality:

Warburton 2017- Health benefits of physical activity: a systematic review of current systematic reviews
Brooks 2020- The psychological impact of quarantine and how to reduce it: rapid review of the evidence
Qiu 2020- Mental wellness system for COVID-19
Holmes 2020- Multidisciplinary research priorities for the COVID-19 pandemic: a call for action for mental health science
Rajkumar 2020- COVID-19 and mental health: A review of the existing literature
Xiang YT 2020- The effect of regular physical activity on aggression and quality of life of students during corona quarantine (Covid-19)

**Cluster 2- Physical exercise and ‘evidence synthesis/depression’**				
2	142	0.819	2016	depression (68.38, 1.0E-4); physical activity (64.35, 1.0E-4); sitting (51.09, 1.0E-4); anxiety (41.45, 1.0E-4); sedentary behavior (35.14, 1.0E-4)

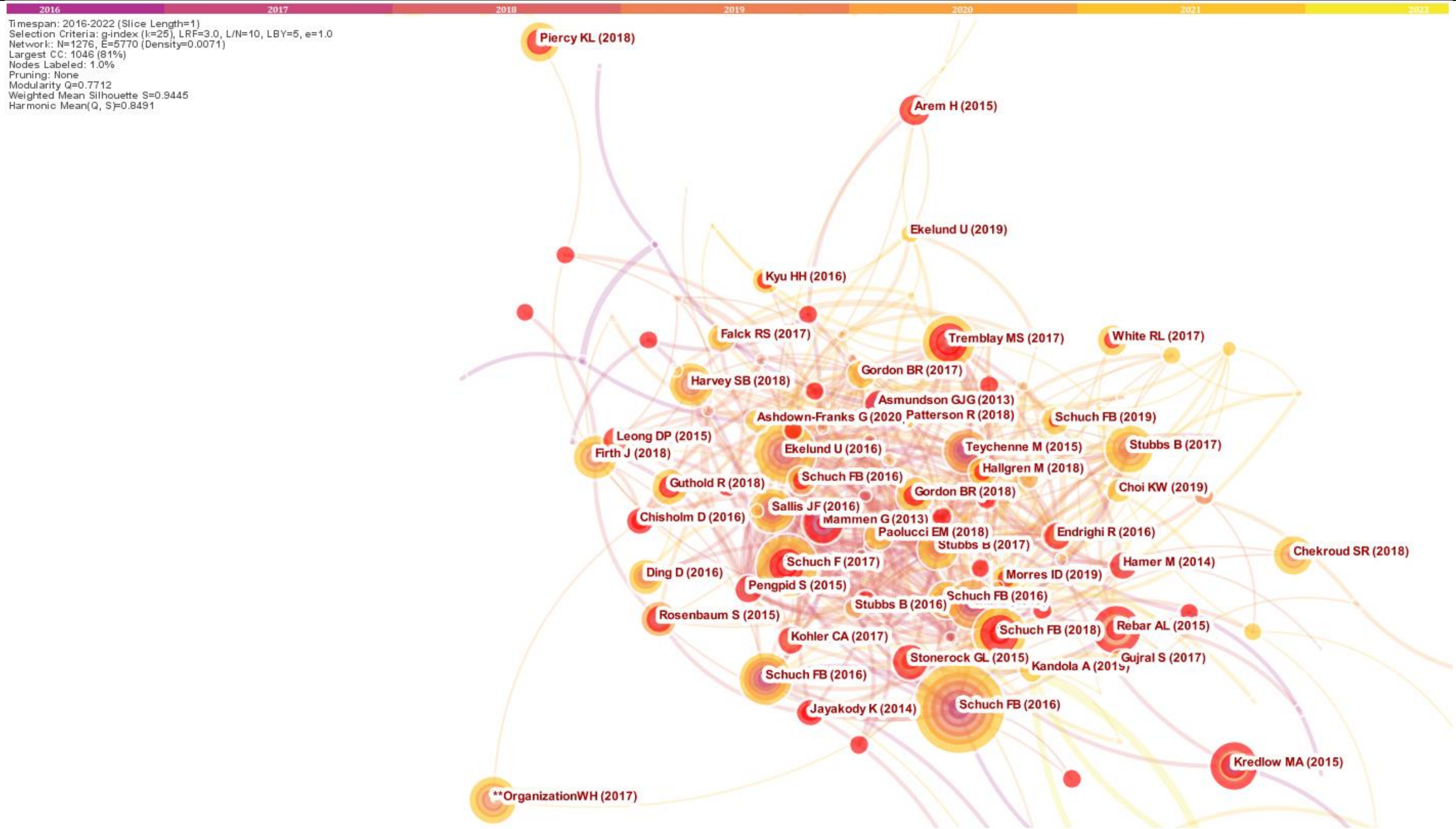

Papers with highest centrality:
Schuch 2016- Exercise as a treatment for depression: A meta-analysis adjusting for publication bias
Kredlow 2015- The effects of physical activity on sleep: a meta-analytic review
Schuch 2017- Physical activity and sedentary behavior in people with major depressive disorder: A systematic review and meta-analysis
Ekelund 2016- Does physical activity attenuate, or even eliminate, the detrimental association of sitting time with mortality? A harmonised meta-analysis of data from more than 1 million men and women
Stubbs 2017- Physical activity and anxiety: A perspective from the World Health Survey
Tremblay 2017- Sedentary Behavior Research Network (SBRN) – Terminology Consensus Project process and outcome
Stubbs 2017- An examination of the anxiolytic effects of exercise for people with anxiety and stress-related disorders: A meta-analysis
Harvey 2018- Exercise and the Prevention of Depression: Results of the HUNT Cohort Study
Firth 2018- Effect of aerobic exercise on hippocampal volume in humans: A systematic review and meta-analysis
Sallis 2016- Physical activity in relation to urban environments in 14 cities worldwide: a cross-sectional study
White 2017- Domain-Specific Physical Activity and Mental Health: A Meta-analysis

**Cluster 3- Physical exercise and ‘schizophrenia/mental illness’**				
3	108	0.965	2014	schizophrenia (364.88, 1.0E-4); psychosis (146.29, 1.0E-4); bipolar disorder (76.88, 1.0E-4); severe mental illness (51.72, 1.0E-4); covid-19 (51.39, 1.0E-4)

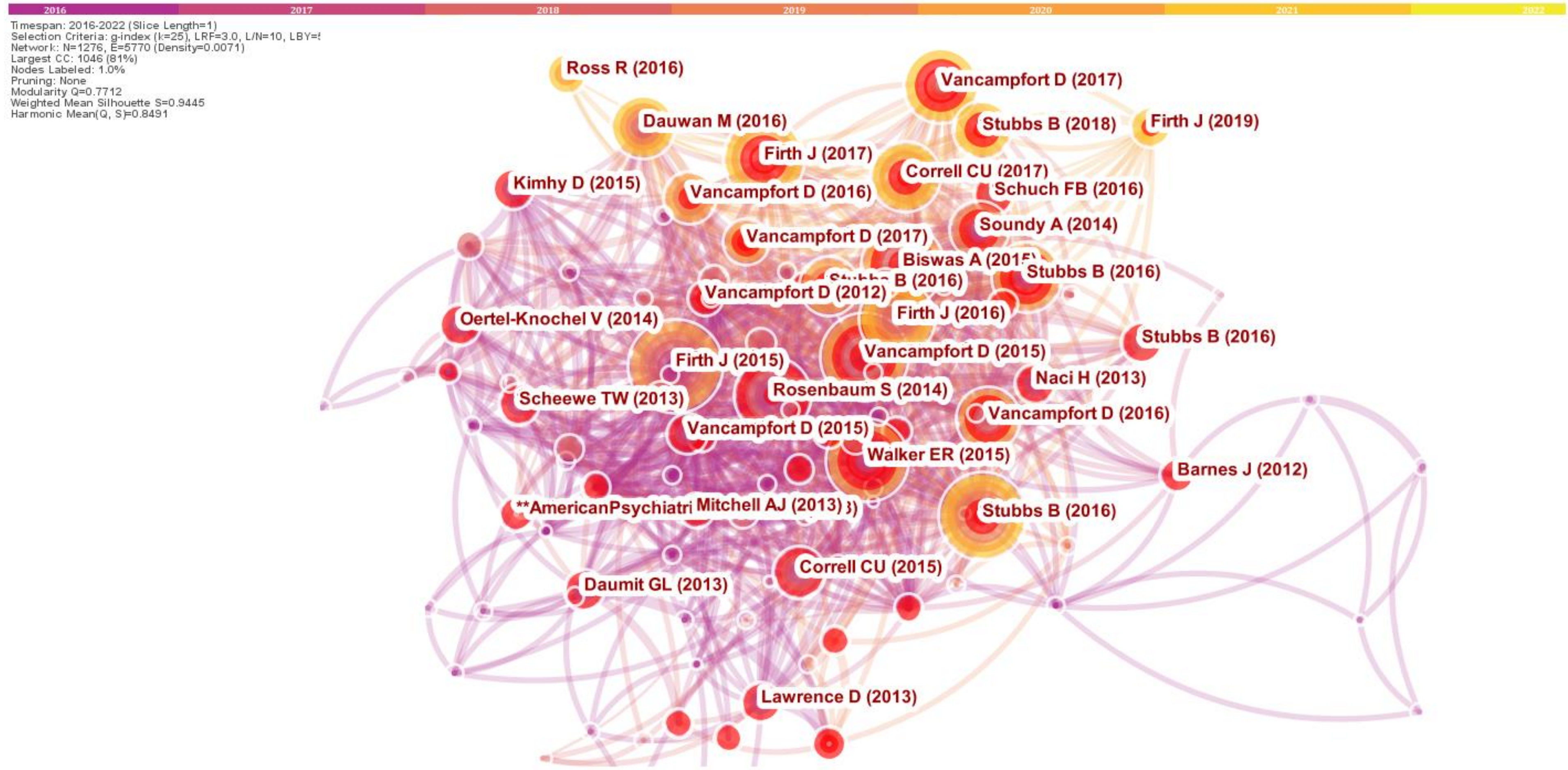

Papers with highest centrality:
Dauwan 2016- Exercise Improves Clinical Symptoms, Quality of Life, Global Functioning, and Depression in Schizophrenia: A Systematic Review and Meta-analysis
Correll 2017- Prevalence, incidence and mortality from cardiovascular disease in patients with pooled and specific severe mental illness: a large-scale meta-analysis of 3,211,768 patients and 113,383,368 controls
Stubbs 2016- How much physical activity do people with schizophrenia engage in? A systematic review, comparative meta-analysis and meta-regression
Walker 2015- Mortality in mental disorders and global disease burden implications: a systematic review and meta-analysis
Firth 2015- A systematic review and meta-analysis of exercise interventions in schizophrenia patients
Rosembaum 2014- Physical activity interventions for people with mental illness: a systematic review and meta-analysis
Dauwan 2016- Yoga as part of a package of care versus non-standard care for schizophrenia

Cluster 4- Physical exercise and 'greenness/urbanicity'

4	94	0.998	2015	green space (226.64, 1.0E-4); greenspace (88.34, 1.0E-4); greenness (85.32, 1.0E-4); exercise (73.41, 1.0E-4); green spaces (63.93, 1.0E-4)

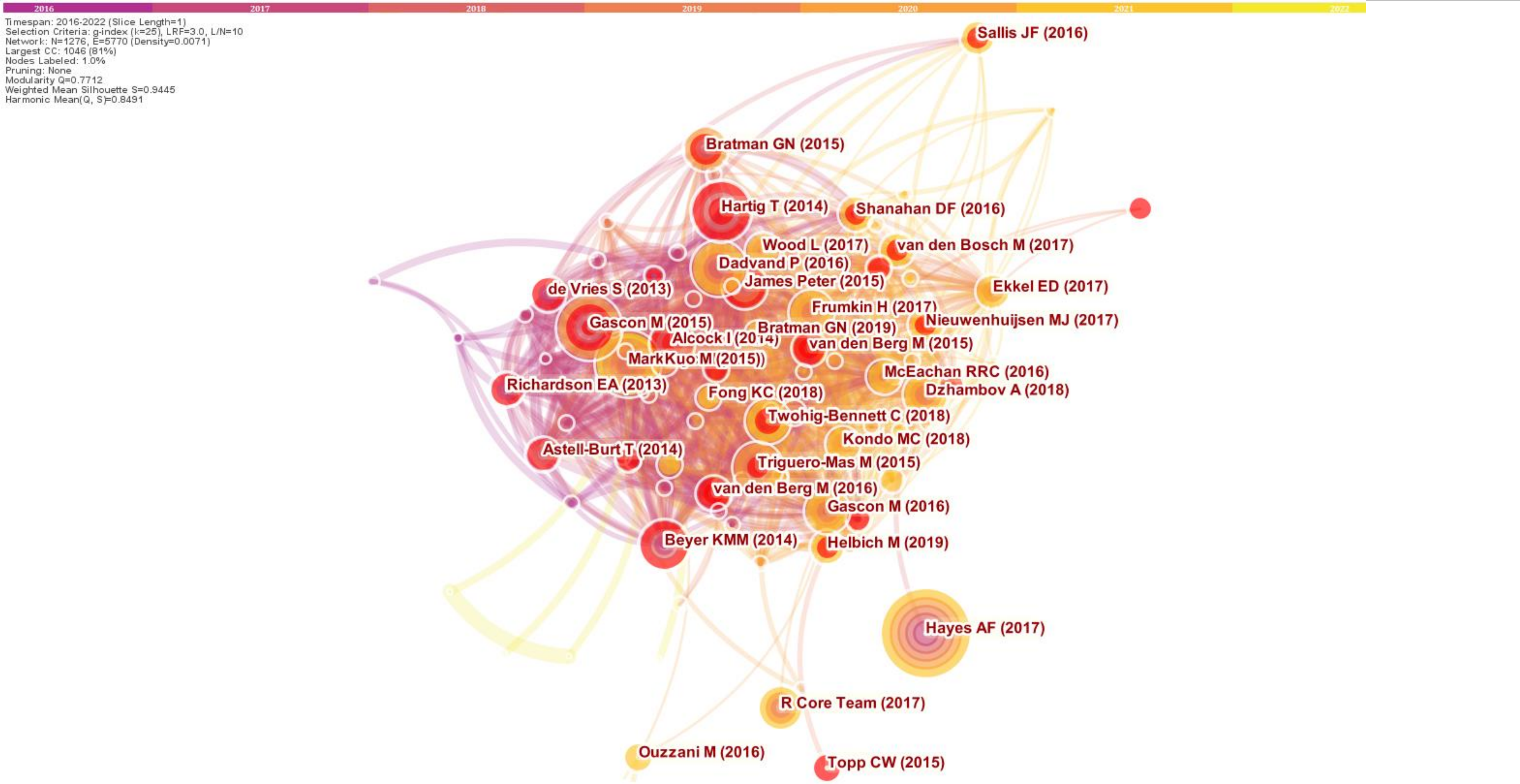

Papers with highest centrality:

Hayes 2017- Introduction to Mediation, Moderation, and Conditional Process Analysis
Beyer 2014- Exposure to neighborhood green space and mental health: evidence from the survey of the health of Wisconsin
Gascon 2015- Residential green spaces and mortality: A systematic review
Twohig-bennett 2018- The health benefits of the great outdoors: A systematic review and meta-analysis of greenspace exposure and health outcomes
Dadvand 2016- Green spaces and General Health: Roles of mental health status, social support, and physical activity
Wood 2017- Public green spaces and positive mental health - investigating the relationship between access, quantity and types of parks and mental wellbeing
James 2015- A Review of the Health Benefits of Greenness
Hartig 2014- Nature and Health
Ekkel 2017- Nearby green space and human health: Evaluating accessibility metrics

Cluster 5- Physical activity and ‘physical health’

5	84	0.941	2014	depression (119.67, 1.0E-4); multiple sclerosis (91.36, 1.0E-4); exercise (38.88, 1.0E-4); fatigue (35.32, 1.0E-4); diet (31.9, 1.0E-4)

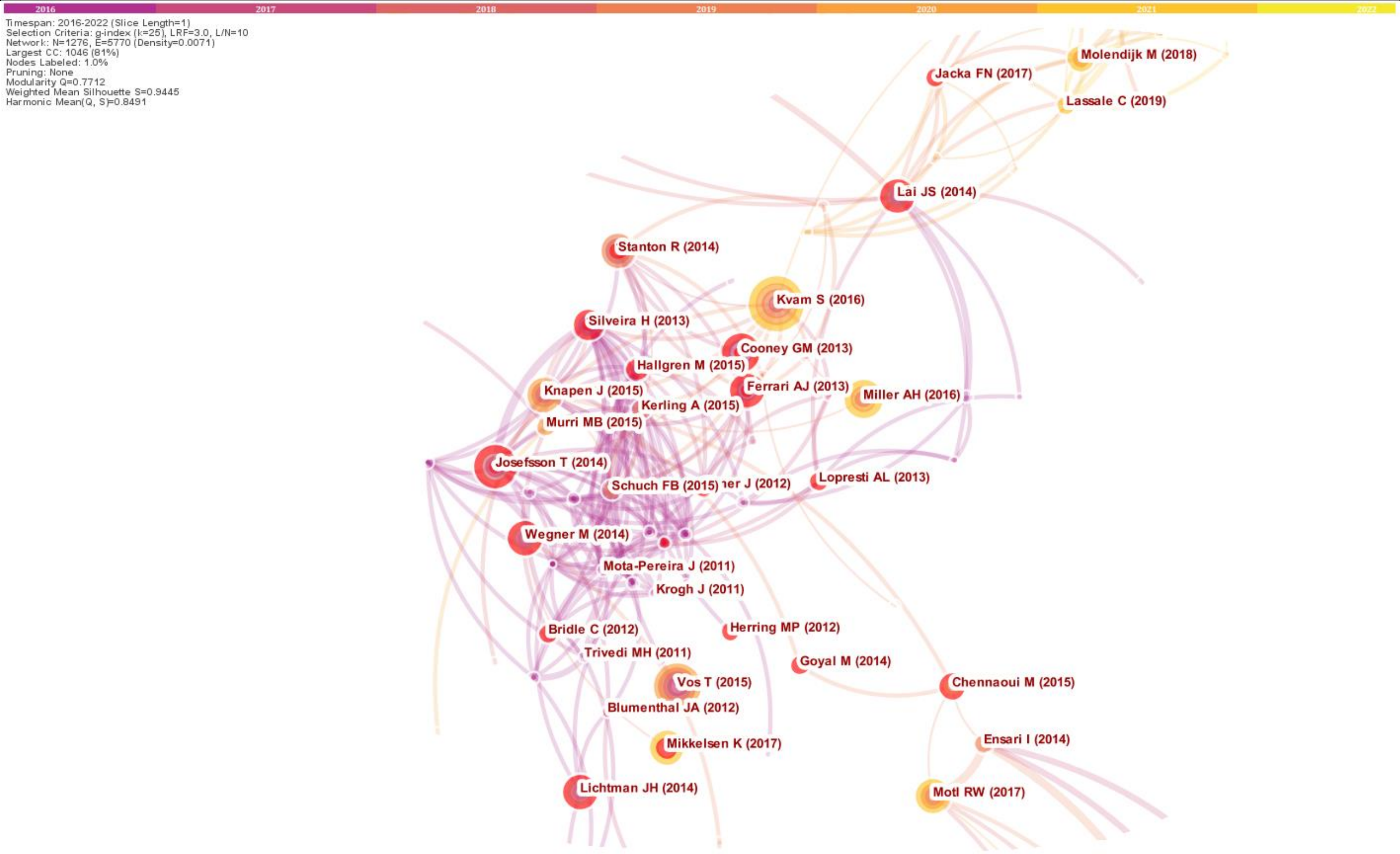

Papers with highest centrality:

Molendijk 2018- Diet quality and depression risk: a systematic review and dose-response meta-analysis of prospective studies
Kvam 2016- Exercise as a treatment for depression A meta-analysis
Lai 2014- A systematic review and meta-analysis of dietary patterns and depression in community-dwelling adults
Miller 2016- The association of physical activity, cognitive processes and automobile driving ability in older adults: A review of the literature
Knapen 2015- Exercise therapy improves both mental and physical health in patients with major depression
Josefsson 2014- Physical exercise intervention in depressive disorders: meta-analysis and systematic review
Vos 2015- Global, regional, and national incidence, prevalence, and years lived with disability for 301 acute and chronic diseases and injuries in 188 countries, 1990-2013: a systematic analysis for the Global Burden of Disease Study 2013

**Cluster 6- Physical exercise and ‘children/ adolescents’**				
6	83	0.926	2016	adolescents (124.85, 1.0E-4); children (115.61, 1.0E-4); screen time (73.97, 1.0E-4); youth (49.24, 1.0E-4); adolescent (48.61, 1.0E-4)

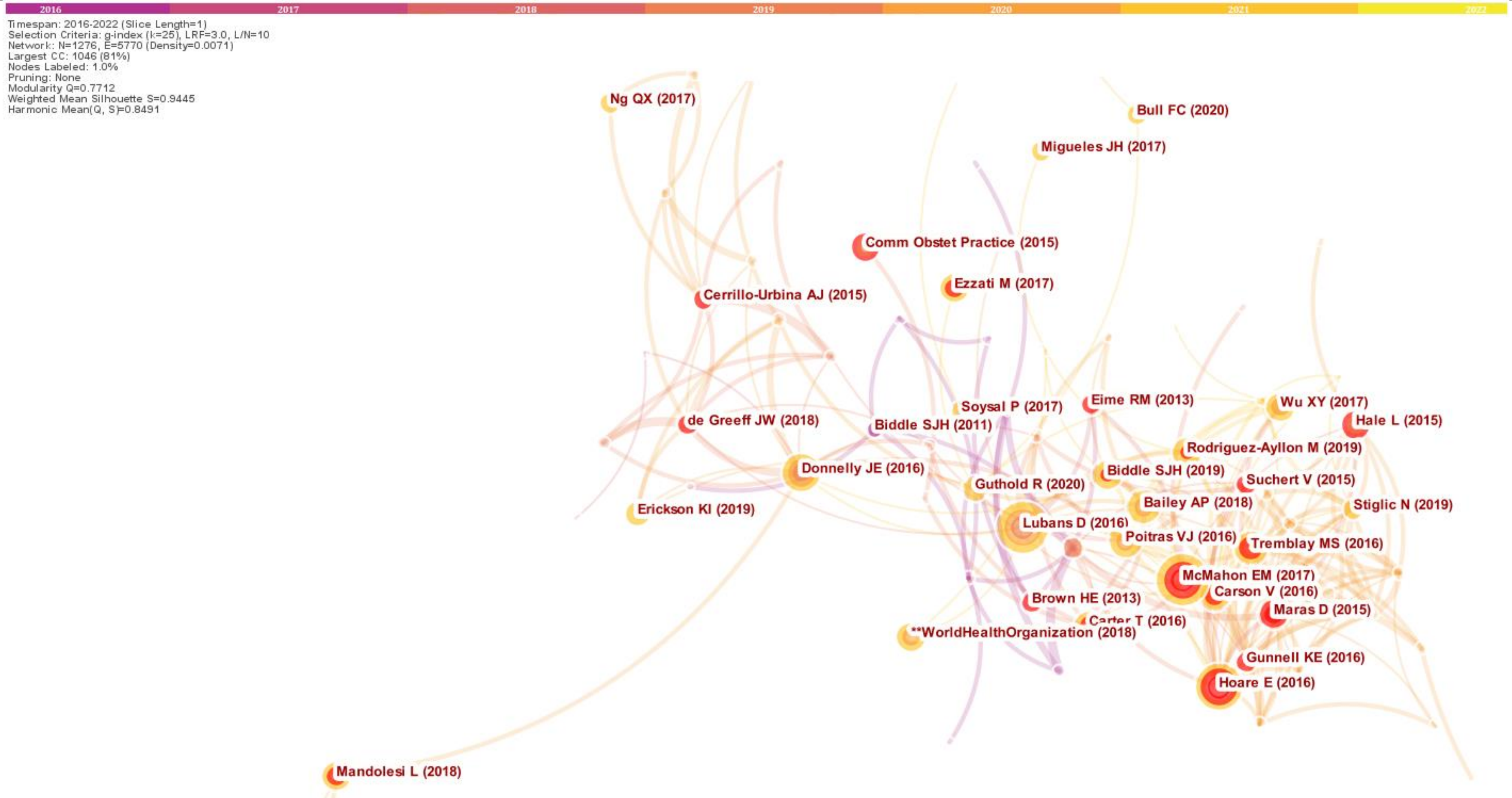

Papers with highest centrality:
Ezzati 2017- Physical Activity, Sedentary Behavior and the Risk of Overweight and Obesity in School-Aged Children
Donnelly 2016- Physical Activity, Fitness, Cognitive Function, and Academic Achievement in Children: A Systematic Review
Lubans 2016- Physical Activity for Cognitive and Mental Health in Youth: A Systematic Review of Mechanisms
Biddle 2019- Physical activity and mental health in children and adolescents: an updated review of reviews and an analysis of causality
Poitras 2016- Systematic review of the relationships between objectively measured physical activity and health indicators in school-aged children and youth
Tremblay 2016- Canadian 24-Hour Movement Guidelines for Children and Youth: An Integration of Physical Activity, Sedentary Behaviour, and Sleep
McMahon 2017- Physical activity in European adolescents and associations with anxiety, depression and well-being
Maras 2015- Screen time is associated with depression and anxiety in Canadian youth
Hoare 2016- The associations between sedentary behaviour and mental health among adolescents: a systematic review

**Cluster 7- Physical activity and ‘concussion/chronic traumatic encephalopathy’**				
7	75	0.986	2015	concussion (237.2, 1.0E-4); chronic traumatic encephalopathy (136.43, 1.0E-4); traumatic brain injury (92.6, 1.0E-4); physical activity (85.94, 1.0E-4); mild traumatic brain injury (81.67, 1.0E-4)

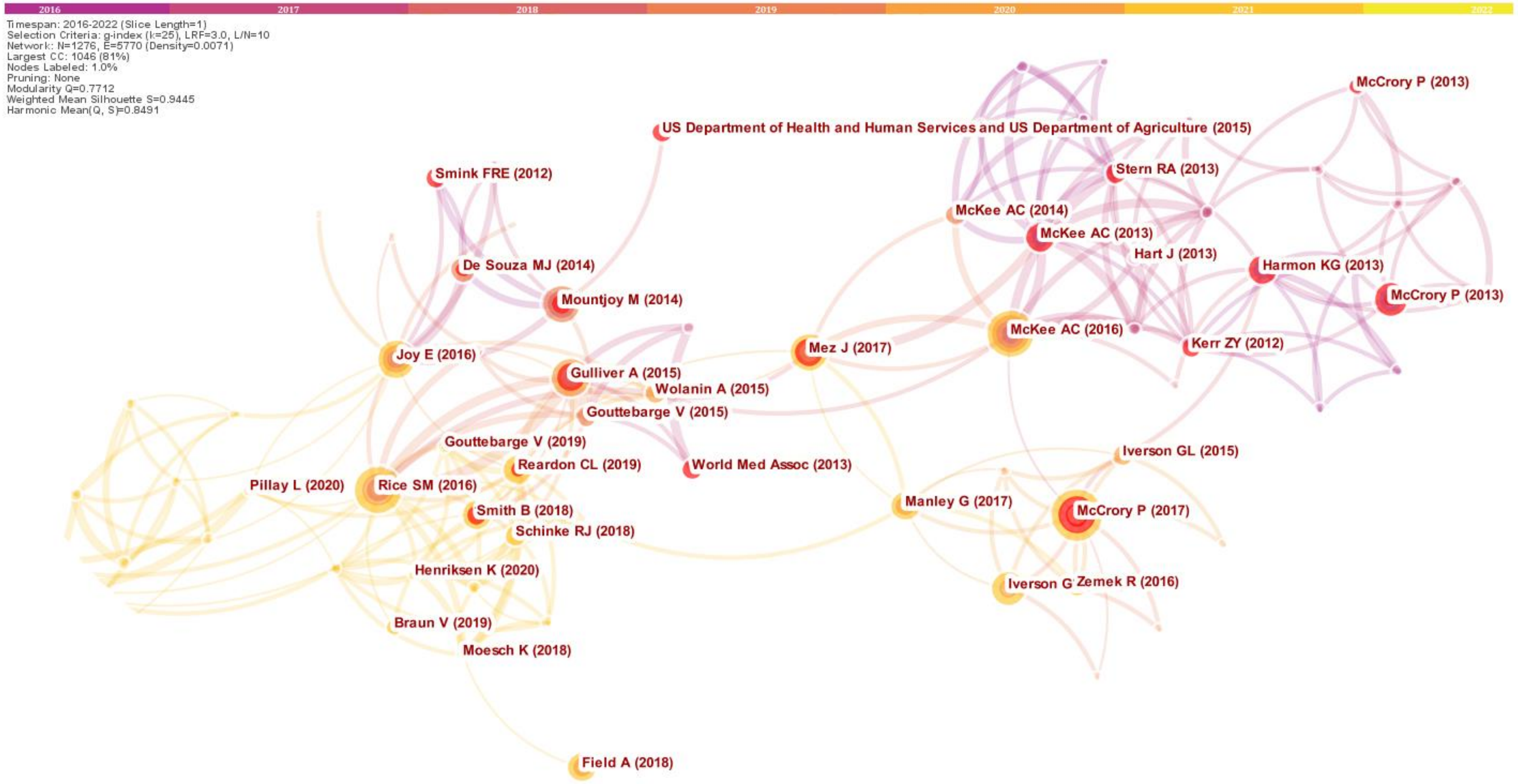

Papers with highest centrality:

Rice 2016- The Mental Health of Elite Athletes: A Narrative Systematic Review

Reardron 2019- Mental health in elite athletes: International Olympic Committee consensus statement (2019)

Mountjoy 2014- The IOC consensus statement: beyond the Female Athlete Triad—Relative Energy Deficiency in Sport (RED-S)

Mez 2017- Clinicopathological Evaluation of Chronic Traumatic Encephalopathy in Players of American Football

Mccrory 2017- Consensus statement on concussion in sport—the 5th international conference on concussion in sport held in Berlin, October 2016

Mckee 2013- The spectrum of disease in chronic traumatic encephalopathy

**Cluster 8- Physical exercise and 'NAFLD'**				
8	28	1	2015	non-alcoholic fatty liver disease (186.64, 1.0E-4); nafld (103.21, 1.0E-4); non-alcoholic steatohepatitis (75.57, 1.0E-4); depression (37.12, 1.0E-4); nonalcoholic steatohepatitis (34.27, 1.0E-4)

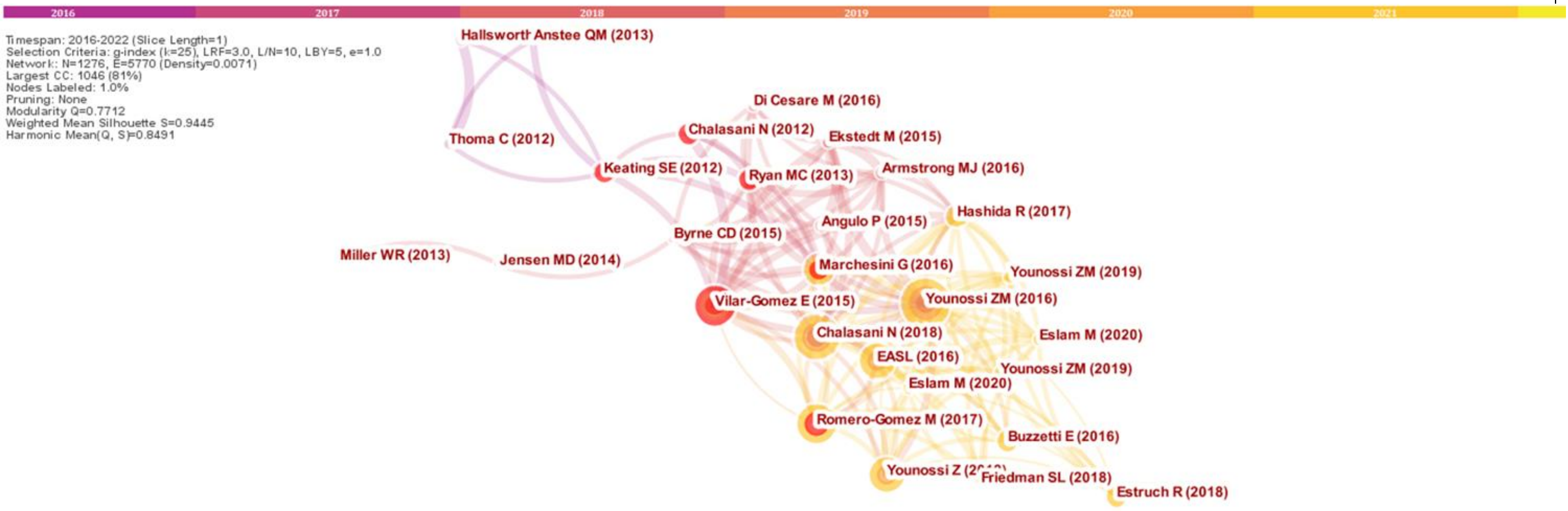

Papers with highest centrality:

Keating 2012- Exercise and non-alcoholic fatty liver disease: a systematic review and meta-analysis

Chalasani 2012- The diagnosis and management of non-alcoholic fatty liver disease: Practice Guideline by the American Association for the Study of Liver Diseases, American College of Gastroenterology, and the American Gastroenterological Association

Vilar-gomez 2015- Weight Loss Through Lifestyle Modification Significantly Reduces Features of Nonalcoholic Steatohepatitis

Chalasani 2018- The diagnosis and management of nonalcoholic fatty liver disease: Practice guidance from the American Association for the Study of Liver Diseases

Romero-gomez 2017- Treatment of NAFLD with diet, physical activity and exercise

Younossi 2016- Global epidemiology of nonalcoholic fatty liver disease-Meta-analytic assessment of prevalence, incidence, and outcomes

Buzzetti E 2016- The multiple-hit pathogenesis of non-alcoholic fatty liver disease (NAFLD)

**Cluster 9- Physical exercise and 'frailty'**				
9	15	0.991	2014	frailty (115.23, 1.0E-4); cognitive frailty (67.29, 1.0E-4); disability (45.7, 1.0E-4); physical frailty (37.31, 1.0E-4); mobility (22.37, 1.0E-4)

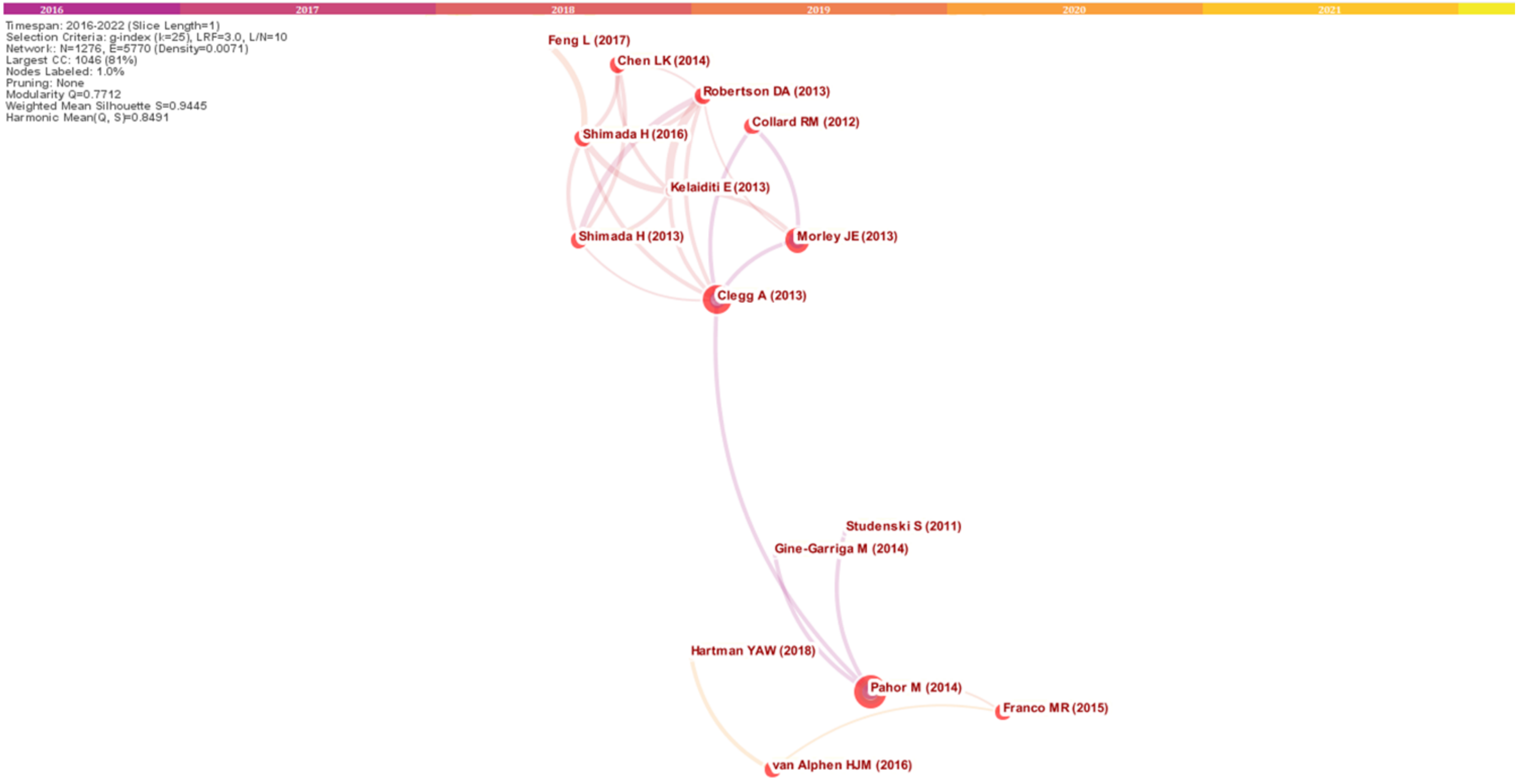

Papers with highest centrality:
Chen 2014- Sarcopenia in Asia: Consensus report of the Asian working group for sarcopenia
Clegg 2013- Frailty in eldery people
Pahor 2014 - Effect of Structured Physical Activity on Prevention of Major Mobility Disability in Older Adults The LIFE Study Randomized Clinical Trial
Morley 2013- Frailty consensus: a call to action

Cluster 11- Physical exercise and 'behaviour change'

11	10	0.997	2013	behaviour change (33.85, 1.0E-4); sleep wake disorders [therapy] (23.54, 1.0E-4); therapy] (23.54, 1.0E-4); depression [therapy] (23.54, 1.0E-4); cognitive therapy (23.54, 1.0E-4)

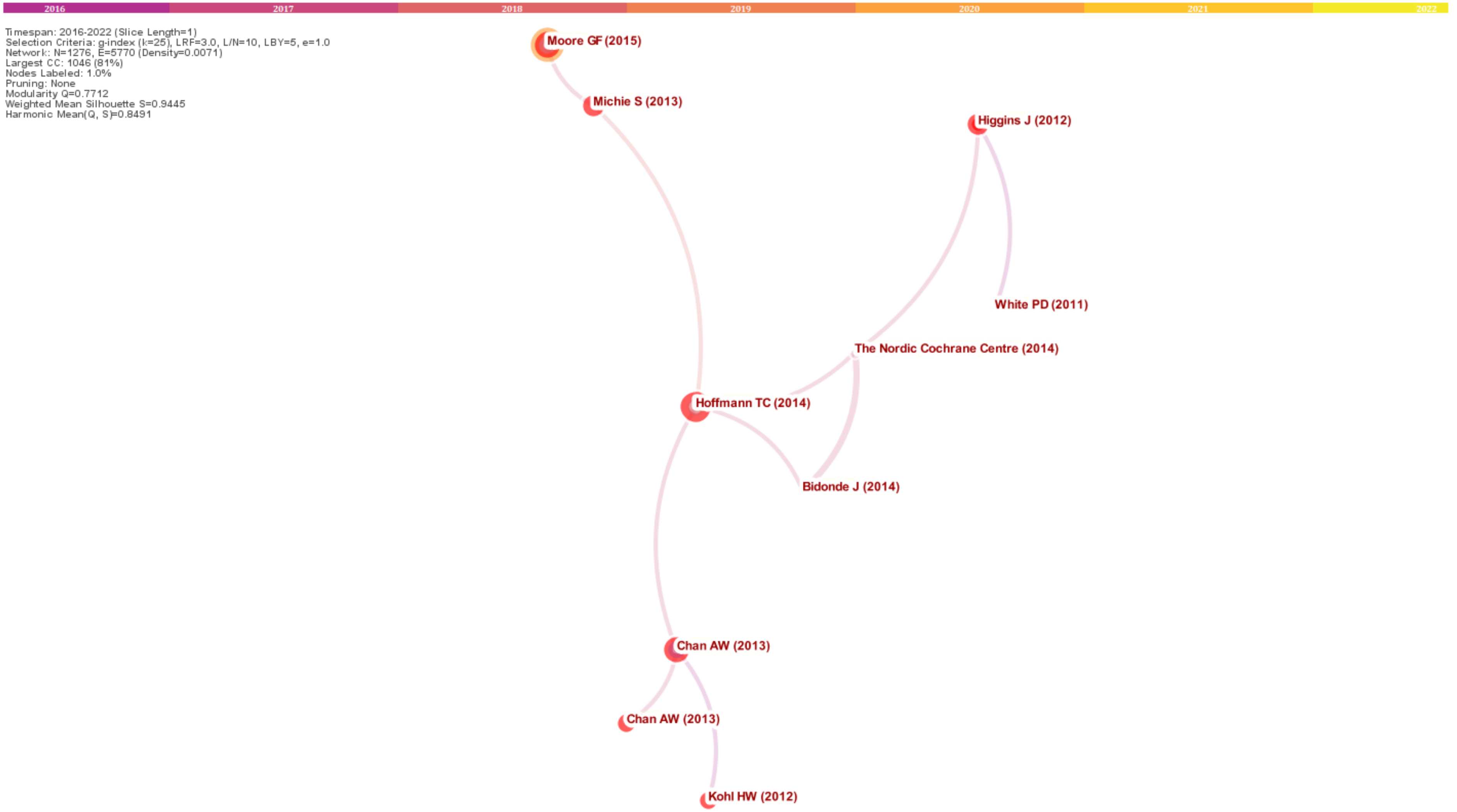

Papers with highest centrality:
Moore 2015- Process evaluation of complex interventions
Michie 2013- The Behavior Change Technique Taxonomy (v1) of 93 Hierarchically Clustered Techniques: Building an International Consensus for the Reporting of Behavior Change Interventions
Hoffmann TC 2014- Shared decision making: what do clinicians need to know and why should they bother?
Chan Aw 2013- Guidance for protocols of clinical trials
Higgins 2012- Consistency and inconsistency in network meta-analysis: concepts and models for multi-arm studies

Cluster 14- Physical exercise and 'Cardio-Metabolic Health Markers'

14	6	0.991	2014	isotemporal substitution (34.86, 1.0E-4); accelerometer (12.08, 0.001); time use (11.56, 0.001); regression (11.56, 0.001); lifestyle activity (11.56, 0.001)

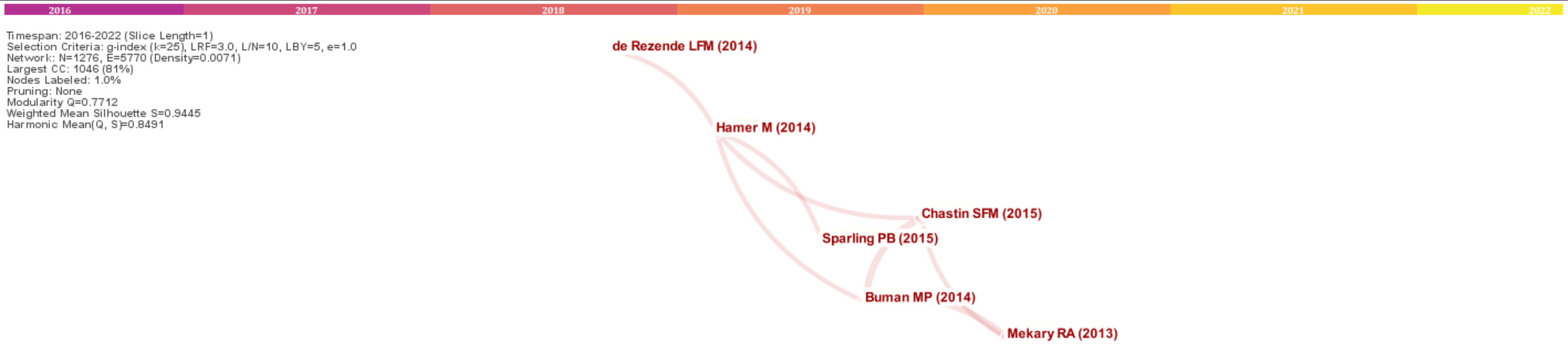

Papers with highest centrality:

Hamer 2014- Taking up physical activity in later life and healthy ageing

Chastin 2015- Combined Effects of Time Spent in Physical Activity, Sedentary Behaviors and Sleep on Obesity and Cardio-Metabolic Health Markers: A Novel Compositional Data Analysis Approach

Buman MP 2014- Reallocating Time to Sleep, Sedentary Behaviors, or Active Behaviors: Associations With Cardiovascular Disease Risk Biomarkers, NHANES 2005–2006

Cluster 19 - Cluster 11- Evidence-based protocols -Non relevant cluster

19	4	0.996	2018	patient-and family-centred care (pfcc) (10.1, 0.005); healthy eating behaviour (10.1, 0.005); preventive practices (10.1, 0.005); multi-methods (10.1, 0.005); consumer participation (10.1, 0.005)

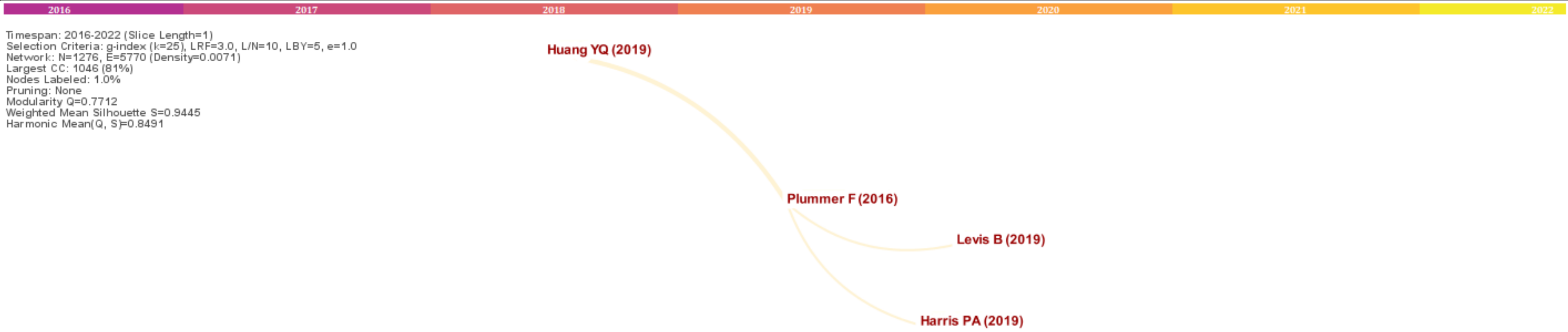

Papers with highest centrality:

Plummer 2016- Screening for anxiety disorders with the GAD-7 and GAD-2: a systematic review and diagnostic metaanalysis

Harris 2019- The REDCap consortium: Building an international community of software platform partners

Levis 2019- Accuracy of Patient Health Questionnaire-9 (PHQ-9) for screening to detect major depression: Individual participant data meta-analysis

Cluster 20- Physical exercise and 'post-covid-19/long covid'

20	4	1	2019	post-covid-19 syndrome (19.27, 1.0E-4); long covid (19.27, 1.0E-4); pasc (19.27, 1.0E-4); immunity (13.76, 0.001); autoimmunity (9.62, 0.005)

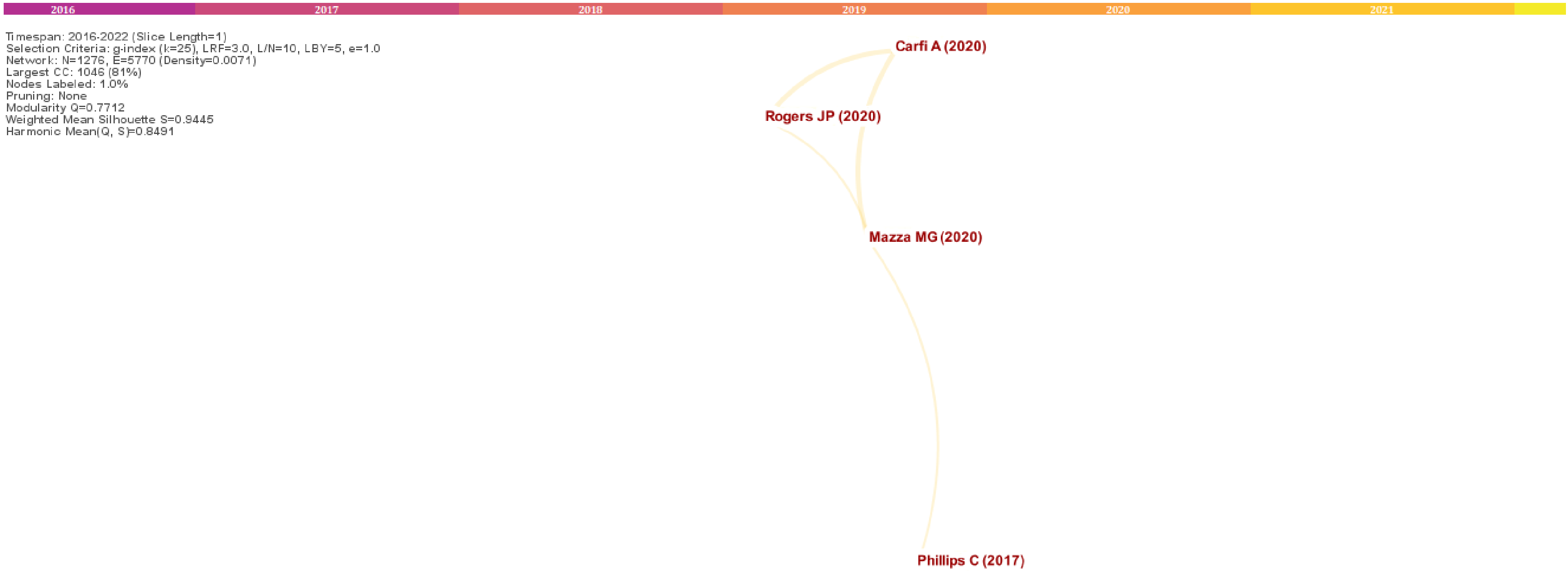

Papers with highest centrality:
Carfi 2020- Persistent Symptoms in Patients After Acute COVID-19
Rogers 2020- Psychiatric and neuropsychiatric presentations associated with severe coronavirus infections: a systematic review and meta-analysis with comparison to the COVID-19 pandemic
Mazza 2020- Anxiety and depression in COVID-19 survivors
Philips 2017- Brain-derived neurotrophic factor, depression, and physical activity: Making the neuroplastic connection.

C. Detail of reference co-citation network clusters (2021)

Co-citation reference with highlight of burstness (2021)

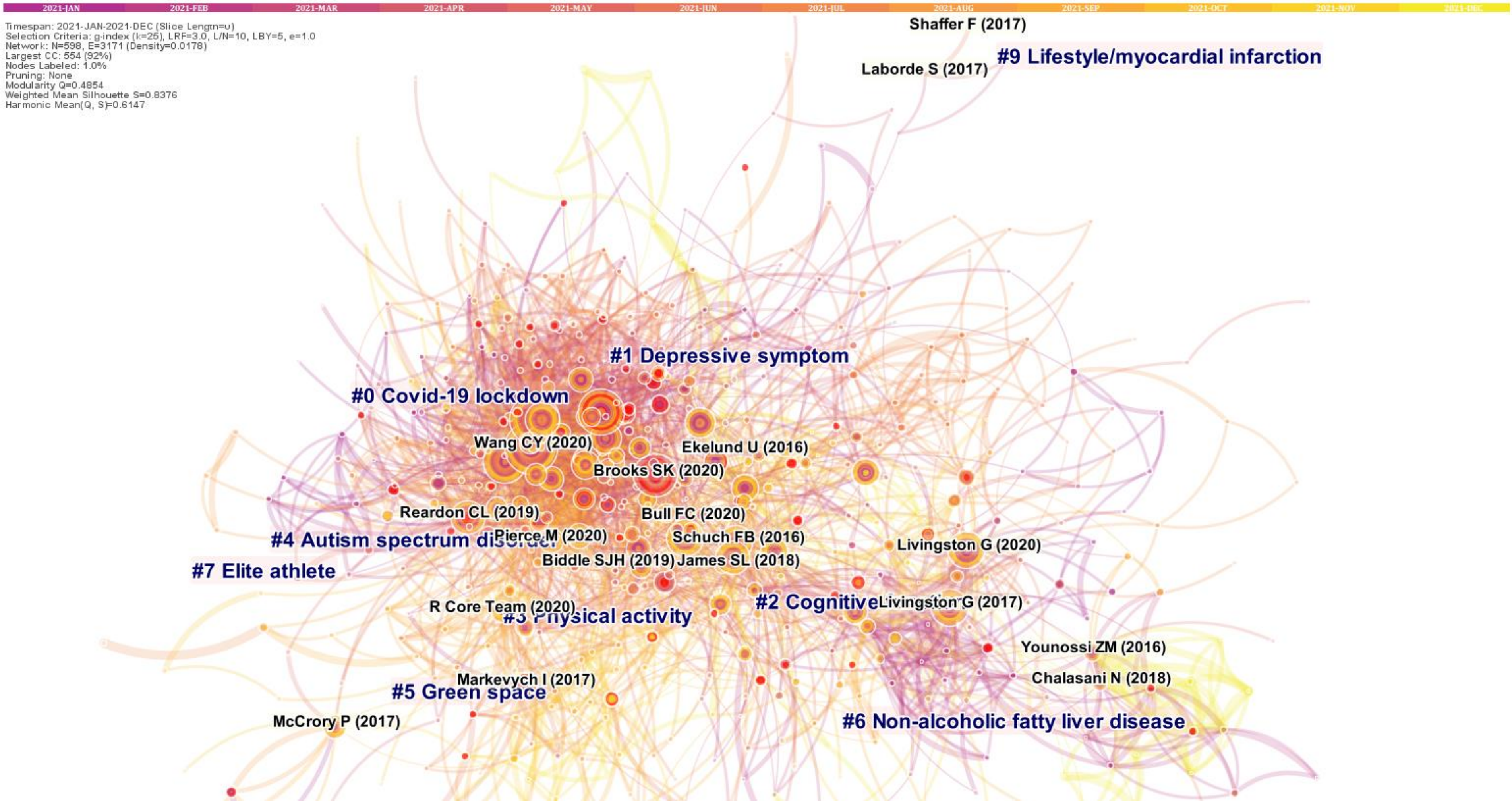

Co-citation reference network with detail of clusters (2021)

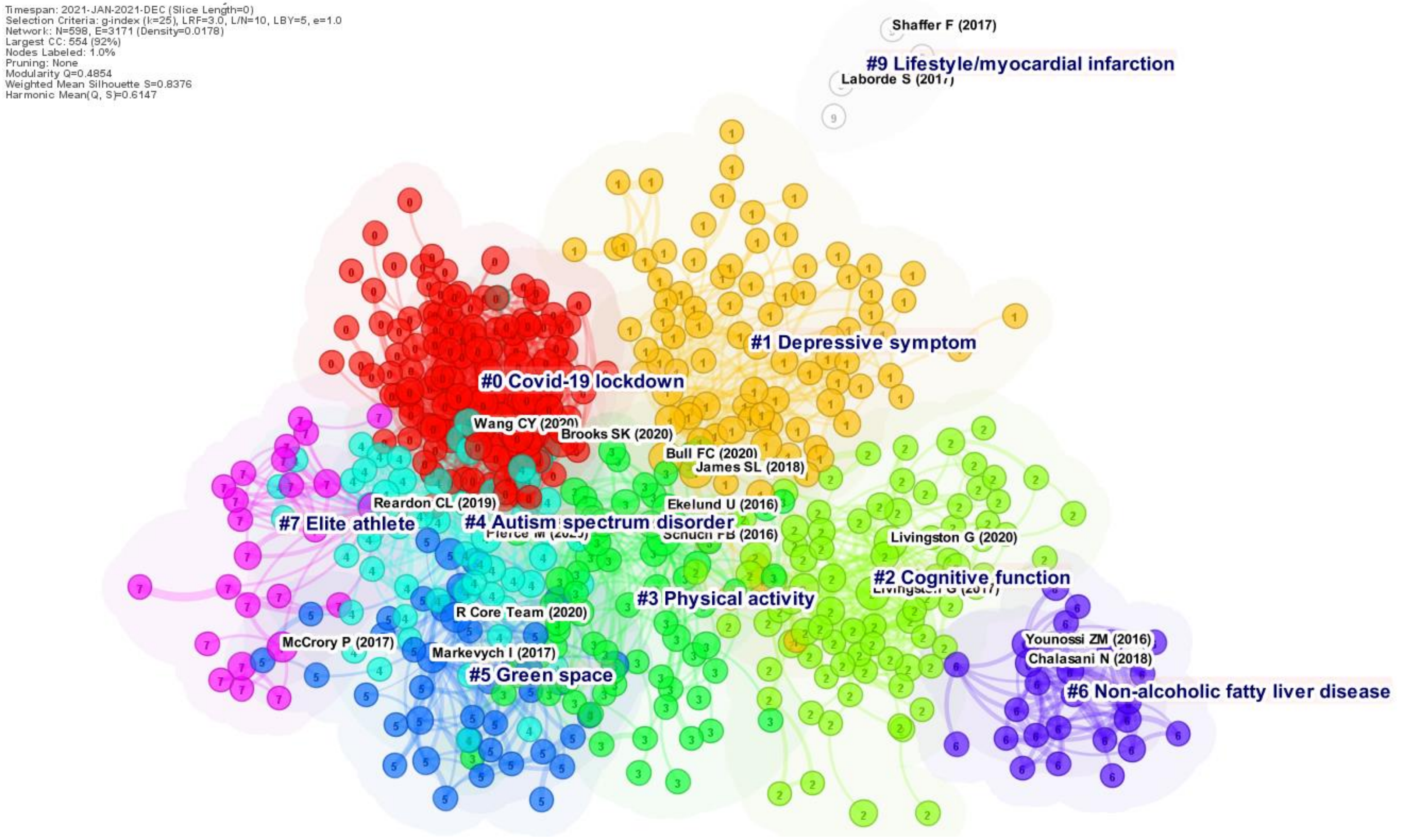

Co-citation reference network time map (2016-2021)

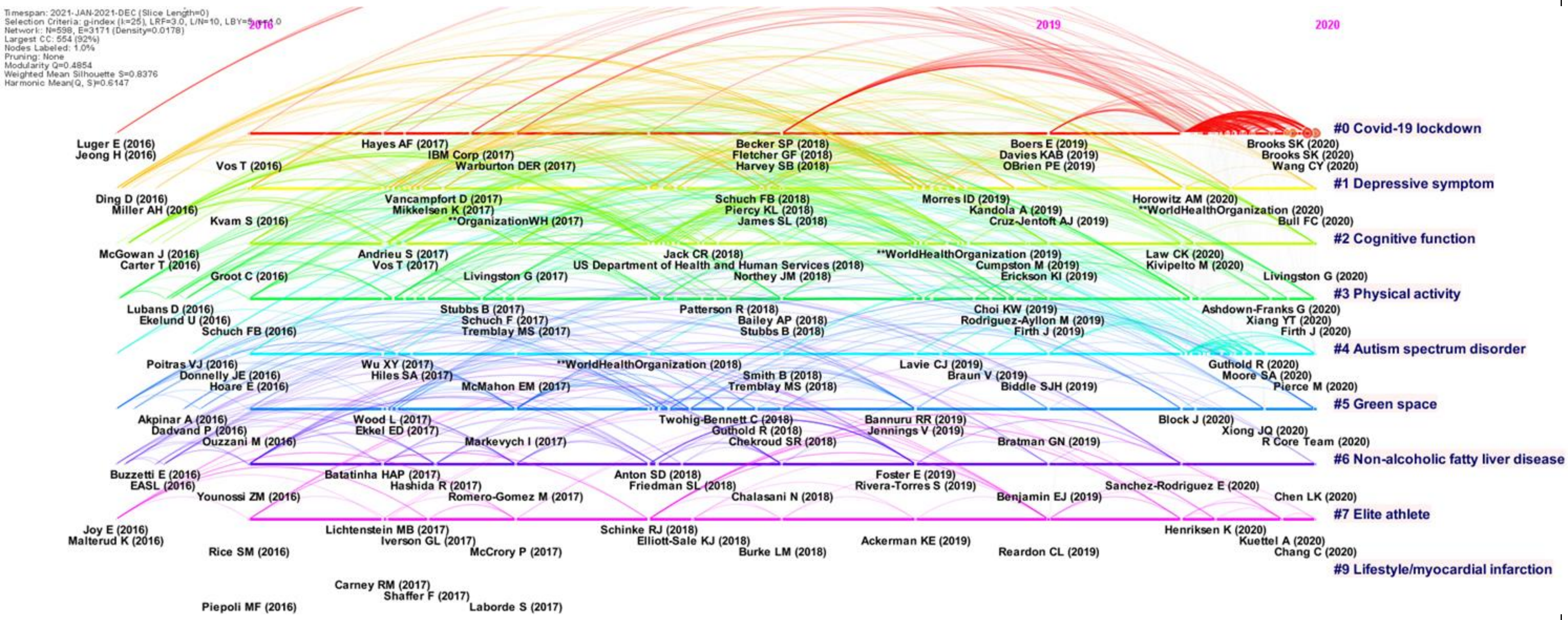

**Cluster 0- Physical exercise and 'covid'**					
0	147	0.818	2019	covid-19 pandemic; physical activity; covid-19 lockdown; cross-sectional study; physical health | mental health; covid-19 outbreak; psychological distress; physical activity level; lifestyle habit	**covid-19 lockdown** (2021.94, 1.0E-4); covid-19 pandemic (1360.83, 1.0E-4); systematic review (1125.59, 1.0E-4); covid-19 outbreak (652.86, 1.0E-4); controlled trial (634.46, 1.0E-4)

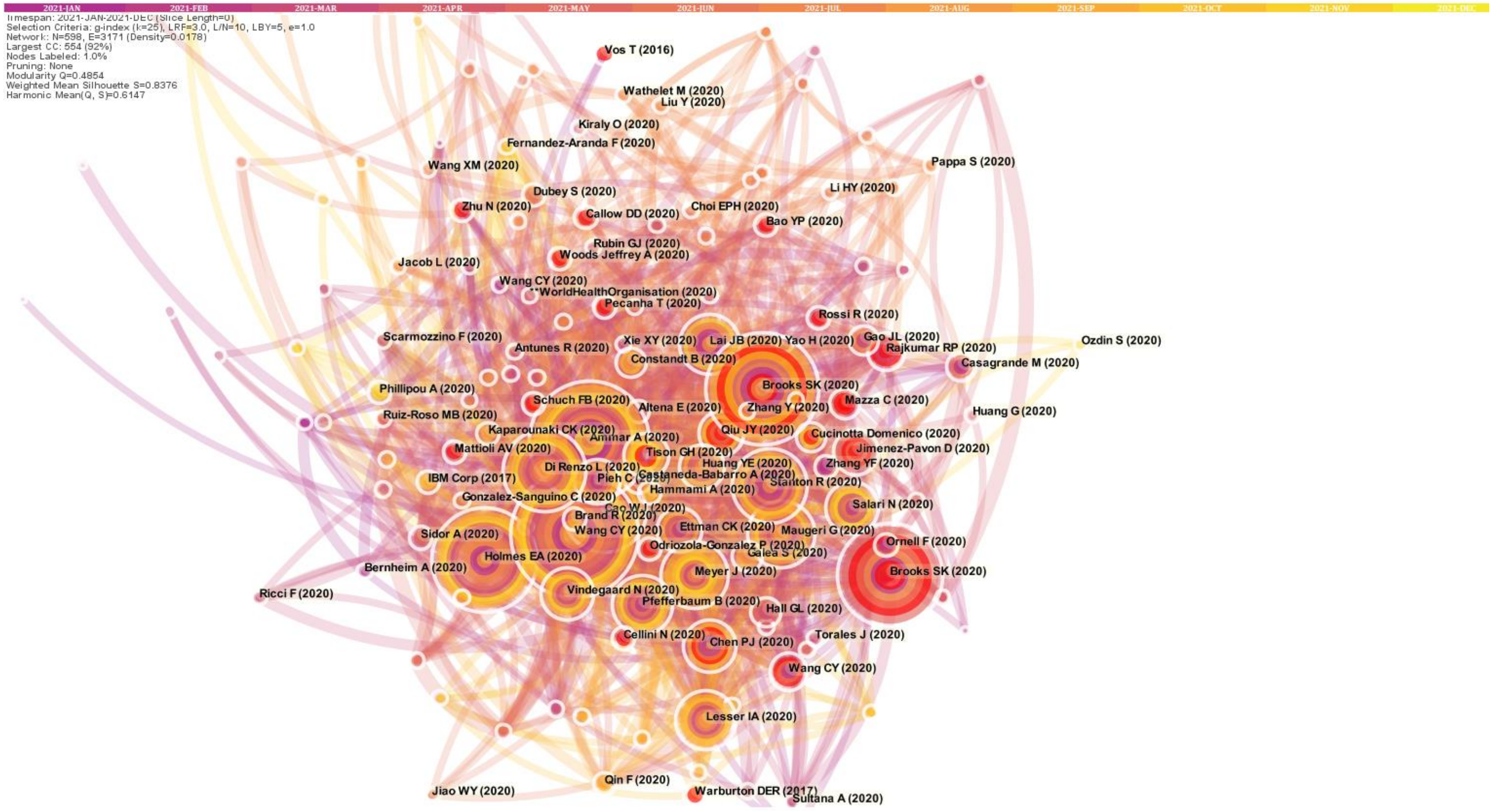

Papers with highest centrality:

Brooks 2020- The psychological impact of quarantine and how to reduce it: rapid review of the evidence
Holmes 2020- Multidisciplinary research priorities for the COVID-19 pandemic: a call for action for mental health science
Wang 2020- A Preventive Role of Exercise Across the Coronavirus 2 (SARS-CoV-2) Pandemic
Ammar 2020- Effects of COVID-19 Home Confinement on Eating Behaviour and Physical Activity: Results of the ECLB-COVID19 International Online Survey
Meyer 2020- Changes in Physical Activity and Sedentary Behavior in Response to COVID-19 and Their Associations with Mental Health in 3052 US Adults
Salari 2020- The prevalence of stress, anxiety and depression within front-line healthcare workers caring for COVID-19 patients: a systematic review and meta-regression
Starton 2020- Depression, Anxiety and Stress during COVID-19: Associations with Changes in Physical Activity, Sleep, Tobacco and Alcohol Use in Australian Adults
Brooks 2020- A Systematic, Thematic Review of Social and Occupational Factors Associated With Psychological Outcomes in Healthcare Employees During an Infectious Disease Outbreak
Lai 2020- Factors Associated With Mental Health Outcomes Among Health Care Workers Exposed to Coronavirus Disease 2019

Cluster 1- Physical exercise and ‘evidence synthesis/depression’					
1	91	0.835	2017	depressive symptom; physical activity; systematic review; mental health; multiple sclerosis | controlled trial; aerobic exercise; anxiety symptom; pilot study; cross-sectional study	depressive symptom (1469.23, 1.0E-4); covid-19 pandemic (908.41, 1.0E-4); multiple sclerosis (797.93, 1.0E-4); covid-19 lockdown (627.48, 1.0E-4); beneficial effect (468.83, 1.0E-4)

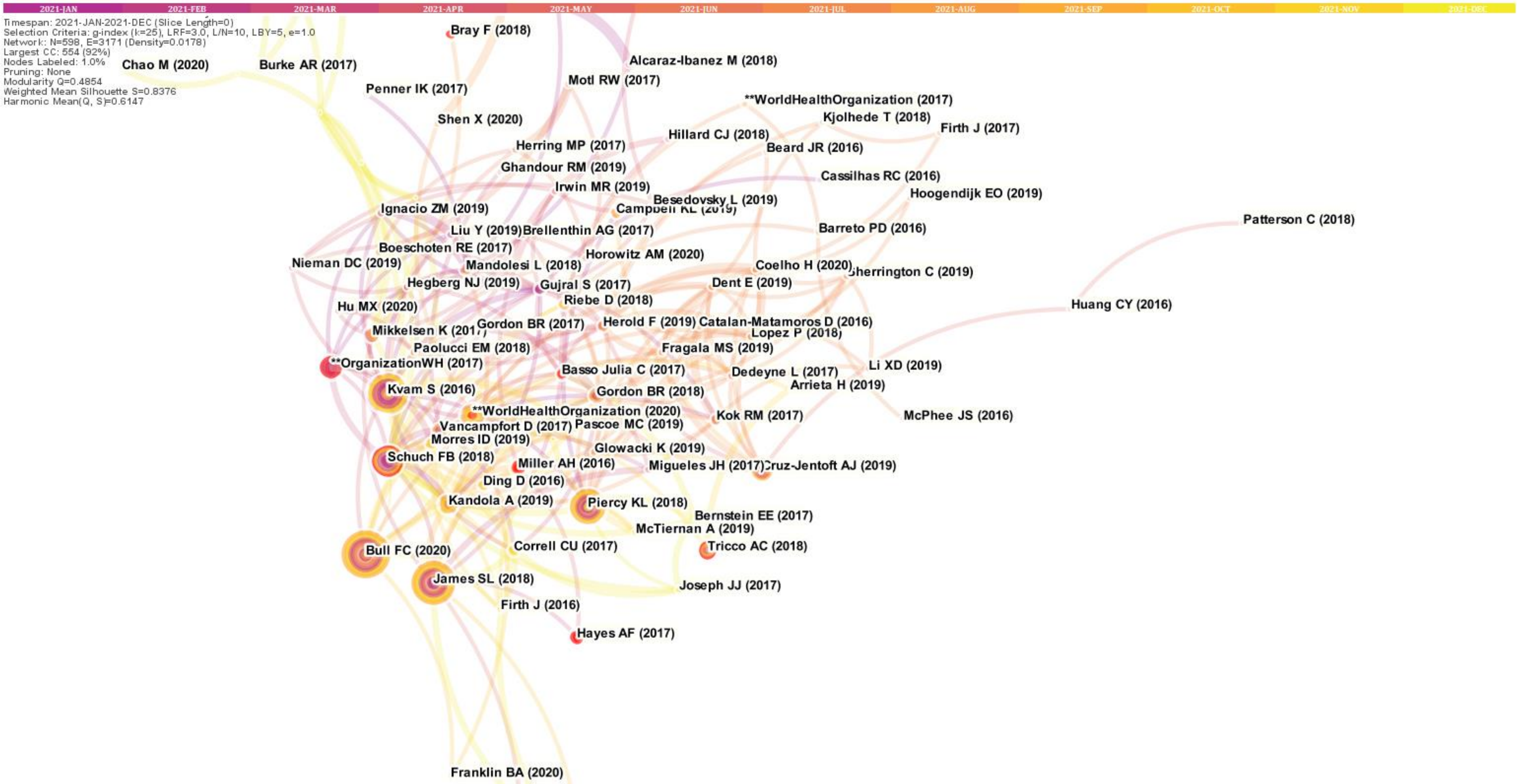

Papers with highest centrality:
Bull 2020- World Health Organization 2020 guidelines on physical activity and sedentary behaviour
Tricco 2018- PRISMA Extension for Scoping Reviews (PRISMA-ScR): Checklist and Explanation
Piercy 2017- The Physical Activity Guidelines for Americans
Schuch 2018- Physical Activity and Incident Depression: A Meta-Analysis of Prospective Cohort Studies
Kvam 2016- Exercise as a treatment for depression: A meta-analysis
Cruz-Jentoft 2019- Sarcopenia: revised European consensus on definition and diagnosis
Gordon 2018- Measurements of physical health and functional capacity

**Cluster 2- Physical exercise and 'dementia'**					
2	86	0.879	2017	systematic review; physical activity; controlled trial; cognitive function; cognitive decline | sex difference; aerobic exercise; eeg marker; physical activities; manchester consensus	covid-19 pandemic (1274.49, 1.0E-4); cognitive decline (676.83, 1.0E-4); dementia risk reduction (630.02, 1.0E-4); cognitive training (606.61, 1.0E-4); cognitive function (592.66, 1.0E-4)

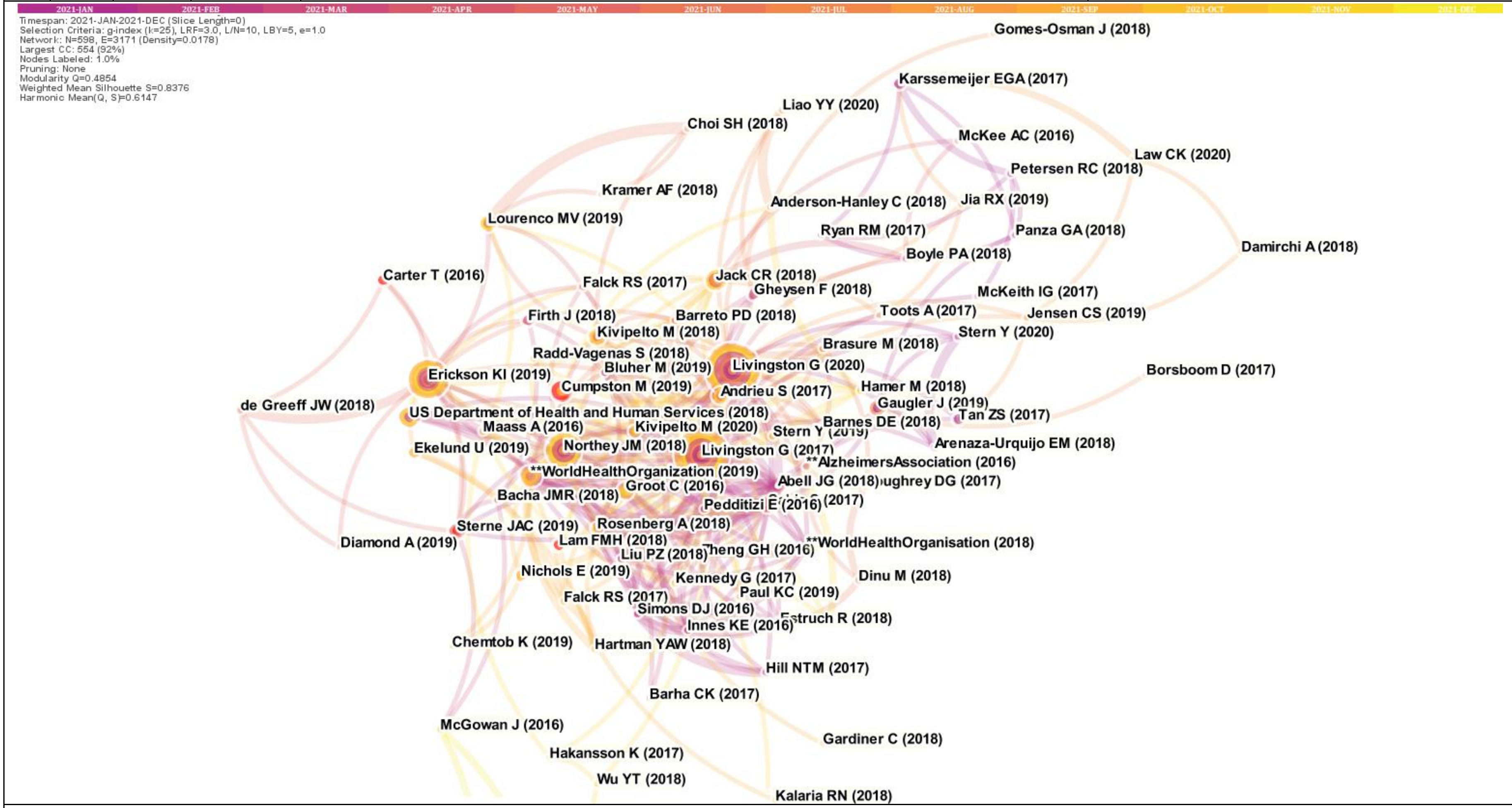

Papers with highest centrality:
Erickson 2019- Physical Activity, Cognition, and Brain Outcomes: A Review of the 2018 Physical Activity Guidelines
Jack 2018- NIA-AA Research Framework: Toward a biological definition of Alzheimer's disease
Cumpston 2018- Updated guidance for trusted systematic reviews: a new edition of the Cochrane Handbook for Systematic Reviews of Interventions
Andrieu 2017- Effect of long-term omega 3 polyunsaturated fatty acid supplementation with or without multidomain intervention on cognitive function in elderly adults with memory complaints (MAPT): a randomised, placebo-controlled trial
Livinsgston 2017- Dementia prevention, intervention, and care
Northey 2016- Exercise interventions for cognitive function in adults older than 50: a systematic review with meta-analysis
Groot 2016- The effect of physical activity on cognitive function in patients with dementia: A meta-analysis of randomized control trials

**Cluster 3- Physical exercise and 'prevention'**					
3	75	0.722	2018	physical activity; systematic review; covid-19 pandemic; mental health; controlled trial | group-based physical activity; integrating physical activity; mental health service; mental health treatment; campus environment	parkinsons disease (452.03, 1.0E-4); covid-19 lockdown (437.27, 1.0E-4); physical fitness (423.83, 1.0E-4); major depressive disorder (285.89, 1.0E-4); replacing sedentary behaviour (284.55, 1.0E-4)

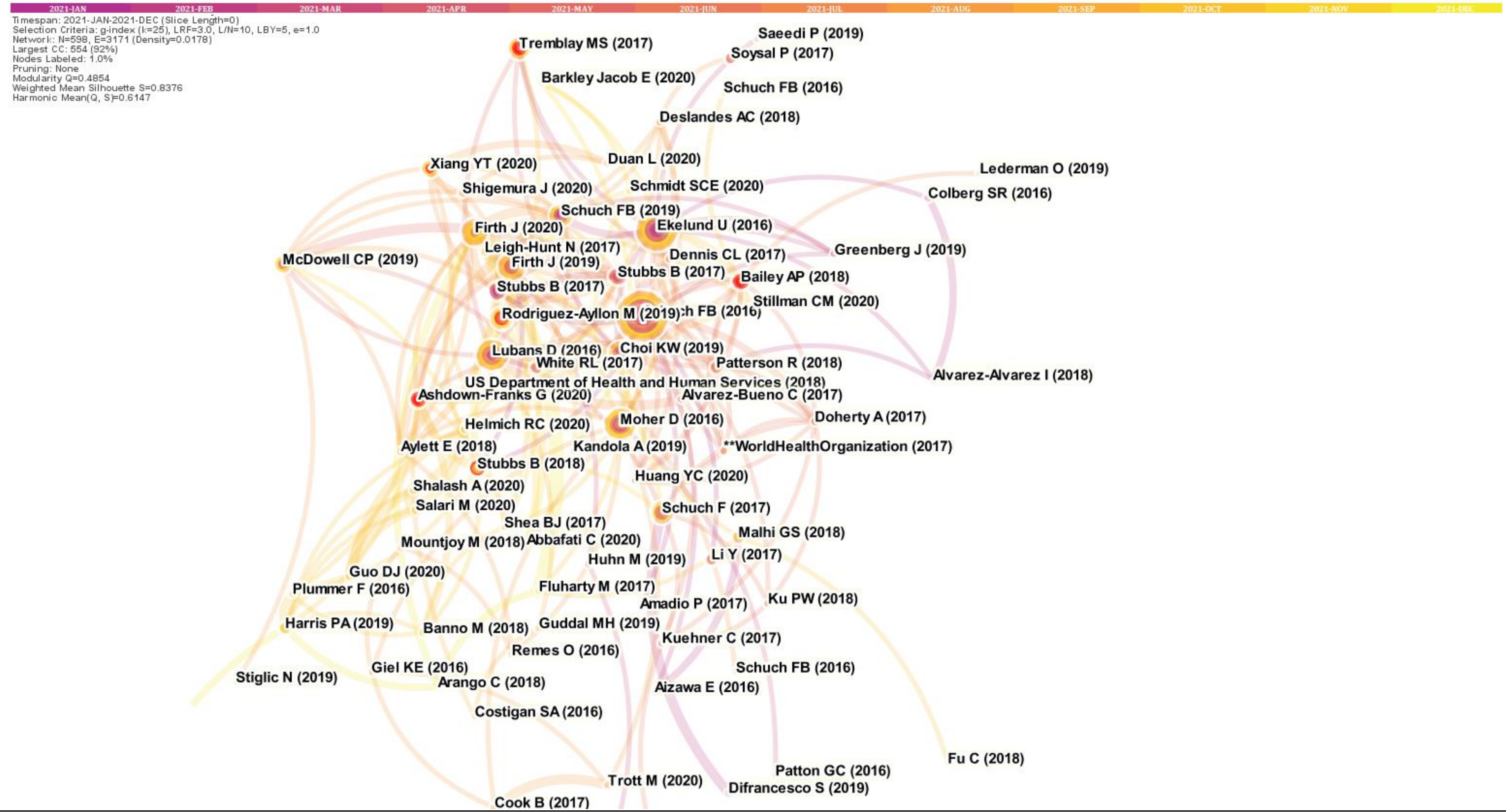

Papers with highest centrality:

Ekelund 2016- Does physical activity attenuate, or even eliminate, the detrimental association of sitting time with mortality? A harmonised meta-analysis of data from more than 1 million men and women
Firth 2020- A meta-review of "lifestyle psychiatry": the role of exercise, smoking, diet and sleep in the prevention and treatment of mental disorders
Stubbs 2017- An examination of the anxiolytic effects of exercise for people with anxiety and stress-related disorders: A meta-analysis
Ashdown-franks 2020- Predictors of physical activity recording in routine mental healthcare
Choi 2019- Assessment of Bidirectional Relationships Between Physical Activity and Depression Among Adults
Schuch 2017- Physical activity and sedentary behavior in people with major depressive disorder: A systematic review and meta-analysis
Harris 2019- Effects of mental fatigue on exercise decision-making

Cluster 4- Physical exercise and 'covid/children'					
4	59	0.837	2019	covid-19 pandemic; mental health; physical activity; scoping review; health-related quality | autism spectrum disorder; mental health outcome; covid-19 lockdown; qualitative study; depressive symptom	covid-19 pandemic (881.51, 1.0E-4); autism spectrum disorder (423.49, 1.0E-4); health-related quality (312.46, 1.0E-4); motorik-modul study (265.71, 1.0E-4); mental health problem (260.83, 1.0E-4)

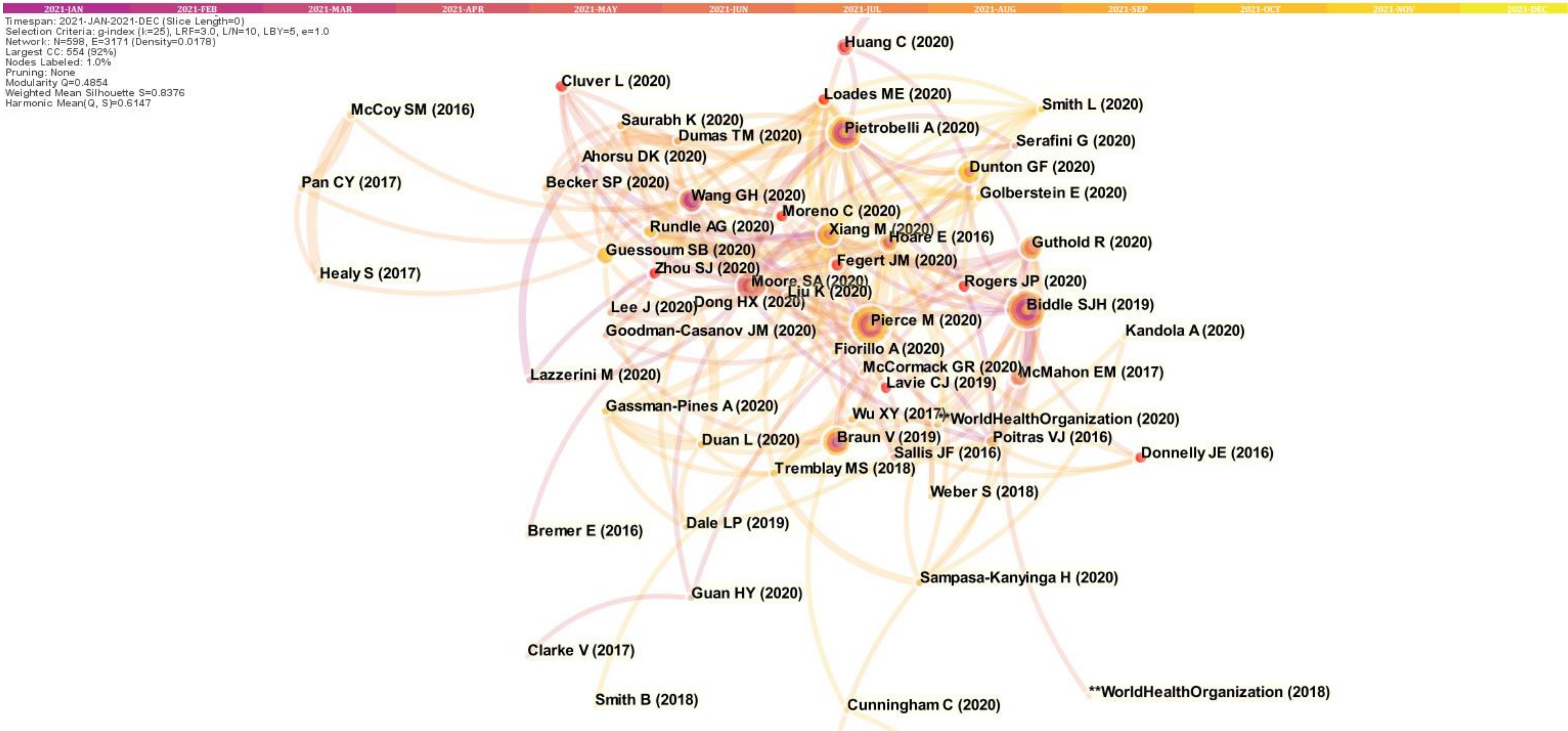

Papers with highest centrality:

Pietrobelli A 2020- Effects of COVID-19 Lockdown on Lifestyle Behaviors in Children with Obesity Living in Verona, Italy: A Longitudinal Study
Dunton GF 2020- Early effects of the COVID-19 pandemic on physical activity and sedentary behavior in children living in the U.S
Guthold 2020- Global trends in insufficient physical activity among adolescents: a pooled analysis of 298 population-based surveys with 1·6 million participants
Biddle 2019- Physical activity and mental health in children and adolescents: An updated review of reviews and an analysis of causality
Pierce 2020- Mental health before and during the COVID-19 pandemic: a longitudinal probability sample survey of the UK population
Moore 2020- Impact of the COVID-19 virus outbreak on movement and play behaviours of Canadian children and youth: a national survey
Braun 2019- Novel insights into patients' life-worlds: the value of qualitative research
Guessoum 2020- Adolescent psychiatric disorders during the COVID-19 pandemic and lockdown

Cluster 5- Physical exercise and ‘greenness/urbanicity’					
5	41	0.866	2017	systematic review; physical activity; mental health; green space; case study | cross-sectional study; uk wetland visitor; qualitative analysis; health resource; asian urban environment	**green space** (775.89, 1.0E-4); case study (504.2, 1.0E-4); green space quality (486.75, 1.0E-4); urban green space (416.84, 1.0E-4); urban nature (288.32, 1.0E-4)

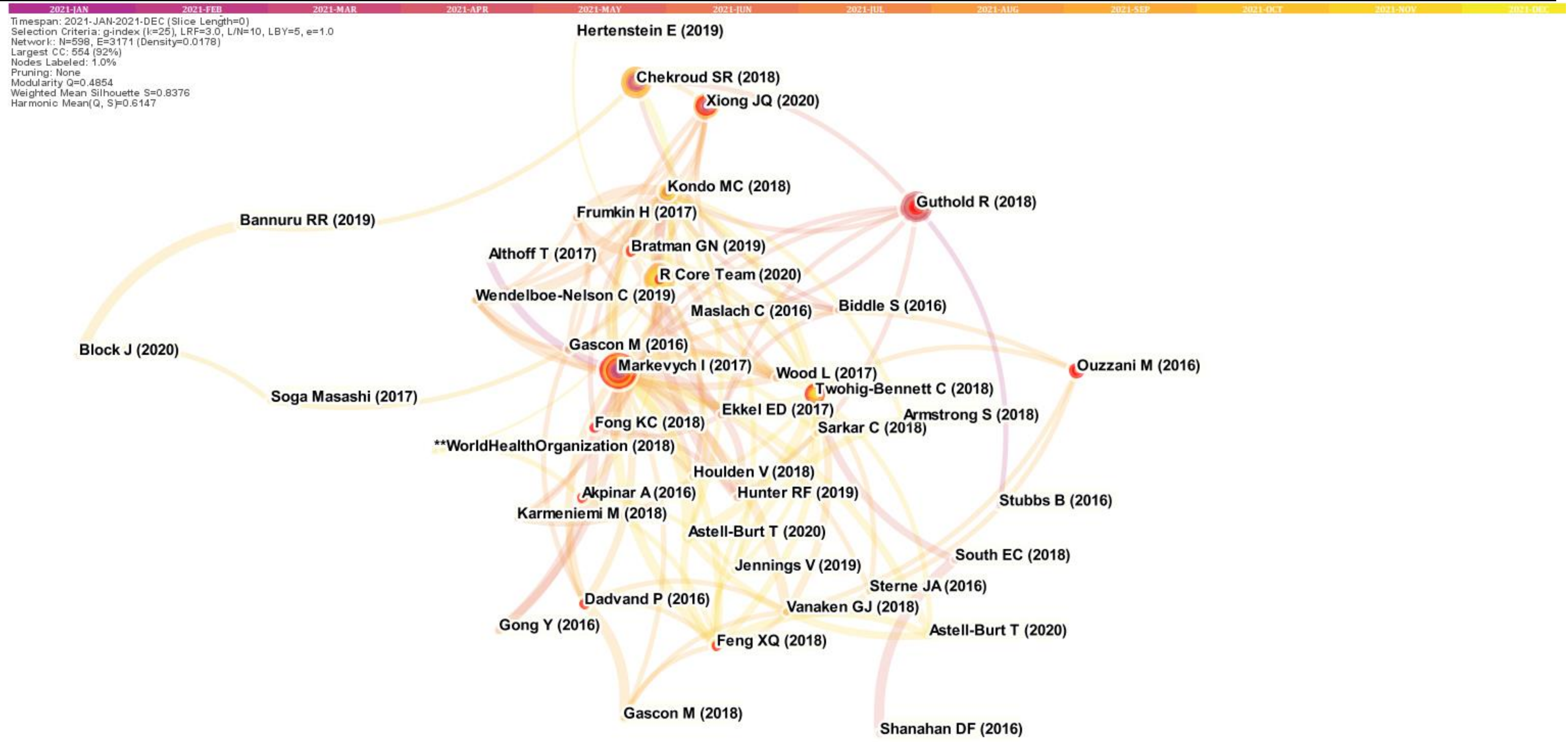

Papers with highest centrality:
Chekroud 2018- Association between physical exercise and mental health in 1·2 million individuals in the USA between 2011 and 2015: a cross-sectional study
Guthold 2018- Worldwide trends in insufficient physical activity from 2001 to 2016: a pooled analysis of 358 population-based surveys with 1·9 million participants
Twohig-benett 2018- The health benefits of the great outdoors: A systematic review and meta-analysis of greenspace exposure and health outcomes
Fong 2018- A Review of Epidemiologic Studies on Greenness and Health: Updated Literature Through 2017
Akpinar 2016- How is quality of urban green spaces associated with physical activity and health?
Dadvand 2016- Green spaces and General Health: Roles of mental health status, social support, and physical activity

Cluster 6- Physical exercise and 'NAFLD'

6	26	0.968	2017	non-alcoholic fatty liver disease; sub-saharan africa; current management; social determinant; study protocol | controlled trial; mediterranean diet adherence; multi-domain intervention; physical activity; uk adult	non-alcoholic fatty liver disease (994.13, 1.0E-4); sub-saharan africa (340.68, 1.0E-4); epidemiology risk factor (169.7, 1.0E-4); social determinant (169.7, 1.0E-4); current management (169.7, 1.0E-4)

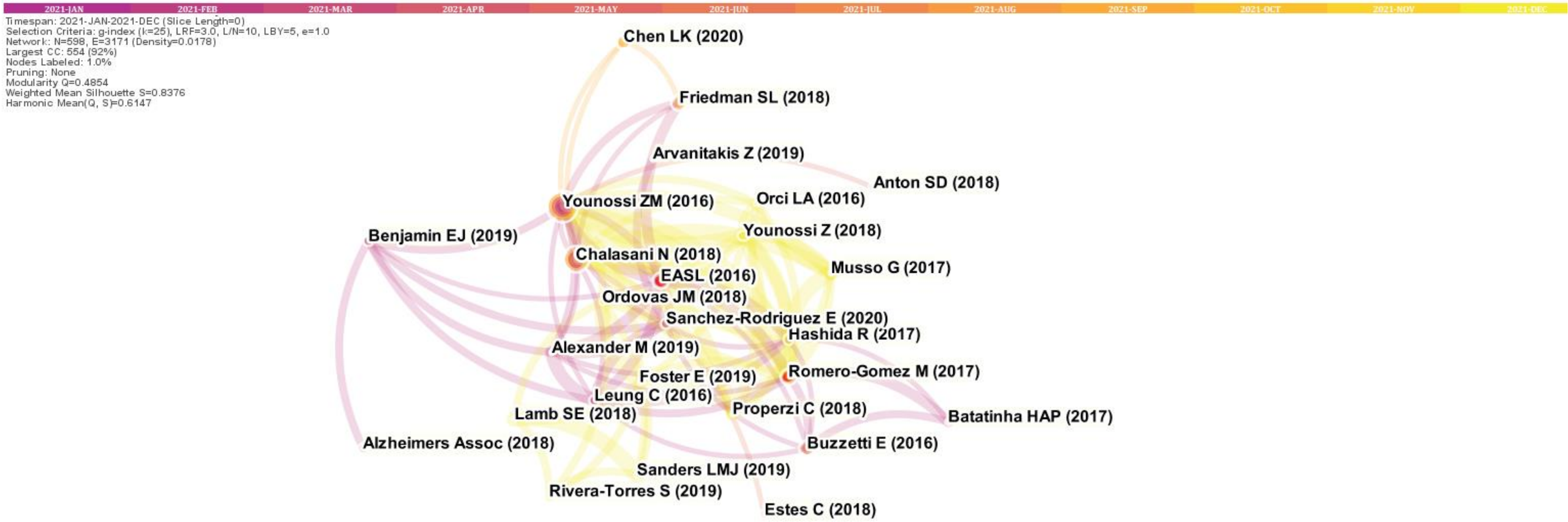

Papers with highest centrality:
Younossi 2016- Global epidemiology of nonalcoholic fatty liver disease-Meta-analytic assessment of prevalence, incidence, and outcomes
Chalasani N 2018- The diagnosis and management of nonalcoholic fatty liver disease: Practice guidance from the American Association for the Study of Liver Diseases
Orci 2016- Exercise-based Interventions for Nonalcoholic Fatty Liver Disease: A Meta-analysis and Meta-regression
Hashida 2017- Aerobic vs. resistance exercise in non-alcoholic fatty liver disease: A systematic review
Romero-Gomez 2017- Treatment of NAFLD with diet, physical activity and exercise
Lamb 2018- Dementia And Physical Activity (DAPA) trial of moderate to high intensity exercise training for people with dementia: randomised controlled trial

**Cluster 7- Physical exercise and ‘elite athletes’**					
7	25	0.961	2017	elite athlete; mental health profile; danish elite athlete; nutritional consideration; female athlete | long-term cognitive function; rugby players-the brain study; parental mental health; youth sport participation; collegiate athlete	elite athlete (444.1, 1.0E-4); mental health profile (247.1, 1.0E-4); danish elite athlete (247.1, 1.0E-4); nutritional consideration (238.55, 1.0E-4); female athlete (238.55, 1.0E-4)

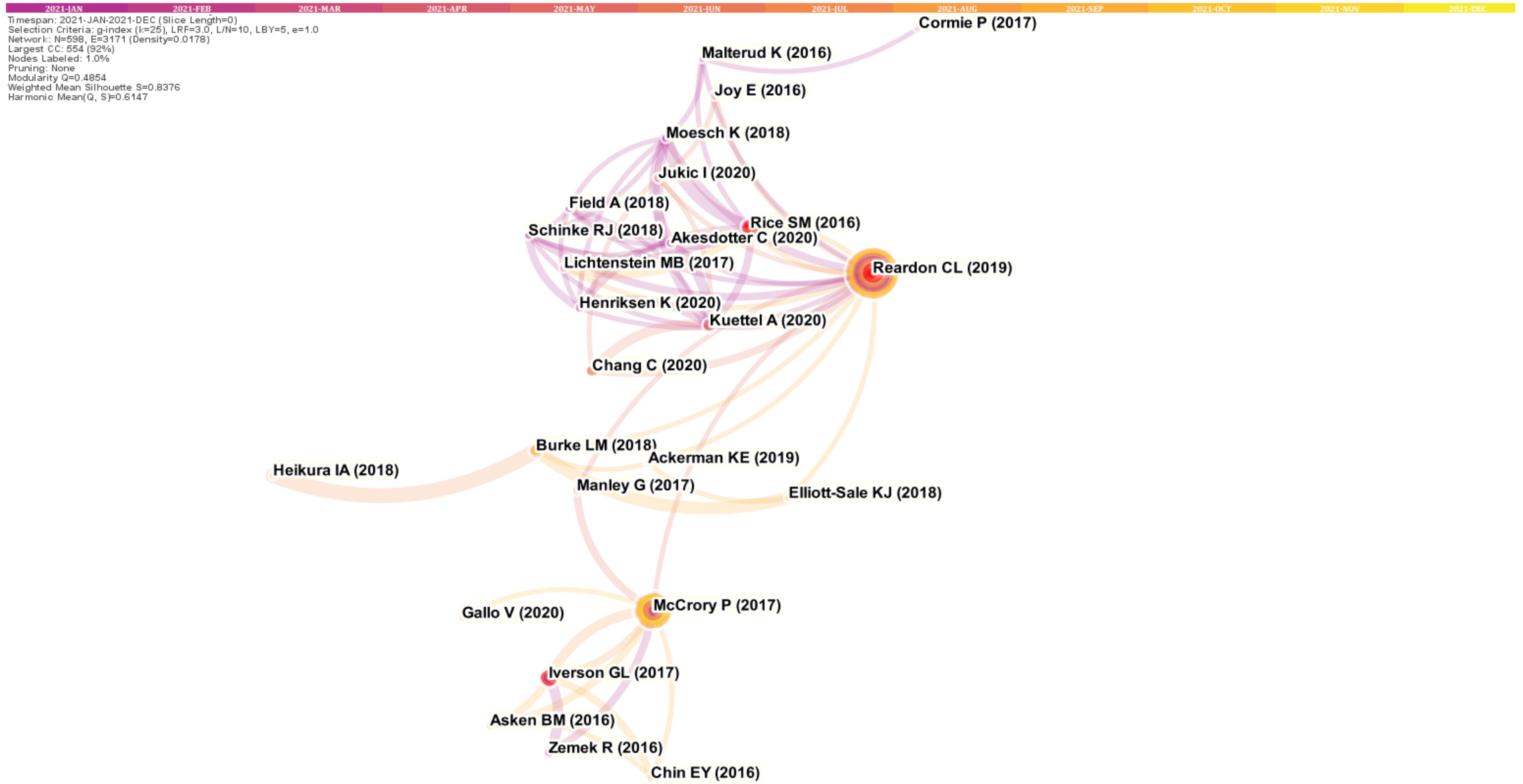

Papers with highest centrality:
Moesch 2018- FEPSAC position statement: Mental health disorders in elite athletes and models of service provision
Rice 2016- The Mental Health of Elite Athletes: A Narrative Systematic Review
Readron 2019- Mental health in elite athletes: International Olympic Committee consensus statement (2019)
Burke 2018- Pitfalls of Conducting and Interpreting Estimates of Energy Availability in Free-Living Athletes
Mccrocy 2017- Consensus statement on concussion in sport-the 5 th international conference on concussion in sport held in Berlin, October 2016
Ioverson 2017- Predictors of clinical recovery from concussion: a systematic review

**Cluster 9- Physical activity and 'cardiovascular disease'**					
9	4	1	2016	myocardial infarction patient; lifestyle modification; psychological effect; different urban green; street environment | myocardial infarction patient; lifestyle modification; fitness-dance training; psychological effect; different urban green	myocardial infarction patient (51.86, 1.0E-4); **lifestyle** modification (51.86, 1.0E-4); street environment (34.44, 1.0E-4); different urban green (34.44, 1.0E-4); dense inner-city area (34.44, 1.0E-4)

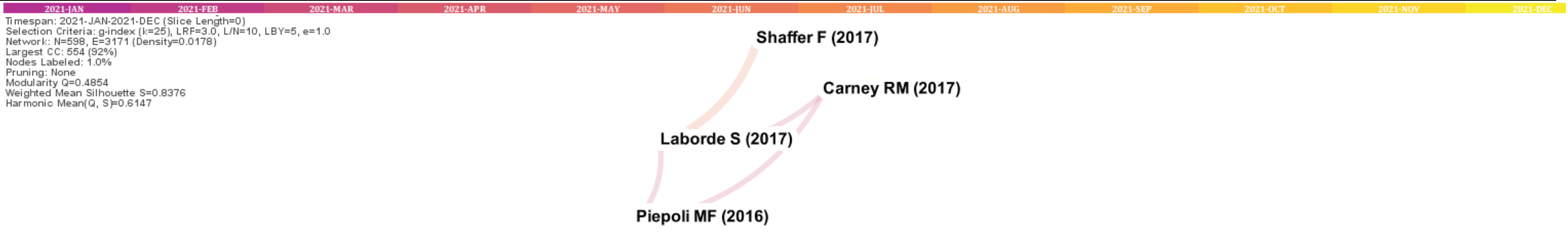

Papers with highest centrality:
Shaffer 2017- An Overview of Heart Rate Variability Metrics and Norms
Carney 2017- Depression and coronary heart disease
Laborder 2017- Heart Rate Variability and Cardiac Vagal Tone in Psychophysiological Research – Recommendations for Experiment Planning, Data Analysis, and Data Reporting
Piepoli 2016- 2016 European Guidelines on cardiovascular disease prevention in clinical practice

Supplementary Figure 5. Co-occurrence authors' keyword network (1988-2021)

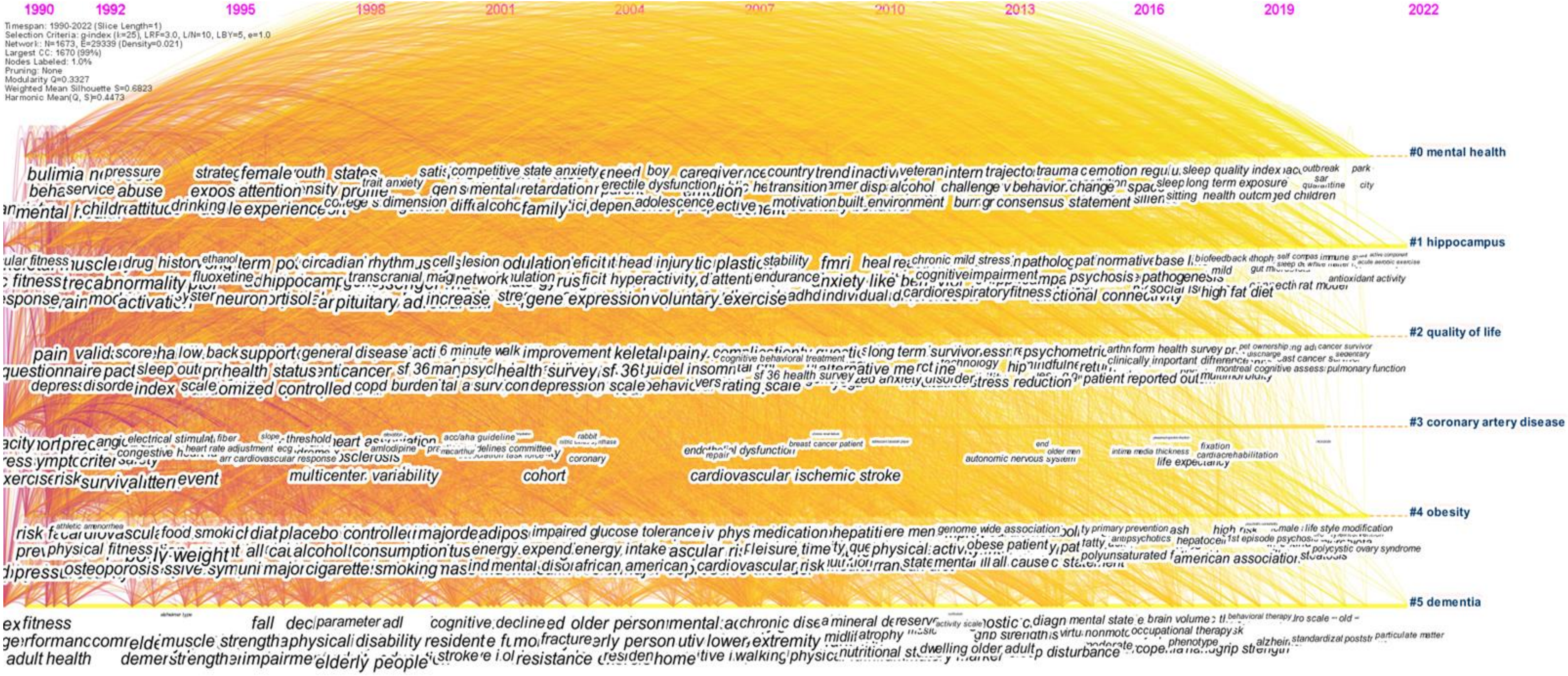

Supplementary Figure 6. Co-cited author’s countries network (2016-2021)

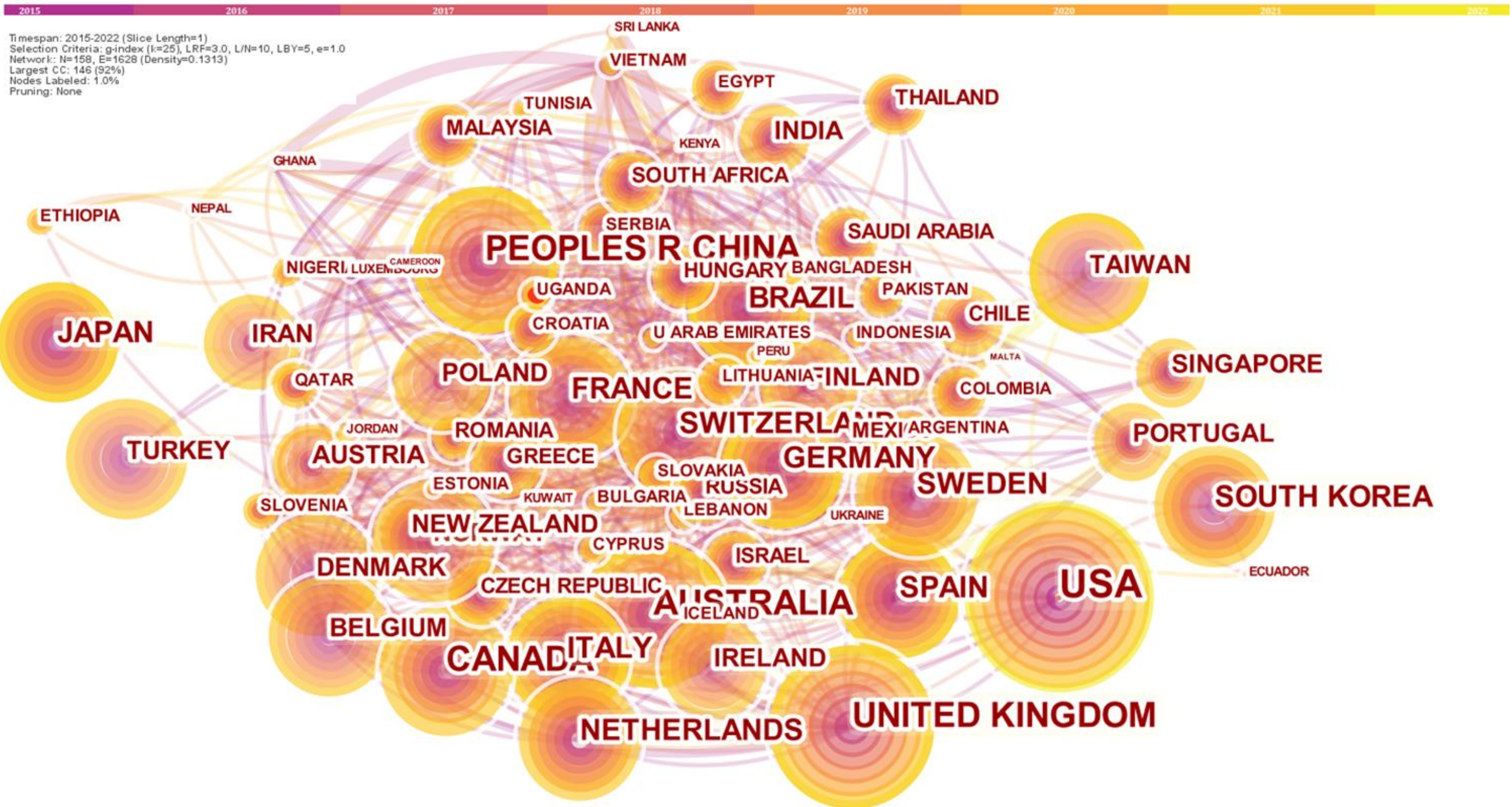

Supplementary Figure 7. Co-cited institution network details (2016-2021)

Co-cited institution network (2016-2021)
**Co-cited institution network (2016-2021)**

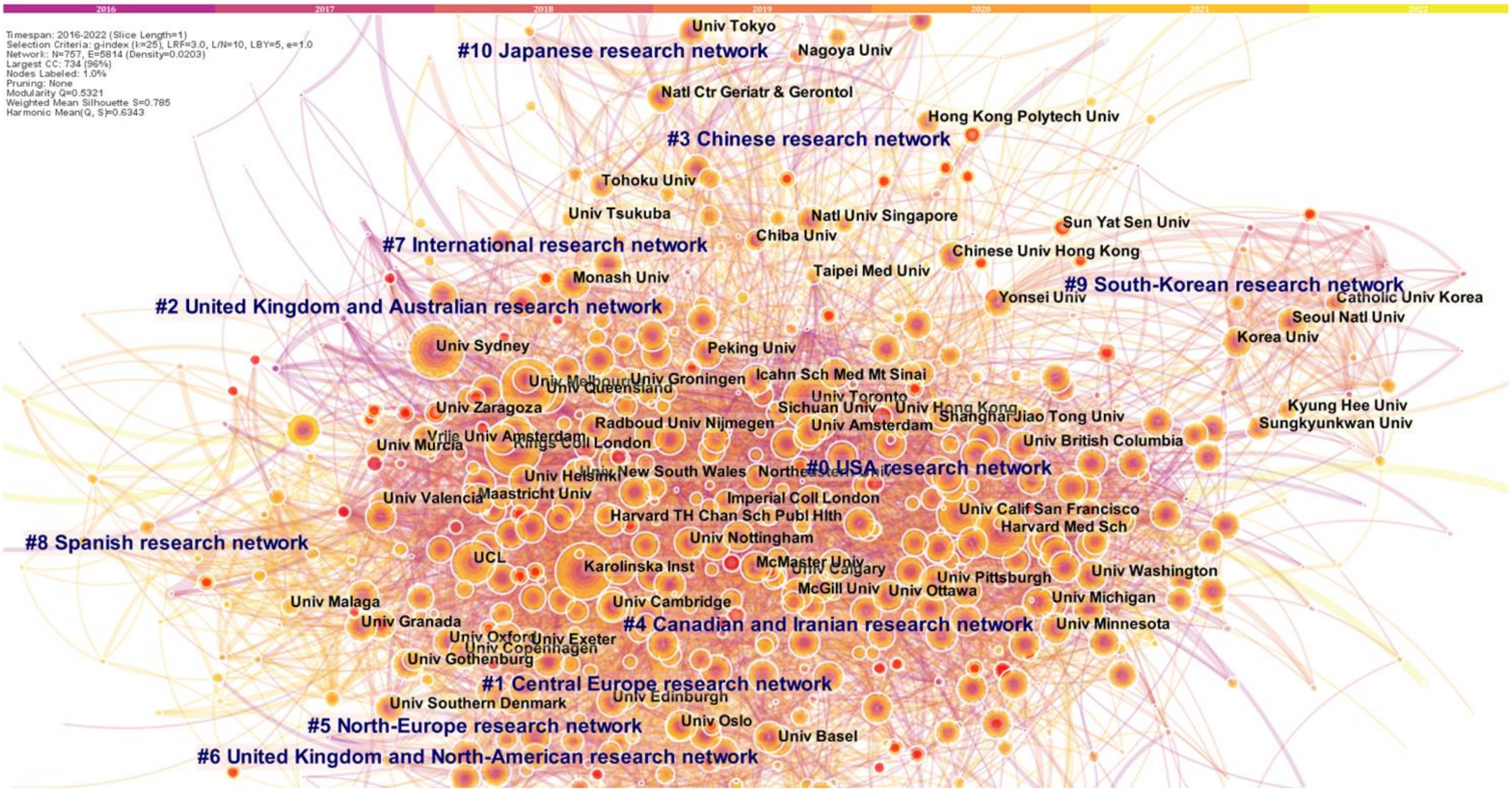

Co-cited institution network with detail of clusters (2016-2021)

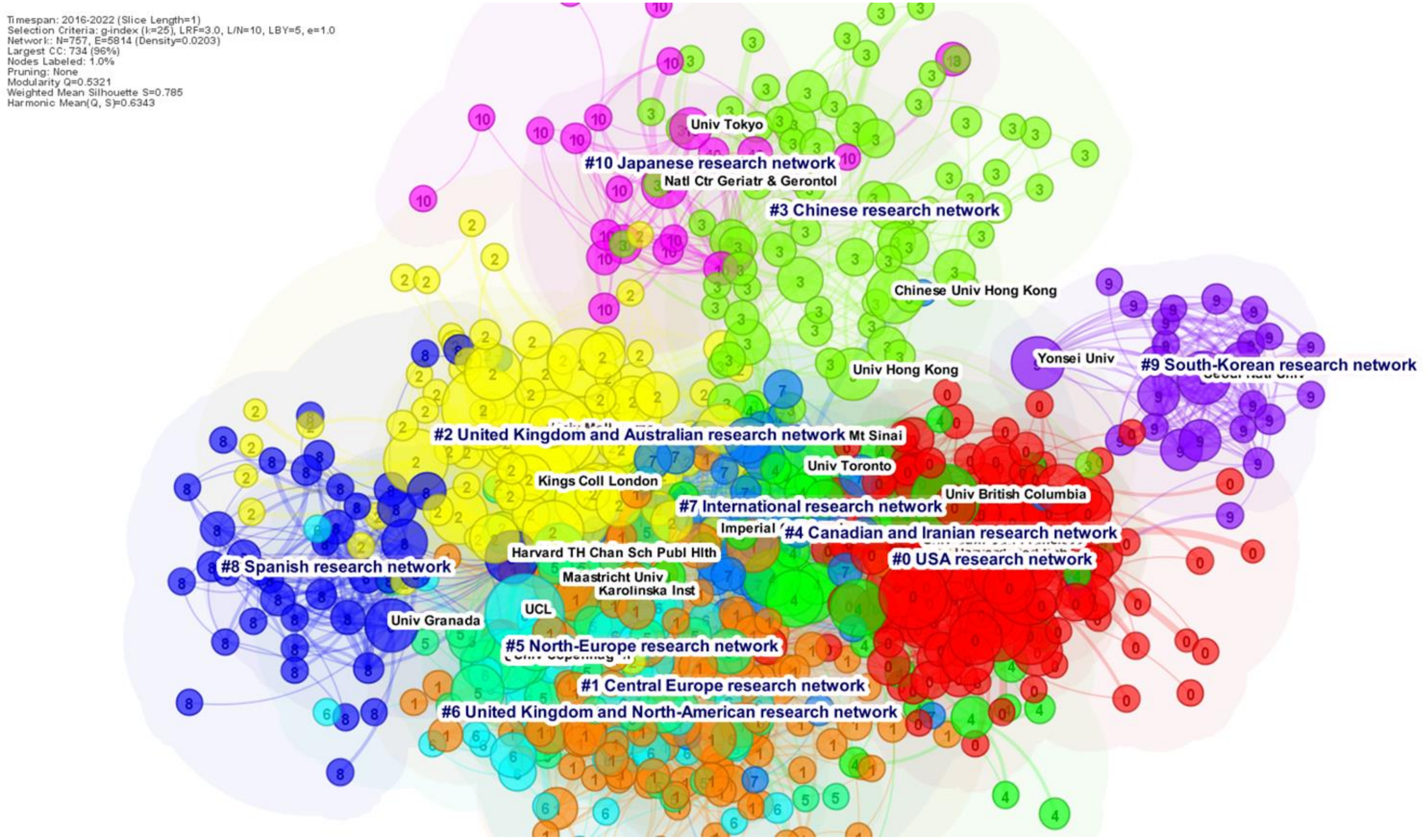

Cluster	Size	Silhouette score	Mean year	Top extracted terms based on keywords (Log-likelihood ratio algorithm; p-level)
0	141	0.762	2017	multi-ethnic study (23302.31, 1.0E-4); atherosclerosis risk (22066.04, 1.0E-4); knee osteoarthritis (18432.36, 1.0E-4); jackson heart study (18276.17, 1.0E-4); middle-income countries (17474.59, 1.0E-4)

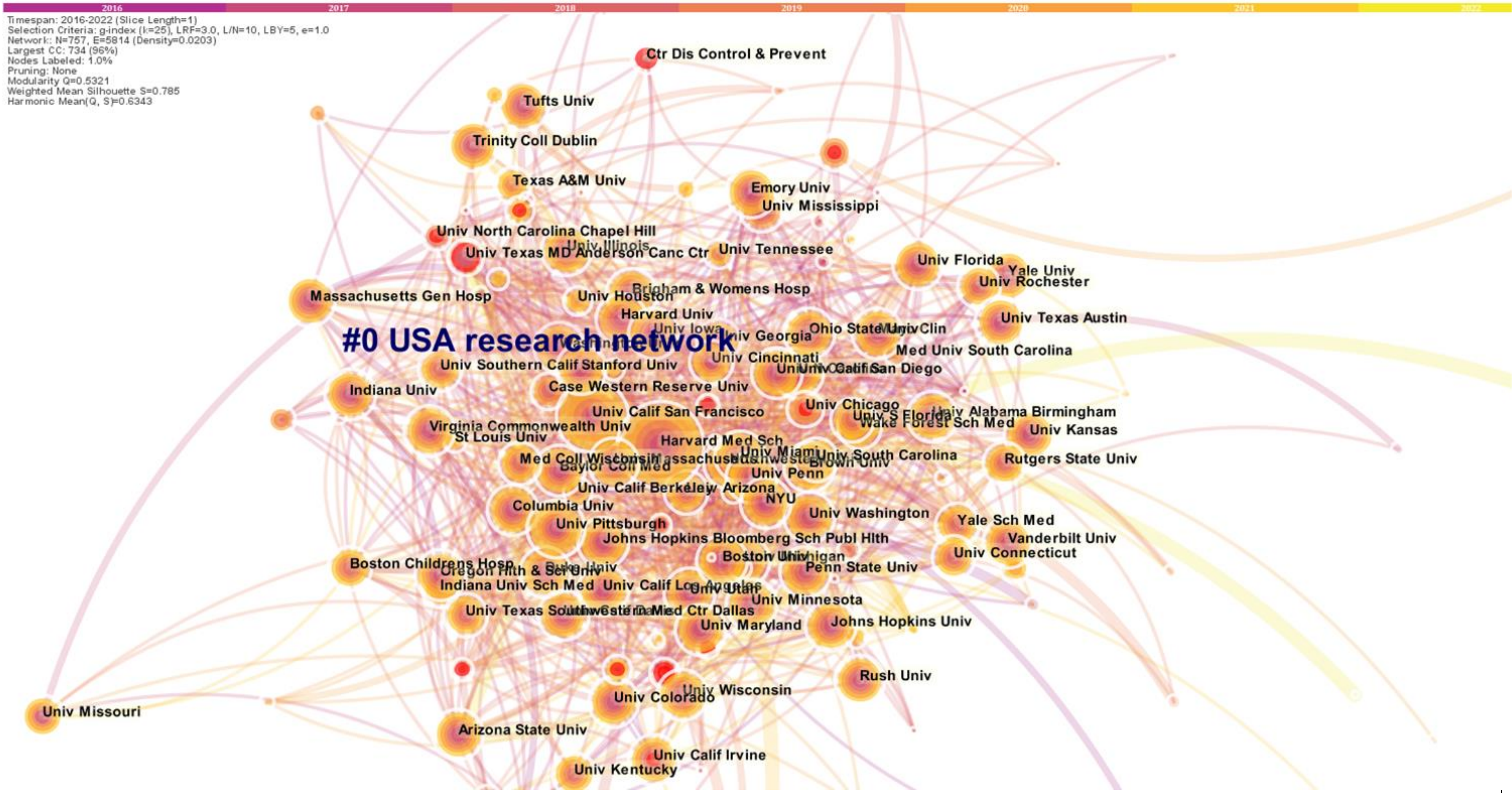

1	129	0.602	2017	late-life depression (10953.28, 1.0E-4); middle-income countries (10774.88, 1.0E-4); panic disorder (10354.96, 1.0E-4); colorectal cancer survivor (8931.79, 1.0E-4); childhood cancer (8770.47, 1.0E-4)

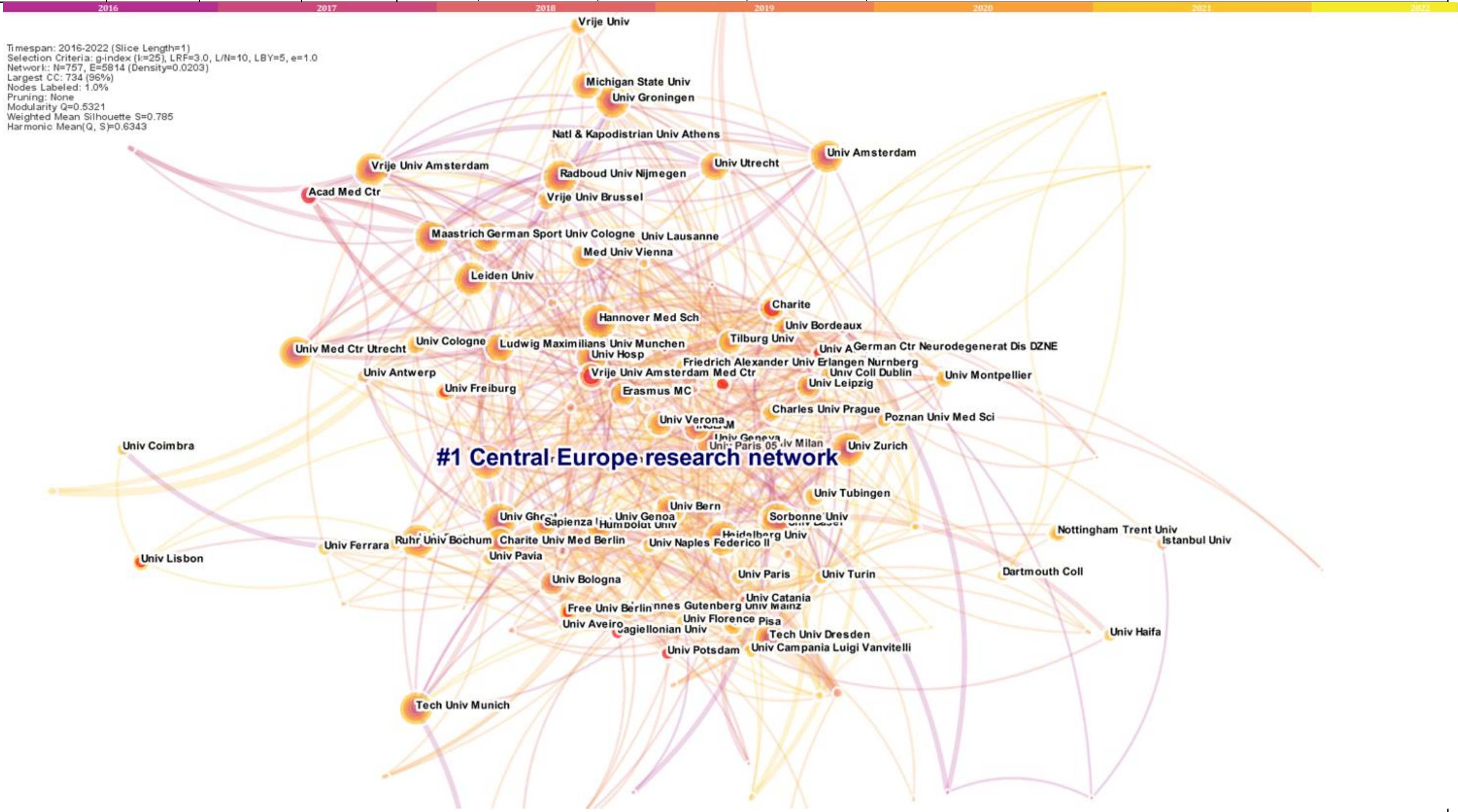

2	100	0.839	2017	middle-income countries (89202.62, 1.0E-4); sedentary behavior (24427, 1.0E-4); systematic review (19315.31, 1.0E-4); community-dwelling adult (14572.85, 1.0E-4); sleep problem (11241.43, 1.0E-4)

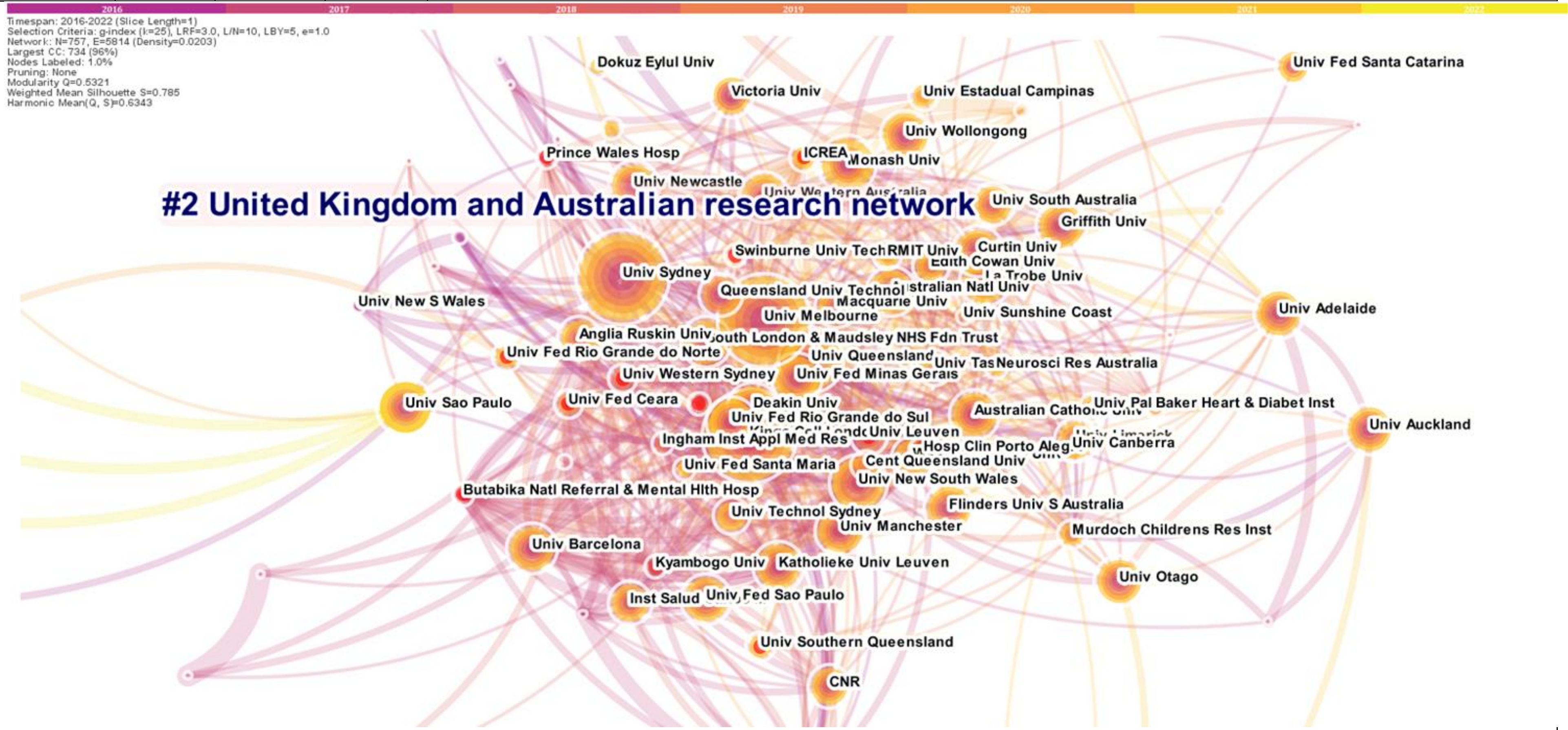

3	76	0.774	2018	wuhan china (8292.83, 1.0E-4); coronavirus disease (7218.4, 1.0E-4); chinese version (6881.02, 1.0E-4); covid-19 outbreak (6304.67, 1.0E-4); chinese adult (6049.04, 1.0E-4)

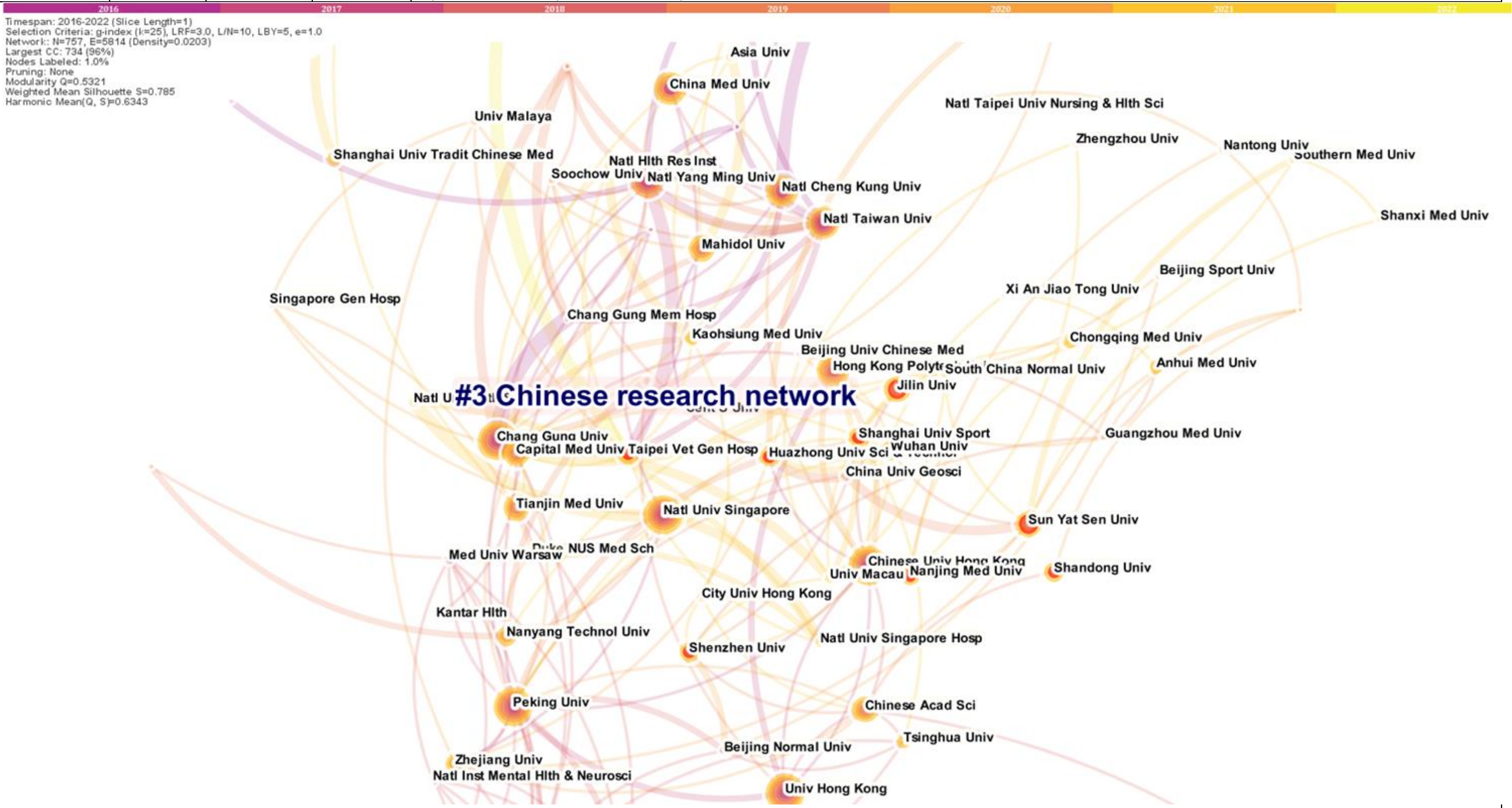

4	53	0.833	2017	iranian children (5646.82, 1.0E-4); following surgery (5343.63, 1.0E-4); spine surgery (5337.97, 1.0E-4); middle-income countries (4942.72, 1.0E-4); of-concept randomised controlled trial (4790.67, 1.0E-4)

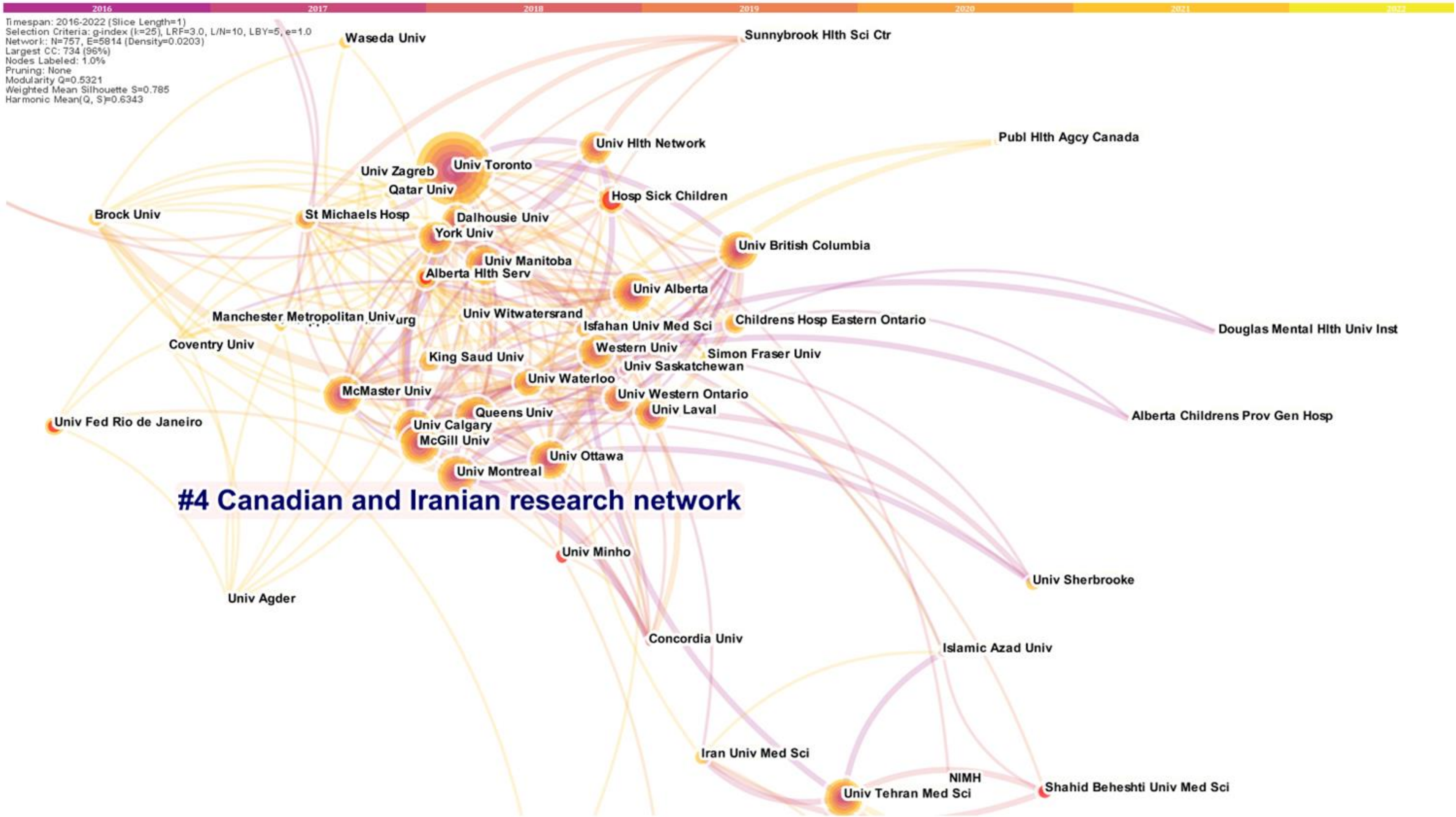

5	51	0.867	2017	systematic review (5671.75, 1.0E-4); hunt study (5154.09, 1.0E-4); population-based cohort study (4865.12, 1.0E-4); finnish geriatric intervention study (4752.36, 1.0E-4); midlife physical activity (4716.38, 1.0E-4)

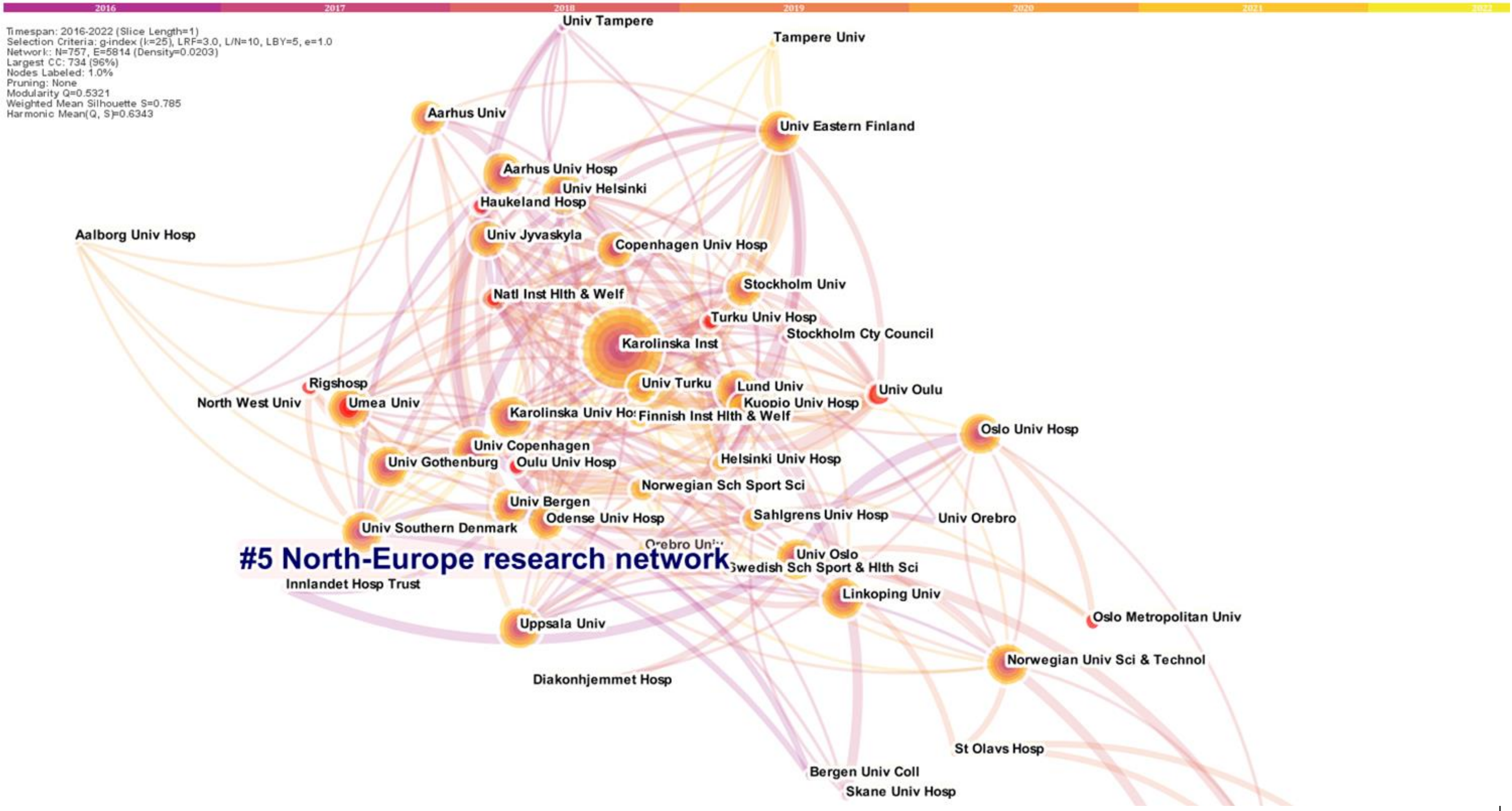

6	46	0.814	2017	structured lifestyle education (5100.18, 1.0E-4); chronic health condition (5088.85, 1.0E-4); inactive adult (5078.38, 1.0E-4); schizophrenia schizoaffective disorder (5021.63, 1.0E-4); using web-based behavioural support (5010.68, 1.0E-4)

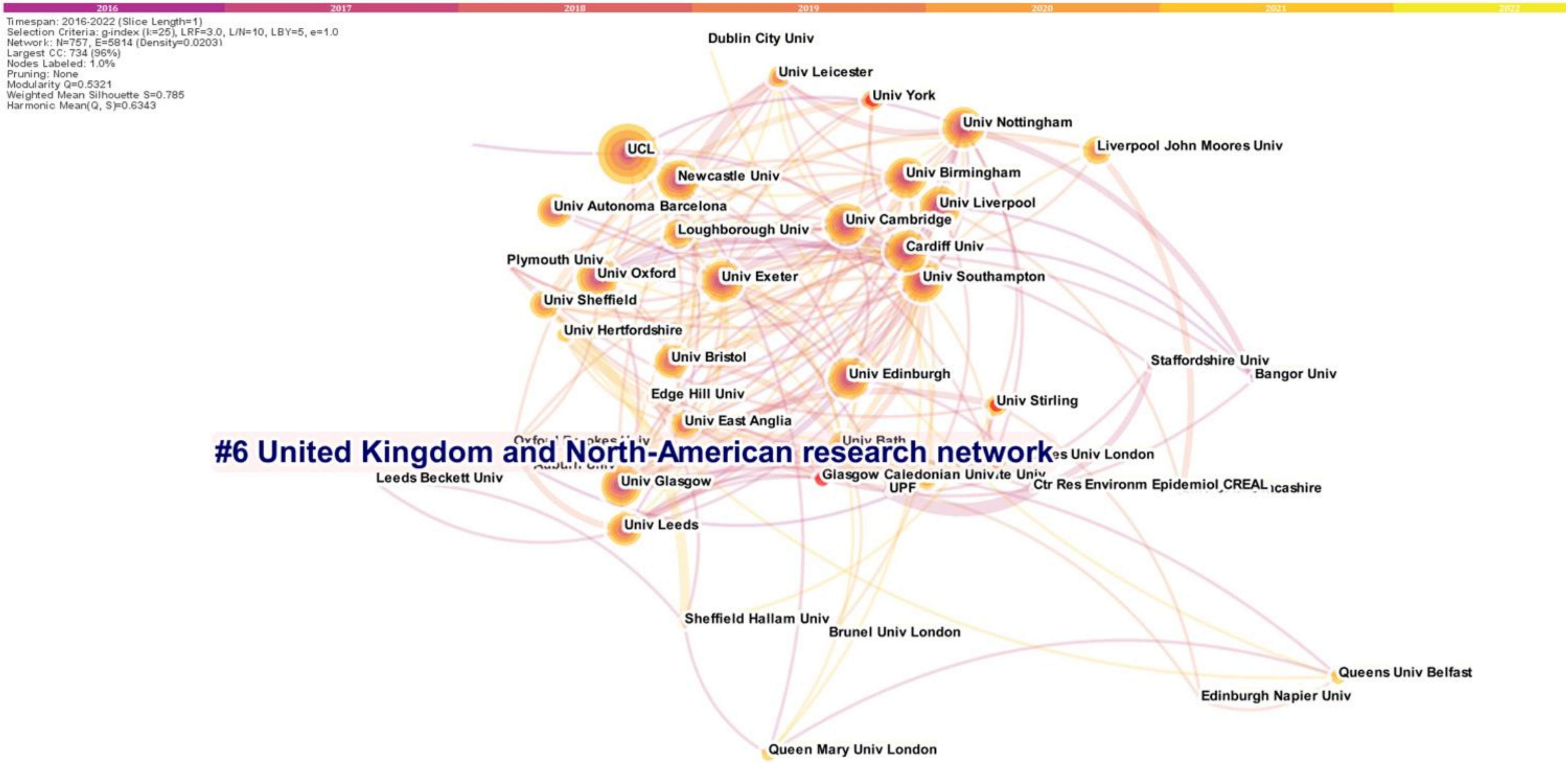

7	46	0.84	2016	turkish version (4791.44, 1.0E-4); anxiety level (4614.95, 1.0E-4); adolescent health (4445.52, 1.0E-4); poor quality (3577.38, 1.0E-4); current perspective (3136.96, 1.0E-4)

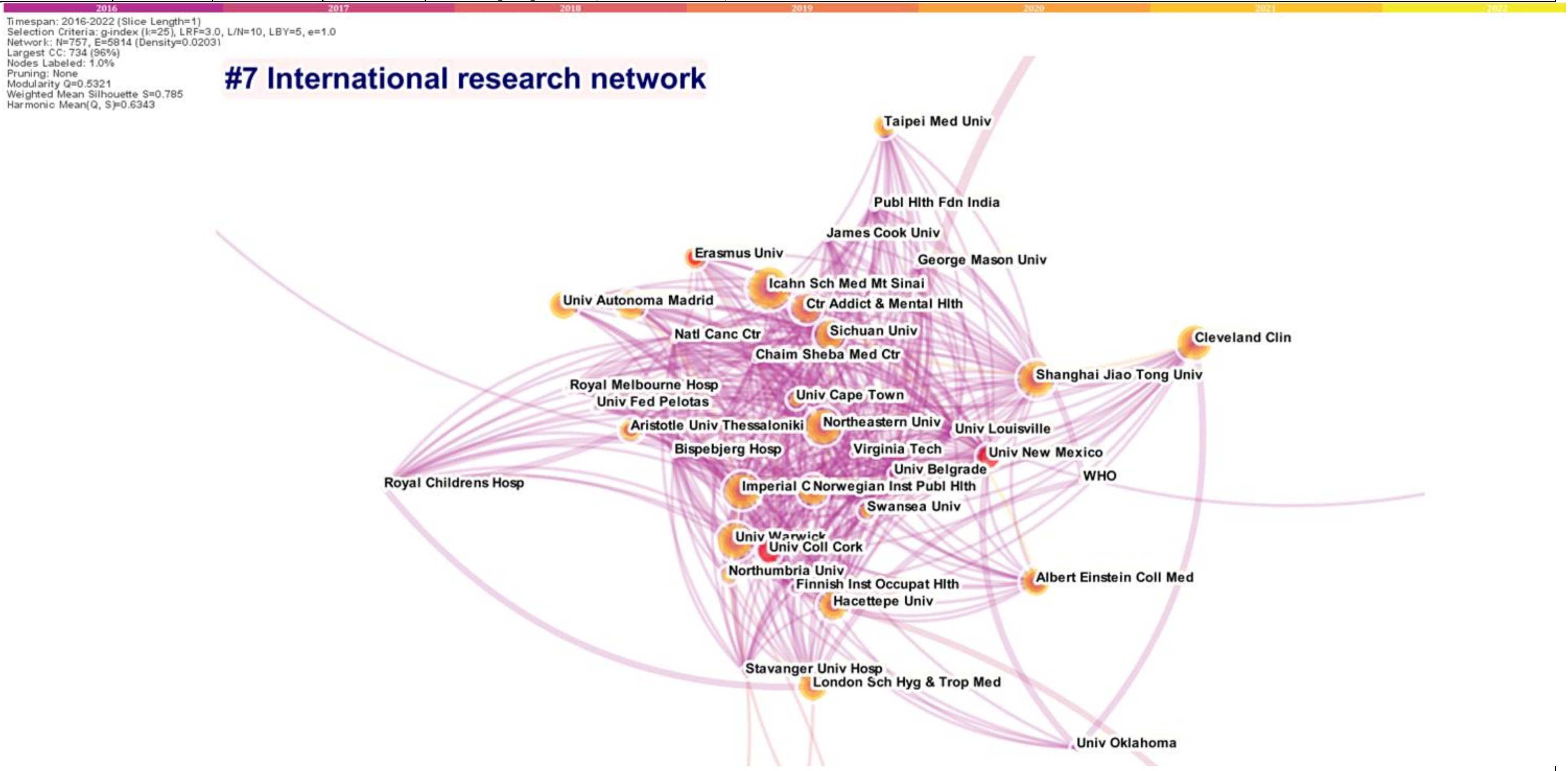

8	42	0.844	2019	covid-19 confinement (5588.91, 1.0E-4); diet quality (3867.07, 1.0E-4); al-andalus project (3828.7, 1.0E-4); high cardiovascular risk (3102.4, 1.0E-4); chronic gastrocnemius (2842.18, 1.0E-4)

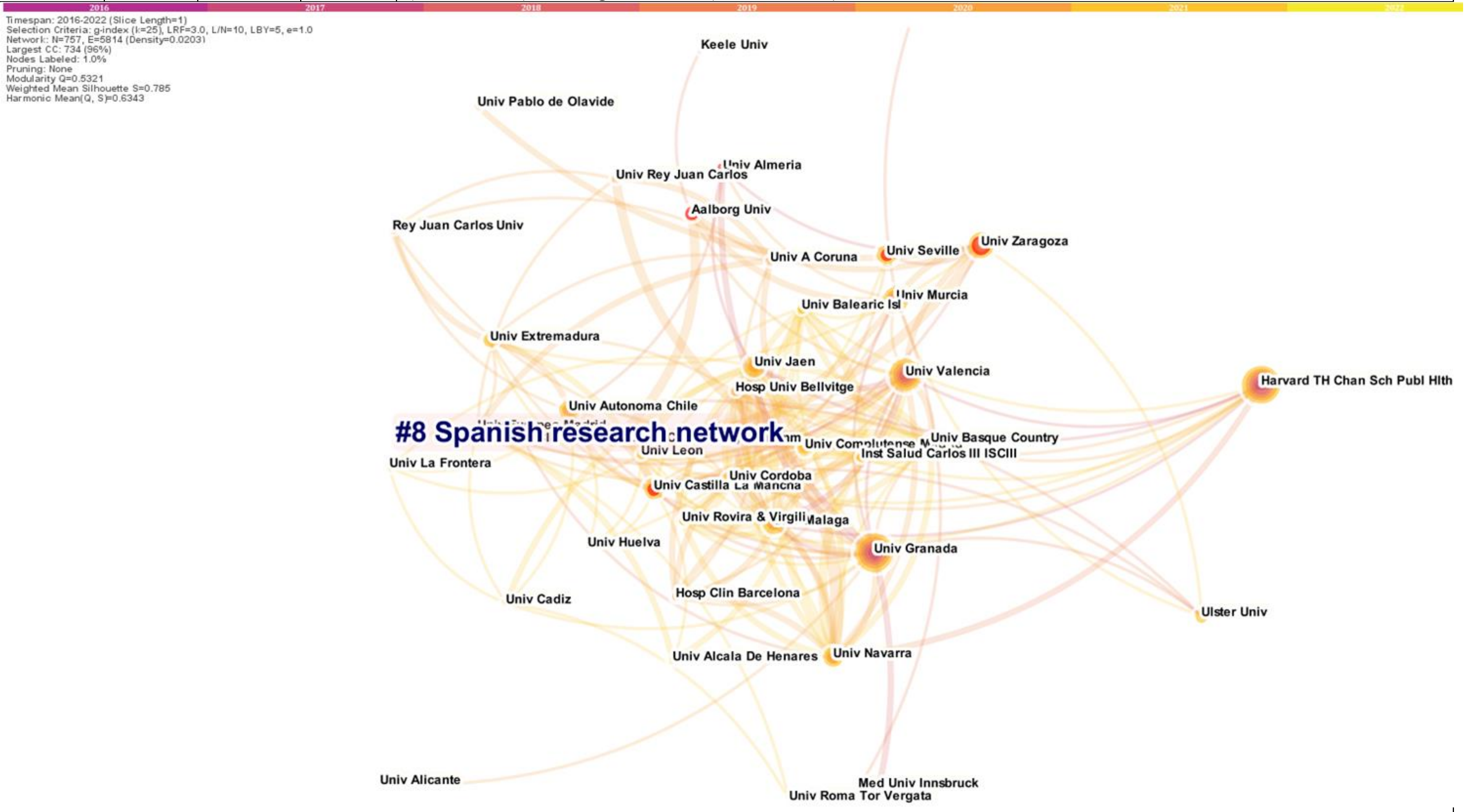

9	26	0.975	2017	south korea (4589.39, 1.0E-4); korean adolescent (3409.48, 1.0E-4); nationwide questionnaire study (2542.78, 1.0E-4); korean longitudinal study (2301.69, 1.0E-4); korean national health (2274.91, 1.0E-4)

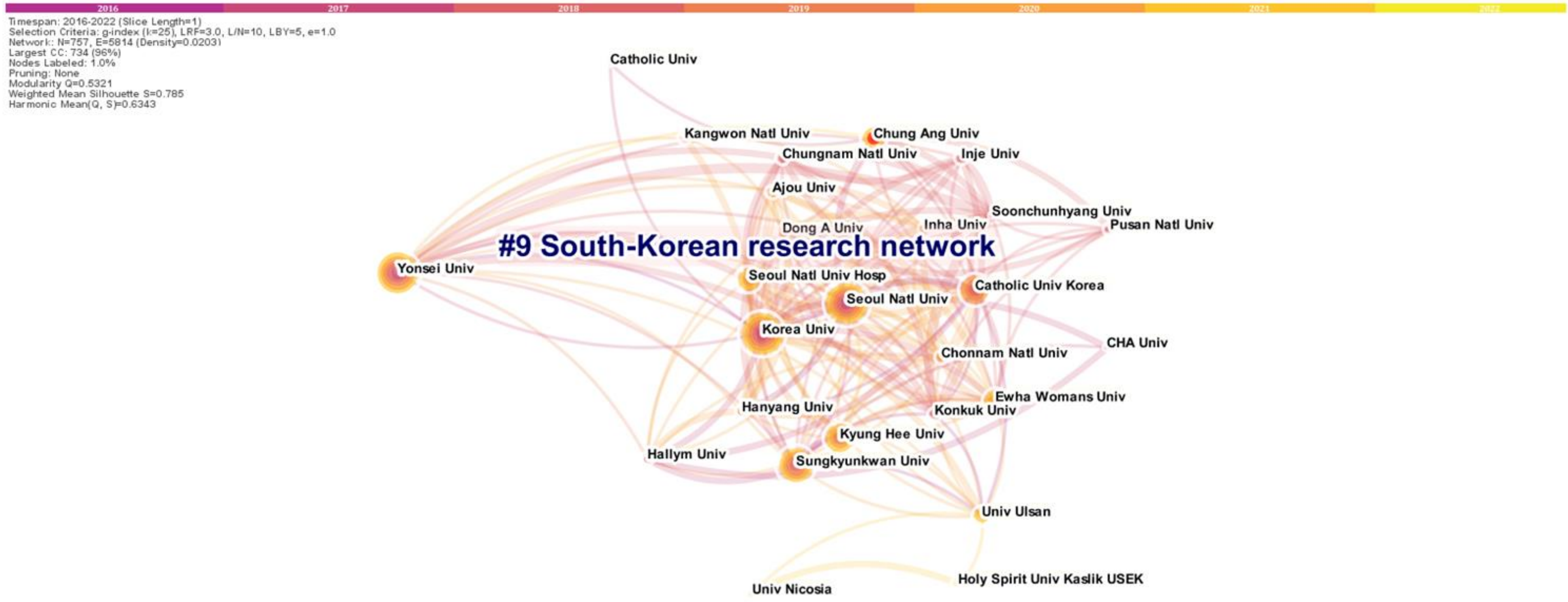

10	24	0.968	2017	japanese adult (5333.25, 1.0E-4); prospective follow-up study (2281.4, 1.0E-4); japan gerontological evaluation study (2223.35, 1.0E-4); social capital (2068.3, 1.0E-4); japan environment (2068.3, 1.0E-4)

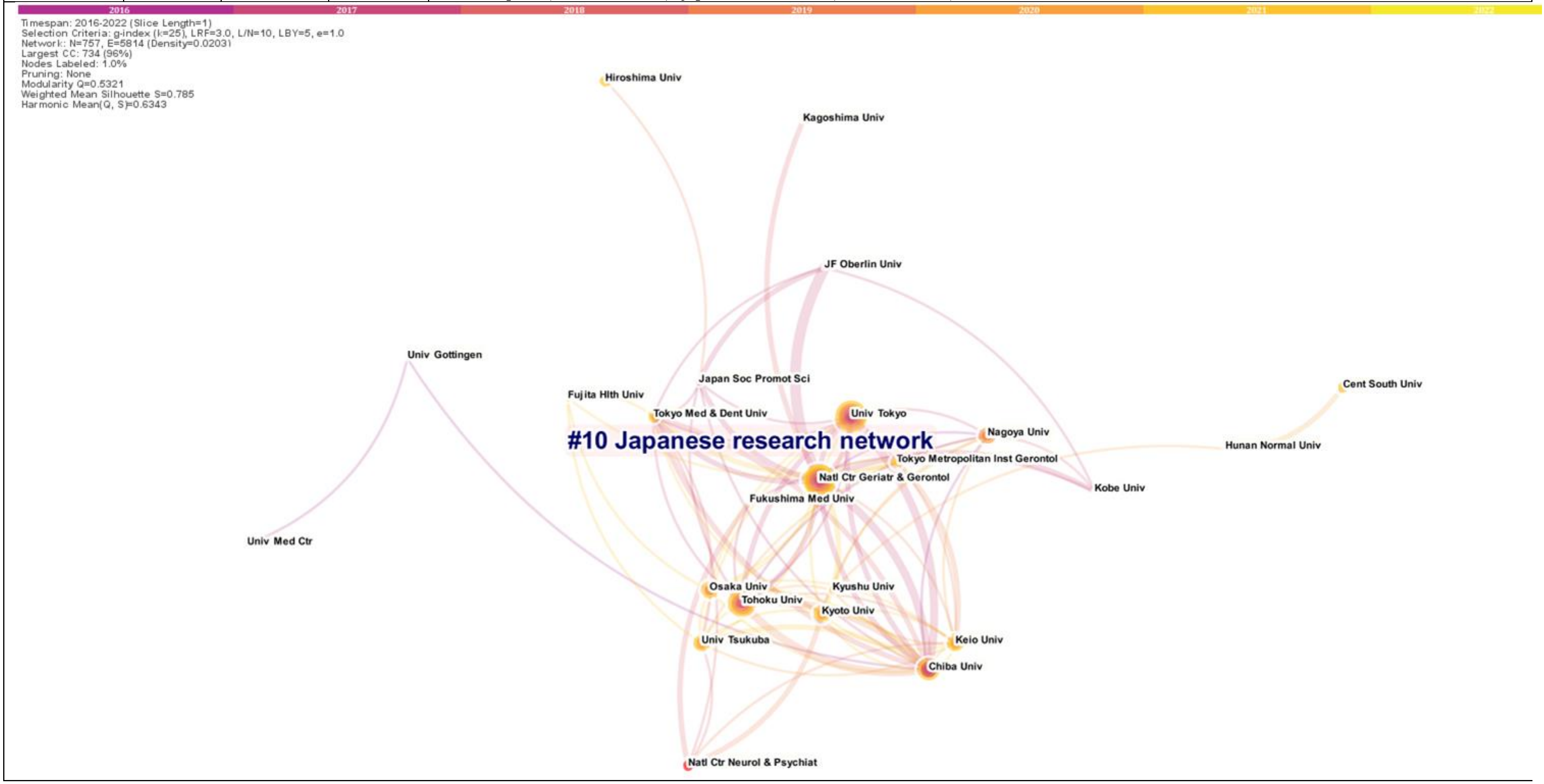

Supplementary Figure 8. Co-authorship network detail (1988-2021)

Co-authorship network (1988-2021)

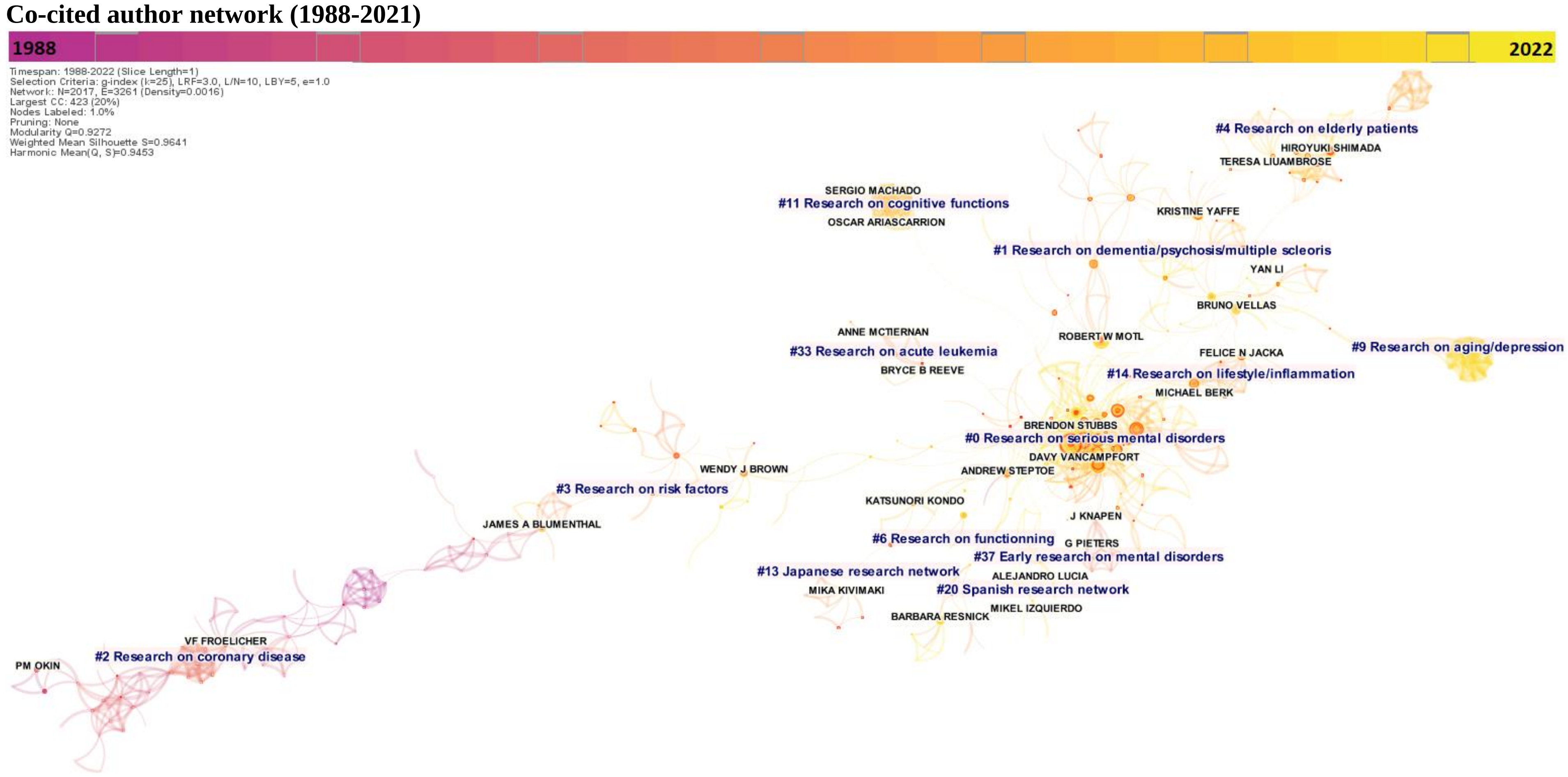

Co-cited author network with detail of clusters (1988-2021)

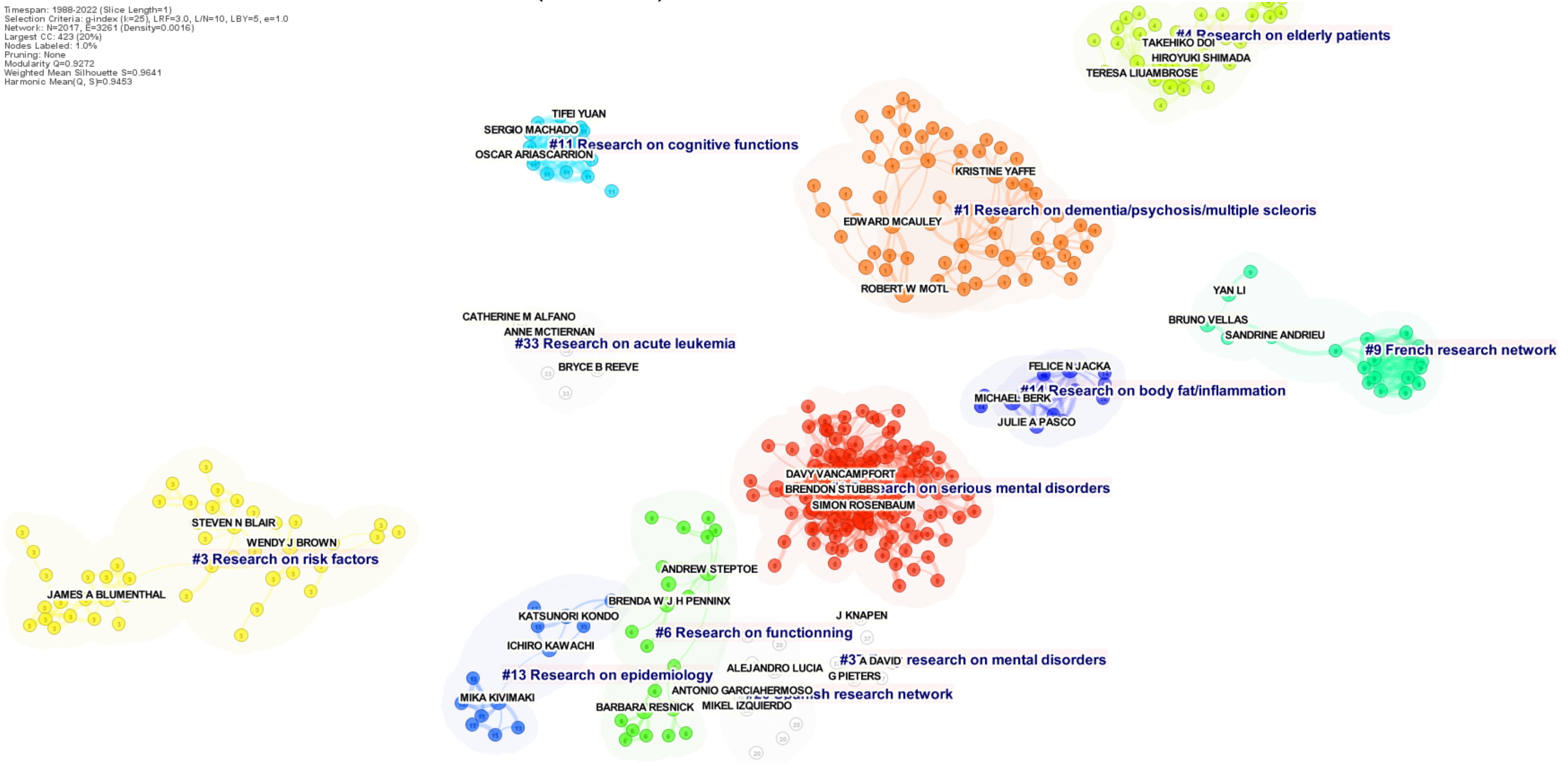

0	116	0.896	2015	physical activity (53.32, 1.0E-4); schizophrenia (49.96, 1.0E-4); psychosis (44.08, 1.0E-4); bipolar disorder (24.96, 1.0E-4); sitting (23.9, 1.0E-4)

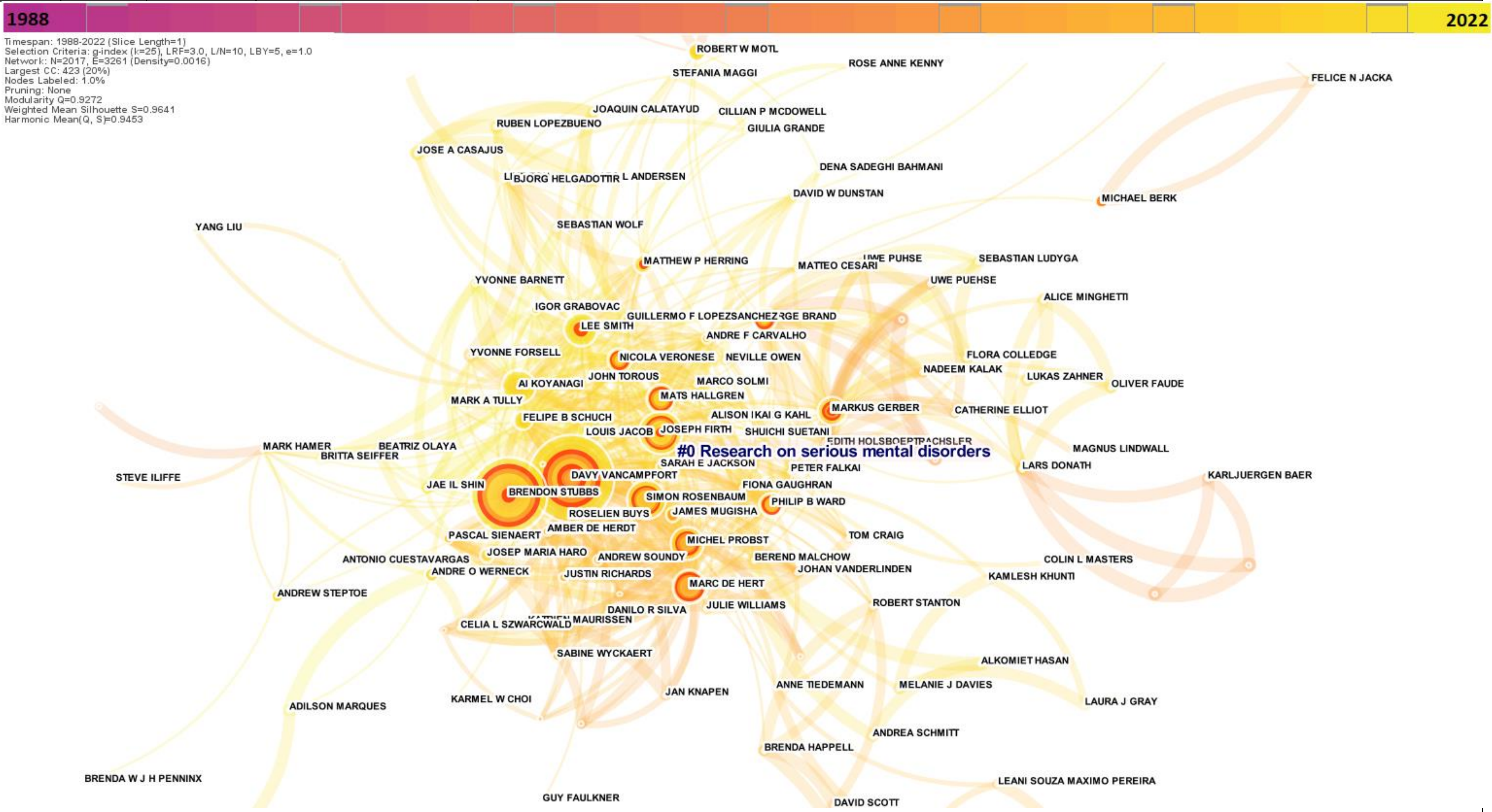

1	64	0.97	2012	dementia (19.95, 1.0E-4); accidental falls (18.61, 1.0E-4); schizophrenia (16.82, 1.0E-4); multiple sclerosis (16.17, 1.0E-4); exercise (15.14, 1.0E-4)

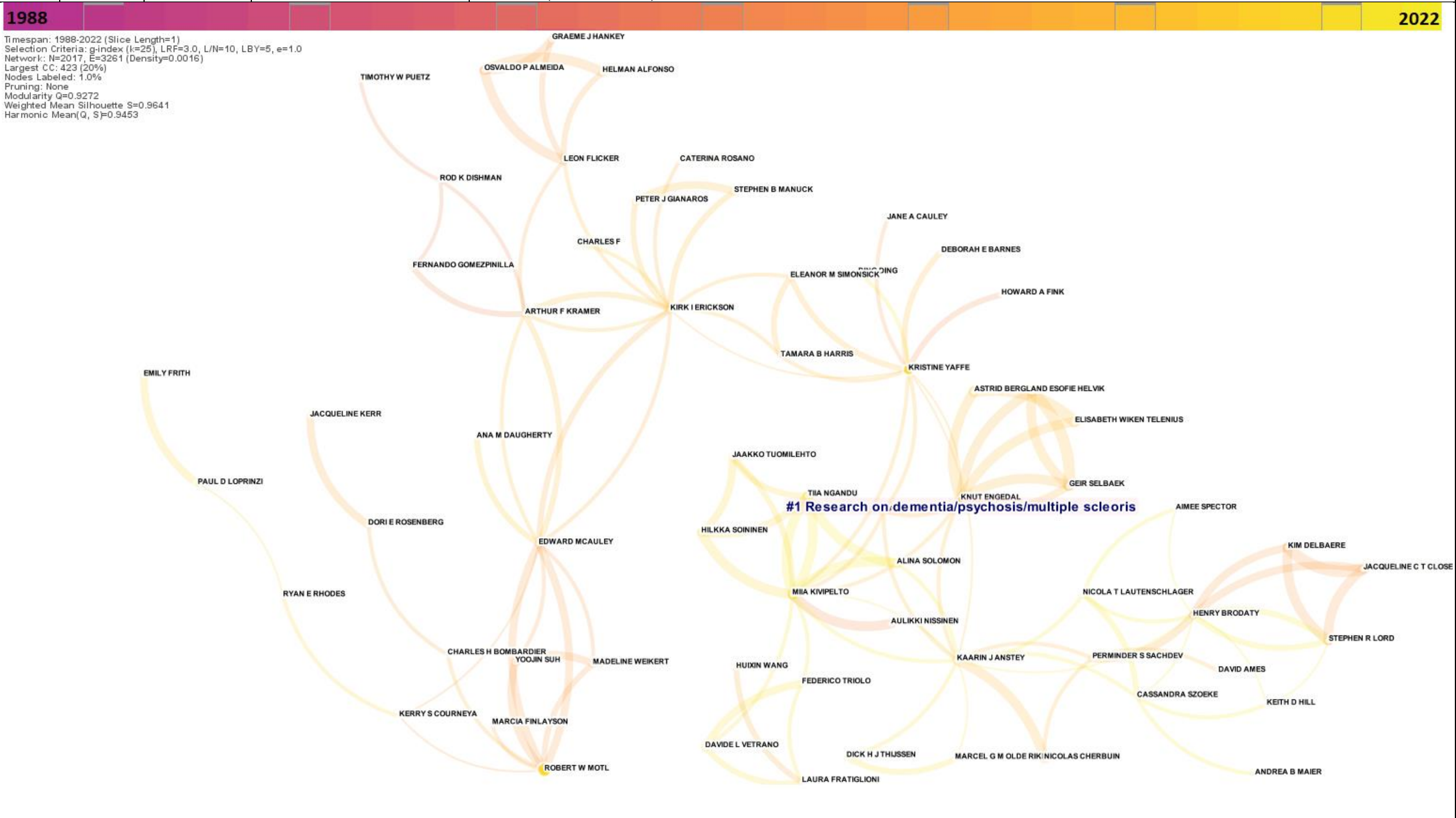

2	58	1	1994	coronary disease (38.01, 1.0E-4); exercise testing (36.33, 1.0E-4); coronary artery disease (21.7, 1.0E-4); coronary angiography (21.7, 1.0E-4); electrocardiography (21.7, 1.0E-4)

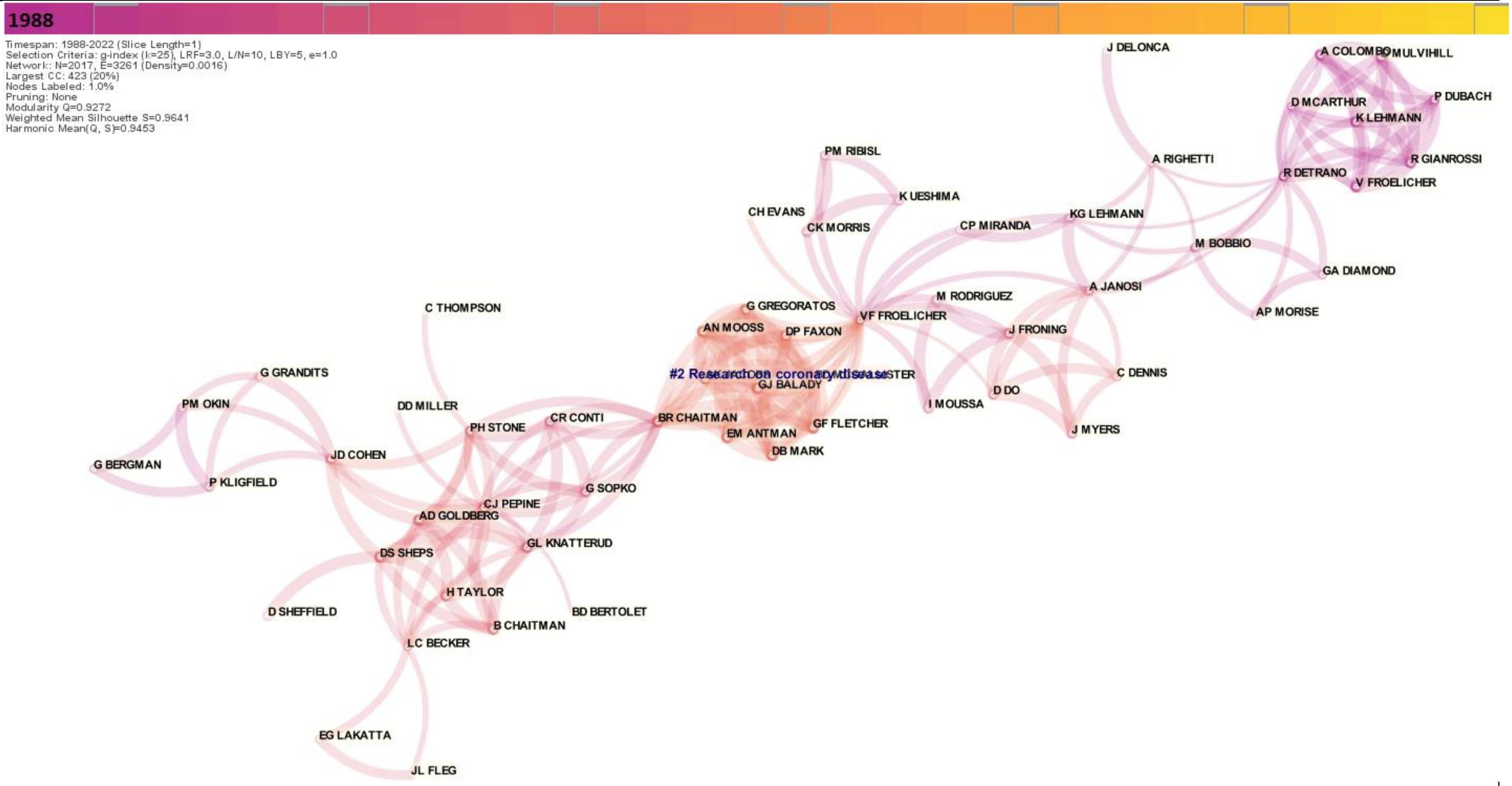

3	46	0.992	2007	risks (10.33, 0.005); benefits (10.33, 0.005); sedentariness (10.33, 0.005); wellbeing (10.33, 0.005); sleep (8.36, 0.005)

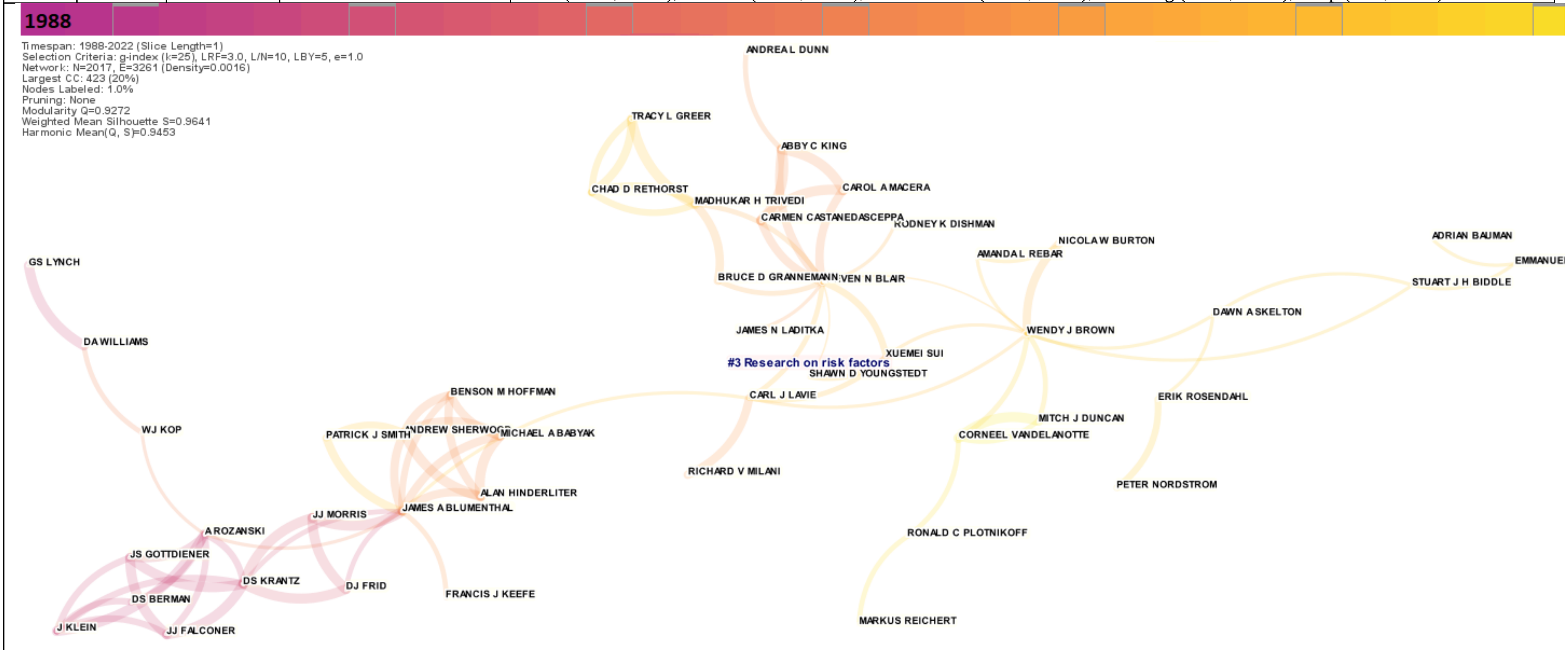

4	33	0.983	2014	gait (17.59, 1.0E-4); elderly (16.73, 1.0E-4); vascular health (11.21, 0.001); cortical thickness (11.21, 0.001); appetite loss (11.21, 0.001)

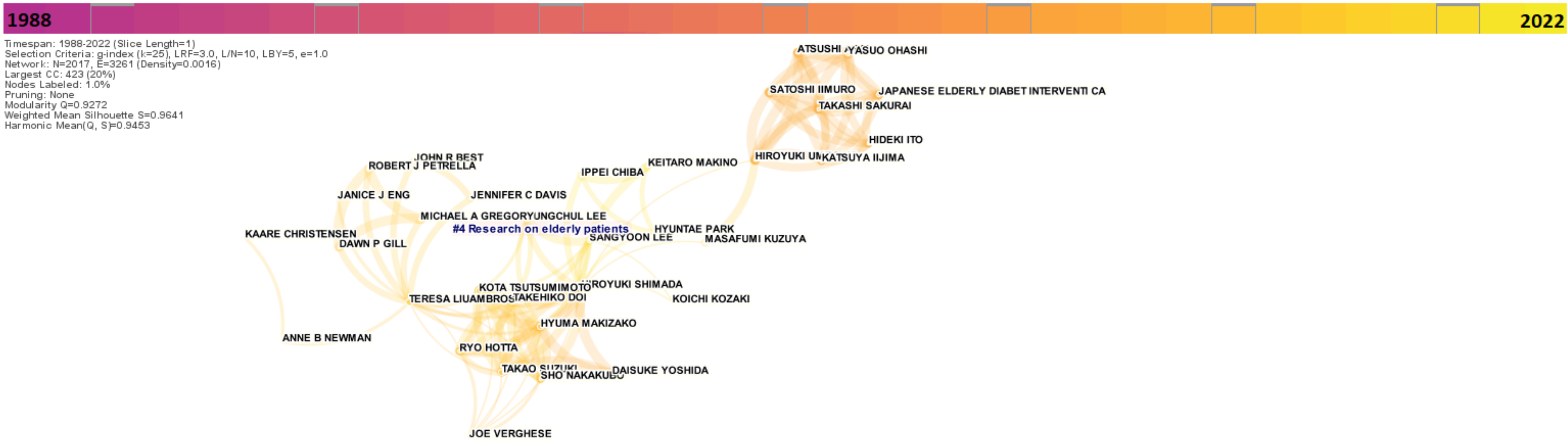

6	22	0.994	2009	function (20.89, 1.0E-4); nursing home residents (15.65, 1.0E-4); actigraphy (11.31, 0.001); cognitive impairment (10.9, 0.001); sleep disorder (10.42, 0.005)

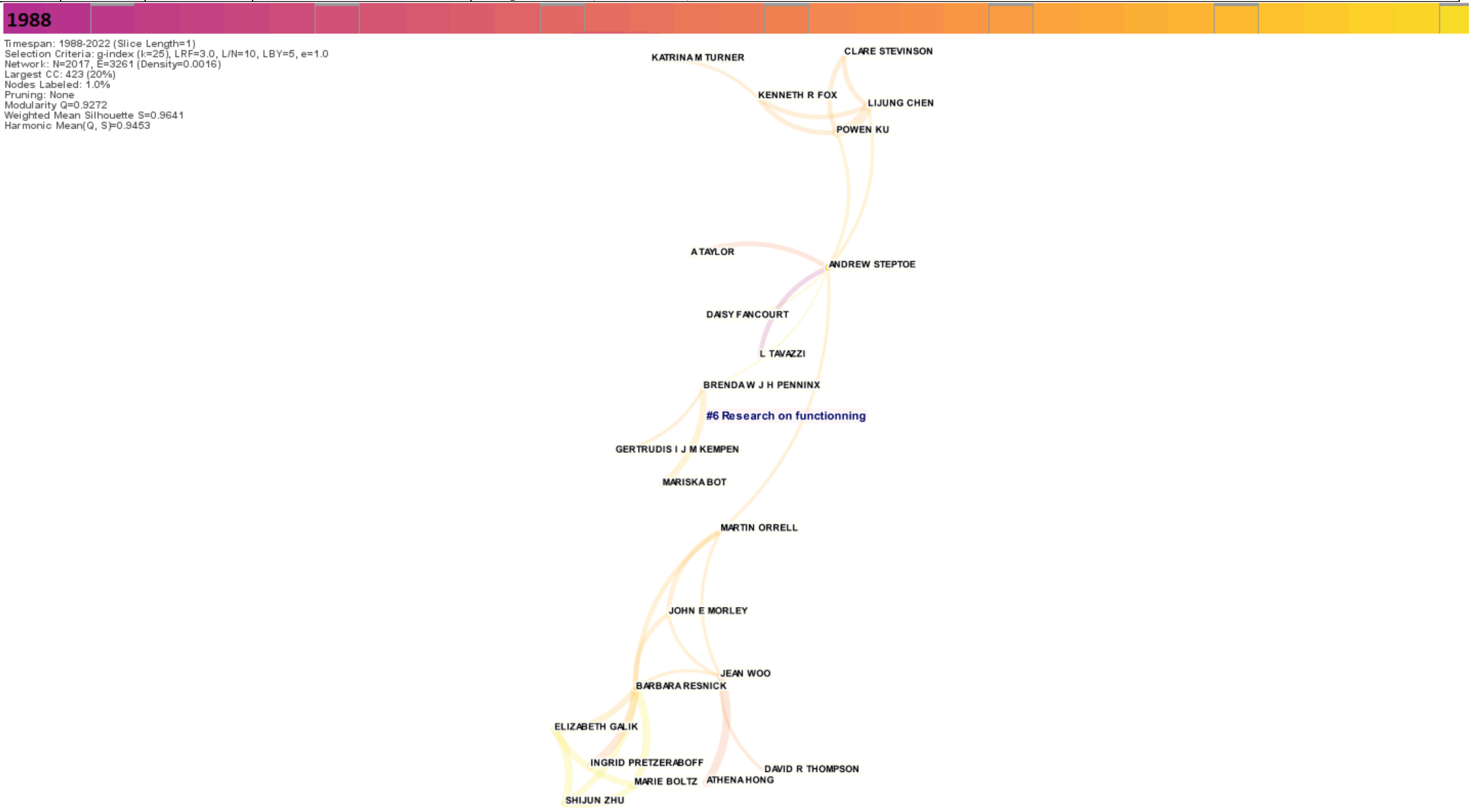

9	20	1	2018	older persons (12.52, 0.001); midlife women (12.52, 0.001); depression (9.3, 0.005); physical activity (8.84, 0.005); prospective (8.79, 0.005)

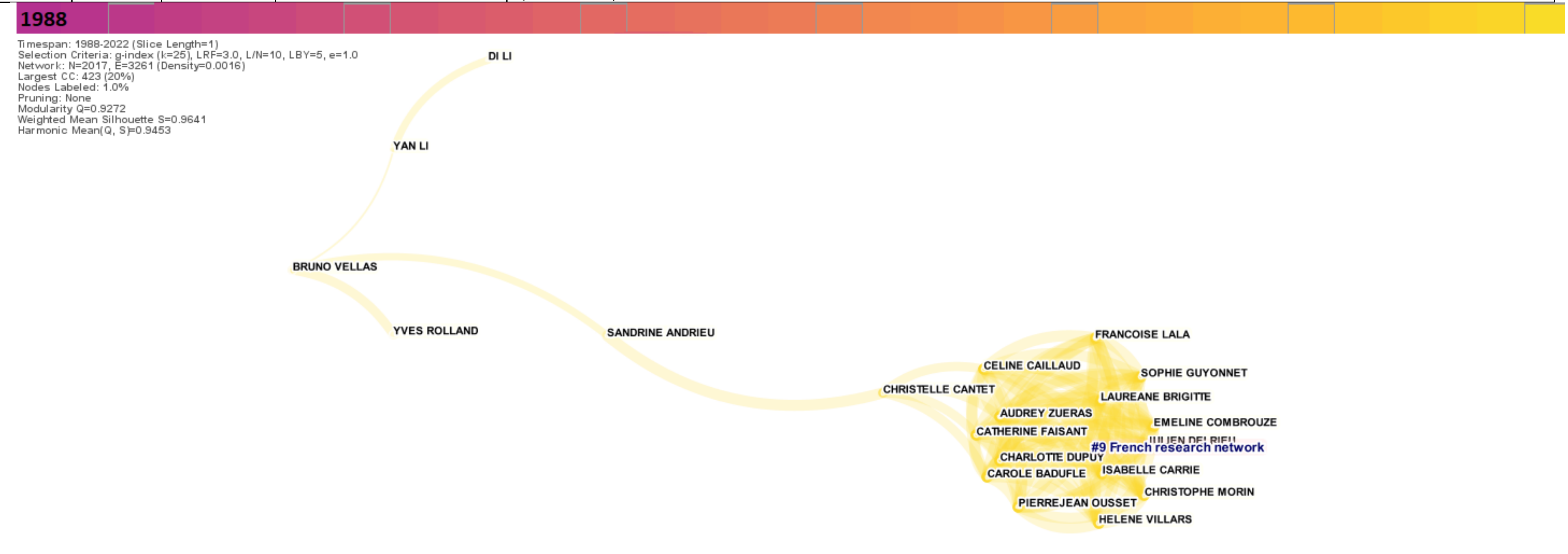

11	16	1	2015	cognitive functions (17.21, 1.0E-4); aerobic exercise (15.46, 1.0E-4); gut microbiota (8.57, 0.005); randomized controlled trials (8.57, 0.005); brain activity (8.57, 0.005)

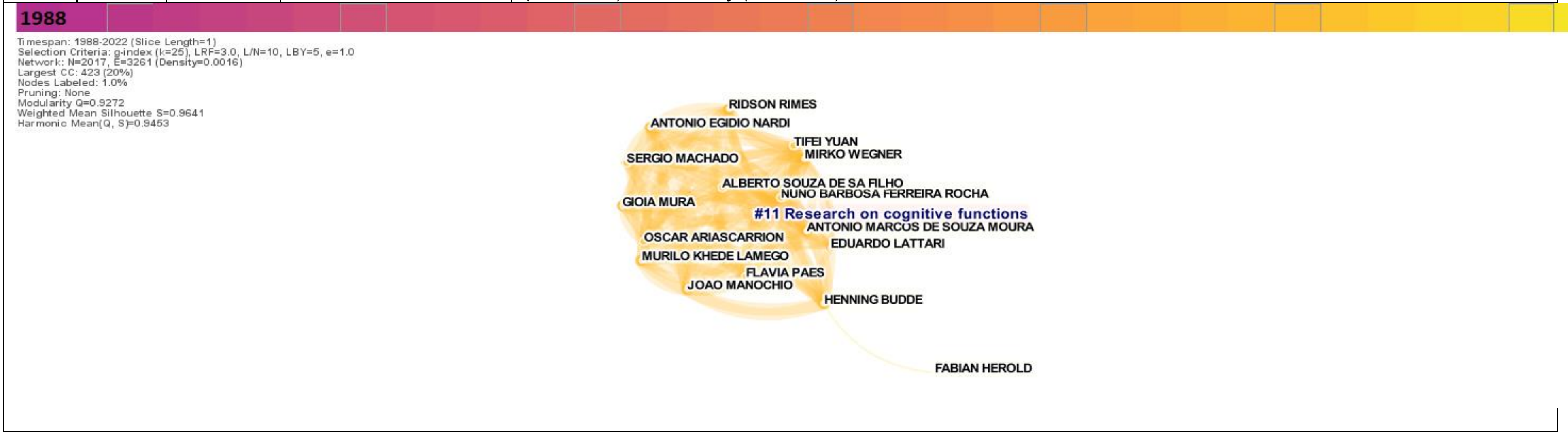

13	13	0.997	2011	japan (22.65, 1.0E-4); social capital (15.06, 0.001); work (15.06, 0.001); older people (14.47, 0.001); physical activity (7.91, 0.005)

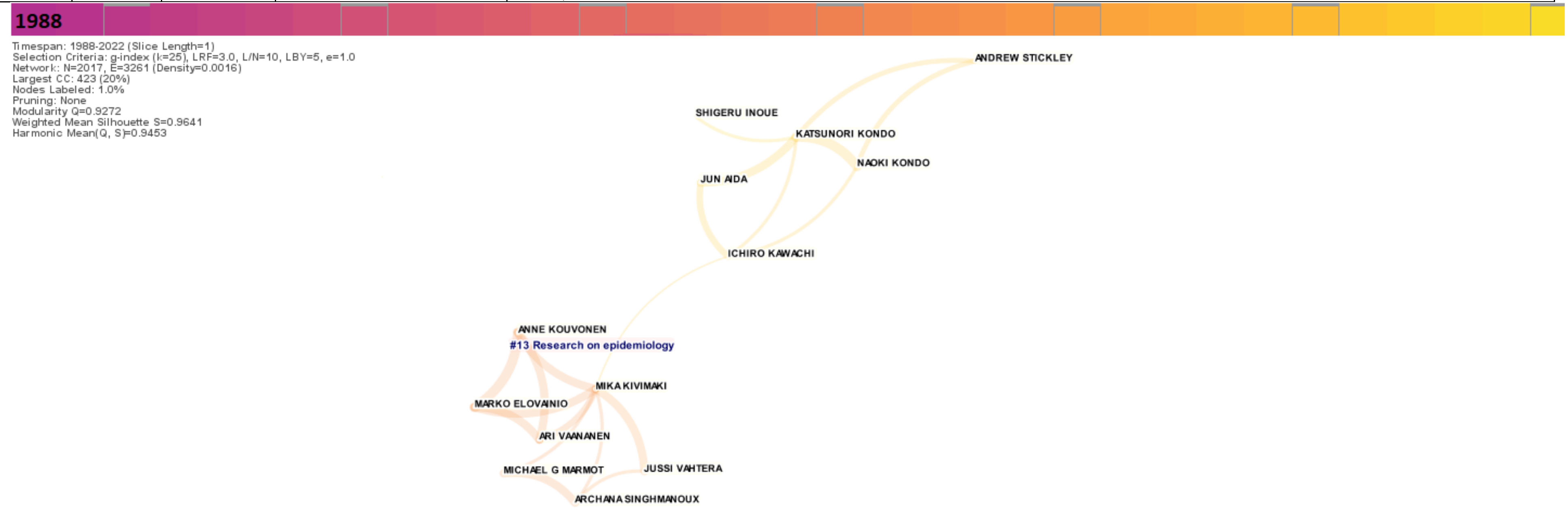

14	13	0.992	2012	psychiatric epidemiology (12.07, 0.001); adiposity (12.07, 0.001); body composition (12.07, 0.001); inflammation (11.6, 0.001); diet (11.34, 0.001)

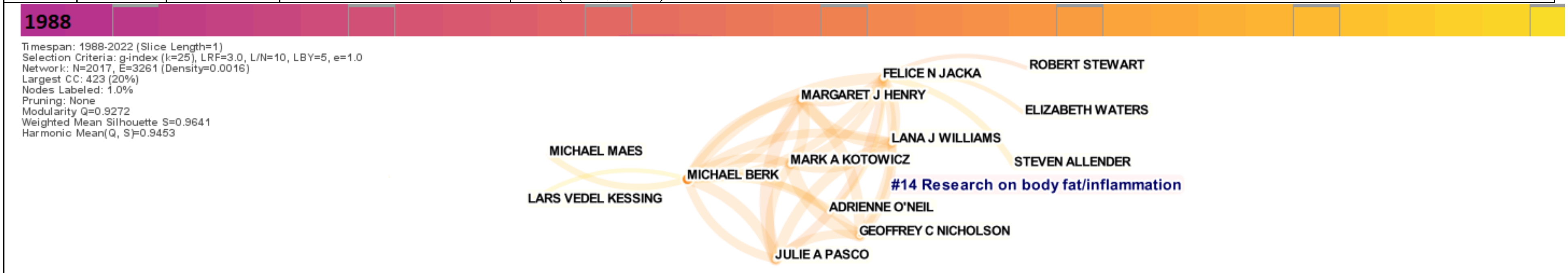

20	9	0.998	2019	amyloid-β (8.07, 0.005); functional decline (8.07, 0.005); antidepressant (8.07, 0.005); quarantine (8.07, 0.005); concussion (8.07, 0.005)

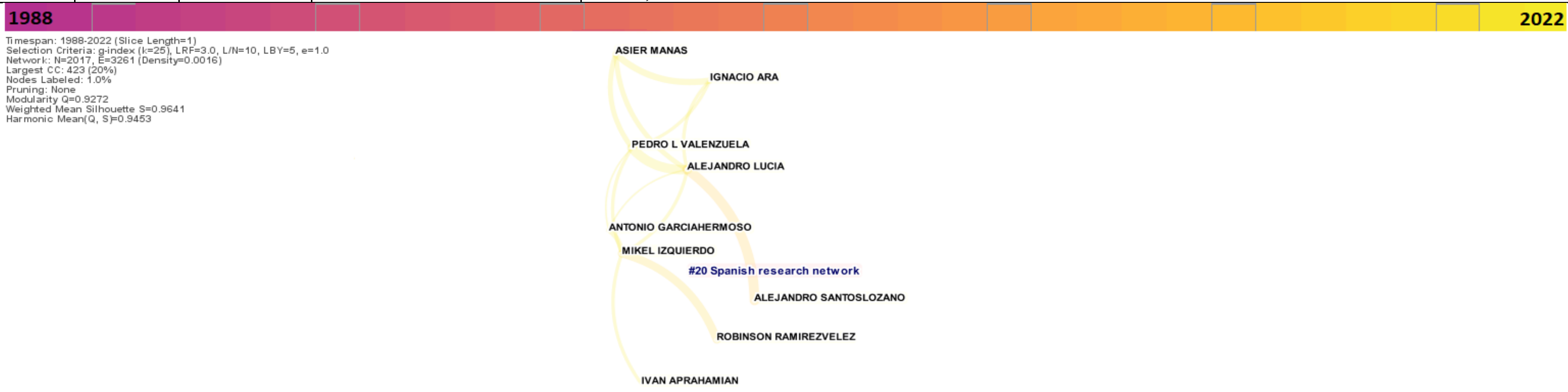

33	7	1	2009	acute leukemia survivors (18.23, 1.0E-4); systematic review (18.23, 1.0E-4); patient-reported symptoms (18.23, 1.0E-4); acute leukemia (18.23, 1.0E-4); symptoms (14.44, 0.001)

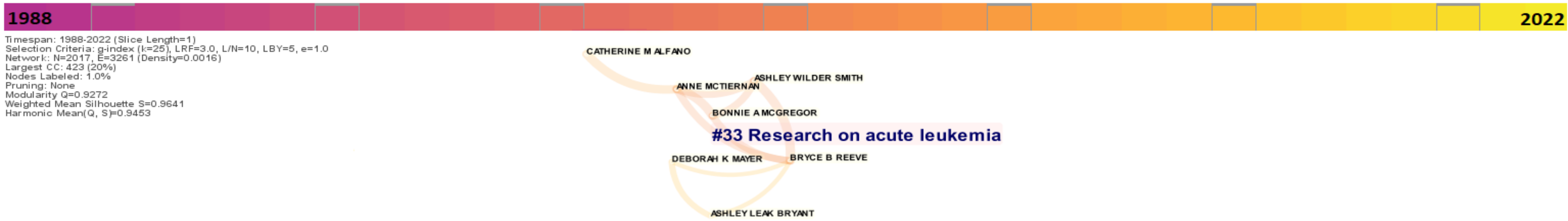

37	6	0.996	2003	exercise therapy (10.88, 0.001); mental disorders (9.84, 0.005); physical fitness (6.33, 0.05); physical activity (0.43, 1.0); exercise (0.3, 1.0)

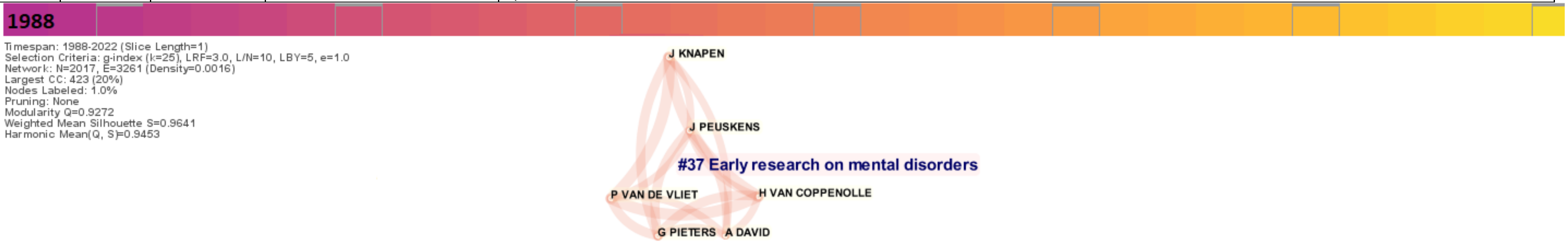

Supplementary Figure 9. Author co-citation network detail (2016-2021)

Author co-citation network (2016-2021)

Author co-citation network with highlights of burstness (2016-2022)

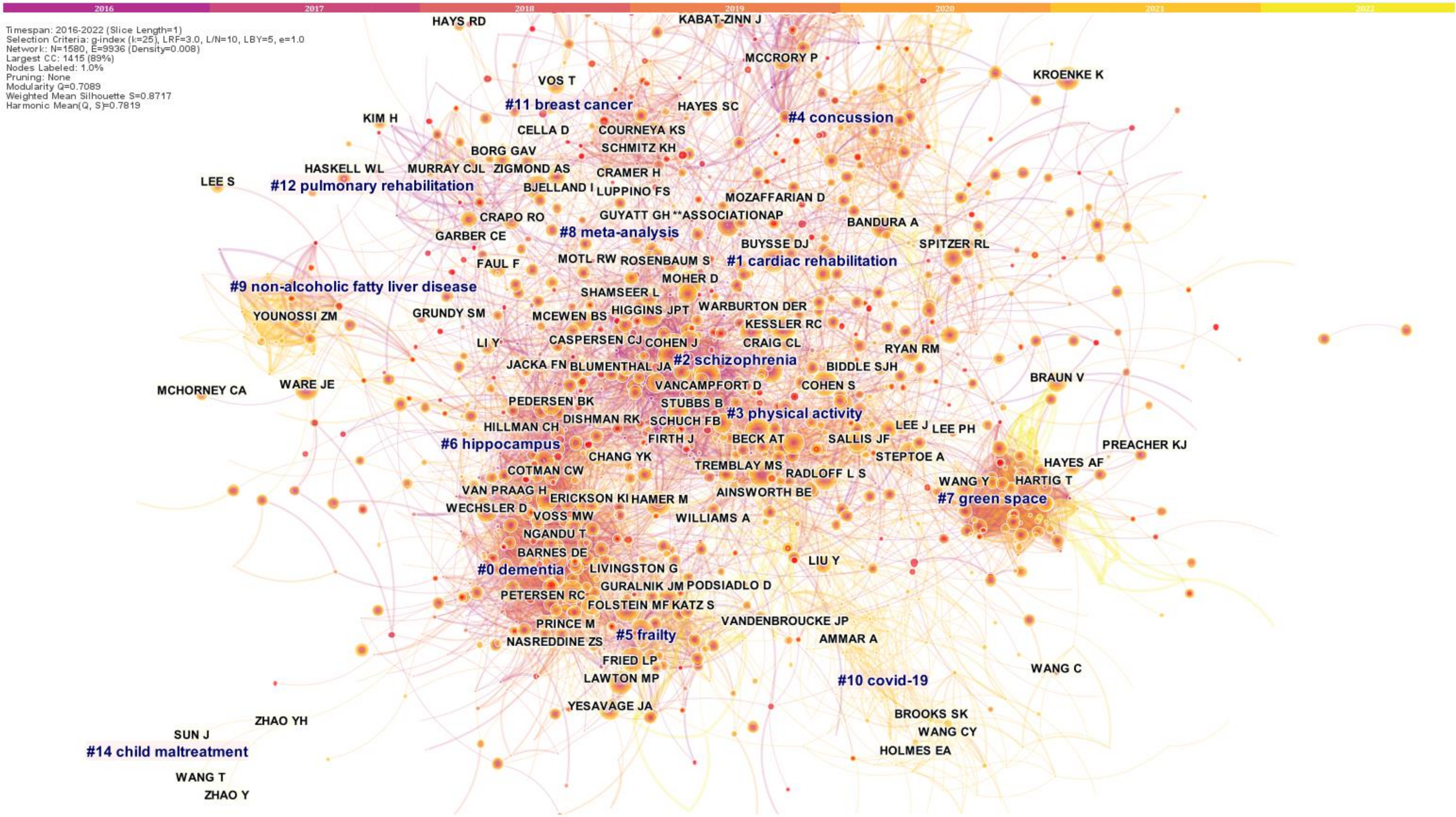

Author co-citation network with highlights of cluster (2016-2022)

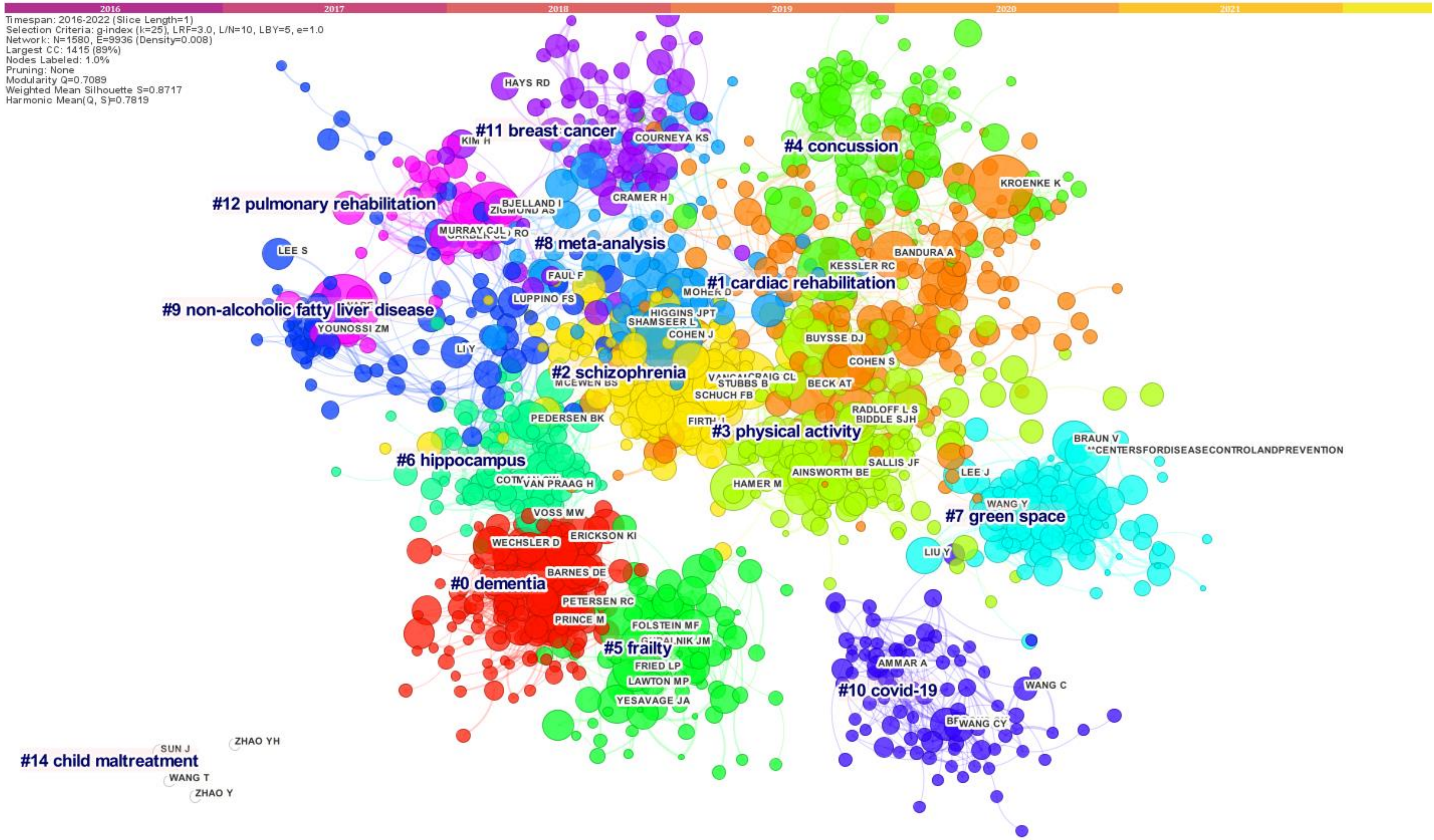

Author co-citation network with time map view (2016-2022)

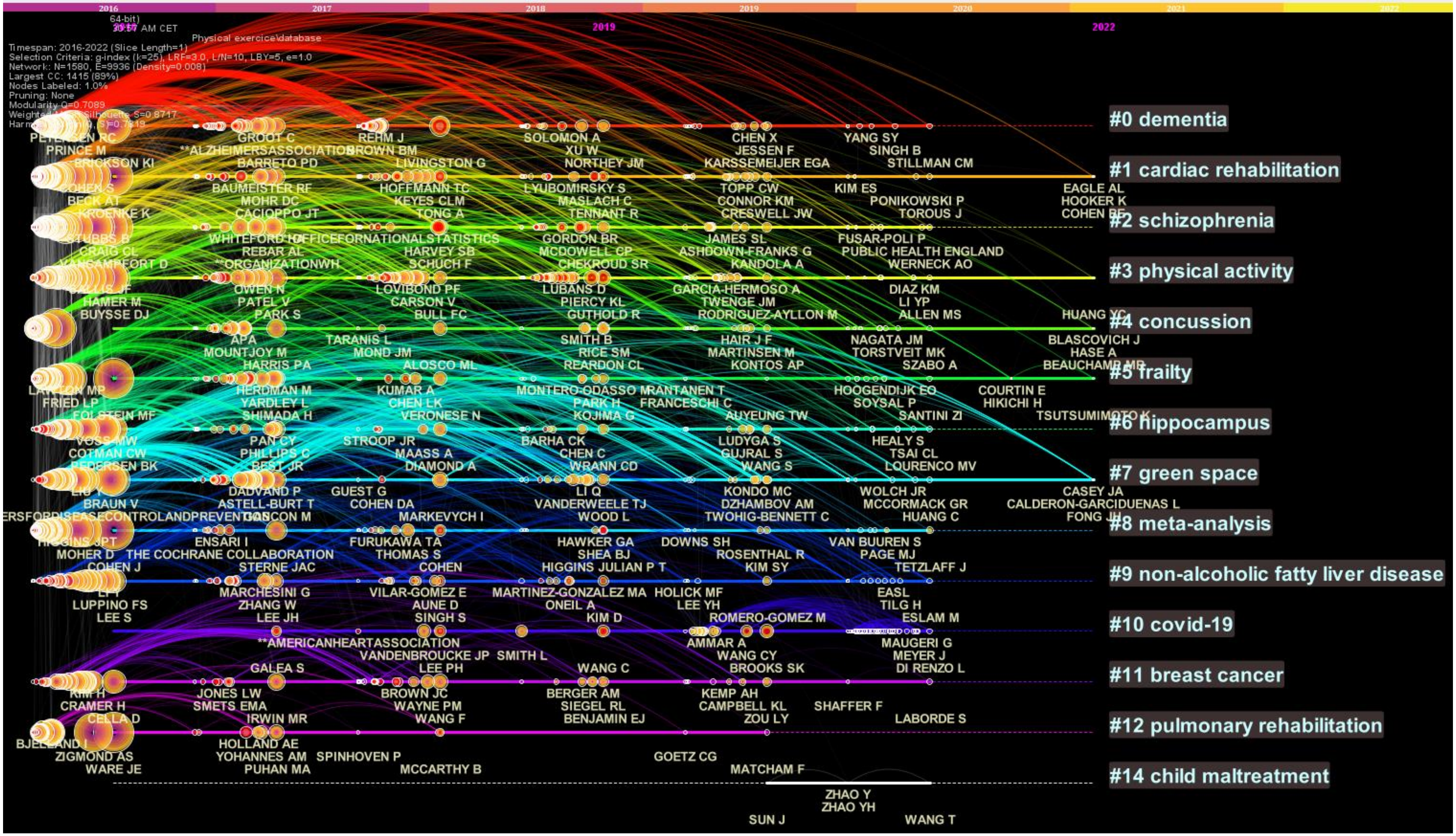

0	184	0.832	2017	**dementia** (914.53, 1.0E-4); alzheimers disease (535.78, 1.0E-4); mild cognitive impairment (424.79, 1.0E-4); cognition (275.01, 1.0E-4); cognitive reserve (178.85, 1.0E-4)

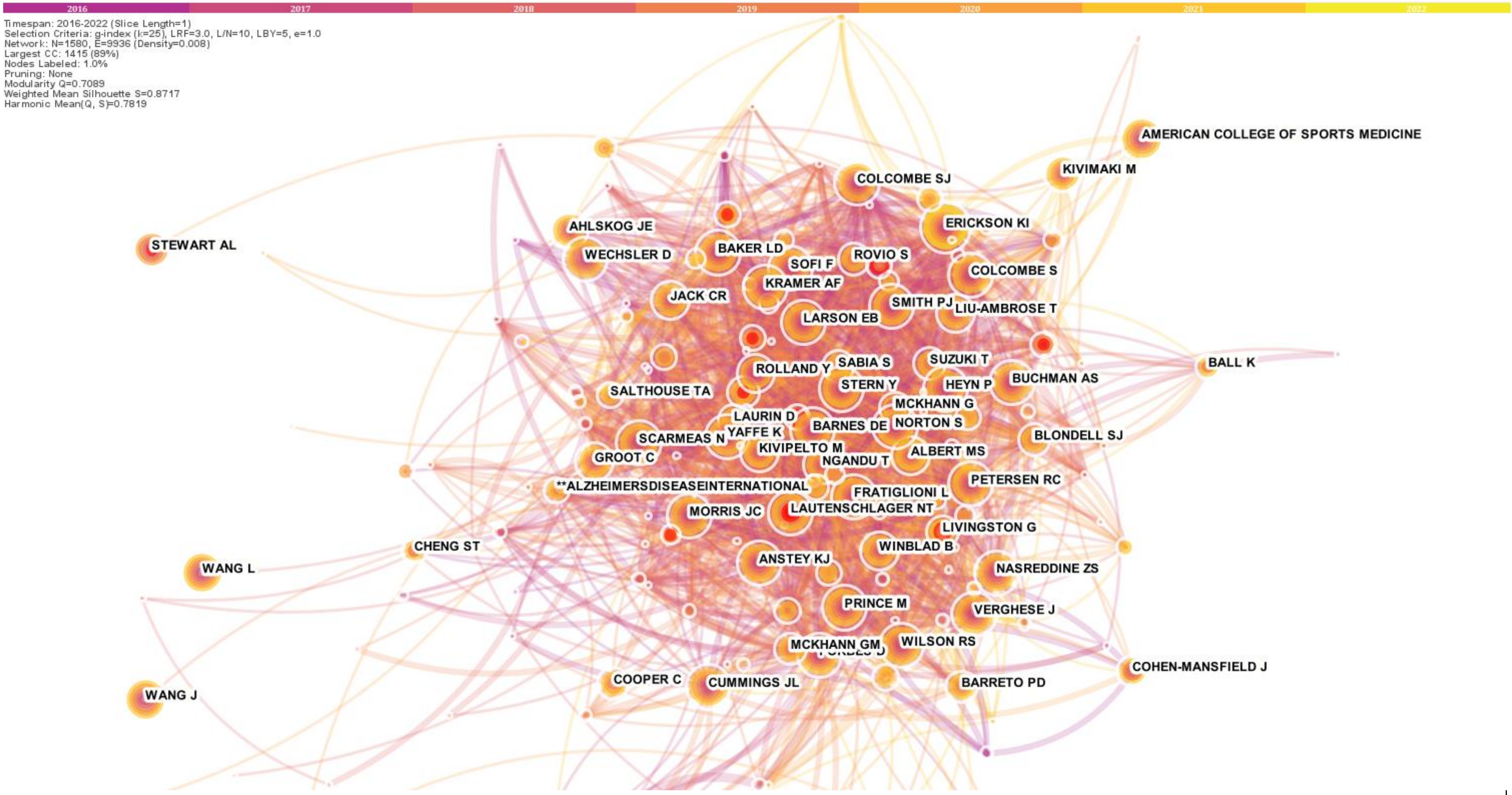

1	167	0.779	2017	**cardiac rehabilitation** (118.12, 1.0E-4); positive psychology (77.17, 1.0E-4); loneliness (72.83, 1.0E-4); cognition (64.02, 1.0E-4); dementia (58.71, 1.0E-4)

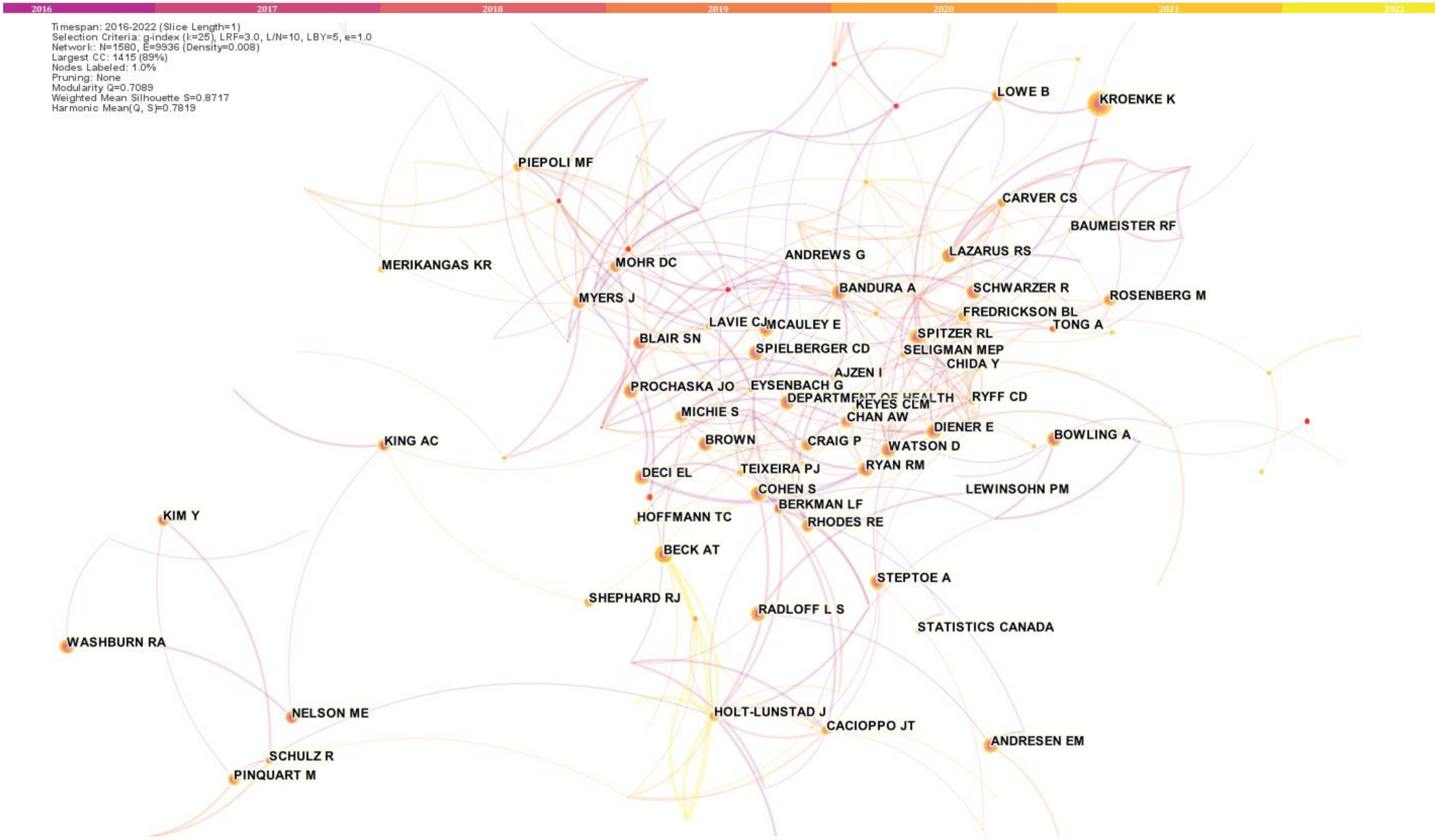

2	165	0.834	2016	**schizophrenia** (530.89, 1.0E-4); depression (194.01, 1.0E-4); psychosis (170.28, 1.0E-4); dementia (119.18, 1.0E-4); bipolar disorder (112.25, 1.0E-4)

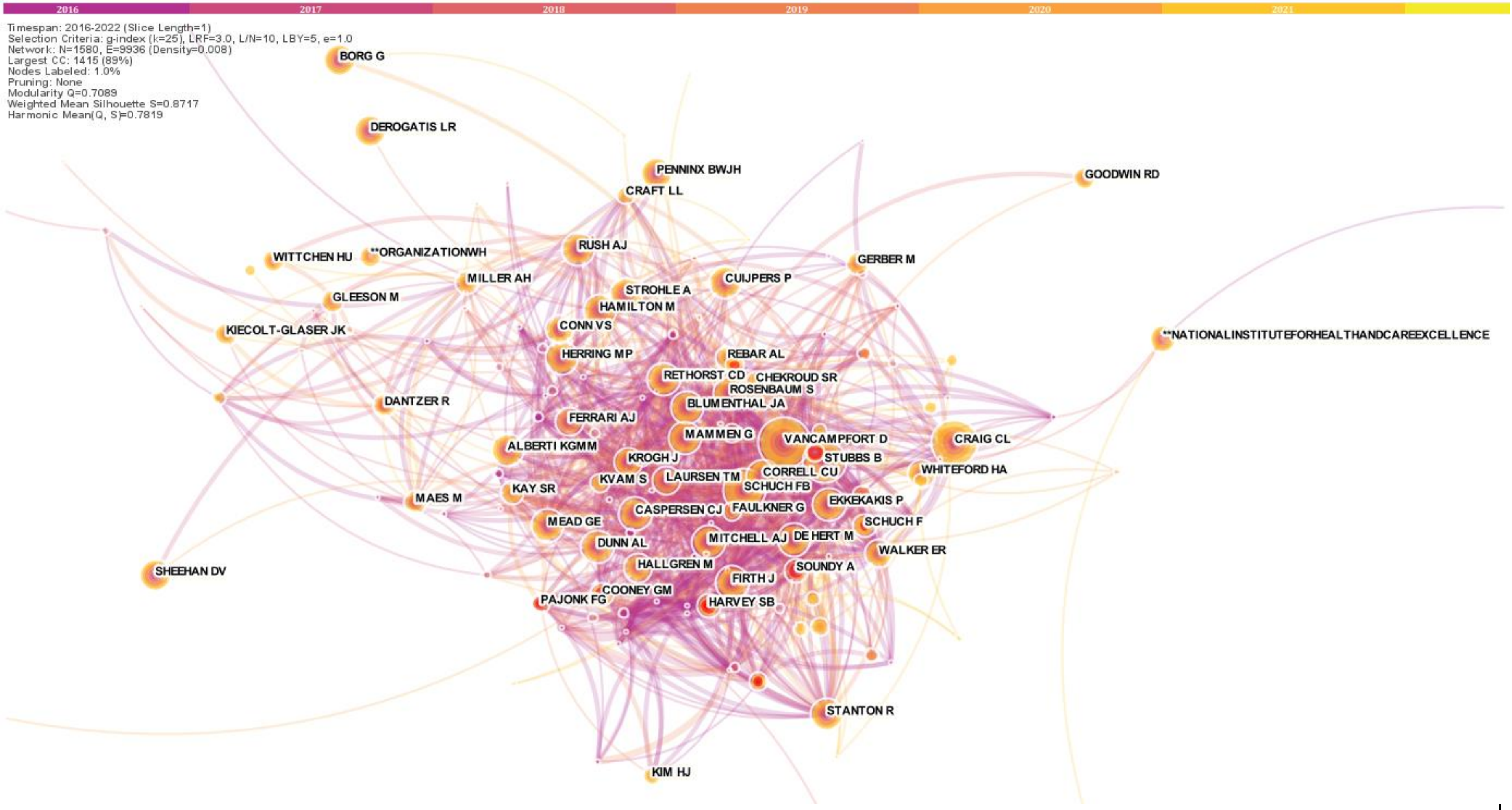

3	162	0.852	2017	**physical activity** (400.54, 1.0E-4); sedentary behavior (230.8, 1.0E-4); sleep (150.36, 1.0E-4); sedentary behaviour (126.76, 1.0E-4); adolescents (117.25, 1.0E-4)

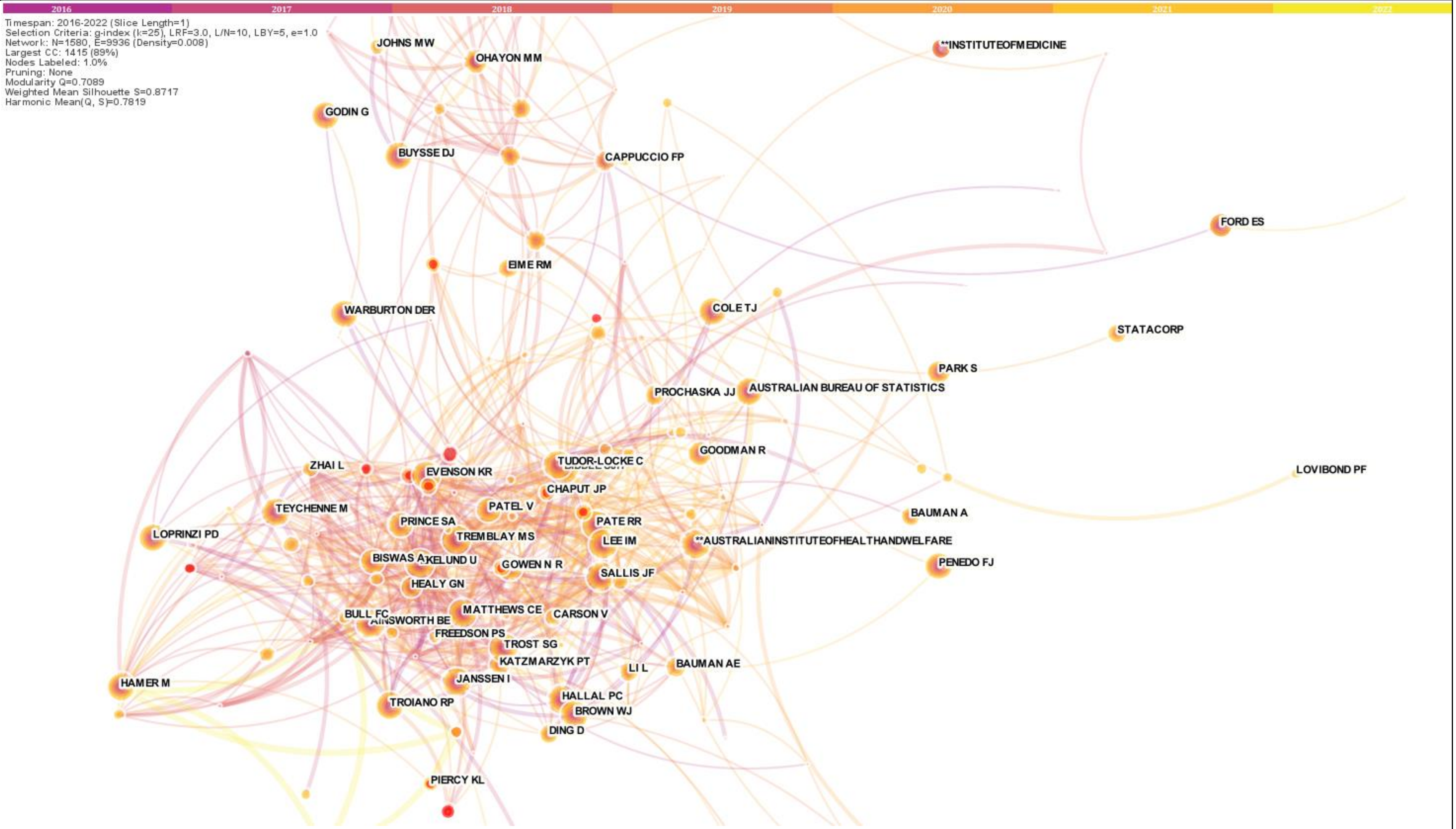

4	102	0.915	2018	**concussion** (548.62, 1.0E-4); eating disorders (407.79, 1.0E-4); mild traumatic brain injury (261.92, 1.0E-4); anorexia nervosa (261, 1.0E-4); disordered eating (218.5, 1.0E-4)

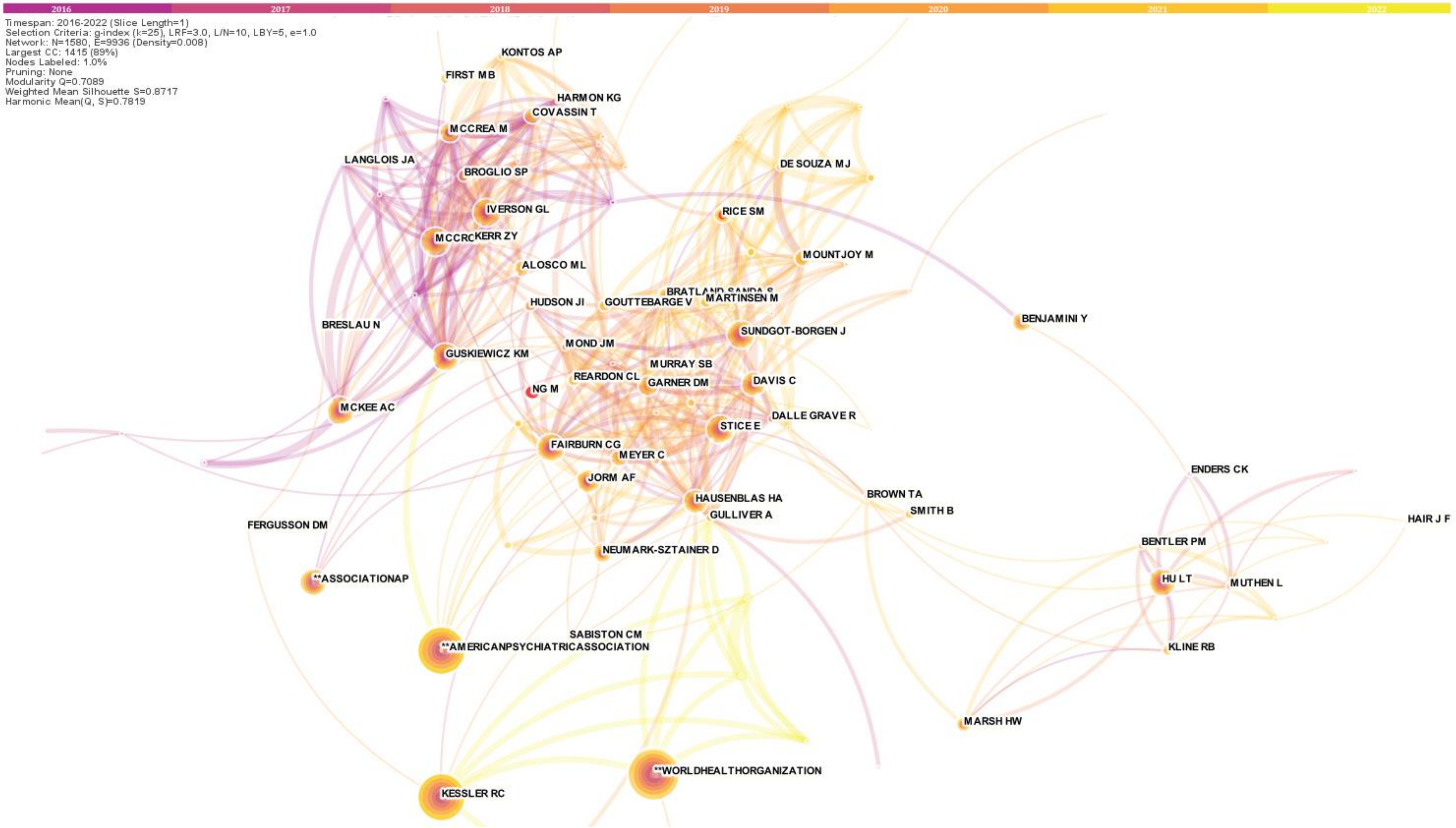

5	93	0.86	2017	**frailty** (742.5, 1.0E-4); sarcopenia (223.83, 1.0E-4); falls (171.54, 1.0E-4); older adults (160.35, 1.0E-4); frail elderly (131.24, 1.0E-4)

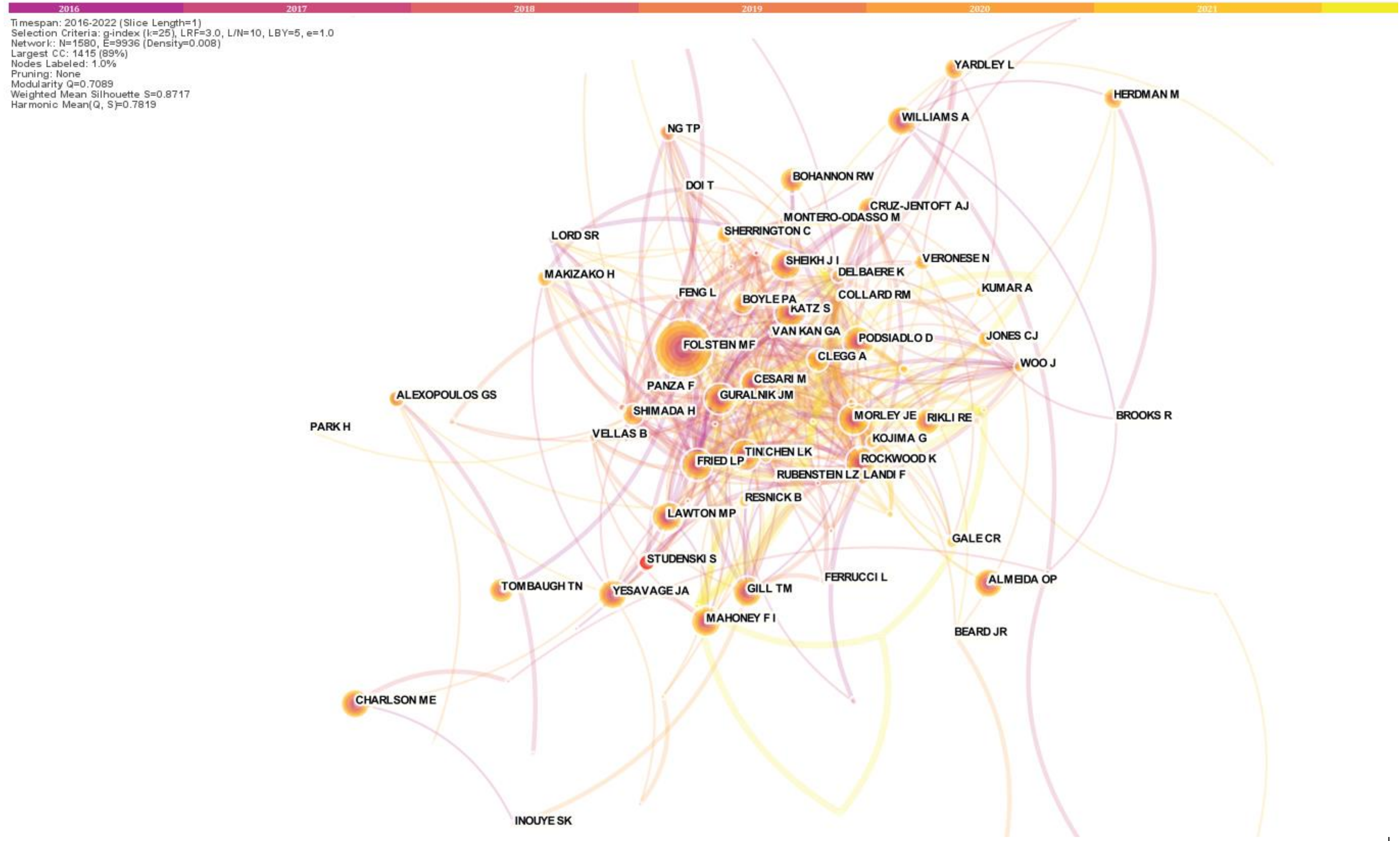

6	92	0.875	2017	**hippocampus** (215.69, 1.0E-4); bdnf (156.13, 1.0E-4); neurogenesis (122.88, 1.0E-4); brain-derived neurotrophic factor (108.61, 1.0E-4); environmental enrichment (106.36, 1.0E-4)

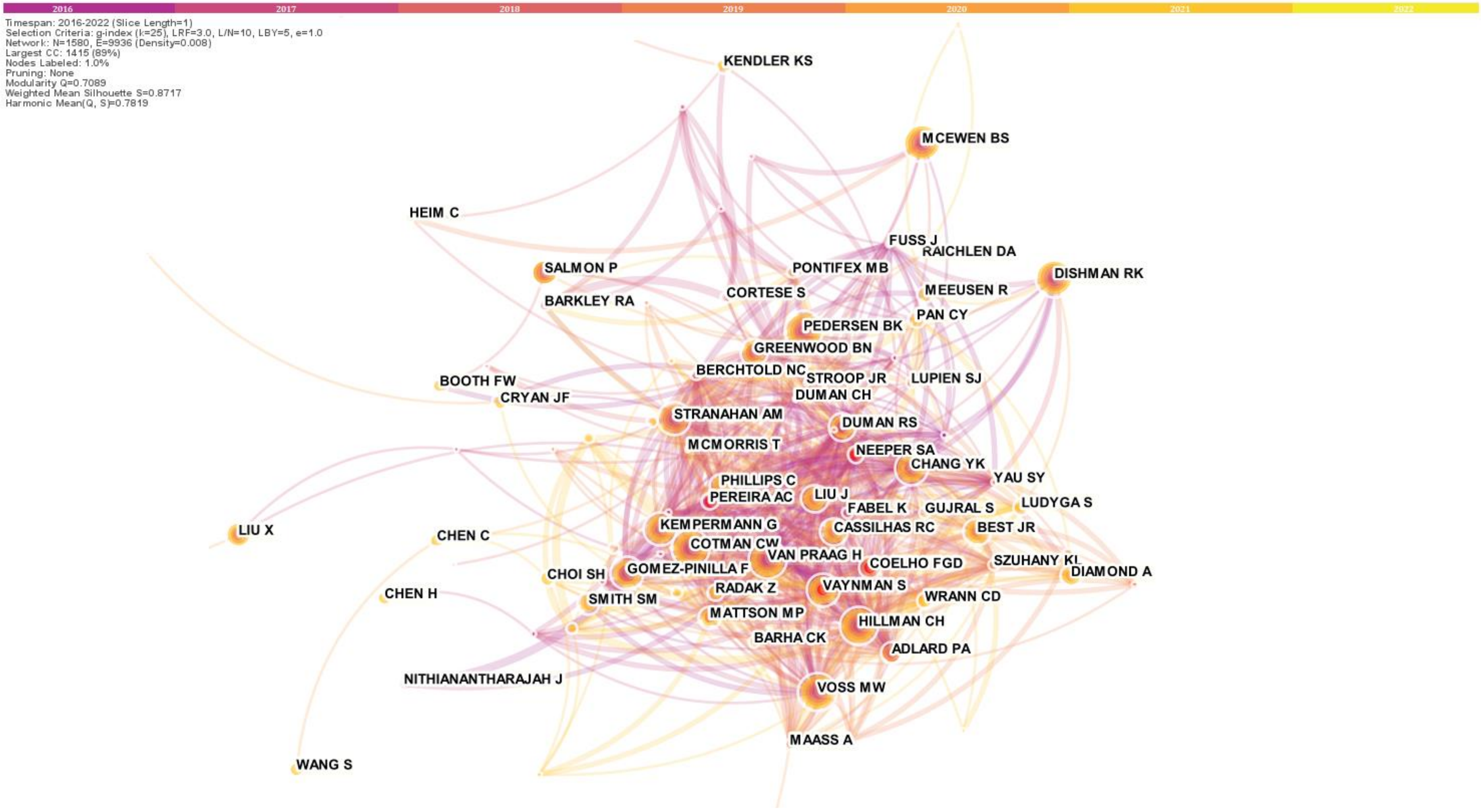

7	92	0.977	2018	**green space** (325.23, 1.0E-4); nature (220.85, 1.0E-4); built environment (154.82, 1.0E-4); greenspace (137.44, 1.0E-4); urban green space (128.27, 1.0E-4)

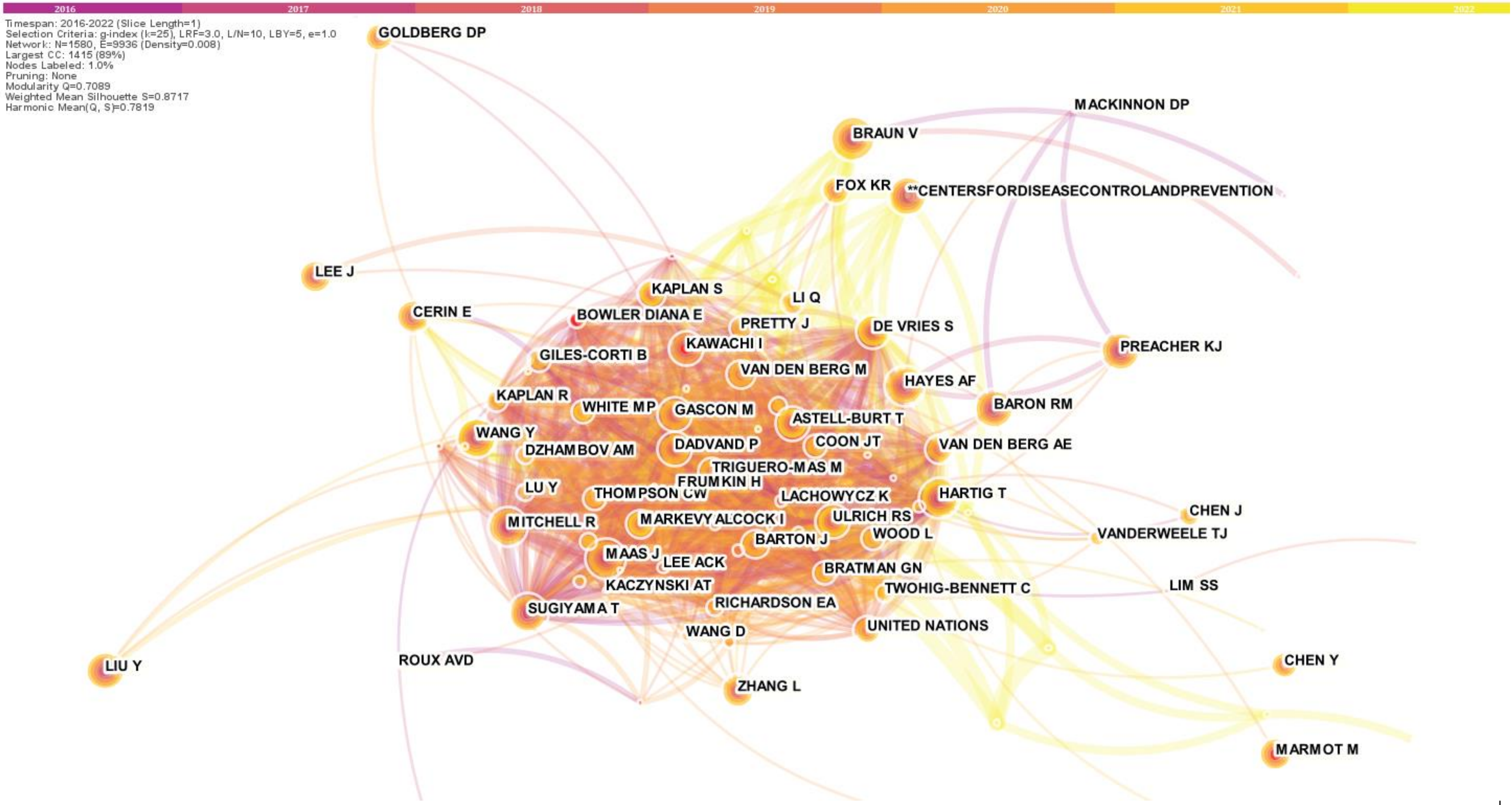

8	86	0.898	2017	**meta-analysis** (348.92, 1.0E-4); multiple sclerosis (339.7, 1.0E-4); systematic review (238.59, 1.0E-4); fibromyalgia (177.77, 1.0E-4); chronic pain (108.6, 1.0E-4)

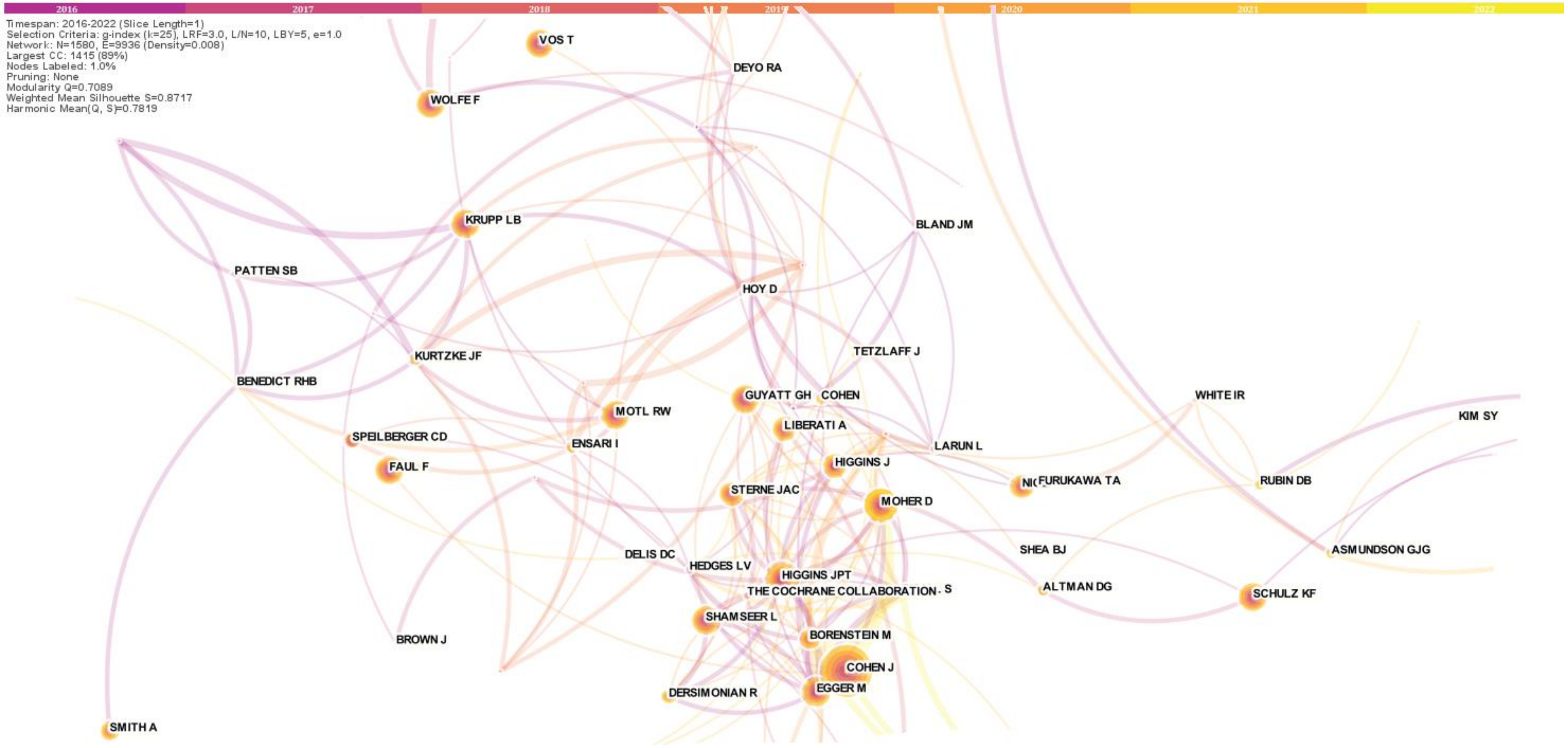

9	86	0.903	2017	**non-alcoholic fatty liver disease** (491.56, 1.0E-4); nafld (277.11, 1.0E-4); obesity (202.1, 1.0E-4); diet (165.84, 1.0E-4); mediterranean diet (143.89, 1.0E-4)

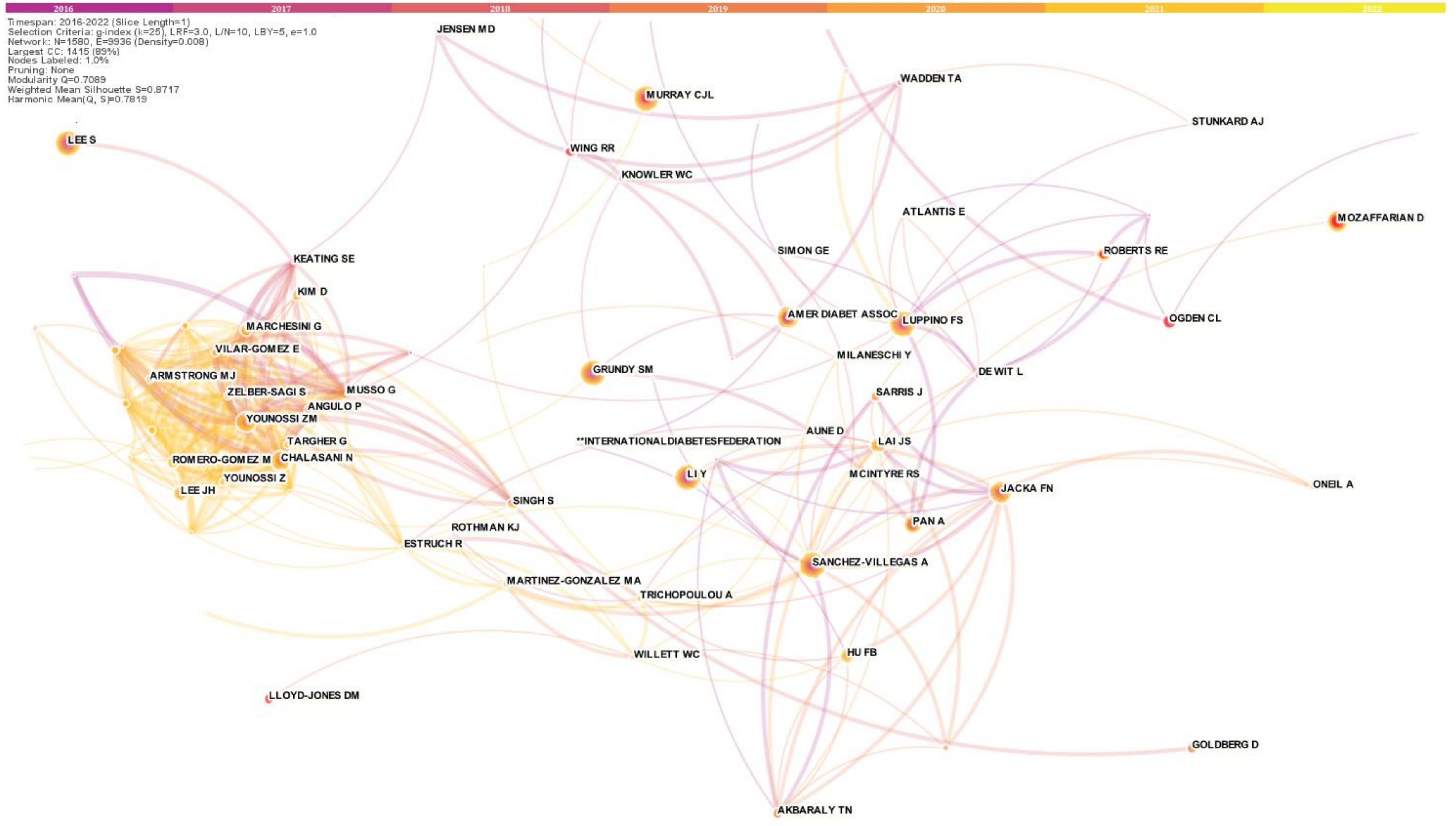

10	82	0.946	2020	**covid-19** (1047.15, 1.0E-4); pandemic (222.87, 1.0E-4); lockdown (154.22, 1.0E-4); coronavirus (137.34, 1.0E-4); quarantine (76.73, 1.0E-4)

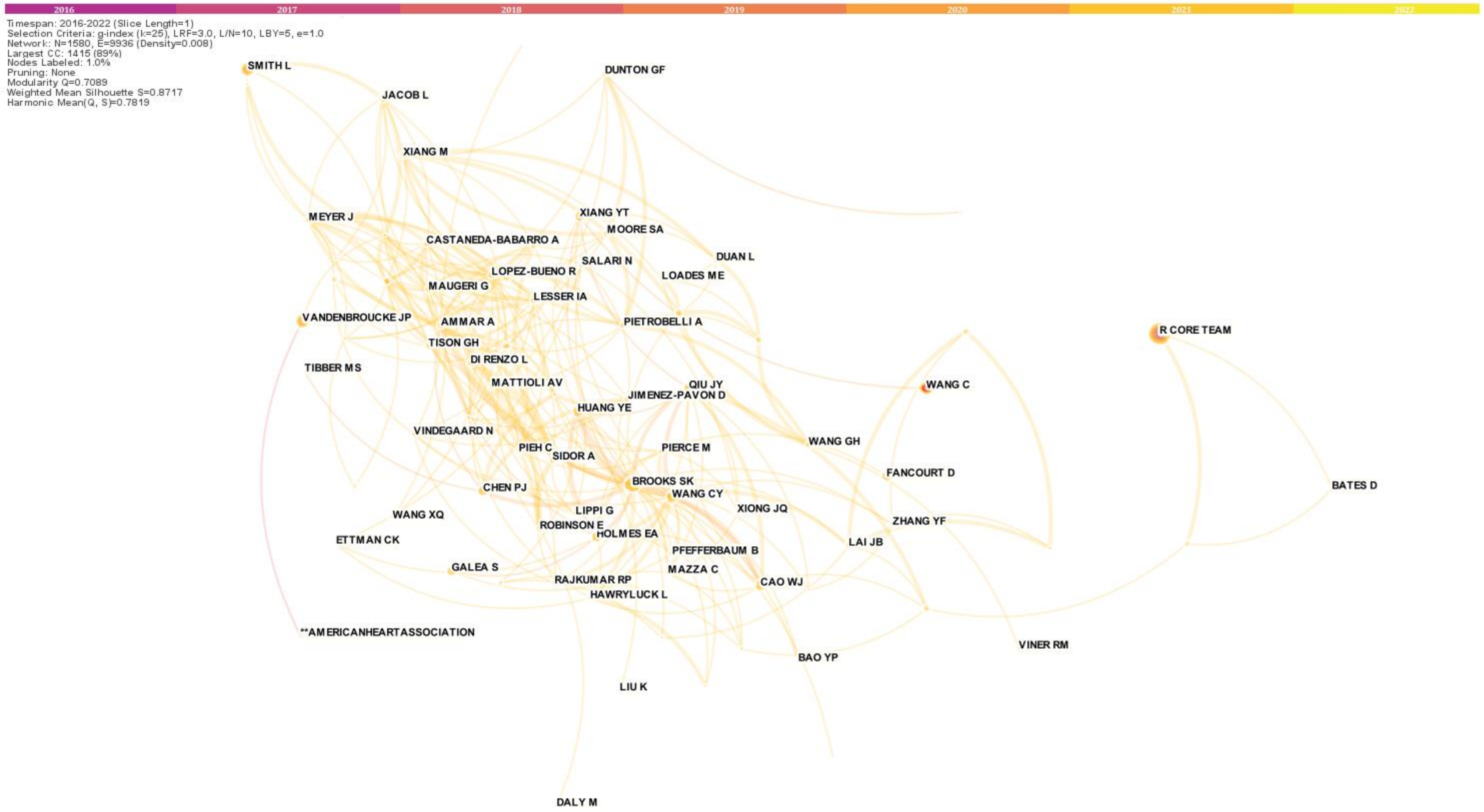

11	67	0.937	2017	**breast cancer** (289.07, 1.0E-4); mindfulness (218.48, 1.0E-4); tai chi (177.98, 1.0E-4); cancer (171.98, 1.0E-4); chemotherapy (151.24, 1.0E-4)

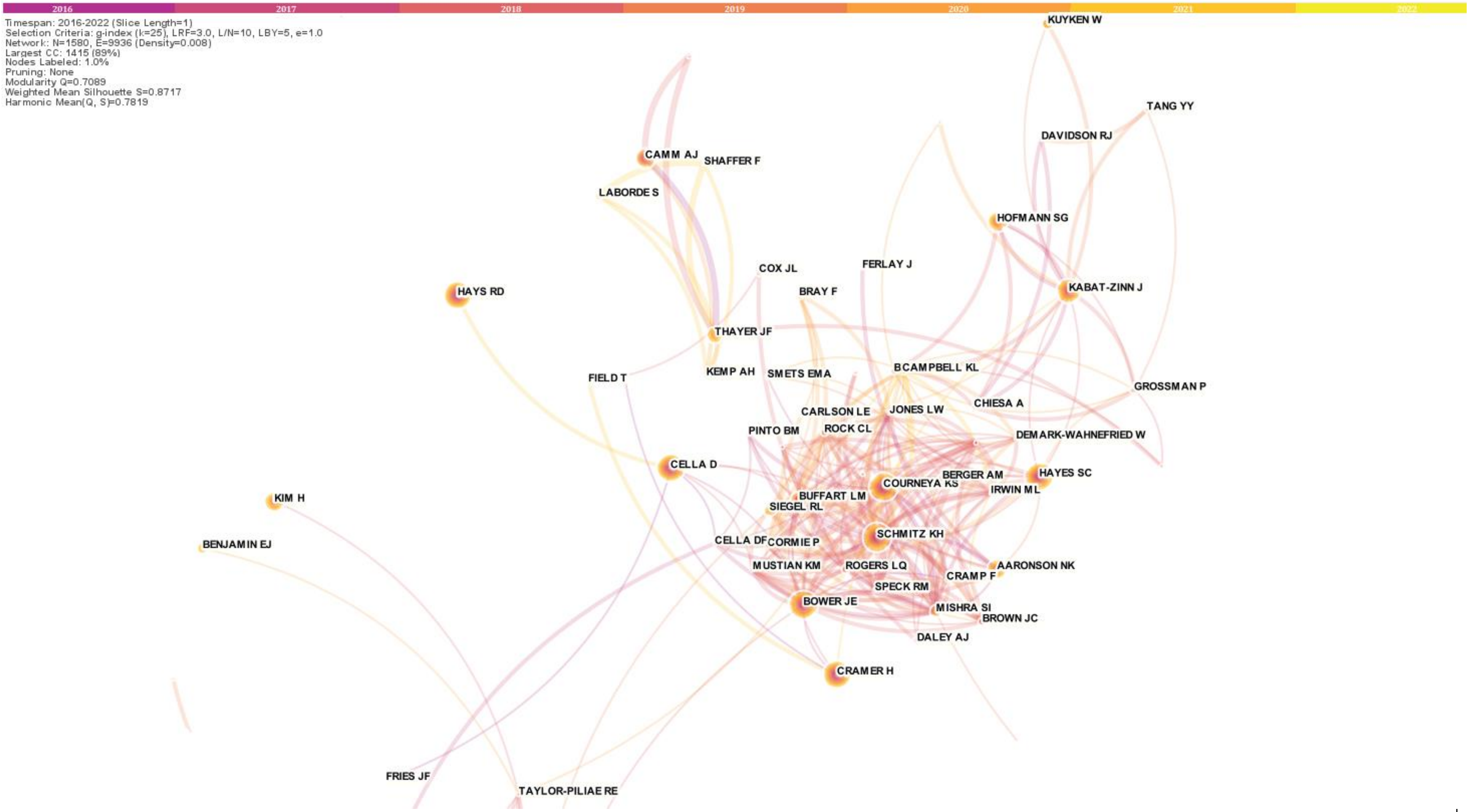

12	33	0.958	2016	**pulmonary rehabilitation** (452.36, 1.0E-4); copd (410.05, 1.0E-4); chronic obstructive pulmonary disease (312.63, 1.0E-4); quality of life (273.48, 1.0E-4); dyspnea (142.38, 1.0E-4)

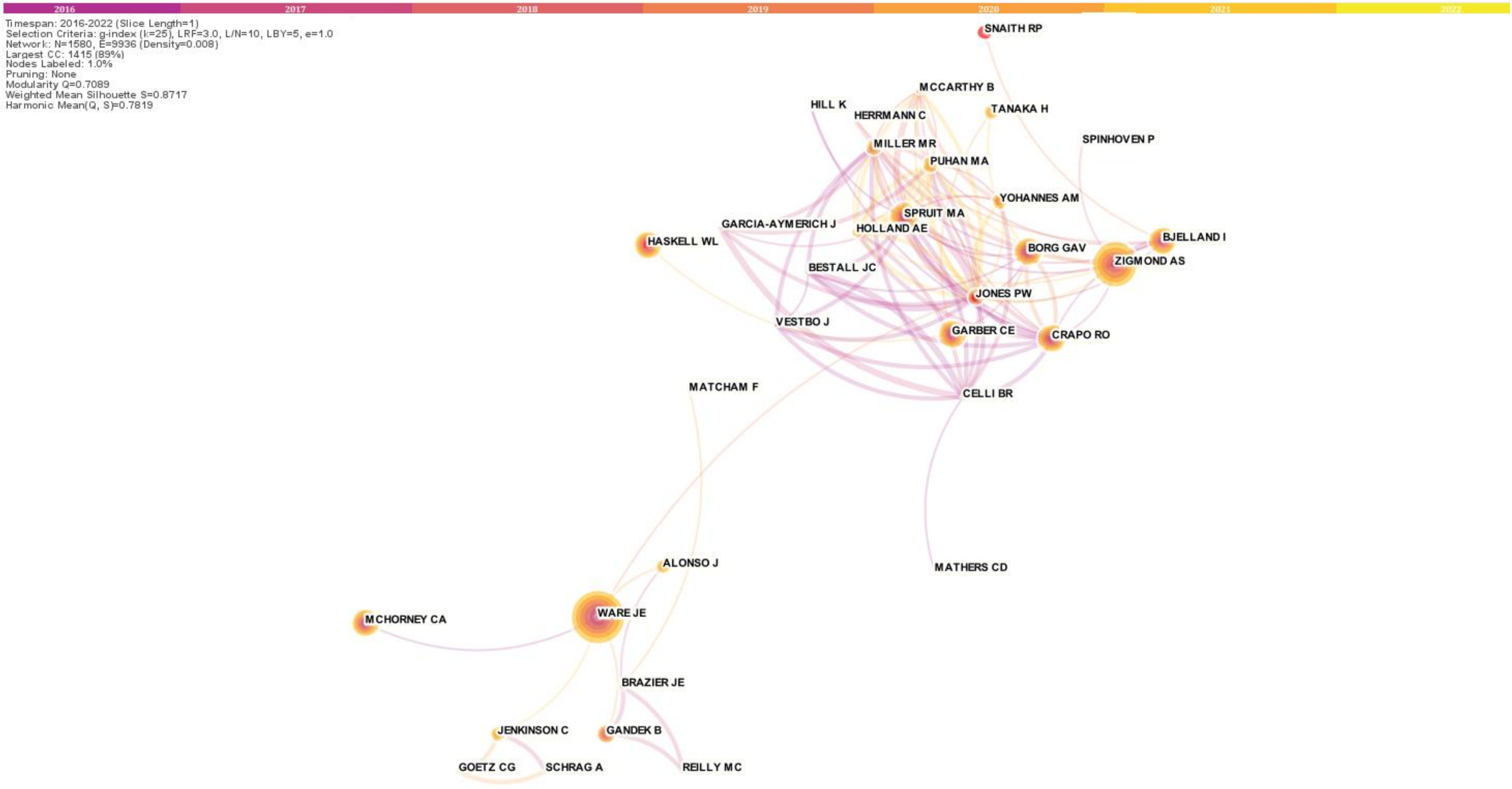

14	4	0.998	2020	**child maltreatment** (12.13, 0.001); gut (12.13, 0.001); microbiota-gut-brain axis (12.13, 0.001); framingham risk score (12.13, 0.001); tri-partite synapses (12.13, 0.001)

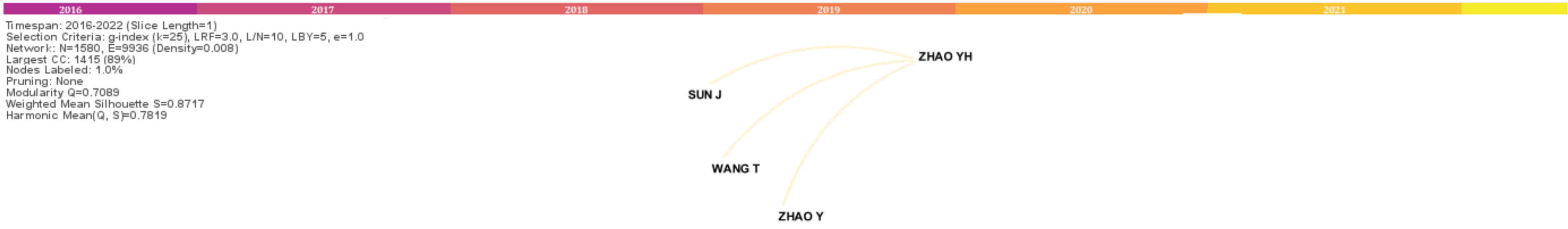

Supplementary Figure 10. Source growths for the top 10 Journals based on the number of articles in our dataset (1988-2021 time period)

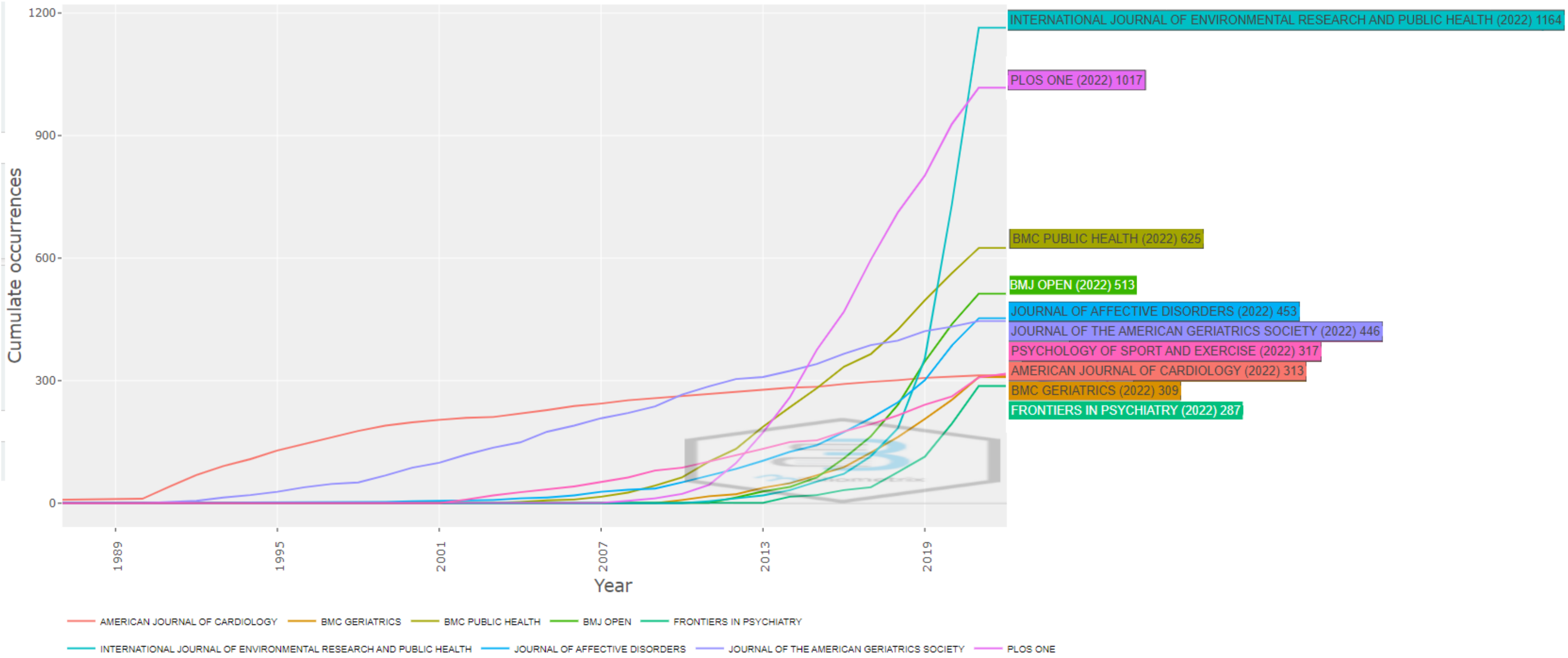

Supplementary Figure 11. Journal co-citation network analysis (a), with corresponding clusters (b) (2016-2021)

A

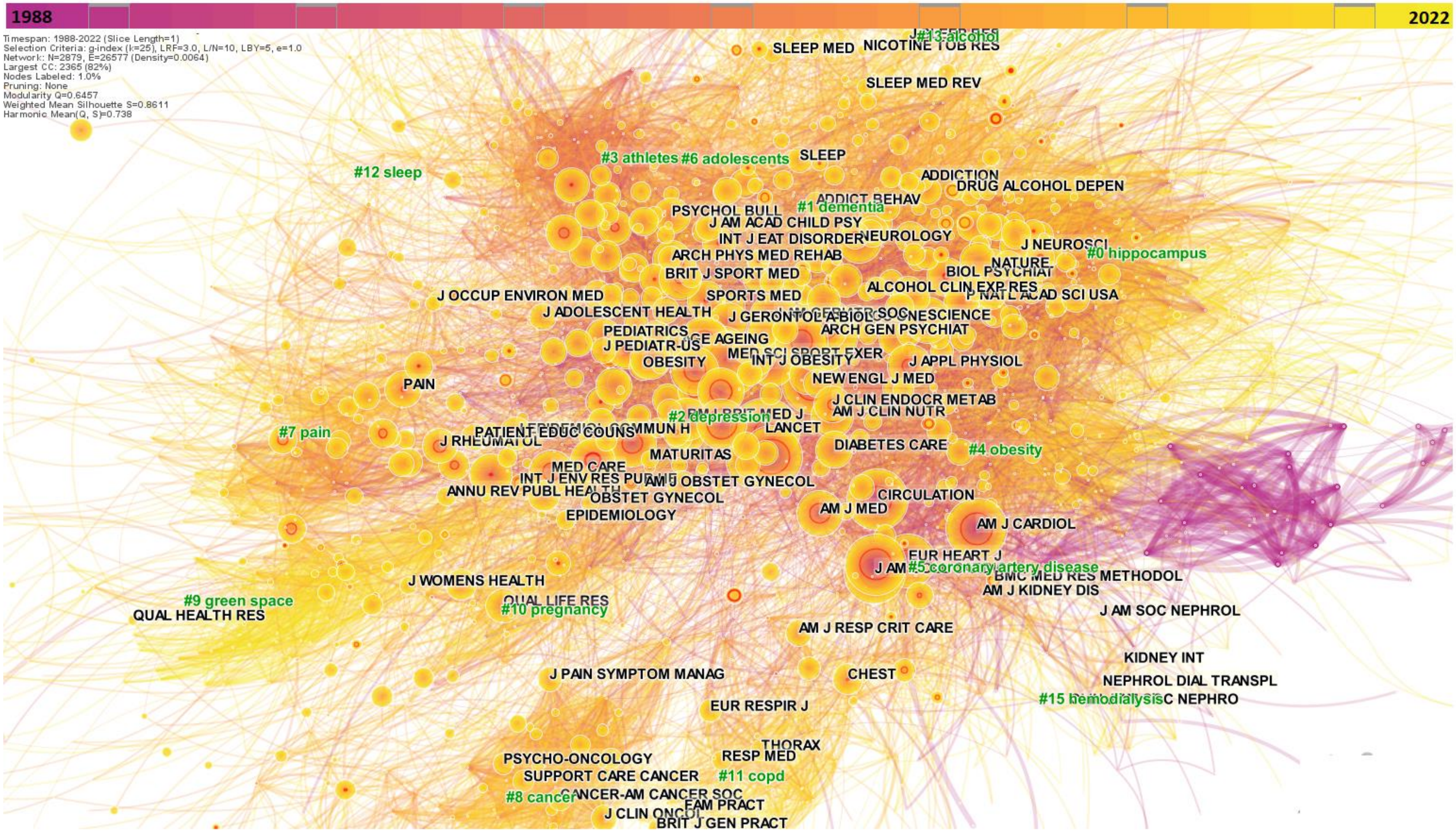

B

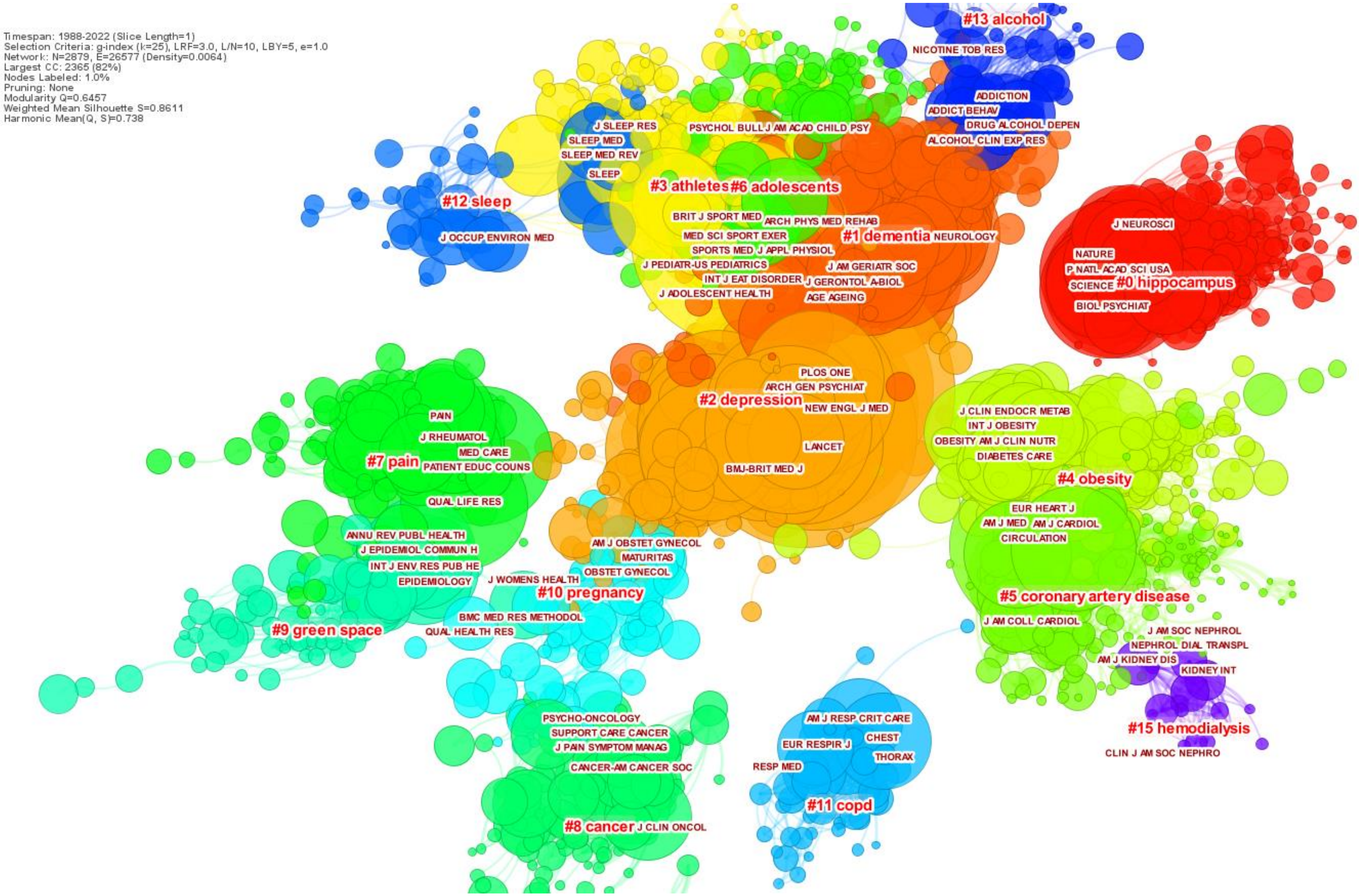
Timespan: 1988-2022 (Slice Length=1)
Selection Criteria: g-index (k=25), LRF=3.0, L/N=10, LBY=5, e=1.0
Network: N=2879, E=26577 (Density=0.0064)
Largest CC: 2365 (82%)
Nodes Labeled: 1.0%
Pruning: None
Modularity Q=0.6457
Weighted Mean Silhouette S=0.8611
Harmonic Mean(Q, S)=0.738
#13 alcohol
NICOTINE TOB RES
ADDICTION
ADDICT BEHAV
DRUG ALCOHOL DEPEN
ALCOHOL CLIN EXP RES
J SLEEP RES
SLEEP MED
SLEEP MED REV
SLEEP
PSYCHOL BULL
J AM ACAD CHILD PSY
#12 sleep
J OCCUP ENVIRON MED
#3 athletes
#6 adolescents
BRIT J SPORT MED
ARCH PHYS MED REHAB
MED SCI SPORT EXER
#1 dementia
NEUROLOGY
SPORTS MED
J APPL PHYSIOL
J PEDIATR-US
PEDIATRICS
J AM GERIATR SOC
INT J EAT DISORDER
J GERONTOL A-BIOL
J ADOLESCENT HEALTH
AGE AGEING
J NEUROSCI
NATURE
P NATL ACAD SCI USA
SCIENCE
#0 hippocampus
BIOL PSYCHIAT
PLOS ONE
ARCH GEN PSYCHIAT
#2 depression
NEW ENGL J MED
LANCET
BMJ-BRIT MED J
PAIN
J RHEUMATOL
MED CARE
#7 pain
PATIENT EDUC COUNS
QUAL LIFE RES
J CLIN ENDOCR METAB
INT J OBESITY
OBESITY
AM J CLIN NUTR
DIABETES CARE
#4 obesity
EUR HEART J
AM J MED
AM J CARDIOL
CIRCULATION
ANNU REV PUBL HEALTH
J EPIDEMIOL COMMUN H
INT J ENV RES PUB HE
EPIDEMIOLOGY
AM J OBSTET GYNECOL
MATURITAS
OBSTET GYNECOL
J WOMENS HEALTH
#10 pregnancy
#5 coronary artery disease
BMC MED RES METHODOL
#9 green space
QUAL HEALTH RES
J AM COLL CARDIOL
J AM SOC NEPHROL
NEPHROL DIAL TRANSPL
AM J KIDNEY DIS
KIDNEY INT
PSYCHO-ONCOLOGY
SUPPORT CARE CANCER
J PAIN SYMPTOM MANAG
CANCER-AM CANCER SOC
AM J RESP CRIT CARE
CHEST
EUR RESPIR J
THORAX
RESP MED
#15 hemodialysis
CLIN J AM SOC NEPHRO
#11 copd
#8 cancer
J CLIN ONCOL
